# Accessing Chiral Pyrrolodiketopiperazines under Organocatalytic Conditions

**DOI:** 10.1021/acs.orglett.2c03924

**Published:** 2022-12-29

**Authors:** Eider Duñabeitia, Aitor Landa, Rosa López, Claudio Palomo

**Affiliations:** Department of Organic Chemistry I, Faculty of Chemistry, University of the Basque Country (UPV-EHU), Manuel de Lardizabal 3, 20018 San Sebastián, Spain

## Abstract

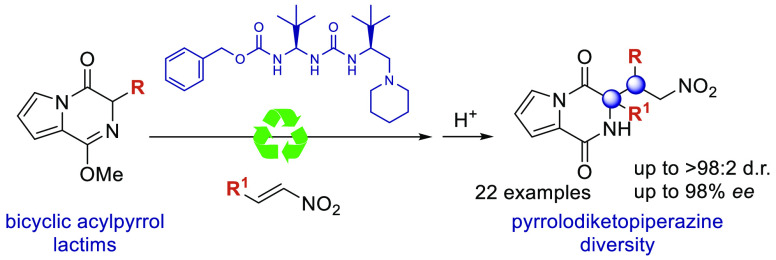

The production of chiral pyrrolodiketopiperazines under organocatalytic conditions demonstrates the capacity of bicyclic acylpyrrol lactims to perform as pronucleophiles in direct carbon–carbon bond forming reactions. The good performance of ureidoaminal-derived Brønsted bases in the Michael addition to nitroolefins affords these heterocyclic scaffolds with high skeleton diversity.

Pyrrolodiketopiperazines and (dihydro)pyrrolopiperazinones are a hybrid class of heterocyclic scaffolds in which the privileged pyrrol and (di)ketopyperazine rings are fused to raise a particular framework that appears within a wide range of bioactive natural products isolated from various sources as fungi, plants, or sponges (Figure 1).^1^ Due to its relatively recent isolation, methods for the construction of these peculiar natural compounds remain somewhat limited, especially in the case of pyrrolodiketopiperazines.

**Figure 1:**
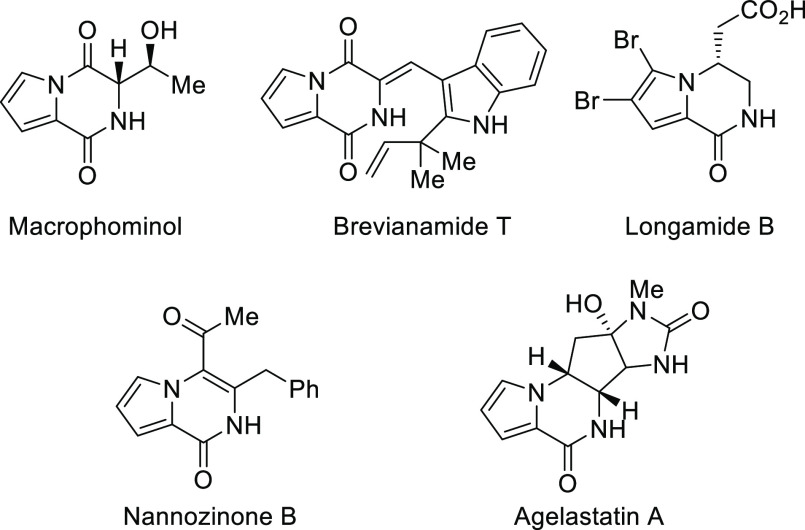
Selected pyrrolodiketopyperazines and pyrrolopyrazinone compounds.^2^

Natural products (NPs) still rank first as the source of inspiration for the design and discovery of new bioactive compounds. Collections based on NPs have been developed using different approaches that go from CtD^3^ (complexity to diversity), through scaffold manipulation and decoration, to BIOS^4^ (biology-oriented synthesis) strategies. DOS^5^ (diversity-oriented synthesis) offers a complementary approach to produce skeletal variety provided by the robust inter- and intramolecular couplings of building blocks to introduce stereochemical information. More recently, design principles for bioactive compound discovery consider that “pseudo-natural products” built by unprecedented combinations of NP fragments may provide access to novel scaffolds retaining chemical and biological properties of NPs.^6^ On the other hand, among drug-like descriptors, the Fsp3 factor (the number of sp3 hybridized carbons/total carbon account) along with the number of stereocenters of the molecule appear to increase the clinical success rate by increasing solubility and affinity for three-dimensional target proteins.^7^

In this context, the pyrrolodiketopiperazine skeleton possesses the potential to participate in CtD and DOS-oriented synthesis and indeed comprises the pseudo NP-design principles and connectivity patterns established to create collections for the modulation of many drug targets (Figure 2A).^8^ Nevertheless, most synthetic efforts have been directed toward the preparation of representative members, isolated from natural sources, rather than designing effective catalytic processes to access pyrrolodiketopiperazine diversity.^9^ For the particular case of the construction of three-dimensional scaffolds, only the aerobic oxidation of α-amino acid-based pyrrolodiketopiperazine skeletons has been reported (Figure 2B).^10,11^ α-Hydroperoxy- or α-hydroxy-pyrrolodiketopiperazines with an in-ring tetrasubstituted stereocenter were obtained in good yields by the action of triplet dioxygen under neutral conditions.

**Figure 2:**
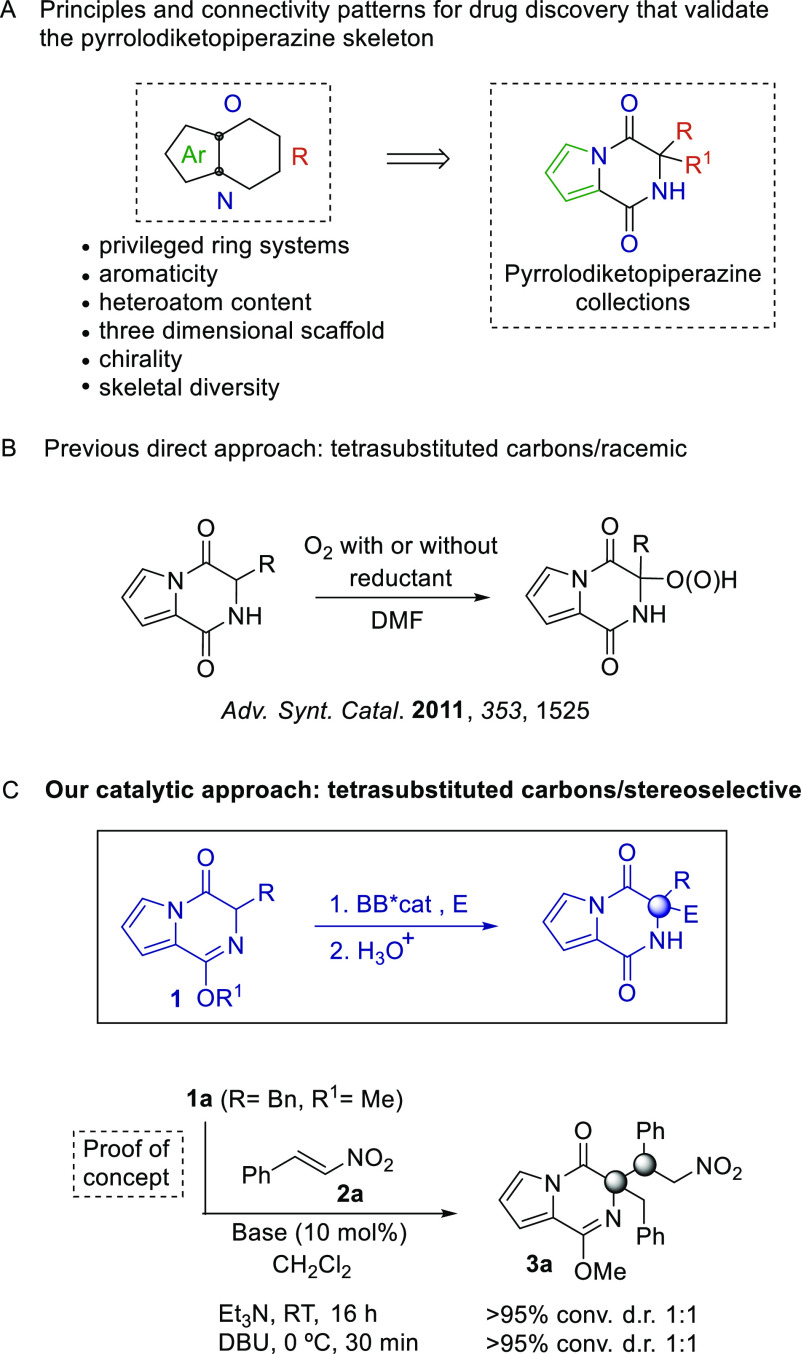
(A) Structural modularity of the pyrrolodiketopierazine skeleton for drug discovery. (B) Precedents for the synthesis of tetrasubstituted pyrrolodiketopierazines. (C) Present work: proof of concept.

Given the lack of catalytic methodologies^12^ and continuing with our interest in exploiting the propitious steric and electronic features of heterocyclic compounds in organocatalytic transformations,^13^ we focused our attention on the unexplored bicyclic acylpyrrol lactims **1**. These heterocycles, which could be considered as Schöllkopf bis-lactim surrogates,^14^ might behave as appropriate platforms to access pyrrolodiketopyperazines under Brønsted base catalysis (Figure 2C).

In the presence of weak bases, their suitability toward deprotonation, through the formation of pseudoaromatic enolates, would constitute a facile strategy for the creation of structural and stereochemical diversity from readily available α-amino acids (Figure 2C). The preparation of **1a** was effected from l-phenylalanine and pyrrole-2-carboxylic acid by peptide coupling, and subsequent cyclization and lactim formation.^15,16^ Initial assessment of the behavior of this compound in conjugate additions was gratifying, as the reaction of **1a** with β-nitrostyrene (**2a**), in the presence of substochiometric amounts of base, afforded the corresponding adduct **3a** that features a tetrasubstituted stereocenter and a tertiary adjacent stereocenter, in a clean and efficient manner. As three-dimensional structures seem to provide a number of superior properties in the search of biologically active molecules, compared with flat aromatic compounds,^17,18^ we envisioned that this approach could serve to mitigate the lack of protocols to generate chiral pyrrolodiketopierazines. In order to address the indispensable control over the stereoselectivity, we relied on the proven ability of chiral Brønsted bases linked to hydrogen bond donors to efficiently perform under proton transfer conditions.^19^ Among other possibilities, ureidoaminal-derived Brønsted bases, previously reported by our group,^20^ were tested in the Michael reaction of **1a** with **2a** (Scheme 1).^21^ These catalysts are readily available by condensation of α-amino acid-derived isocyanates with chiral amines, a simple protocol that provides an easy evaluation of the impact of the catalyst structure in the reaction efficiency. Initially, we confirmed that catalysts built up from carbamate protected *tert*-leucine and (1*S*,2*S*)-2-(piperidin-1-yl)cyclohexan-1-amine (**C1**–**C5**) provided adduct **3aa** with diastereomeric ratios greater than 92:8 and high enantioselectivity.^22^ The replacement of the Brønsted base moiety in catalyst **C6** provoked a noticeable reduction of the enantiomeric excess that was recovered when ureidopeptide **C7** was employed to promote the Michael addition. As **C7** constitutes an unexplored variant of ureidoaminal-derived Brønsted bases with increased flexibility, we chose to investigate the effect of reaction conditions in the asymmetric induction exerted by this new catalyst. Upon the customary screening of temperature and solvent, we were delighthed to find that **C7** furnished **3a** with 96:4 diastereomeric ratio and 88% enantiomeric excess in toluene at −20 °C.^23^

**Scheme 1:**
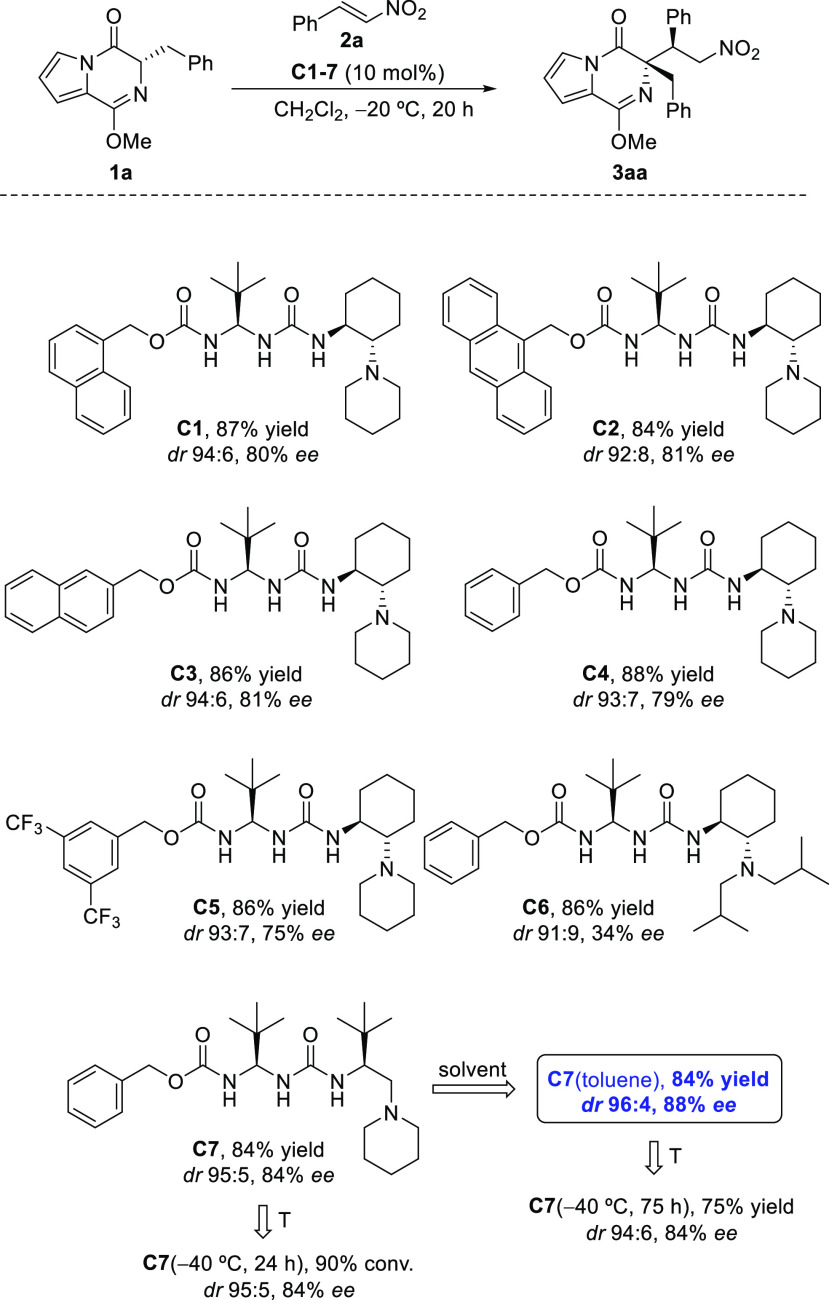
Evaluation of Catalysts and Conditions for the Michael Addition Reaction conditions: **1a** (0.1 mmol), **2a** (0.15 mmol), catalyst (10 mol %), solvent (0.3 mL). Isolated yields. Diastereomeric ratio and enantioselectivity determined by chiral HPLC.

Encouraged by these results, we proceeded to study the scope of the reaction (Scheme 2). First, we evaluated the compatibility of the catalyst system with the electrophilic counterpart. We were pleased to find that the reaction with **1a** exhibits remarkable scope for a representative selection of nitroolefins bearing β-aryl substituents, giving the corresponding adducts **3a**–**e** with excellent diastereomeric ratios, typically greater than 95:5 and *ee* values of up to 88%. The method also works with nitroolefins having heteroaromatic β-substituents to afford adducts **3af**, **3ag**, and **3ah**, and even with recalcitrant β-alkyl-substituted nitroolefins to produce **3ai**, essentially as single diastereomers and *ee* values up to 98%. The effectiveness of the method is highlighted by the fact that pyrrol lactims **1** derived from natural and synthetic α-amino acids are readily accommodated by this process. The reaction with pronucleophiles derived from l-leucine (**1b**), *O*-methyl-l-tyrosine (**1c**), and L-tryptophan (**1e**) produced the corresponding adducts in good yields and as single diastereomers for certain combinations, e.g., **3bf**, **3ca,f,h**, and **3ea**. Nevertheless, the presence of extra coordinating groups as in pyrrol lactim **1e** impairs enantioselectivity, presumably by the formation of energetically closer diastereomeric transition states. The incorporation of d,l-homophenylalanine, d,l-allylglycine, d,l-phenylglycine, and 2-aminocaprylic acid in pyrrol lactims **1d**, **1f**, **1g**, and **1h**, respectively, resulted in efficient transformations, as well.

**Scheme 2:**
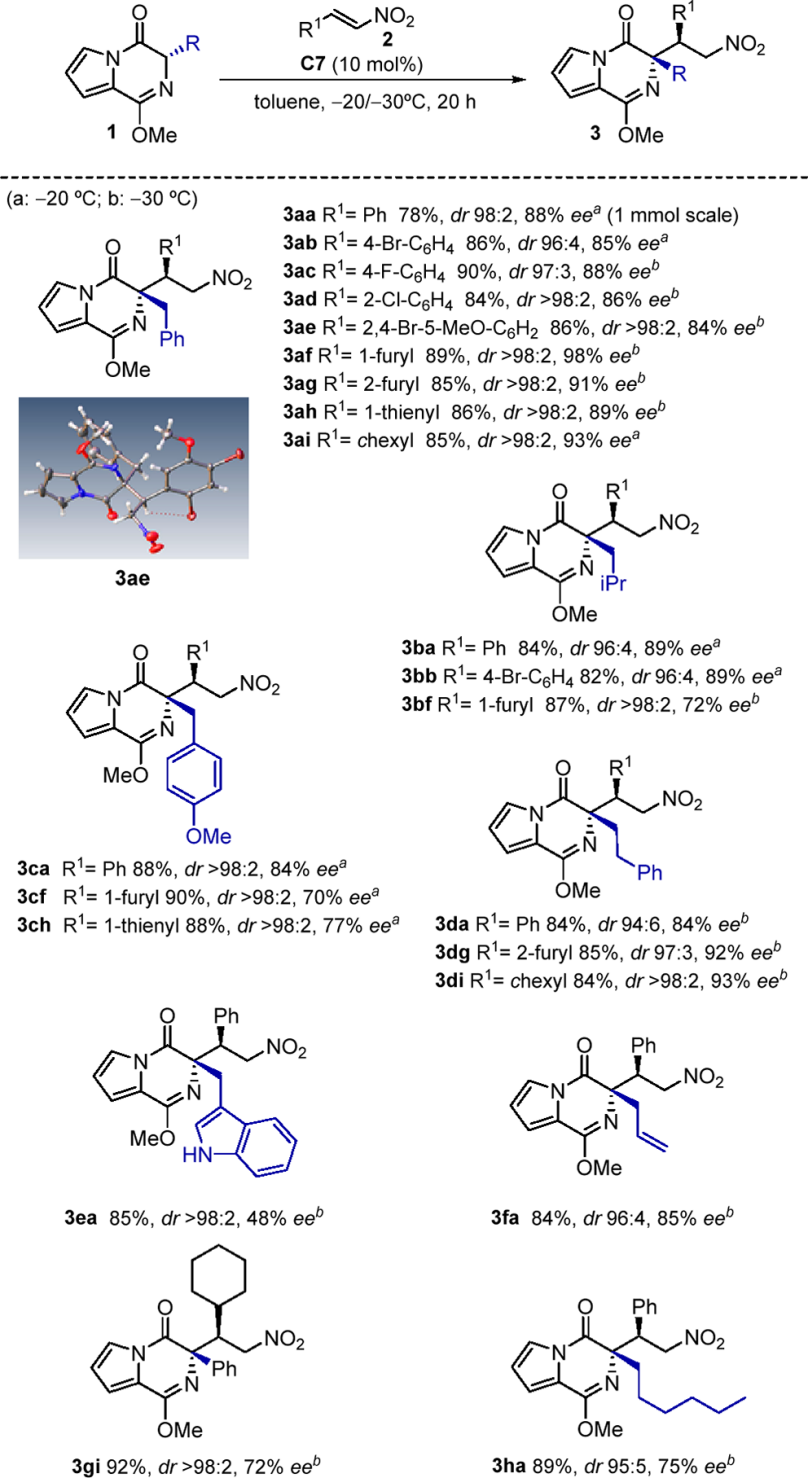
Scope of the Enantioselective Michael Addition

As is known for certain bifuncional Brønsted bases,^24^ self-aggregation may cause reactivity and stereoselectivity to be strongly dependent on the concentration and temperature at which the transformations are carried out. Nonetheless, in the reaction between **1a** and **2a**, neither the concentration (referred to **1a**) nor the catalyst loading affected the asymmetric induction exerted over adduct **3aa** (Figure 3). With these experimental results, it might be argued that, under the conditions in which the Michael addition is performed, the catalyst appears as a monomeric species in solution and only one molecule of catalyst would be involved in the stereodetermining step.^25^ Among a different hypothesis, the asymmetric induction exerted over the kinetically produced adducts^26^ could be related to the prevalence of a major conformer of catalyst **C7** rather than to the increased steric demand at the stereogenic centers. Indeed, the most stable conformation computed for **C7** in toluene shows how the *tert*-butyl groups, located at both sides of the urea moiety, tend to separate to minimize steric interactions.^27^

**Figure 3:**
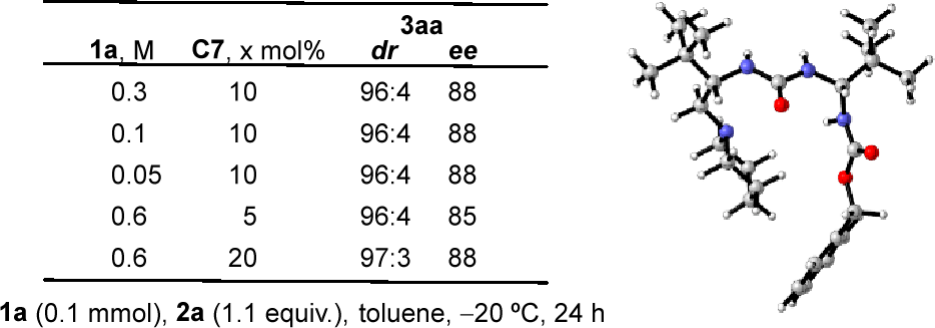
Impact of concentration and catalyst loading on stereoselectivity. Most stable computed conformation of **C7** in toluene.

To corroborate the synthetic utility of this organocatalytic methodology, we confirmed that adducts **3** are efficiently converted into the target pyrrolodiketopiperazines **4**, under acidic conditions. Additionally, pyrrolodiketopiperazines **4** may be adequate platforms to access more diversity by exploiting the orthogonal properties of the functional groups installed in the core. For example, the reduction of the nitro group in **3aa**, followed by protection, affords the corresponding protected primary amine **5** and the manipulation of **3fa**, under mild reaction conditions, produces the complex spiro compound **6** as a single diastereomer (Scheme 3).

**Scheme 3:**
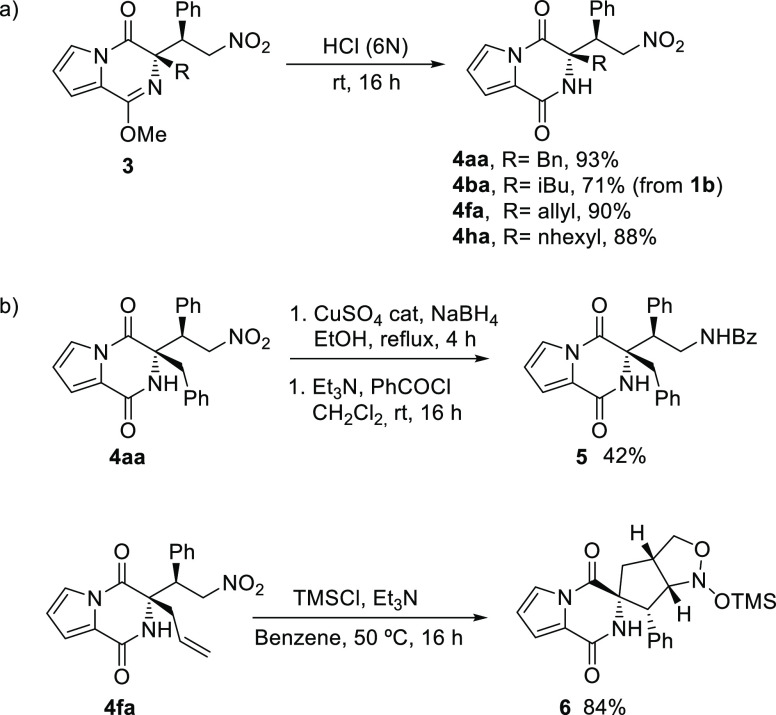
(a) Production of the Target Chiral Pyrrolopyrazinones **4**; (b) Modified Pyrrolopyrazinones **4** under Mild Conditions

In summary, we report here the first enantioselective construction of chiral pyrrolodiketopiperazines, via a direct carbon–carbon bond forming reaction, promoted by a ureidoaminal-derived Brønsted base that affords high skeleton diversity with chemical and sterochemical efficiency. We believe that this methodology produces versatile pyrrolodiketopiperazines that could enter drug discovery programs.

## Data Availability

The data underlying this study are available in the published article and its Supporting Information.

## Supporting Information Available

Experimental procedures, spectroscopic data of products, ^1^H NMR and ^13^C NMR spectra, HPLC charts, computational methods, Cartesian coordinates, and crystallographic data (PDF)

## Dedication

Dedicated to Prof. Dr. Joan Bosch on the occasion of his 75th anniversary.
