# Supplementary material for: Accessing Chiral Pyrrolodiketopiperazines under Organocatalytic Conditions
Source: Org Lett. 2022 Dec 29;25(1):125–9. doi: 10.1021/acs.orglett.2c03924 (PMC10018776; doi:10.1021/acs.orglett.2c03924)
Supplement: Supplementary file 1 — ol2c03924_si_001.pdf [file ol2c03924_si_001.pdf]

Supporting Information

Accessing Chiral Pyrrolodiketopiperazines under Organocatalytic Conditions

Eider Duñabeitia, Aitor Landa, Rosa López, Claudio Palomo

Table of Contents

1. Materials and general techniques……S2
2. Experimental procedures……S4
   - 2.1. General procedure for the synthesis of catalysts……S4
   - 2.2. Preparation of acyl pyrrol lactims **1**……S18
   - 2.3. Michael addition acyl pyrrol lactims **1** to nitroalkenes **2**……S27
     - 2.3.1. Catalyst screening……S27
     - 2.3.2. Solvent screening……S28
     - 2.3.3. Reaction scope……S29
   - 2.4. Elaboration of adducts……S39
     - 2.4.1. Preparation of diketopyrrolo piperazines **4**……S39
     - 2.4.2. Preparation of primary amine **5**……S41
     - 2.4.3. Preparation of spiro compound **6**……S42
3. Computational details……S43
4. X-Ray crystallographic data of **3ae**……S47
5. ^{1}H and ^{13}C NMR spectra for selected compounds……S48
6. HPLC chromatograms for selected compounds……S93

1. Materials and general techniques

General experimental: All non-aqueous reactions were performed under argon atmosphere in flame dried glassware with efficient magnetic stirring. Heat requiring reactions were performed using a hot plate with a sand or an oil bath and a condenser. Reactions requiring low temperatures were performed using cooling bath circulators, Huber T100E, and acetone or isopropanol baths. Yields refer to chromatographically purified and spectroscopically pure compounds, unless otherwise stated.

Solvents and reagents: All reagents bought from commercial sources were used as sold. Organic solvents were evaporated under reduced pressure using a Büchi rotary evaporator. When anhydrous solvents were required, they were dried following stablished protocols. Dichloromethane and acetonitrile were dried over CaH_2. Toluene was dried over sodium. *N,N*-dimethylformamide and dimethyl sulfoxide were dried over MS (3Å). Tetrahydrofuran was dried over sodium/benzophenone. Triethylamine, DBU and *N,N*-diisopropylamine were purified by distillation. After purification, catalysts were basified with aqueous saturated $NaHCO_3$ before usage.

Chromatography: Reactions were monitored by thin layer chromatography (TLC) using Merck silica gel 60 F254 plates and visualized by fluorescence quenching under UV light. In addition, TLC plates were stained with a dipping solution of potassium permanganate (1 g) in 100 mL of water (limited lifetime), followed by heating. Chromatographic purification was performed on ROCC 60 silica gel 40-63 µm.

Melting points: Melting points were obtained on a Stuart SHP3 melting point apparatus microscope and are uncorrected.

Mass spectra: MS spectra were recorded on an ESI-ion trap Mass spectrometer (Agilent 1100 series LC/MSD, SL model). Mass spectrometry analyses were performed in the General Research Service (SGIker) of the University of the Basque Country (UPV/EHU)

NMR spectra: NMR spectra were recorded using a Bruker Avance 300 MHz spectrometer, chemical shifts (δ) are quoted in parts per million referenced to the residual solvent peak. In case of diastereomeric mixture, data of the major diastereomer were provided. The multiplicity of each signal is designated using the following abbreviations: s, singlet; d, doublet; t, triplet; q, quartet; m, multiplet; brs, broad singlet. Coupling constants (*J*) are reported in Hertz (Hz).

Determination of enantiomeric excesses: Enantiomeric excesses were determined using analytical high performance liquid chromatography (HPLC) performed on a Waters 600 (Photodiode Array Detector Waters 2996) (column and solvent conditions are given with the compound).

Optical rotations: Optical rotations were recorded using a Jasco P-2000 polarimeter; specific rotation (SR) ([α]*D*) are reported in 10-1 deg·cm2·g-1; concentrations (*c*) are quoted in g/100 mL; *D* refers to the D-line of sodium (589 nm); temperatures (*T*) are given in degree Celsius (°C).

X-Ray diffraction analysis: The X-ray diffraction analysis experiments were performed on a SuperNova, Single source at offset/far, Atlas diffractometer. The crystal was kept at 149.93(16) K during data collection. Using Olex2,[1] the structure was solved with the ShelXT[2] structure solution program using Intrinsic Phasing and refined with the ShelXL[3] refinement package using Least Squares minimisation.

[1] Dolomanov, O.V., Bourhis, L.J., Gildea, R.J, Howard, J.A.K. & Puschmann, H. **2009**, J. Appl. Cryst. 42, 339-341.

[2] Sheldrick, G.M. **2015**. Acta Cryst. A71, 3-8.

[3] Sheldrick, G.M. **2015**. Acta Cryst. C71, 3-8.

2. Experimental procedures

2.1. General procedure for the synthesis of catalysts

All new bifunctional catalysts employed in this work include a chiral amine as Brønsted-base fragment. The synthesis of these amines is first described in the following section. The rest of catalyst employed in the screening were prepared as previously described, catalysts **C9**,[4] **C10**,[5] and **C11**,[6] or purchased from commercial suppliers, catalyst **C13**.

2.1.1. Preparation of chiral amines

2.1.1.1. 9-amino-(9-deoxy)epiquinine[7]

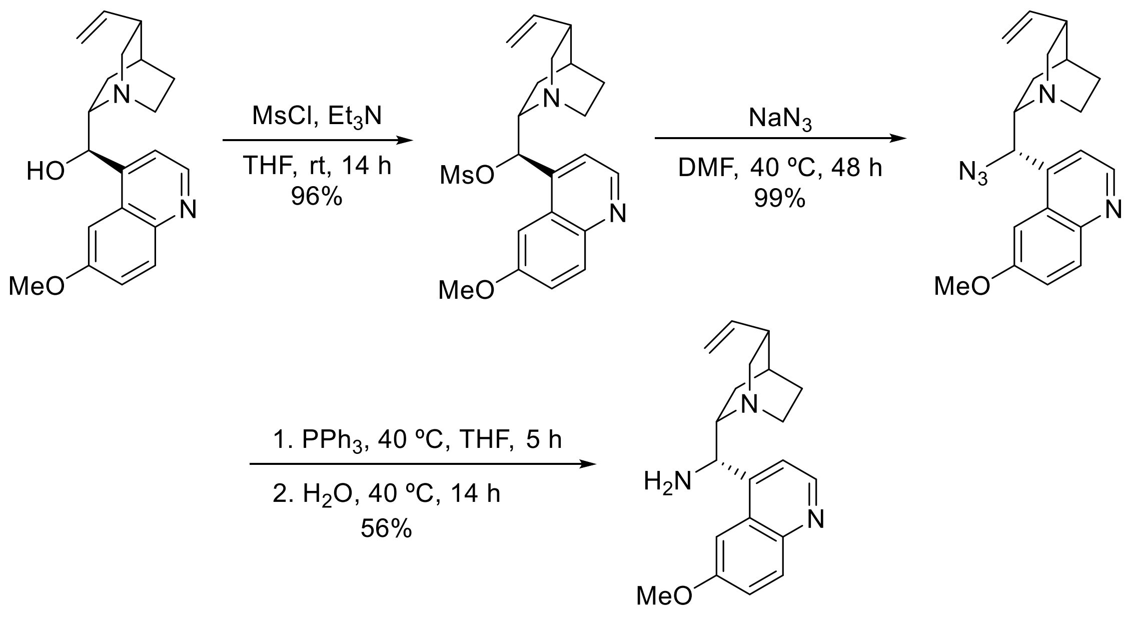

Step 1:[8] A mixture of quinine (16.2 g, 50 mmol, 1 equiv.) and Et_3N (25.1 mL, 180 mmol, 3.6 equiv.) in dry THF (250 mL) was cooled to 0 °C and then methanesulfonyl chloride (7.0 mL, 90 mmol, 1.8 equiv.) was added dropwise. The mixture was stirred 14 hours at room temperature. The reaction was quenched with water (40 mL) and then the solvent was removed under vacuum. The residue was dissolved in CH_2Cl_2 (40 mL) and washed successively with water (30 mL) and saturated $NaHCO_3$ (30 mL). The organic layer was dried over $MgSO_4$, filtered and concentred under vacuum to afford the crude

[4] Yang, W.; Du, D.-M. *Adv. Synth. Catal.* **2011**, *353*, 1241-1246.

[5] Yang, W.; Du, D.-M. *Org. Lett.* **2010**, *12*, 5450-5453.

[6] Vakulya, B.; Varga, S.; Csámpai, A.; Soós, T. *Org. Lett.* **2005**, *7*, 1967-1969.

[7] Adapted from: Brunner, H.; Büegler, J.; Nuber, B. *Tetrahedron: Asymmetry*, **1995**, *6*, 1699-1702.

[8] Adapted from: Zielinska-Blajet, M.; Kucharska, M.; Skarzewski, J. *Synthesis* **2006**, 4383-4387.

mesylate in 96% yield (19.3 g), which was used in the next step without further purification.

Step 2:[9] The crude product (19.3 g, 48 mmol, 1 equiv.) was dissolved in DMF (150 mL). The solution was cooled to 0 °C and NaN_3 (6.2 g, 96 mmol, 2 equiv.) was added portionwise. The reaction mixture was stirred at 40 ^oC for 48 hours and then was quenched with water (80 mL) and EtOAc (150 mL) was added. The organic layer was separated and washed with saturated NaCl (5 x 60 mL), dried over $MgSO_4$, filtered and evaporated under reduced pressure to obtain the crude product in quantitative yield (16.8 g), which was used in the next step without further purification.

Step 3:**[3]** The crude product was dissolved in THF (250 mL) and PPh_3 (12.6 g, 48 mmol, 1 equiv.) was added. The reaction mixture was heated at 40 °C and stirred until the gas evolution ceased (aprox. 5 hours). Then, water (8 mL) was added and the mixture was stirred overnight at 40 °C. The solvent was removed under vacuum and the residue was dissolved in CH_2Cl_2 (150 mL). HCl 6 M (250 mL) was added and the aqueous phase was separated and washed with CH_2Cl_2 (2 x 100 mL). Then the aqueous layer was cooled to 0 °C and basified until pH > 10 with NaOH 40%. The aqueous phase was then extracted with CH_2Cl_2 (3 x 150 mL), dried over $MgSO_4$ and concentrated under reduced pressure to afford 9-amino-(9-deoxy)*epi*quinine as a yellow viscous oil. Yield: 56% (8.7 g, 26.9 mmol). ^1H NMR was consistent with that previously reported.[10] ^1H NMR (300 MHz, $CDCl_3$), δ: 8.75 (d, *J* = 4.6 Hz, 1H), 7.36 – 8.05 (m, 4H), 5.79 – 5.75 (m, 1H), 4.97 (m, 2H), 4.57 (d, *J* = 10.4 Hz, 1H), 3.97 (s, 3H), 3.02 – 3.34 (m, 3H), 2.75 – 2.77 (m, 2H), 2.27 – 2.24 (m, 1H), 2.08 (s, 2H), 1.26 – 1.63 (m, 4H), 0.80 – 0.78 (m, 1H).

2.1.1.2. (1S,2S)- and (1R,2R)-2-(piperidin-1-yl)cyclohexan-1-amine[11]

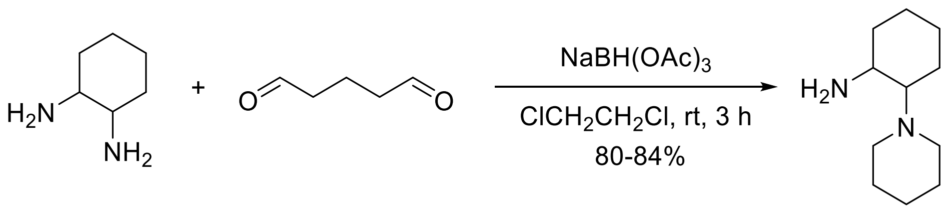

[9] Adapted from: Sudermeier, U.; Döbler, C.; Mehlretter, G. M.; Baumann, W. *Chirality*, **2003**, *15*, 127-134.
[10] He, W.; Liu, P.; Zhang, B. L.; Sun, X. L.; Zhang, S. Y. *Appl. Organometal. Chem.* **2006**, *20*, 328-334.
[11] Gonzalez-Sabin, J.; Gotor, V.; Rebolledo, F. *Chem. Eur. J.* **2004**, 10, 5788-5794.

(1*S*,2*S*)-2-(Piperidin-1-yl)cyclohexan-1-amine[6]

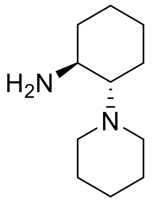

Glutaraldehyde (50 wt % H_2O, 0.93 mL, 5.1 mmol, 1.05 equiv.) was added dropwise to a mixture of (1*S*,2*S*)-(+)-1,2-diaminocyclohexane (0.56 g, 4.9 mmol, 1.0 equiv.) and $NaBH(OAc)_3$ (4.16 g, 19.6 mmol, 4.0 equiv.) in $ClCH_2CH_2Cl$ (30 mL) at room temperature. The mixture was stirred at room temperature for 3 hors, and quenched with NaOH 6.0 M (15 mL). The organic layer was separated and the aqueous layer was extracted with CH_2Cl_2 twice (2 x 15 mL). The organic layers were combined and washed with brine (1 x 15 mL), dried over $MgSO_4$, filtered and concentrated under reduced pressure to afford the diamine as a brown oil. Yield: 80% (0.715 g, 3.92 mmol). Spectral data were in agreement with the data described in the literature. ^{1}H NMR (300 MHz, $CDCl_3$) δ: 2.71 – 2.51 (m, 3H), 2.40 – 2.19 (m, 2H), 1.87 – 1.59 (m, 5H), 1.60 – 1.35 (m, 6H), 1.25 – 0.99 (m, 4H).

(1*R*,2*R*)-2-(Piperidin-1-yl)cyclohexan-1-amine[12]

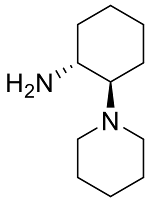

The same procedure described above was employed using (1*R*,2*R*)-(+)-1,2-diaminocyclohexane. Yellow liquid. Yield: 84% (0.750 g, 4.12 mmol). Spectral data were in agreement with the data described in the literature. ^{1}H NMR (300 MHz, $CDCl_3$) δ: 2.87 – 2.68 (m, 1H), 2.67 – 2.49 (m, 3H), 2.41 – 2.19 (m, 2H), 2.16 – 1.92 (m, 2H), 1.88 – 1.34 (m, 8H), 1.31 – 0.97 (m, 4H).

2.1.1.3. (1S,2S)-N1,N1-diisobutylcyclohexane-1,2-diamine

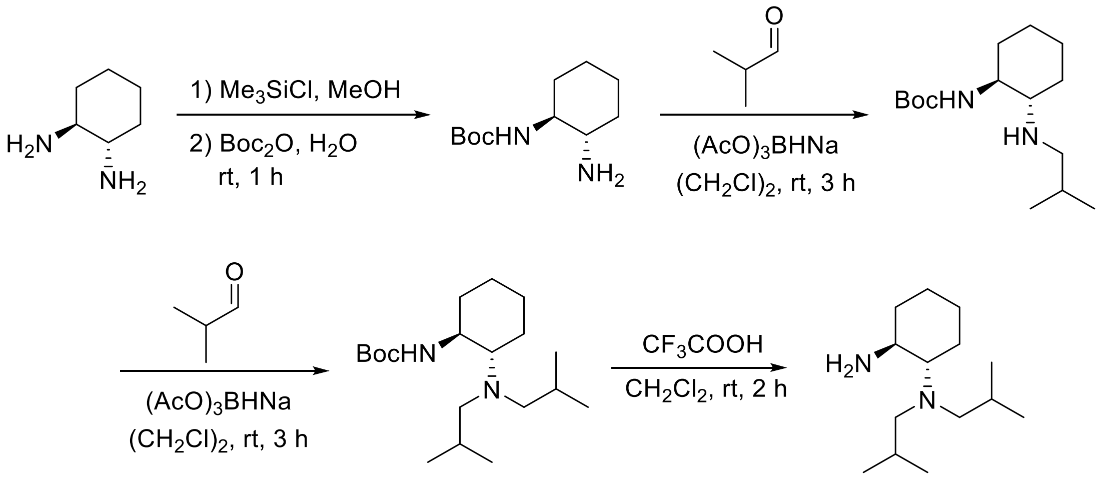

[12] Zhu, Y.; Malerich, J. P.; Rawal, V. H. *Angew. Chem. Int. Ed.* **2010**, *49*, 153-156.

Step 1:[13] To the (1*S*,2*S*)-(+)-1,2-diaminocyclohexane (1.14 g, 10 mmol, 1 equiv.) was added MeOH (12 mL) at 0 °C under stirring, followed by the dropwise addition of freshly distilled Me_3SiCl (1.27 mL, 10 mmol, 1 equiv.). A white precipitate appeared at the bottom of the flask. Then the mixture was allowed to warm up to room temperature and water (1 mL), followed by Boc_2O (1 equiv.) in MeOH (3 mL), was added. The mixture was stirred at room temperature for 1 hour, diluted with water and the aqueous layer washed with Et_2O (2 x 50 mL). The aqueous layer was adjusted to pH > 12 with NaOH 2N and extracted with CH_2Cl_2 (3 x 50 mL). The combined organic layers were dried over anhydrous $MgSO_4$, filtrated and the evaporation of the solvents under vacuum gave the corresponding monoprotected diamine which was used in the next step without further purification. White solid. ^{1}H NMR (300 MHz, $CDCl_3$) δ: 4.49 (brs, 1H), 3.13 (d, J = 6.2 Hz, 1 H), 2.33 (ddd, J = 10.4, 3.8, 3.8 Hz, 1H), 1.98 (m, 2H), 1.70 (m, 2H), 1.45 (s, 9H), 1.28 (m, 2H), 1.12 (m, 2H).

Step 2 and step 3: The protected diamine (1.71 g, 8 mmol, 1 equiv.) and sodium $NaBH(OAc)_3$ (3.39, 16 mmol, 2 equiv.) were dissolved in $ClCH_2CH_2Cl$ (23 mL) and isobutyraldehyde (0.77 mL, 8.4 mmol, 1.05 equiv.) was added dropwise at 0 °C. After 3 h the reaction was quenched with NaOH 6N and extracted with CH_2Cl_2 (4 x 25 mL). The organic layers were dried over $MgSO_4$, filtrated and evaporated under vacuum. The crude mixture was purified by a flash column chromatography (eluting with Hexane/EtOAc 80/20). White solid. ^{1}H NMR (300 MHz, $CDCl_3$) δ: 4.57 (s, 1H), 3.36 – 3.19 (m, 1H), 2.53 (dd, *J* = 11.2, 6.8 Hz, 1H), 2.30 (dd, *J* = 11.2, 6.6 Hz, 1H), 2.21 (td, *J* = 10.1, 3.9 Hz, 1H), 1.79 – 1.61 (m, 4H), 1.59 – 1.50 (m, 4H), 1.47 (s, 9H), 1.24 – 1.08 (m, 3H), 0.92 (d, *J* = 6.6 Hz, 6H).

Step 2 was repeated to provide the diprotected diamine, which was was used in the next step without further purification. ^{1}H NMR (300 MHz, $CDCl_3$) δ: 5.43 (s, 1H), 3.15 (tt, *J* = 10.6, 3.5 Hz, 1H), 2.80 – 2.47 (m, 3H), 2.31 – 2.15 (m, 3H), 1.93 – 1.83 (m, 1H), 1.83 – 1.75 (m, 2H), 1.66 (dt, *J* = 9.9, 3.3 Hz, 3H), 1.44 (s, 9H), 0.92 (d, *J* = 6.5 Hz, 6H), 0.86 (d, *J* = 6.6 Hz, 6H).

Step 4: The previously obtained diamine (1.14 g, 3.5 mmol, 1 equiv.) was dissolved in CH_2Cl_2 (8 mL) and CF_3COOH (2 mL) and stirred at room temperature for 2 h. The solvent was then removed under reduced pressure and the residue was redissolved in CH_2Cl_2 (10 mL). The solution was washed with NaOH (40%), dried over $MgSO_4$, filtrated

[13] Servín, F. A.; Romero, J. A.; Aguirre, G.; Grotjahn, D.; Somanathan, R.; Chávez, D. *J. Mex. Chem. Soc.* **2017**, *61*, 23-27.

and evaporated under vacuum to afford the desired product as a yellow oil. Yield: 0.697 g, 3.1 mmol, 88%. ^{1}H NMR (300 MHz, $CDCl_3$) δ: 2.62 – 2.51 (m, 1H), 2.28 – 2.15 (m, 2H), 2.02 (dd, J = 12.7, 10.0 Hz, 3H), 1.79 – 1.61 (m, 9H), 1.26 – 1.02 (m, 5H), 0.92 (d, J = 6.5 Hz, 6H), 0.84 (d, J = 6.7 Hz, 4H).

2.1.1.4. Preparation of (S)-3,3-dimethyl-1-(piperidin-1-yl)butan-2-amine[14]

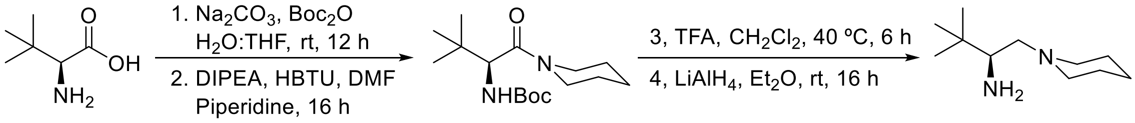

Steps 1 and 2: Na_2CO_3 (2.12 g, 20 mmol, 2 equiv.) and Boc_2O (3.3 g, 15 mmol, 1.5 equiv.) were added to a solution of *t*-leucine (1.31 g, 10 mmol, 1 equiv.) in water (20 mL) and THF (5 mL) at 0 °C. After stirring for 12 hours at room temperature, HCl (10 %) was added until pH 2 and the mixture was extracted with EtOAc (3 x 30 mL). The organic phases were combined, washed with brine (50 mL), dried over $MgSO_4$, after which the solvent was removed under reduced pressure. The residue was redissolved in dry DMF (20 mL) and DIPEA (2.58 g, 20 mmol, 2 equiv.) and HBTU (5.7 g, 15 mmol, 1,5 equiv.) were added. After stirring for 1 hour, piperidine (0.94 g, 11 mmol, 1.1 equiv.) was added and the mixture was stirred for further 16 h. The reaction was quenched adding HCl 1 M (20 mL) and the mixture was extracted with EtOAc (2 x 20 mL). The organic phases were combined, washed with a HCl 1 M and brine (20 mL), dried over $MgSO_4$ and the solvent was removed under reduced pressure. The residue was purified by flash column chromatography on silica gel (eluting with Hexane/EtOAc 85/15) to afford the corresponding *t*-leucine derivative as a white solid. Yield: 2.5 g, 8.3 mmol, 83%. All spectroscopic data were identical to those reported in the literature. ^{1}H NMR (300 MHz, CDCl3) δ: 0.98 (s, 9H), 1.43 (s, 9H), 1.52 – 1.62 (m, 6H), 3.46 – 3.69 (m, 4 H), 4.54 (d, J = 9.7 Hz, 1H), 5.38 (d, J = 9.6 Hz, 1H).

Steps 3 and 4: The previous compound (2.5 g, 8 mmol, 1 equiv.) was dissolved in a mixture of CH_2Cl_2 (8 mL) and CF_3COOH (2 mL) and stirred at 40 °C until no more starting material was observed by TLC (eluting with hexane/ EtOAc 70/30). The solvent was then removed under reduced pressure and the residue was redissolved in CH_2Cl_2 (10 mL). The solution was washed with NaOH (40%), dried over $MgSO_4$ and the solvent was removed under reduced pressure to produce the aminoamide as a yellow oil. The aminoamide was then dissolved in dry Et_2O (10 mL) and added dropwise over a suspension of $LiAlH_4$

[14] Adapted from: Gao, Y.; Ren, Q.; Wang, J. *Chem. Eur. J.* **2010**, *16*, 13068-13071.

(0.879 g, 24 mmol, 3 equiv.) in Et_2O (40 mL) at 0 °C under nitrogen atmosphere. The mixture was stirred at the same temperature for some minutes and afterwards it was stirred at room temperature for 16 hours. The reaction was quenched adding water (1.2 mL), NaOH 15% (1,2 mL) and water (3.6 mL) at 0 °C. The resulting suspension was filtered and the liquid was extracted with Et_2O (2 x 10 mL). The combined organic layers were dried over $MgSO_4$ and the solvent was eliminated under reduced pressure. The residue was purified by flash column chromatography on silica gel (eluting with Hexane/ EtOAc 1/1) to afford (*S*)-3,3-dimethyl-1-(piperidin-1-yl)butan-2- amine as yellow oil. Yield: 1.16 g, 6.8 mmol, 92%. All spectroscopic data were identical to those reported in the literature. 1H NMR (500 MHz, $CDCl_3$) δ: 2.66 (dd, J = 11.0, 2.5 Hz, 1H), 2.52 (d, J = 12.3 Hz, 4H), 2.28 (dd, J = 12.3, 2.8 Hz, 3H), 2.13 (dd, J = 12.1, 11.2 Hz, 1H), 1.61-1.53 (m, 4H), 1.44 – 1.42 (m, 2H), 0.90 (s, 9H).

2.1.2. Preparation of catalysts

2.1.2.1. Preparation of ureidoaminal-derived Brønsted base catalysts

2.1.2.1.1. Preparation of N-protected L-tert-leucine

The catalysts were prepared by coupling the previously described chiral amines with *L*-*tert*-leucine derived isocyanates. The preparation of no commercially avavilable *N*-protected L-tert-leucines is described first.

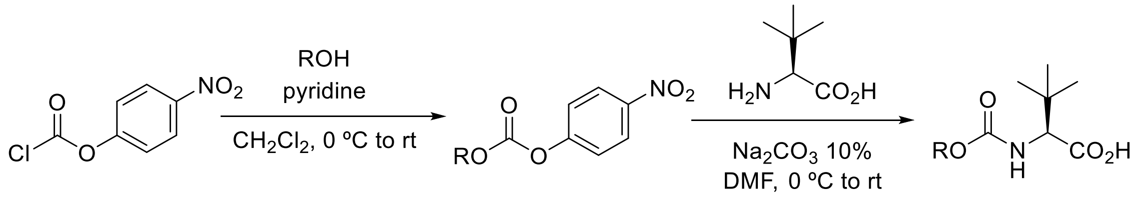

Step 1: Pyridine (0.9 mL, 11 mmol, 1.1 equiv.) was added to a stirred solution of *p*-nitrophenyl chloroformate (2.2 g, 11 mmol, 1.1 equiv.) in CH_2Cl_2 (13.6 mL). The white slurry was cooled to 0 °C and the corresponding alcohol (10 mmol, 1 equiv.) was slowly added at the same temperature. Then, the reaction mixture was allowed to warm to room temperature and stirred for 16 hours. The reaction mixture was diluted with CH_2Cl_2 (40 mL) and washed with HCl 1M (20 mL), water (20 mL) and brine (20 mL). The organic layer was dried over $MgSO_4$ and concentred under reduced pressure. The residue was used in the next step without further purification.

Step 2: To a stirred solution of *L*-tert-leucine (1.31 g, 10 mmol, 1 equiv.) in 10% Na_2CO_3 (26 mL), and DMF (10 mL), a solution of the corresponding carbonate (10 mmol, 1 equiv.) in DMF (30 mL) was slowly added at 0 °C. The mixture was stirred at the same

temperature for 1 hour and at room temperature for 16 hours.The reaction mixture was poured into water (100 mL) and washed with Et_2O (3 x 50 mL). The aqueous layer was cooled in an ice bath and acidified with concentrated HCl, followed by extraction with EtOAc (3 x 50 mL). The combined organic phases were washed with brine (5 x 50 mL), dried over $MgSO_4$ and concentrated under reduced pressure to produce the corresponding *N*-protected L-tert-leucine.

(*S*)-2-((((3,5-Bis(trifluoromethyl)benzyl)oxy)carbonyl)amino)-3,3-dimethylbutanoic acid[15]

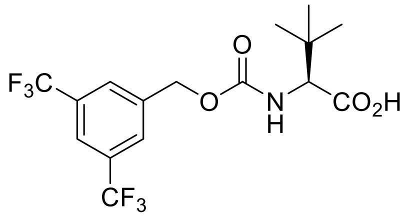

The title compound was prepared from 3,5-bis(trifluoromethyl)benzyl alcohol (2.44 g, 10 mmol) according to the general procedure. Removal of the remaining fenol was not possible by column chromatography, so after the work up described in the general procedure, the crude was dissolved in Et_2O (20 mL) and basified with NaOH 20%. The aqueous phase was washed with Et_2O (3 x 20 mL), acidified with concentrated HCl and extracted with EtOAc (3 x 25 mL). The organic phase was dried over $MgSO_4$ and evaporated under reduced pressure to afford the acid as a white solid. Yield: 91% (3.65 g, 9.1 mmol). All spectroscopic data were consistent with those previously reported. ^1H NMR (300 MHz, $CDCl_3$) δ 7.82 (s, 1H), 7.80 (s, 2H), 5.56 (d, *J* = 9.6 Hz, 1H), 5.36 –5.09 (m, 2H), 4.20 (d, *J* = 9.6 Hz, 1H), 1.03 (s, 9H).

(*S*)-3,3-Dimethyl-2-(((naphthalen-2-ylmethoxy)carbonyl)amino)butanoic acid[16]

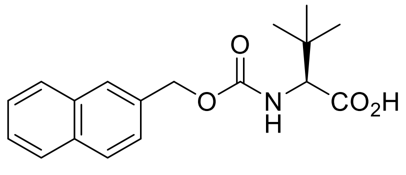

The title compound was prepared from 2-naphthalenemethanol (1.58 g, 10 mmol) according to the general procedure. Removal of the remaining fenol was not possible by column chromatography. After the work up described in the general procedure, the crude was dissolved in Et_2O (30 mL) and basified with saturate $NaHCO_3$ (1 x 20 mL). The aqueous phase was washed with Et_2O (3 x 20 mL), acidified with concentrated HCl and extracted with EtOAc (3 x 25 mL). The organic phase was dried over $MgSO_4$ and evaporated under reduced pressure to afford the acid as a white solid. Yield 48% (1.5 g, 4.8 mmol). All the spectroscopic data were coincident withthose previously reported. ^1H NMR (300 MHz, $CDCl_3$) δ 7.92 – 7.74 (m, 4H), 7.58 – 7.36(m, 3H), 5.47 (d, *J* = 9.4 Hz, 1H), 5.30 (s, 2H), 4.26 (d, *J* = 9.6 Hz, 1H),

[15] Bastida, I.; San Segundo, M.; López, R.; Palomo, C. *Chem. Eur. J.* **2017**, *23*, 13332-13336.

[16] Diosdado, S.; Etxabe, J.; Izquierdo, J.; Landa, A.; Mielgo, A.; Olaizola, I.; López, R.; Palomo, C. *Angew. Chem. Int. Ed.* **2013**, *52*, 11846-11851.

1.04 (s, 9H).

(S)-3,3-Dimethyl-2-(((naphthalen-1-ylmethoxy)carbonyl)amino)butanoic acid[17]

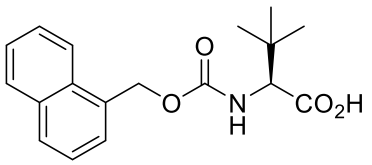

The title compound was prepared from 1-naphthalenemethanol (1.58 g, 10 mmol) according to the general procedure. Purification by column chromatography (Hexane/EtOAc, 80/20) afforded the product as a white solid. Yield: 88% (2.8 g, 8.8 mmol). All the spectroscopic data were coincident withthose previously reported. ^{1}H NMR (300 MHz, $CDCl_3$) δ: 10.10 (s,1H), 8.04 (d, *J* = 8.0 Hz, 1H), 7.87 (t, *J* = 8.8 Hz, 2H), 7.49 (dt, *J* = 27.2, 7.3 Hz, 4H), 5.60 (q, *J* = 12.3 Hz, 2H), 5.40 (d, *J* = 9.5 Hz, 1H), 4.26 (d, *J* = 9.6 Hz, 1H), 1.02 (s, 9H).

Anthracen-9-ylmethyl (S)-(1-amino-2,2-dimethylpropyl)carbamate[18]

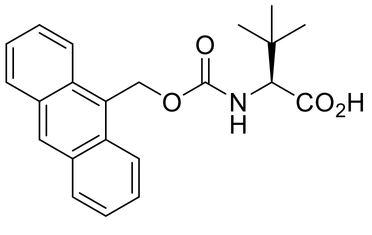

The title compound was prepared from 9-anthracenemethanol (1.58 g, 10 mmol) according to the general procedure. Purification by column chromatography (Hexane/EtOAc, 80/20) afforded the product as a white solid. Yield: 88% (2.8 g, 8.8 mmol). All the spectroscopic data were coincident with those previously reported. ^{1}H NMR (300 MHz, $CDCl_3$) δ: 8.52 (s, 1H), 8.38 (d, J = 8.9, 2H), 8.03 (d, J = 8.4, 2H), 7.54 (dt, J = 15.0, 7.0, 4H), 6.18 (q, J = 12.1, 2H), 5.24 (d, J = 9.5, 1H), 4.28 (d, J = 9.4, 1H), 1.01 (s, 9H).

2.1.2.1.2. L-tert-leucine derived isocyanate generation and coupling with chiral amines[10]

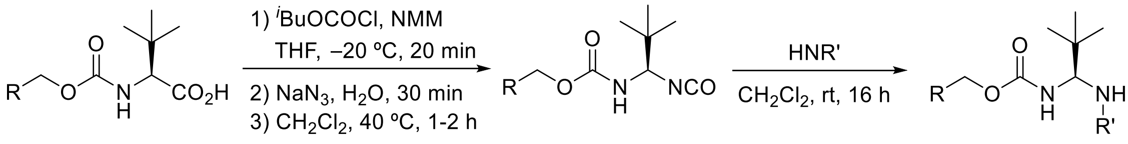

To a cooled solution of the corresponding *N*-protected α-amino acid (5 mmol, 1 equiv.) in dry THF (20 mL), isobutyl chloroformate (0.65 mL, 5 mmol, 1 equiv.) and *N*-methylmorpholine (0.6 mL, 5 mmol, 1 equiv.) were added at −20 °C. The mixture was stirred at the same temperature for 20 min. Then, a suspension of NaN_3 (0.48 g, 7.5mmol, 1.5 equiv.) in 5 mL of H_2O was added and the reaction mixture stirred at the same temperature for 30 min. The organic layer was separated, evaporated and the

[17] Vera, S.; Vázquez, A.; Rodriguez, R.; del Pozo, S.; Urruzuno, I.; de Cózar, A.; Mielgo, A.; Palomo C. *J. Org. Chem.* **2021**, *86, 7757-7772.*
[18] Echave, H.; López, R.; Palomo, C. *Angew. Chem. Int. Ed.* **2016**, *55*, 3364-3368.

residue was dissolved in CH_2Cl_2 (30 mL), and washed with water (15 mL). The organic phase was dried over $MgSO_4$, filtered and concentrated in *vacuo* to give a yellow oil which was dissolved in dry CH_2Cl_2 (10 mL). The resulting solution was stirred at 40 °C under nitrogen for 1-2 hours. The reaction was monitored by IR analysis until disappearance of the azide band (from azide λ = 2136 cm^{-1} to isocyanate λ = 2239 cm^{-1}).

After complete isocyanate generation, the corresponding amine was added (3.5 mmol, 0.7 equiv.) and the reaction mixture was stirred for 16 hours at room temperature. The solvent was evaporated under reduced pressure and the residue was purified by flash column chromatography on non-acidic silica gel to afford the desired catalysts.

Naphthalen-1-ylmethyl ((1*S*)-2,2-dimethyl-1-(3-((2*S*)-2-(piperidin-1-yl)cyclohexyl)ureido)propyl)carbamate (C1)[11]

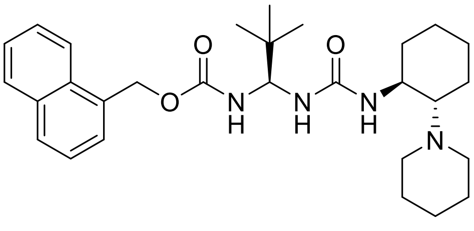

Prepared according to the general procedure starting from (*S*)-3,3-dimethyl-2-(((naphthalen-1-ylmethoxy)carbonyl)amino)butanoic acid (5 mmol). Purified by column chromatography by non-acid silica gel (eluting with Hexane/EtOAc 90/10). White solid. Yield: 60% (1.42 g, 3 mmol). $[\alpha]_D^{25}$ = -5.9 (c=1, CH_2Cl_2). All the spectroscopic data were coincident with those previously reported. ^{1}H NMR (500 MHz, DMSO-d_6, 70 °C) δ: 8.32 – 8.27 (m, 1H), 8.21 – 8.18 (m, 1H), 8.14 (d, *J* = 8.2 Hz, 1H), 7.85 – 7.77 (m, 3H), 7.76 – 7.69 (m, 1H), 7.19 (br s, 1H), 6.16 (d, *J* = 9.1 Hz, 1H), 6.00 – 5.95 (m, 1H), 5.75 (q, 2H), 5.39 (t, *J* = 9.2 Hz, 1H), 3.63 (br s, *J* = 6.7, 5.6 Hz, 1H), 2.85 (br s, 2H), 2.60 (br s, 2H), 2.43 (s, 1H), 2.28 (d, *J* = 12.5 Hz, 1H), 2.04 (d, *J* = 11.0 Hz, 1H), 1.94 (d, *J* = 11.4 Hz, 1H), 1.82 (d, *J* = 10.1 Hz, 1H), 1.74 – 1.66 (m, 5H), 1.64 – 1.53 (m, 3H), 1.47 – 1.36 (m, 3H), 1.12 (s, 9H).

Anthracen-9-ylmethyl ((*S*)-2,2-dimethyl-1-(3-((1*S*,2*S*)-2-(piperidin-1-yl)cyclohexyl)ureido)propyl)carbamate (C2)[11]

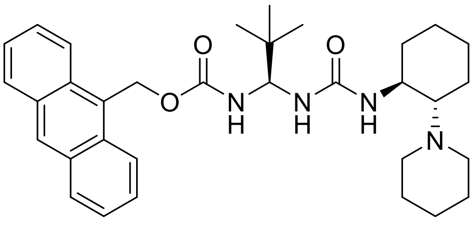

Prepared according to the general procedure starting from anthracen-9-ylmethyl (*S*)-(1-amino-2,2-dimethylpropyl)carbamate (5 mmol). Purified by column chromatography by non-acid silica gel (eluting with Hexane/EtOAc 90/10). White solid. Yield: 58% (1.58 g, 2.9 mmol). $[\alpha]_D^{25}$ = -6.7 (c=1, CH_2Cl_2). All the spectroscopic data were coincident withthose previously reported. ^{1}H NMR (500 MHz,

DMSO-d_6, 70 °C) δ: 8.67 (s, 1H), 8.37 (d, *J* = 8.8 Hz, 2H), 8.12 (dd, *J* = 8.4, 1.3 Hz, 2H), 7.59 (ddd, *J* = 8.6, 6.5, 1.5 Hz, 2H), 7.58 – 7.51 (m, 2H), 7.21 (d, *J* = 8.5 Hz, 1H), 6.04 (q, *J* = 11.5, 10.9 Hz, 3H), 5.79 (s, 1H), 5.16 (s, 1H), 2.54 (s, 1H), 2.28 (s, 1H), 2.01 – 1.95 (m, 1H), 1.79 – 1.72 (m, 1H), 1.69 – 1.63 (m, 2H), 1.56 – 1.50 (m, 1H), 1.39 (s, 5H), 1.28 – 1.22 (m, 3H), 1.19 – 1.07 (m, 4H), 0.82 (s, 9H).

Naphthalen-2-ylmethyl ((1*S*)-2,2-dimethyl-1-(3-((2*S*)-2-(piperidin-1-yl)cyclohexyl)ureido)propyl)carbamate (C3)[11]

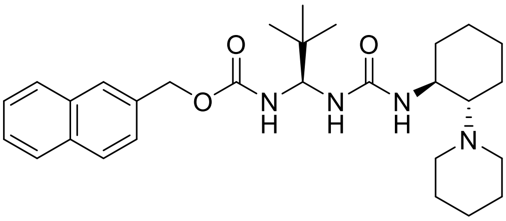

Prepared according to the general procedure starting from (*S*)-3,3-dimethyl-2-(((naphthalen-2-ylmethoxy)carbonyl)amino) butanoic acid (5 mmol). Purified by column chromatography by non-acid silica gel (eluting with Hexane/EtOAc 70/30). White solid. Yield: 61% (1.06 g, 2.14 mmol). $[\alpha]_D^{25}$ = -24.2 (c=1, CH_2Cl_2). All the spectroscopic data were coincident with those previously reported. ^{1}H NMR (500 MHz, DMSO-d_6, 70 °C) δ: 7.99 – 7.80 (m, 4H), 7.65 – 7.41 (m, 3H), 6.88 (s, 1H), 5.91 (d, *J* = 9.2 Hz, 1H), 5.69 (d, *J* = 6.3 Hz, 1H), 5.21 (s, 2H), 5.15 (t, *J* = 9.2 Hz, 1H), 3.48 – 3.31 (m, 1H), 2.60 – 2.55 (m, 2H), 2.39 – 2.27 (m, 2H), 2.24 – 2.11 (m, 1H), 2.09 – 2.03 (m, 1H), 1.82 – 1.74 (m, 1H), 1.72 – 1.64 (m, 1H), 1.64 – 1.52 (m, 1H), 1.52 – 1.39 (m, 4H), 1.39 – 1.25 (m, 3H), 1.24 – 1.12 (m, 3H), 0.91 (s, 9H).

Benzyl ((*S*)-2,2-dimethyl-1-(3-((1*S*,2*S*)-2-(piperidin-1-yl)cyclohexyl)ureido)propyl)carbamate (C4)

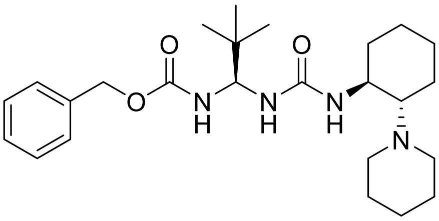

Prepared according to the general procedure starting from (*S*)-2-(((benzyloxy)carbonyl)amino)-3,3-dimethylbutanoic acid (5 mmol). Purified by column chromatography by non-acid silica gel (eluting with Hexane/EtOAc 80/20). White solid. Yield: 62% (1.38 g, 3.1 mmol). $[\alpha]_D^{25}$ = –21.3 (c=1, CH_2Cl_2). m.p.= 146-150 °C. ^{1}H NMR (300 MHz, $CDCl_3$) δ: 7.35 – 7.16 (m, 5H), 6.41 – 5.92 (m, 2H), 5.79 – 5.31 (m, 1H), 5.05 (q, *J* = 12.3 Hz, 4H), 3.53 (s, 1H), 2.78 – 2.54 (m, 2H), 2.47 – 2.29 (m, 1H), 2.25 – 2.12 (m, 1H), 1.87 – 1.69 (m, 3H), 1.66 – 1.45 (m, 5H), 1.40 – 1.30 (m, 2H), 1.29 – 1.09 (m, 4H), 0.95 (s, 9H). ^{13}C NMR (75 MHz, $CDCl_3$) δ: 158.1, 156.4, 136.6, 128.4, 128.0, 68.1, 66.9, 66.6, 53.5, 49.7, 35.6, 33.8, 25.7, 25.6, 25.4, 24.8, 23.7. UPLC-DAD-QTOF: $C_{25}H_{41}N_4O_3$ [M+H]+ calcd.: 445.3179, found: 445.3181.

3,5-Bis(trifluoromethyl)benzyl ((1*S*)-2,2-dimethyl-1-(3-((2*S*)-2-(piperidin-1-yl)cyclohexyl)ureido)propyl)carbamate (C5)

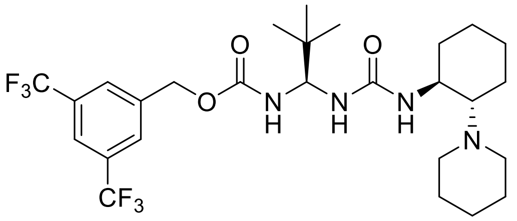

Prepared according to the general procedure starting from (*S*)-2-((((3,5-Bis(trifluoromethyl) benzyl)oxy)carbonyl)amino)-3,3-dimethylbutanoic acid (5 mmol). Purified by column chromatography by non-acid silica gel (eluting with Hexane/EtOAc 80/20). White solid. Yield: 58% (1.68 g, 2.8 mmol). m.p.= 291-294 °C. $[\alpha]_D^{25}$ = –3.50 (c=1, CH_2Cl_2). ^{1}H NMR (500 MHz, DMSO-d_6, 70 °C) δ: 8.05 (s, 2H), 7.96 (s, 1H), 7.05 (s, 1H), 5.94 (d, *J* = 9.1 Hz, 1H), 5.70 (d, *J* = 6.2 Hz, 1H), 5.29 – 5.19 (m, 2H), 5.13 (t, *J* = 9.1 Hz, 1H), 3.39 (s, 1H), 2.59 (br s, 2H), 2.34 (br s, 2H), 2.19 (d, *J* = 10.3 Hz, 1H), 2.05 (d, *J* = 12.6 Hz, 1H), 1.78 (s, 1H), 1.71 (d, *J* = 8.5 Hz, 1H), 1.58 (d, *J* = 10.1 Hz, 1H), 1.46 (s, 4H), 1.32 (d, *J* = 11.7 Hz, 3H), 1.22 – 1.13 (m, 3H), 0.90 (s, 9H). ^{13}C NMR (75 MHz, $CDCl_3$) δ: 158.7, 156.3, 140.2, 133.1, 132.6, 132.2, 131.8, 128.3, 125.6, 122.4, 122.0, 77.9, 68.8, 67.7, 65.4, 63.7, 36.3, 35.4, 34.5, 30.3, 27.1, 26.3, 26.2, 26.1, 25.9, 25.3, 24.2. UPLC-DAD-QTOF: $C_{25}H_{41}N_4O_3$ [M+H]+ calcd.: 581.2921, found: 581.2923.

Benzyl ((*S*)-1-(3-((1*S*,2*S*)-2-(diisobutylamino)cyclohexyl)ureido)-2,2-dimethylpropyl)carbamate (C6)

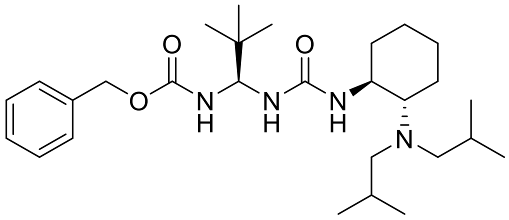

Prepared according to the general procedure starting from (*S*)-2-(((benzyloxy)carbonyl) amino)-3,3-dimethylbutanoic acid (5 mmol). Purified by column chromatography by non-acid silica gel (eluting with Hexane/EtOAc 90/10). White solid. Yield: 68% (1.66 g, 3.4 mmol). m.p.= 175–177 °C. $[\alpha]_D^{23}$ = –31.6 (*c*=1, CH_2Cl_2). ^{1}H NMR (300 MHz, DMSO-d_6) δ: 7.38 – 7.20 (m, 5H), 5.98 (d, *J* = 9.4 Hz, 1H), 5.59 (d, *J* = 7.1 Hz, 1H), 5.11 – 4.93 (m, 3H), 2.30 – 2.20 (m, 1H), 2.12 (d, *J* = 7.1 Hz, 4H), 1.88 (d, *J* = 11.8 Hz, 1H), 1.82 – 1.72 (m, 1H), 1.70 – 1.63 (m, 1H), 1.56 (td, *J* = 12.9, 12.4, 6.2 Hz, 3H), 1.17 – 1.00 (m, 3H), 0.84 (s, 9H), 0.80 (dd, *J* = 8.6, 6.6 Hz, 12H). ^{13}C NMR (75 MHz, DMSO) δ: 156.6, 155.7, 137.3, 128.3, 127.7, 127.7, 65.1, 63.0, 59.2, 50.4, 35.8, 34.5, 26.7, 25.7, 25.5, 25.4, 24.8, 21.0, 20.7. UPLC-DAD-QTOF: $C_{28}H_{49}N_4O_3$ [M+H]+ calcd.: 489.3805, found: 489.3812.

Benzyl ((*S*)-1-(3-((*S*)-3,3-dimethyl-1-(piperidin-1-yl)butan-2-yl)ureido)-2,2-dimethylpropyl)carbamate (C7)

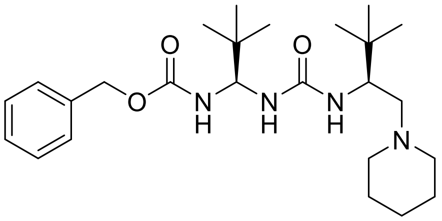

Prepared according to the general procedure starting from (*S*)-2-(((benzyloxy)carbonyl) amino)-3,3-dimethylbutanoic acid (5 mmol). Purified by column chromatography by non-acid silica gel (eluting with Hexane/EtOAc 90/10). White solid. Yield: 70% (1.56 g, 3.5 mmol). m.p.= 162–164 °C. $[\alpha]_D^{23}$ = –2.9 (*c*=1, CH_2Cl_2). ^{1}H NMR (300 MHz, $CDCl_3$) δ: 7.39 – 7.25 (m, 5H), 5.83 (s, 1H), 5.67 – 5.42 (m, 1H), 5.09 (s, 3H), 3.84 – 3.46 (m, 1H), 2.56 – 2.39 (m, 3H), 2.36 – 2.18 (m, 3H), 1.61 – 1.44 (m, 4H), 1.38 (q, *J* = 5.7 Hz, 2H), 0.97 (s, 9H), 0.89 (s, 9H). ^{13}C NMR (75 MHz, $CDCl_3$) δ: 158.5, 156.7, 136.4, 128.5, 128.5, 128.1, 128.0, 128.0, 127.9, 66.9, 66.9, 60.2, 54.7, 54.6, 35.9, 35.8, 34.8, 34.5, 27.1, 26.7, 26.1, 25.7, 25.6, 24.4. UPLC-DAD-QTOF: $C_{25}H_{43}N_4O_3$ [M+H]+ calcd.: 447.3335, found: 447.3339.

Naphthalen-1-ylmethyl ((*S*)-2,2-dimethyl-1-(3-((1*R*,2*R*)-2-(piperidin-1-yl)cyclohexyl)ureido)propyl)carbamate (diast-C1)

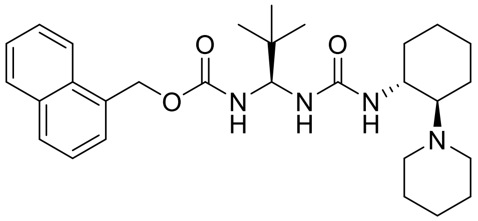

Prepared according to the general procedure starting from (*S*)-3,3-dimethyl-2-(((naphthalen-1-ylmethoxy)carbonyl)amino) butanoic acid (5 mmol). Purified by column chromatography by non-acid silica gel (eluting with Hexane/EtOAc 90/10). White solid. Yield: 63% (1.56 g, 3.15 mmol). m.p.= 169-174 °C. $[\alpha]_D^{23}$ = 13.8 (*c*=0.5, CH_2Cl_2). ^{1}H NMR (300 MHz, CD_3OD) δ: 7.82 (d, *J* = 7.9 Hz, 2H), 7.70 – 7.60 (m, 3H), 7.38 – 7.18 (m, 7H), 7.01 (s, 1H), 6.62 (s, 1H), 5.41 – 5.25 (m, 2H), 3.58 (s, 1H), 3.10 (s, 3H), 2.92 (s, 1H), 1.95 – 1.85 (m, 1H), 1.80 – 1.41 (m, 6H), 1.37 – 0.98 (m, 6H), 0.74 (s, 9H). ^{13}C NMR (75 MHz, CD_3OD) δ: 159.4, 158.1, 135.1, 133.5, 132.7, 129.9, 129.6, 127.8, 127.4, 126.9, 126.2, 124.6, 69.4, 66.9, 65.8, 53.2, 37.0, 34.5, 25.9, 25.5, 25.0, 24.5, 24.2, 23.0. UPLC-DAD-QTOF: $C_{25}H_{41}N_4O_3$ [M+H]+ calcd.: 495.3330, found: 495.3342.

Naphthalen-1-ylmethyl ((*S*)-1-(3-((*S*)-(6-methoxyquinolin-4-yl)((1*S*,2*S*,4*S*,5*R*)-5-vinylquinuclidin-2-yl)methyl)ureido)-2,2-dimethylpropyl)carbamate (C8)

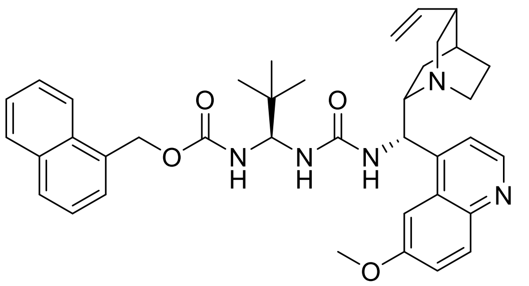

Prepared according to the general procedure starting from (*S*)-3,3-dimethyl-2-(((naphthalen-1-ylmethoxy)carbonyl)amino) butanoic acid (5 mmol). Purified by column chromatography by non-acid silica gel (eluting with Hexane/EtOAc 90/10). White solid. Yield: 65% (2.07 g, 3.25 mmol). m.p.= 163–171 °C. $[\alpha]_D^{23}$ = 21.76 (*c*=0.5, CH_2Cl_2). All the spectroscopic data were coincident with those previously reported. ^{1}H NMR (500 MHz, $CDCl_3$) δ: 8.35 (s, 1H), 8.04 (dd, *J* = 6.6, 3.0 Hz, 1H), 7.96 (d, *J* = 9.2 Hz, 1H), 7.89 – 7.83 (m, 1H), 7.80 (d, *J* = 8.2 Hz, 1H), 7.69 (d, *J* = 2.7 Hz, 1H), 7.55 – 7.47 (m, 3H), 7.39 (d, *J* = 7.8 Hz, 1H), 7.33 (dd, *J* = 9.2, 2.6 Hz, 1H), 7.00 (s, 1H), 6.66 (s, 1H), 5.71 (td, *J* = 17.0, 13.8, 8.5 Hz, 2H), 5.64 – 5.42 (m, 4H), 5.38 (brs, 1H), 5.10 – 4.95 (m, 4H), 3.92 (s, 3H), 3.38 – 2.99 (m, 3H), 2.89 – 2.76 (m, 1H), 2.47 (s, 1H), 1.64 – 1.34 (m, 4H), 0.80 (s, 9H). ^{13}C NMR (75 MHz, $CDCl_3$) δ: 158.1, 157.7, 156.5, 147.5, 144.7, 133.8, 132.1, 131.8, 131.6, 129.4, 128.8, 128.6, 127.8, 126.7, 126.1, 125.4, 123.9, 121.9, 118.8, 115.2, 102.1, 66.9, 65.1, 60.1, 55.8, 55.5, 41.0, 38.8, 35.4, 29.8, 27.2, 25.8, 25.4. UPLC-DAD-QTOF: $C_{38}H_{46}N_5O_4$ [M+H]+ calcd.: 636.3550, found: 636.3551.

2.1.2.1.3. Preparation of benzyl ((S)-1-((3,4-dioxo-2-(((1S,2S)-2-(piperidin-1-yl)cyclohexyl)amino)cyclobut-1-en-1-yl)amino)-2,2-dimethylpropyl)carbamate (**C12**)

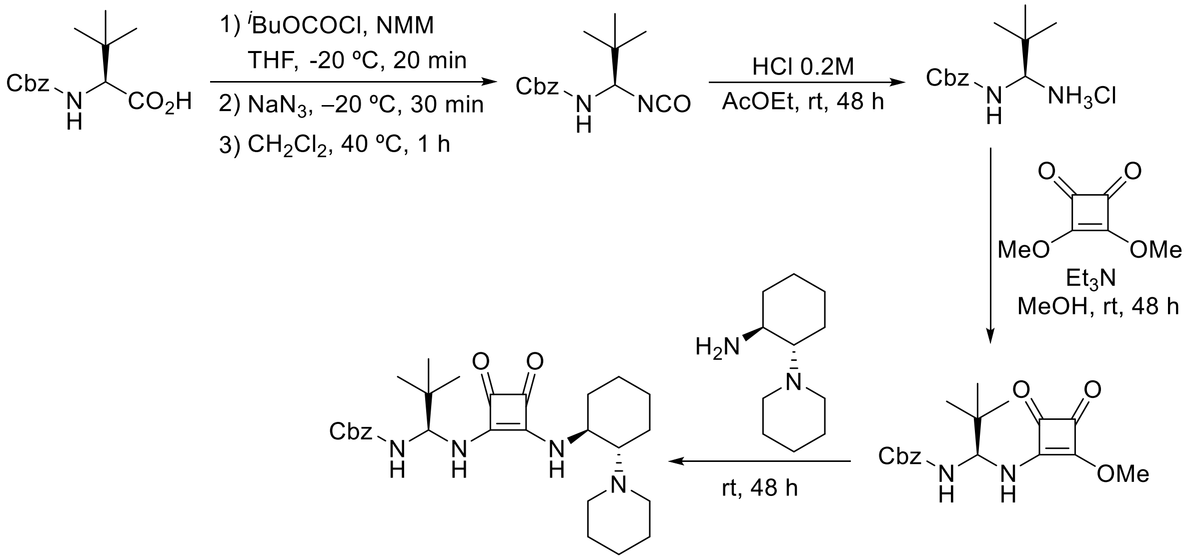

Step 1: To a cooled solution of the corresponding *N*-protected α-amino acid (5 mmol, 1 equiv.) in dry THF (20 mL), were added isobutyl chloroformate (0.65 mL, 5 mmol, 1 equiv.) and *N*-methylmorpholine (0.6 mL, 5 mmol, 1 equiv.) at –20 °C and the mixture was stirred for 20 minutes. Then, a suspension of NaN_3 (0.48 g, 7.5 mmol, 1.5 equiv., in 5 mL of H_2O) was added and the reaction mixture was stirred at the same temperature. After 30 minutes, the organic layer was separated, evaporated and the residue was dissolved in CH_2Cl_2 (30 mL), and washed with water (15 mL). The organic phase was dried over $MgSO_4$ and concentrated in vacuo to give a yellow oil which was dissolved in dry CH_2Cl_2 (10 mL). The resulting solution was heated at 40 °C under nitrogen for 1-2 hours. The reaction was monitored by infrared analysis until disappearance of the isocyanate band. After completion, HCl 0.2M was added (5 mL/1 mmol) and the reaction mixture was stirred at room temperature for 48 hours. The solvents were evaporated under reduced pressure to afford the corresponding salt which was used in the next step without further purification. ^{1}H NMR (300 MHz, DMSO-d_6) δ 8.16 (d, J = 9.2, 3H), 7.41 – 7.30 (m, 5H), 5.13 (s, 2H), 4.64 – 4.54 (m, 1H), 0.97 (s, 9H).

Step 2: To a solution of the amine hydrochloride (5.46 g, 2 mmol, 1 equiv.) and triethylamine (0.84 mL, 6 mmol, 3 equiv.) in MeOH (5 mL/1 mmol), 3,4-dimethoxy-3-cyclobutane-1,2-dione (2.84 g, 2 mmol, 1 equiv.) was added and the reaction mixture was stirred at room temperature for 48 hours. Subsequently, the (1*S*,2*S*)-2-(piperidin-1-yl)cyclohexan-1-amine was added (0.36 g, 2 mmol, 1 equiv.). The suspension was stirred at room temperature for another 48 hours. The solvent was removed under vacuum and the residue was purified by flash column chromatography on silica gel (eluting from 98/2 to 96/4 CH_2Cl_2/MeOH). Yield: 42%, 2 steps (1.04 g, 2.1 mmol). White solid. m.p.= 181–183 °C. $[\alpha]_D^{23}$ = 31.5 (*c*=0.5, EtOAc). ^{1}H NMR (300 MHz, $CDCl_3$) δ: 7.94 – 7.84 (m, 1H), 7.74 – 7.62 (m, 1H), 7.34 (s, 5H), 5.39 (s, 1H), 5.04 (s, 2H), 3.88 – 3.77 (m, 1H), 2.59 (t, *J* = 8.3 Hz, 2H), 2.32 – 2.19 (m, 3H), 2.00 (d, *J* = 10.4 Hz, 1H), 1.87 – 1.77 (m, 1H), 1.75 – 1.60 (m, 2H), 1.43 – 1.12 (m, 11H), 0.92 (s, 9H). ^{13}C NMR (75 MHz, DMSO) δ: 182.5, 181.4, 167.7, 167.2, 155.5, 136.8, 128.3, 127.9, 69.0, 68.3, 65.6, 53.8, 49.2, 35.8, 34.4, 29.0, 26.2, 25.0, 24.8, 24.6, 24.5, 23.6. UPLC-DAD-QTOF: $C_{28}H_{41}N_4O_4$ $[M+H]^+$ calcd.: 497.3128, found: 497.3133.

2.2. Preparation of acyl pyrrol lactims 1

Acyl pyrrol lactims **1** were synthesized following the general procedure described below starting from commercially available α-amino acids, except in the case of the 2-amino-4-phenylbutanoic acid, which was prepared as follows.

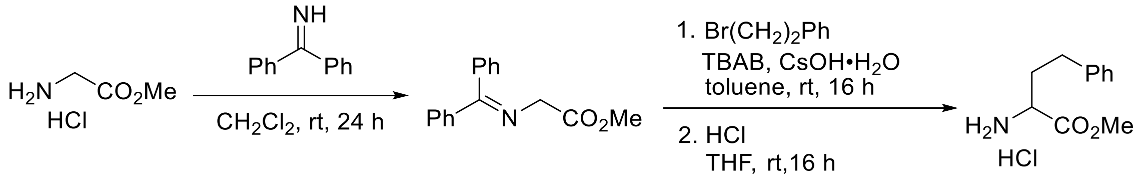

Step 1:[19] A solution of benzophenone imine (1.81 g, 10 mmol, 1 equiv.), glycine methyl ester hydrochloride (10 mmol, 1 equiv.) in CH_2Cl_2 (40 mL) was stirred for 24 hours at room termperature, filtrated and the organic solvent was evaporated *in vacuo*. The crude product was purified by a flash column chromatography (eluting with Hexane/EtOAc 80/20) to afford the desired imine as a white solid (2.33 g, 9.2 mmol, 92%). ^{1}H NMR (300 MHz, $CDCl_3$) δ: 7.74 – 7.63 (m, 2H), 7.54 – 7.45 (m, 3H), 7.44 – 7.32 (m, 3H), 7.24 – 7.18 (m, 2H), 4.24 (s, 2H), 3.77 (s, 3H).

Step 2:[20] To a solution of the imine (2.03 g, 8.00 mmol) in toluene (32 mL) were added a catalytic amount of TBAB (1.16 g, 2.4 mmol, 0.3 equiv.), cesium hydroxide monohydrate (1.48 g, 8.8 mmol, 1.1 equiv.) and (2-bromoethyl)benzene (1.1 mL, 8.00 mmol, 1 equiv.). The reaction mixture was left to stir overnight at room temperature, then the solvent was evaporated under vacuum and the crude was purified by flash column chromatography (eluting with Hexane/EtOAc 80/20) affording the desired product (2.18 g, 6.09 mmol, 76%). ^{1}H NMR (300 MHz, $CDCl_3$) δ: 7.69 (dt, *J* = 8.4, 2.0 Hz, 2H), 7.49 – 7.32 (m, 8H), 7.27 – 7.12 (m, 5H), 4.16 (dd, *J* = 7.6, 5.5 Hz, 1H), 3.73 (s, 3H), 2.62 (dddd, *J* = 35.0, 13.8, 9.8, 6.4 Hz, 2H), 2.28 (dddd, *J* = 14.2, 9.6, 7.8, 3.7 Hz, 2H).

Step 3:[21] To a solution of the α-substituted imine (1.89 g, 5.3 mmol) in THF (7 mL) was added HCl 1M (7 mL) and the reaction was stirred overnight at room temperature. The solvent was evaporated and the crude was used in the next step without further purification (1.2 g, 5.3 mmol, quantitative). ^{1}H NMR (300 MHz, CD_3OD)

[19] Zhang, H.; Syed, S.; Barbas III, C. F. *Org. Lett.* **2010**, *12*, 708-711.

[20] Adapted from: Corey, E. J.; Xu, F.; Noe, M. C. *J. Am. Chem. Soc.* **1997**, *119*, 12414-12415.

[21] Genêt, J.-P.; Jugé, S.; Ruiz Montès, J.; Gaudin, J.-M. *J. Chem. Soc. Chem. Commun.* **1988**, 718-719.

δ: 7.34 –7.09 (m, 5H), 4.04 (t, *J*= 6.4 Hz, 1H), 3.79 (s, 3H), 2.90 –2.58 (m, 2H), 2.35–1.95 (m, 2H).

2.2.1. Acylation of α-amino acids[22]

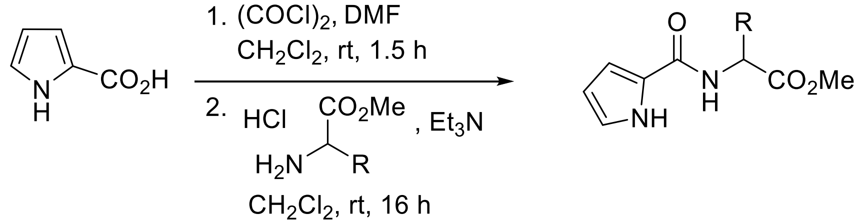

To a mixture of pyrrole-2-carboxylic acid (1.11 g, 10.0 mmol, 1 equiv.) was added oxalyl chloride (1.3 mL, 15 mmol, 1.5 equiv.) at 0 °C and the mixture was left stirring at room temperature for 1.5 hours. Then, the solvent was evaporated.

To a solution of the corresponding α-amino methyl ester hydrochloride (10.0 mmol, 1 equiv.) in dry CH_2Cl_2 (40 mL) at 0 °C, was added Et_3N (20 mmol, 2 equiv.) followed by a solution of the acyl chloride in CH_2Cl_2 (10 mL). After stirring overnight, the reaction mixture was quenched with water and extracted with CH_2Cl_2. The organic layer was washed with HCl 1M, $NaHCO_3$ and brine, dried over $MgSO_4$, filtrated, concentrated and purified by flash chromatography (eluting with Hexane/EtOAc 70/30).

Methyl (1*H*-pyrrole-2-carbonyl)-*L*-phenylalaninate

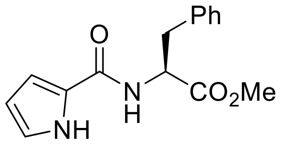

The title compound was prepared following the general procedure starting from *L*-phenylalanine methyl ester hydrochloride (2.5 g, 9.2 mmol, 92%). ^1H NMR (300 MHz, $CDCl_3$) δ: 9.26 (s, 1H), 7.31–7.20 (m, 3H), 7.13 (dd, *J* = 7.6, 1.8 Hz, 2H), 6.93 (td, *J* = 2.7, 1.3 Hz, 1H), 6.53 (ddd, *J* = 3.8, 2.5, 1.3 Hz, 1H), 6.26 (s, 1H), 6.23 (dt, *J* = 3.8, 2.6 Hz, 1H) 5.05 (td, *J* = 7.8 and 5.7 Hz, 1H), 3.73 (s, 3H), 3.21 (dd, *J* = 5.7, 1.4 Hz, 2H).

Methyl (1*H*-pyrrole-2-carbonyl)-*L*-leucinate

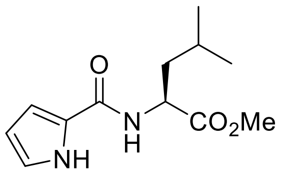

The title compound was prepared following the general procedure starting from *L*-leucine methyl ester hydrochloride (2.0 g, 8.4 mmol, 84%). ^1H NMR (300 MHz, $CDCl_3$) δ: 9.37 (s, 1H), 6.93 (td, *J* = 2.7, 1.3 Hz, 1H), 6.63 (ddd, *J* = 3.8, 2.5, 1.3 Hz, 1H),

---

[22] Tian, H.; Ermolenko, L.; Gabant, M.; Vergne, C.; Moriou, C.; Retailleau, P.; Al-Mourabit, L. *Adv. Synth. Catal.* **2011**, *353*, 1525-1533.

6.25 (dt, J = 3.8, 2.6 Hz, 1H), 6.2 (brs, J = 8.6 Hz, 1H), 4.81 (td, J = 8.7, 5.2 Hz, 1H), 3.76 (s, 3H), 1.79 – 1.62 (m, 3H), 0.97 (dd, J = 6.1, 4.9 Hz, 6H).

Methyl (*S*)-3-(4-methoxyphenyl)-2-(1*H*-pyrrole-2-carboxamido)propanoate

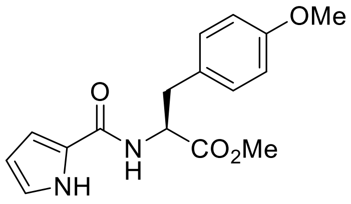

The title compound was prepared following the general procedure starting from *L*-tyrosine methyl ester hydrochloride (2.6 g, 8.7 mmol, 87%). ^{1}H NMR (300 MHz, $CDCl_3$) δ: 9.29 (s, 1H), 7.08 – 7.00 (m, 2H), 6.93 (td, J = 2.7, 1.3 Hz, 1H), 6.88 – 6.77 (m, 2H), 6.54 (ddd, J = 3.8, 2.5, 1.3 Hz, 1H), 6.25 (ddt, J = 11.3, 3.8, 2.6 Hz, 2H), 5.00 (dt, J = 7.9, 5.5 Hz, 1H), 3.79 (s, 3H), 3.75 (s, 3H), 3.15 (d, J = 5.5 Hz, 2H).

Methyl (1*H*-pyrrole-2-carbonyl)-*L*-tryptophanate

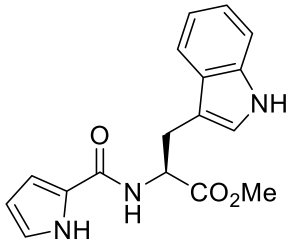

The title compound was prepared following the general procedure starting with the *L*-tryptophan methyl ester hydrochloride (2.49 g, 8.0 mmol, 80%). ^{1}H NMR (300 MHz, $CDCl_3$) δ: 9.27 (s, 1H), 8.09 (s, 1H), 7.59 (dd, J = 7.7, 1.1 Hz, 1H), 7.40 (dt, J = 8.1, 0.9 Hz, 1H), 7.29 – 7.20 (m, 1H), 7.14 (td, J = 7.6, 7.1, 1.1 Hz, 1H), 7.05 (d, J = 2.4 Hz, 1H), 6.94 (ddd, J = 4.0, 2.5, 1.3 Hz, 1H), 6.44 (ddd, J = 3.8, 2.5, 1.3 Hz, 1H), 6.37 (d, J = 7.9 Hz, 1H), 6.22 (dt, J = 3.9, 2.6 Hz, 1H), 5.13 (dt, J = 8.1, 5.2 Hz, 1H), 3.72 (s, 3H), 3.43 (t, J = 4.9 Hz, 1H).

Methyl 4-phenyl-2-(1*H*-pyrrole-2-carboxamido)butanoate

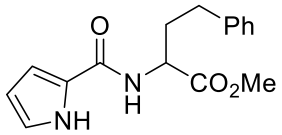

The title compound was prepared following the general procedure starting from *D,L*-homophenyl alanine methyl ester hydrochloride (2.6 g, 8.9 mmol, 89%). ^{1}H NMR (300 MHz, $CDCl_3$) δ: 9.94 (s, 1H), 7.35 – 7.24 (m, 2H), 7.22 (td, J = 6.2, 1.6 Hz, 2H), 6.94 (q, J = 1.4 Hz, 1H), 6.61 (ddd, J = 3.8, 2.5, 1.3 Hz, 1H), 6.54 (d, J = 8.0 Hz, 1H), 6.25 (dt, J = 3.8, 2.6 Hz, 1H), 4.87 (td, J = 7.7, 5.1 Hz, 1H), 3.77 (s, 3H), 2.79 – 2.69 (m, 2H), 2.40 – 2.22 (m, 1H), 2.21 – 2.07 (m, 1H). ^{13}C NMR (75 MHz, $CDCl_3$) δ: 173.1, 161.0, 140.8, 128.7, 128.6, 126.4, 125.4, 122.2, 110.0, 52.6, 52.0, 34.2, 31.9. UPLC-DAD-QTOF: $C_{16}H_{19}N_2O_3$ [M+H]+ calcd.: 287.1390, found: 287.1388.

Methyl 2-(1*H*-pyrrole-2-carboxamido)pent-4-enoate

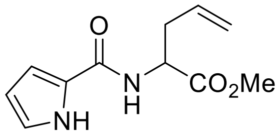

The title compound was prepared following the general procedure starting with the *D,L*-allylglycine methyl ester hydrochloride (1.9 g, 8.5 mmol, 85%). ^{1}H NMR (300 MHz, $CDCl_3$) δ: 10.67 (s, 1H), 6.93 (td, J = 2.7, 1.3 Hz, 1H), 6.62 (ddd, J = 3.8,

2.5, 1.3 Hz, 1H), 6.35 (d, *J* = 7.9 Hz, 1H), 6.25 (dt, *J* = 3.8, 2.6 Hz, 1H), 5.74 (ddt, *J* = 17.1, 9.7, 7.2 Hz, 1H), 5.23 – 5.14 (m, 1H), 5.13 (dt, *J* = 2.3, 1.2 Hz, 1H), 4.84 (dt, *J* = 7.9, 5.8 Hz, 1H), 3.78 (s, 3H), 2.64 (m, 2H). ^{13}C NMR (75 MHz, $CDCl_3$) δ: 172.7, 161.2, 132.4, 125.1, 122.4, 119.0, 110.3, 109.5, 52.4, 51.7, 36.5. UPLC-DAD-QTOF: $C_{11}H_{15}N_2O_3$ [M+H]+ calcd.: 223.1077, found: 223.1080.

Methyl (*S*)-2-phenyl-2-(1*H*-pyrrole-2-carboxamido)acetate

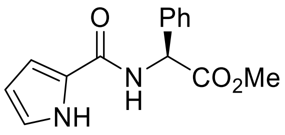

The title compound was prepared following the general procedure starting from *L*-phenylglycine methyl ester hydrochloride (1.9 g, 7.4 mmol, 74%). ^{1}H NMR (300 MHz, $CDCl_3$) δ: 9.67 (s, 1H), 7.48 – 7.29 (m, 5H), 6.93 – 6.81 (m, 2H), 6.69 (ddd, *J* = 3.8, 2.5, 1.3 Hz, 1H), 6.23 (ddt, *J* = 7.3, 3.5, 2.5 Hz, 1H), 5.73 (d, *J* = 7.0 Hz, 1H), 3.76 (s, 3H). ^{13}C NMR (75 MHz, $CDCl_3$) δ: 171.6, 160.5, 136.8, 129.1, 128.7, 127.4, 125.2, 122.3, 110.1, 110.0, 56.5, 53.0. UPLC-DAD-QTOF: $C_{14}H_{15}N_2O_3$ [M+H]+ calcd.: 259.1077, found: 259.1085.

Methyl 2-(1*H*-pyrrole-2-carboxamido)octanoate

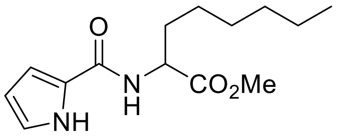

The title compound was prepared following the general procedure starting from methyl *D,L*-2-aminooctanoate hydrochloride (1.9 g, 7.0 mmol, 70%). ^{1}H NMR (300 MHz, $CDCl_3$) δ: 9.29 (s, 1H), 6.93 (td, *J* = 2.7, 1.3 Hz, 1H), 6.64 (ddd, *J* = 3.8, 2.5, 1.3 Hz, 1H), 6.30 (d, *J* = 8.3 Hz, 1H), 6.25 (dt, *J* = 3.7, 2.6 Hz, 1H), 4.76 (td, *J* = 7.7, 5.4 Hz, 1H), 3.77 (s, 3H), 1.91 (dq, *J* = 15.0, 4.9 Hz, 1H), 1.84 – 1.65 (m, 1H), 1.45 – 1.19 (m, 8H), 0.87 (t, *J* = 6.8 Hz, 3H). ^{13}C NMR (75 MHz, $CDCl_3$) δ: 173.7, 161.3, 125.2, 122.3, 110.4, 109.5, 52.3, 52.2, 32.4, 31.5, 28.8, 25.4, 22.5, 14.0. UPLC-DAD-QTOF: $C_{14}H_{23}N_2O_3$ [M+H]+ calcd.: 267.1703, found: 266.1699.

2.2.2. Cyclization of pyrrol esters[22]

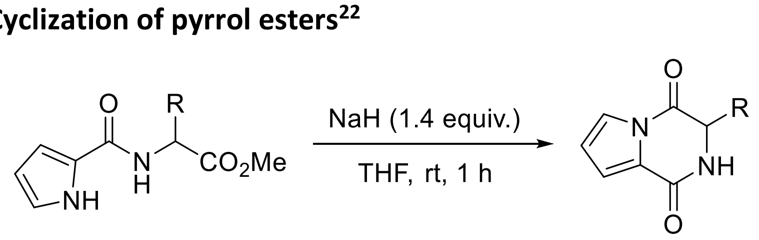

The corresponding methyl ester (5 mmol, 1 equiv.) was dissolved in degassed anhydrous THF (50 mL) and cooled to 0 °C. Sodium hydride (7 mmol, 1.4 equiv.) was added and the mixture was stirred at 0 °C for five minutes and then at room temperature for 1 hour. After reaction completion, the mixture was poured into acetate buffer pH 3.8 (30 mL) and quickly extracted with AcOEt. The organic layer was dried over $MgSO_4$, the solvent was evaporated and the residue was dried under vacuum. The compound was stored and used without further purification.

(*S*)-3-Benzyl-2,3-dihydropyrrolo[1,2-*a*]pyrazine-1,4-dione

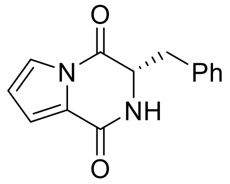

The title compound was prepared following the general procedure starting with the methyl (1*H*-pyrrole-2-carbonyl)-*L*-phenylalaninate (0.95 g, 3.95 mmol, 79%). ^{1}H NMR (300 MHz, $CDCl_3$) δ: 7.53 (dd, *J* = 3.2, 1.5 Hz, 1H), 7.38–7.21 (m, 5H), 7.08 (dd, *J* = 3.5, 1.5 Hz, 1H), 6.51 (t, *J* = 3.4 Hz, 1H), 5.63 (s, 1H), 4.60 (ddd, *J* = 9.6, 3.6, 2.1 Hz, 1H), 3.55 (dd, *J* = 13.7, 3.6 Hz, 1H), 3.02 (dd, *J* = 13.7, 9.6 Hz, 1H).

(*S*)-3-Isobutyl-2,3-dihydropyrrolo[1,2-*a*]pyrazine-1,4-dione

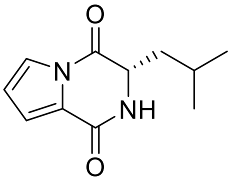

The title compound was prepared following the general procedure starting with the methyl (1*H*-pyrrole-2-carbonyl)-*L*-leucinate (0.77 g, 3.75 mmol, 75%). ^{1}H NMR (300 MHz, $CDCl_3$) δ: 7.53 (dd, *J* = 3.2, 1.5 Hz, 1H), 7.14 (dd, *J* = 3.5, 1.5 Hz, 1H), 6.53 (t, *J* = 3.3 Hz, 1H), 5.99 (s, 1H), 4.42 (ddd, *J* = 8.8, 4.2, 2.1 Hz, 1H), 1.99 – 1.77 (m, 3H), 0.99 (t, *J* = 6.2 Hz, 8H).

(*S*)-3-(4-Methoxybenzyl)-2,3-dihydropyrrolo[1,2-*a*]pyrazine-1,4-dione

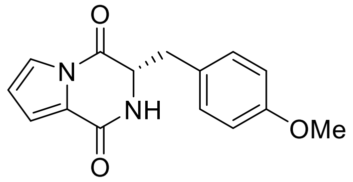

The title compound was prepared following the general procedure starting with the methyl (*S*)-3-(4-methoxyphenyl)-2-(1*H*-pyrrole-2-carboxamido) propanoate (1.08 g, 4.0 mmol, 80%). ^{1}H NMR (300 MHz, $CDCl_3$) δ: 7.52 (dd, *J* = 3.2, 1.5 Hz, 1H), 7.19 – 7.08 (m, 2H), 7.07 (dd, *J* = 3.5, 1.5 Hz, 1H), 6.91 – 6.80 (m, 2H), 6.50 (t, *J* = 3.3 Hz, 1H), 5.78 (s, 1H), 4.55 (ddd, *J* = 9.2, 3.8, 2.1 Hz, 1H), 3.45 (dd, *J* = 13.8, 3.7 Hz, 1H), 2.99 (dd, *J* = 13.8, 9.2 Hz, 1H), 2.04 (s, 3H).

(*S*)-3-((1*H*-Indol-3-yl)methyl)-2,3-dihydropyrrolo[1,2-*a*]pyrazine-1,4-dione

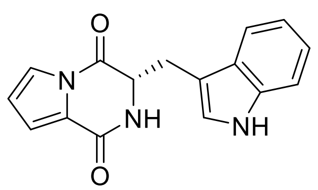

The title compound was prepared following the general procedure starting with the methyl (1*H*-pyrrole-2-carbonyl)-*L*-tryptophanate (1.01 g, 3.6 mmol, 72%). ^{1}H NMR (300 MHz, $CDCl_3$) δ: 8.18 (s, 1H), 7.63 (ddd, *J* = 8.0, 1.3, 0.8 Hz, 1H), 7.51 (dd, *J* = 3.2, 1.5 Hz, 1H), 7.43 – 7.33 (m, 1H), 7.26 – 7.12 (m, 2H), 7.06 (dd, *J* = 3.5, 1.5 Hz, 1H), 6.47 (t, *J* = 3.3 Hz, 1H), 5.87 (s, 1H), 4.68 (ddd, *J* = 9.6, 3.5, 2.0 Hz, 1H), 3.72 (ddd, *J* = 14.4, 3.5, 0.9 Hz, 1H), 3.22 (dd, *J* = 14.4, 9.6 Hz, 1H).

3-Phenethyl-2,3-dihydropyrrolo[1,2-*a*]pyrazine-1,4-dione

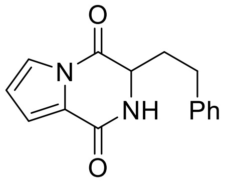

The title compound was prepared following the general procedure starting with the methyl 4-phenyl-2-(1*H*-pyrrole-2-carboxamido)butanoate (1.08 g, 4.25 mmol, 85%). ^{1}H NMR (300 MHz, $CDCl_3$) δ: 7.49 (dd, *J* = 3.2, 1.5 Hz, 1H), 7.34 – 7.24 (m, 1H), 7.24 – 7.11 (m, 5H), 6.53 (t, *J* = 3.4 Hz, 1H), 5.88 (s, 1H) 4.44 (td, *J* = 5.5, 2.0 Hz, 1H), 2.84 – 2.72 (m, 2H), 2.40 – 2.31 (m, 2H).

3-Allyl-2,3-dihydropyrrolo[1,2-*a*]pyrazine-1,4-dione

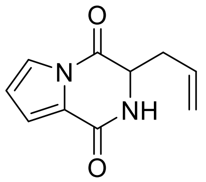

The title compound was prepared following the general procedure starting with the methyl 2-(1*H*-pyrrole-2-carboxamido)pent-4-enoate (0.74 g, 3.9 mmol, 78 %). ^{1}H NMR (300 MHz, $CDCl_3$) δ: 7.53 (dd, *J* = 3.2, 1.5 Hz, 1H), 7.15 (dd, *J* = 3.5, 1.5 Hz, 1H), 6.54 (t, *J* = 3.3 Hz, 1H), 5.26 (d, *J* = 10.7 Hz, 2H), 4.50 – 4.40 (m, 1H), 2.99 – 2.85 (m, 2H).

(*S*)-3-Phenyl-2,3-dihydropyrrolo[1,2-*a*]pyrazine-1,4-dione

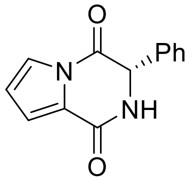

The title compound was prepared following the general procedure starting with the methyl (*S*)-2-phenyl-2-(1*H*-pyrrole-2-carboxamido)acetate (0.90 g, 4.0 mmol, 80 %). ^{1}H NMR (300 MHz, $CDCl_3$) δ: 7.59 (d, *J* = 8.0 Hz, 1H), 7.49 (dd, *J* = 3.2, 1.5 Hz, 1H), 7.44 – 7.31 (m, 5H), 7.23 (dd, *J* = 3.5, 1.5 Hz, 1H), 6.55 (t, *J* = 3.3 Hz, 1H), 5.46 (d, *J* = 2.1 Hz, 1H).

3-Hexyl-2,3-dihydropyrrolo[1,2-*a*]pyrazine-1,4-dione

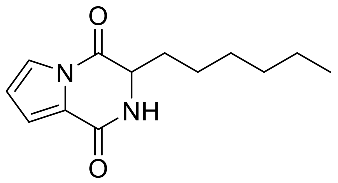

The title compound was prepared following the general procedure starting with the methyl 2-(1*H*-pyrrole-2-carboxamido)octanoate (0.84 g, 3.6 mmol, 71 %). ^{1}H NMR (300 MHz, $CDCl_3$) δ: 7.53 (dd, *J* = 3.2, 1.5 Hz, 1H), 7.14 (dd, *J* = 3.5, 1.5 Hz, 1H), 6.53 (t, *J* = 3.3 Hz, 1H), 6.19 (s, 1H), 4.43 (t, *J* = 5.3 Hz, 1H), 1.74 (dt, *J* = 14.9, 5.2 Hz, 2H), 1.38 – 1.23 (m, 8H), 0.87 (t, *J* = 6.7 Hz, 3H).

2.2.3. Preparation of the pyrrol lactims 1

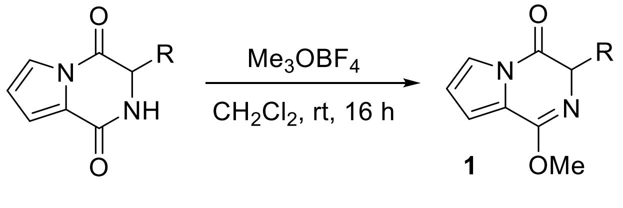

To a well-stirred solution of the corresponding diketopiperazine (3 mmol, 1 equiv.) in CH_2Cl_2 (15 mL), trimethyloxonium tetrafluoroborate (3.3 mmol, 1.1 equiv.) was

added in one portion. The reaction mixture was stirred at room temperature overnight. After reaction completion, the reaction mixture was quenched with a saturated aqueous sodium carbonate solution and extracted with CH_2Cl_2, dried over $MgSO_4$, filtered and concentrated under reduced pressure. The crude product was purified by flash column chromatography (eluting with Hexane/EtOAc 90/10).

(*S*)-3-Benzyl-1-methoxypyrrolo[1,2-*a*]pyrazin-4(3*H*)-one (1a)

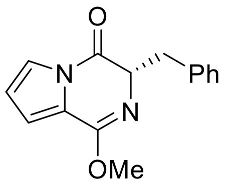

The title compound was prepared following the general procedure starting with the (*S*)-3-benzyl-2,3-dihydropyrrolo[1,2-*a*]pyrazine-1,4-dione (0.661 g, 2.6 mmol, 87%). ^1H NMR (300 MHz, $CDCl_3$) δ: 7.54 (dd, *J* = 3.2, 1.5 Hz, 1H), 7.33 – 7.22 (m, 5H), 6.55 (dd, *J* = 3.3, 1.4 Hz, 1H), 6.45 (t, *J* = 3.3 Hz, 1H), 4.90 (t, *J* = 5.3 Hz, 1H), 3.99 (s, 3H), 3.48 (t, *J* = 5.1 Hz, 2H). ^{13}C NMR (75 MHz, $CDCl_3$) δ: 169.7, 152.8, 136.2, 129.7, 128.0, 126.8, 122.7, 117.6, 114.7, 112.2, 63.2, 52.9, 41.0. UPLC-DAD-QTOF: $C_{15}H_{16}N_2O_2$ [M+H]+ calcd.: 255.1134, found: 255.1141.

(*S*)-3-Isobutyl-1-methoxypyrrolo[1,2-*a*]pyrazin-4(3*H*)-one (1b)

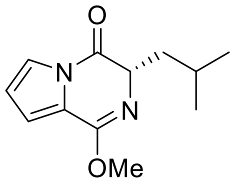

The title compound was prepared following the general procedure starting with the (*S*)-3-isobutyl-2,3-dihydropyrrolo[1,2-*a*]pyrazine-1,4-dione (0.551 g, 2.5 mmol, 82%). ^1H NMR (300 MHz, $CDCl_3$) δ: 7.48 (dd, *J* = 3.2, 1.3 Hz, 1H), 6.65 – 6.56 (m, 1H), 6.44 (dd, *J* = 3.7, 2.8 Hz, 1H), 4.47 (dd, *J* = 8.8, 4.9 Hz, 2H), 3.86 (s, 3H), 2.12 – 1.83 (m, 2H), 1.67 (ddd, *J* = 13.1, 8.8, 5.5 Hz, 1H), 1.00 (d, *J* = 6.5 Hz, 3H), 0.91 (d, *J* = 6.6 Hz, 3H). ^{13}C NMR (75 MHz, $CDCl_3$) δ: 171.6, 152.5, 123.3, 118.4, 115.2, 112.6, 61.3, 53.4, 45.1, 25.5, 23.7, 22.6. UPLC-DAD-QTOF: $C_{12}H_{18}N_2O_2$ [M+H]+ calcd.: 221.1290, found: 221.1295.

(*S*)-1-Methoxy-3-(4-methoxybenzyl)pyrrolo[1,2-*a*]pyrazin-4(3*H*)-one (1c)

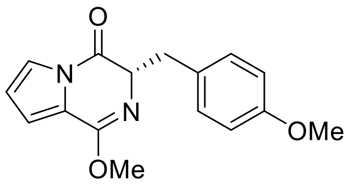

The title compound was prepared following the general procedure starting with the (*S*)-3-(4-methoxybenzyl)-2,3-dihydropyrrolo[1,2-*a*]pyrazine-1,4-dione (0.654 g, 2.3 mmol, 77%). ^1H NMR (300 MHz, $CDCl_3$) δ: 7.41 (dd, *J* = 3.2, 1.5 Hz, 1H), 7.10 – 6.99 (m, 2H), 6.44 (ddd, *J* = 3.4, 1.5, 0.6 Hz, 1H), 6.32 (t, *J* = 3.3 Hz, 1H), 4.74 (t, *J* = 5.2 Hz, 1H), 3.88 (s, 3H), 3.71 (s, 3H), 3.31 (d, *J* = 5.2 Hz, 2H). ^{13}C NMR (75 MHz, $CDCl_3$) δ: 169.6, 158.5, 152.7, 130.6, 128.2, 122.7, 117.4, 114.6, 113.4, 112.1, 63.3, 55.1, 52.8, 40.0. UPLC-DAD-QTOF: $C_{16}H_{17}N_2O_3$ [M+H]+ calcd.: 285.1239, found: 285.1239.

1-Methoxy-3-phenethylpyrrolo[1,2-*a*]pyrazin-4(3*H*)-one (1d)

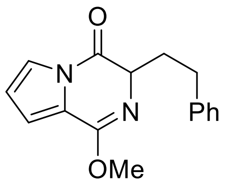

The title compound was prepared following the general procedure starting with the 3-phenethyl-2,3-dihydropyrrolo[1,2-*a*]pyrazine-1,4-dione (0.698 g, 2.6 mmol, 88%). ^{1}H NMR (300 MHz, $CDCl_3$) δ: 7.46 (dd, *J* = 3.2, 1.5 Hz, 1H), 7.32 – 7.11 (m, 5H), 6.64 (ddd, *J* = 3.4, 1.4, 0.6 Hz, 1H), 6.44 (t, *J* = 3.3 Hz, 1H), 4.48 (dd, *J* = 7.4, 4.7 Hz, 1H), 3.91 (s, 3H), 2.88 – 2.65 (m, 2H), 2.44 (dddd, *J* = 13.8, 9.1, 7.1, 4.8 Hz, 1H), 2.23 (dddd, *J* = 13.4, 9.1, 7.4, 6.0 Hz, 1H). ^{13}C NMR (75 MHz, $CDCl_3$) δ: 170.3, 152.5, 141.2, 128.7, 128.4, 126.1, 122.8, 117.9, 114.8, 112.3, 61.3, 52.9, 36.6, 31.6. UPLC-DAD-QTOF: $C_{16}H_{17}N_2O_2$ [M+H]+ calcd.: 269.1290, found: 269.1293.

(*S*)-3-((1*H*-Indol-3-yl)methyl)-1-methoxypyrrolo[1,2-*a*]pyrazin-4(3*H*)-one (1e)

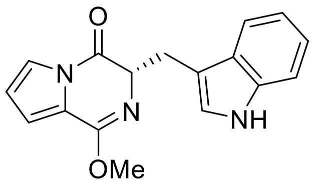

The title compound was prepared following the general procedure starting with the (*S*)-3-((1*H*-indol-3-yl)methyl)-2,3-dihydropyrrolo[1,2-*a*]pyrazine-1,4-dione (0.616 g, 2.1 mmol, 71%). ^{1}H NMR (300 MHz, $CDCl_3$) δ: 8.03 (s, 1H), 7.70 (dq, *J* = 7.2, 0.8 Hz, 1H), 7.36 (dd, *J* = 3.2, 1.5 Hz, 1H), 7.26 – 7.21 (m, 1H), 7.11 (dtd, *J* = 14.0, 7.0, 1.4 Hz, 2H), 6.93 (d, *J* = 2.5 Hz, 1H), 6.38 (dd, *J* = 3.4, 1.5 Hz, 1H), 6.26 (t, *J* = 3.3 Hz, 1H), 4.85 (t, *J* = 5.0 Hz, 1H), 3.86 (s, 3H), 3.59 (dt, *J* = 4.9, 0.8 Hz, 2H). ^{13}C NMR (75 MHz, $CDCl_3$) δ: 170.3, 152.8, 135.9, 128.0, 123.0, 122.8, 121.9, 119.5, 119.3, 117.5, 114.5, 112.0, 110.9, 110.6, 63.4, 60.5, 52.8, 30.8. UPLC-DAD-QTOF: $C_{17}H_{16}N_3O_2$ [M+H]+ calcd.: 294.1243, found: 294.1242.

3-Allyl-1-methoxypyrrolo[1,2-*a*]pyrazin-4(3*H*)-one (1f)

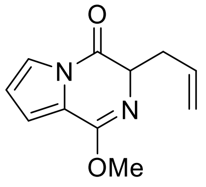

The title compound was prepared following the general procedure starting with the 3-allyl-2,3-dihydropyrrolo[1,2-a]pyrazine-1,4-dione (0.531 g, 2.6 mmol, 88%). ^{1}H NMR (300 MHz, $CDCl_3$) δ: 7.45 (dd, *J* = 3.2, 1.4 Hz, 1H), 6.58 (ddt, *J* = 3.5, 1.3, 0.6 Hz, 1H), 6.40 (td, *J* = 3.3, 0.9 Hz, 1H), 5.64 (ddt, *J* = 17.2, 10.1, 7.2 Hz, 1H), 5.08 (ddd, *J* = 17.2, 2.2, 1.1 Hz, 1H), 4.98 (ddt, *J* = 10.2, 2.0, 1.0 Hz, 1H), 4.53 (t, *J* = 5.6 Hz, 1H), 3.84 (s, 3H), 2.88 – 2.63 (m, 2H). ^{13}C NMR (75 MHz, $CDCl_3$) δ: 169.6, 152.7, 132.6, 122.8, 118.8, 117.8, 117.7, 114.8, 112.3, 62.0, 52.9, 39.2. UPLC-DAD-QTOF: $C_{11}H_{13}N_2O_2$ [M+H]+ calcd.: 205.0977, found: 205.0978.

(*S*)-1-Methoxy-3-phenylpyrrolo[1,2-*a*]pyrazin-4(3*H*)-one (1g)

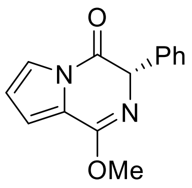

The title compound was prepared following the general procedure starting with the 3-phenyl-2,3-dihydropyrrolo[1,2-*a*]pyrazine-1,4-dione (0.649 g, 2.7 mmol, 90%). ^{1}H NMR (300 MHz, $CDCl_3$) δ: 7.49 (dd, *J* = 3.2, 1.5 Hz, 1H), 7.48 – 7.29 (m, 5H), 6.76 (dd, *J* = 3.4, 1.4 Hz, 1H), 6.47 (td, *J*

= 3.3, 1.6 Hz, 1H), 5.64 (s, 1H), 3.99 (s, 3H). ^{13}C NMR (75 MHz, $CDCl_3$) δ: 167.6, 152.9, 137.1, 128.2, 127.6, 127.0, 122.1, 118.1, 114.4, 112.5, 65.3, 52.6. UPLC-DAD-QTOF: $C_{14}H_{13}N_2O_2$ [M+H]+ calcd.: 241.0977, found: 241.0979.

3-Hexyl-1-methoxypyrrolo[1,2-*a*]pyrazin-4(3*H*)-one (1h)

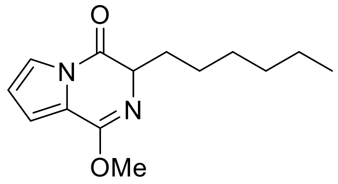

The title compound was prepared following the general procedure starting with the 3-hexyl-2,3-dihydropyrrolo[1,2-*a*]pyrazine-1,4-dione (0.621 g, 2.5 mmol, 84%). ^{1}H NMR (300 MHz, $CDCl_3$) δ: 7.41 (dt, *J* = 3.2, 1.7 Hz, 1H), 6.52 (dq, *J* = 3.3, 1.6 Hz, 1H), 6.39 – 6.32 (m, 1H), 4.39 (ddd, *J* = 6.8, 4.8, 1.8 Hz, 1H), 3.78 (s, 3H), 1.99 (ddd, *J* = 14.4, 7.1, 4.0 Hz, 1H), 1.91 – 1.77 (m, 1H), 1.36 – 1.14 (m, 8H), 0.78 (td, *J* = 6.9, 6.3, 2.5 Hz, 3H). ^{13}C NMR (75 MHz, $CDCl_3$) δ: 170.3, 152.2, 122.7, 117.5, 114.5, 112.0, 62.0, 52.6, 35.2, 31.6, 29.0, 24.9, 22.5, 14.0. UPLC-DAD-QTOF: $C_{14}H_{21}N_2O_2$ [M+H]+ calcd.: 249.1603, found: 249.1607.

2.3. Michael addition of 1-methoxypyrrolo[1,2-*a*]pyrazin-4(3*H*)-ones 1 to nitroalkenes 2.

2.3.1. Catalyst screening

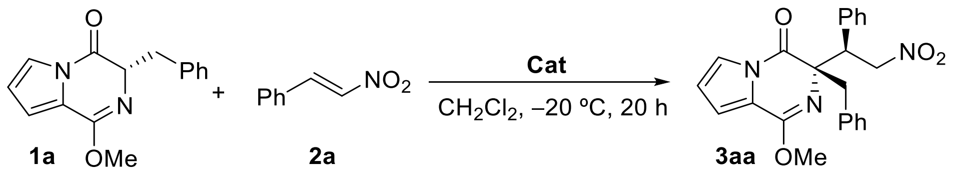

1a **2a** **3aa**

Cat.	Yield (%)	*dr*[a]	*ee* (%)[a]
**C1**	87	94:6	80
**C2**	84	92:8	81
**C3**	86	94:6	81
**C4**	88	93:7	79
**C5**	86	93:7	75
**C6**	86	91:9	34
**C7**	84	95:5	84
Diast-**C1**	81	85:15	-59
**C8**	84	78:22	63
**C9**	85	93:7	52
**C10**	74	92:8	0
**C11**	83	96:4	82
**C12**	66	89:11	52[b]
**C13**	81	88:12	-68

The reactions were performed using 0.1 mmol of **1a**, 0.15 mmol of nitrostyrene **2a** and 10 mol% catalyst in 0.3 mL of CH_2Cl_2. [a] *dr* and *ee* of major diastereomer as determined by HPLC. [b] 85% conversion in 116 h.

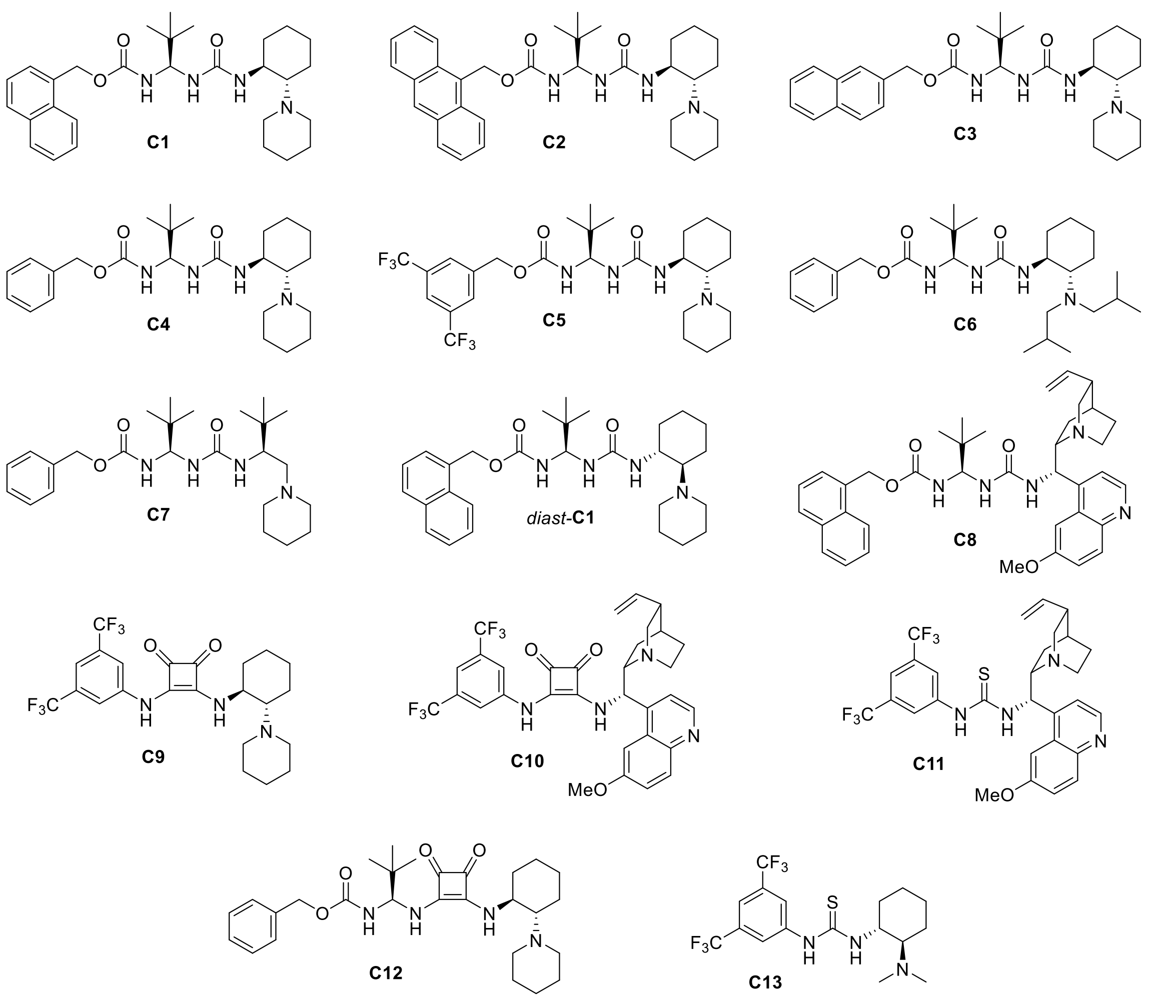

2.3.2. Solvent screening

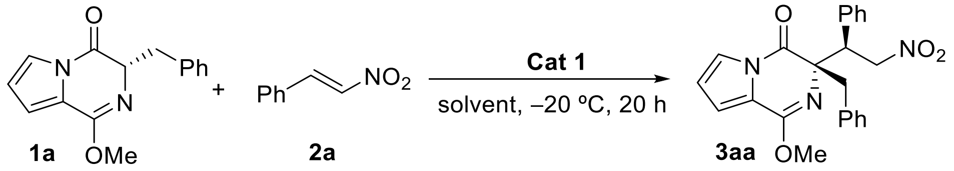

Solvent	conv. (%)	Yield (%)	d.r.[b]	ee, %[b]
CH_2Cl_2	>95	87	94:6	80
MeCN	>95	89	54:46	35
EtOAc	>95	83	95:5	70
Toluene	>95	85	98:2	88
$PhCF_3$	>95	84	96:4	84
m-xylene	>95	83	97:3	84

The reactions were performed using 0.1 mmol of **1a**, 0.15 mmol of nitrostyrene **2a** and 10 mol% catalyst in 0.3 mL of CH_2Cl_2. [a] *dr* and *ee* of major diastereomer as determined by HPLC.

2.3.3. Reaction scope

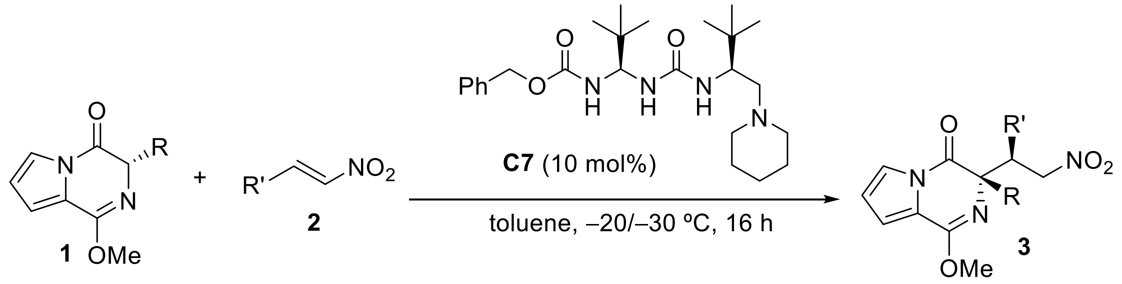

To a solution of the corresponding pyrrol lactim **1** (1 equiv., 0.1 mmol) and nitroalkene **2** (3.0 equiv., 0.3 mmol) in toluene (0.3 mL) catalyst **C7** (0.01 mmol) was added at the indicated temperature. The resulting mixture was stirred until consumption of the starting material (typically 16 h). The solvent was eliminated and the crude was purified by flash column chromatograph on silica gel to afford the expected adduct **3**.

(*S*)-3-Benzyl-1-methoxy-3-((*S*)-2-nitro-1-phenylethyl)pyrrolo[1,2-*a*]pyrazin-4(3*H*)-one (3aa)

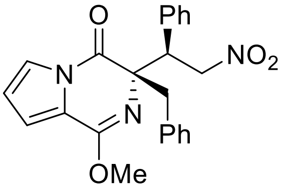

The title compound was prepared starting from (*S*)-3-benzyl-1-methoxypyrrolo[1,2-*a*]pyrazin-4(3*H*)-one **1a** according to the general procedure at –20 ºC at 1 mmol scale. The crude material was purified by flash column chromatography eluting 1:1 Hexane:CH_2Cl_2. Colourless solid. Yield: 315 mg, 0.78 mmol, 78%. m.p. 118-123 °C. $[\alpha]_D^{21}$= +34.9 (c=1, CH_2Cl_2). *dr* 98:2, 88% *ee*. ^{1}H NMR (300 MHz, $CDCl_3$) δ: 7.57 (dd, *J* = 7.6, 2.0 Hz, 2H), 7.40 – 7.30 (m, 4H), 6.99 (dd, *J* = 5.0, 2.0 Hz, 3H), 6.82 (dd, *J* = 6.4, 3.1 Hz, 2H), 6.21 (d, *J* = 2.3 Hz, 2H), 4.83 (dd, *J* = 12.4, 10.4 Hz, 1H), 4.61 – 4.43 (m, 2H), 3.98 (s, 3H), 3.21 (d, *J* = 12.5 Hz, 1H), 2.66 (d, *J* = 12.5 Hz, 1H). ^{13}C NMR (75 MHz, $CDCl_3$) δ: 170.8, 153.2, 135.4, 134.1, 130.0, 129.7, 128.5, 128.4, 127.8, 127.1, 122.0, 117.8, 115.2, 112.5, 77.5, 71.0, 53.2, 51.5, 46.6. UPLC-DAD-QTOF: $C_{23}H_{22}N_3O_4$ [M+H]+ calcd.: 404.1610, found: 404.1613.

(*S*)-3-Benzyl-3-((*S*)-1-(4-bromophenyl)-2-nitroethyl)-1-methoxypyrrolo[1,2-*a*]pyrazin-4(3*H*)-one (3ab)

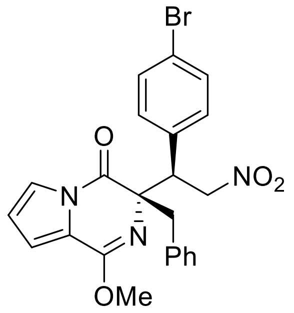

The title compound was prepared starting from (*S*)-3-benzyl-1-methoxypyrrolo[1,2-*a*]pyrazin-4(3*H*)-one **1a** according to the general procedure at –20 °C. The crude material was purified by flash column chromatography eluting 1:1 Hexane:CH_2Cl_2. Colourless solid. Yield: 42 mg, 0.086 mmol, 86%. m.p.= 66-69 °C. $[\alpha]_D^{21}$= +38.7 (c=1, CH_2Cl_2). *dr* 96:4, 85% *ee*. ^{1}H NMR (300 MHz, $CDCl_3$) δ: 7.48 (d, *J* = 2.2 Hz, 4H), 7.32 (dd, *J* = 2.8, 1.9 Hz, 1H), 7.09 – 6.91 (m, 3H), 6.83 – 6.75 (m, 2H), 6.25 – 6.20 (m, 2H), 4.82 – 4.69 (m, 1H), 4.54 – 4.43 (m, 2H), 3.96 (s, 3H), 3.16 (d, *J* = 12.4 Hz, 1H), 2.61 (d, *J* = 12.5 Hz, 1H). ^{13}C NMR (75 MHz, $CDCl_3$) δ: 170.6, 153.5, 134.5, 133.7, 131.7, 129.6, 127.9, 127.3, 122.7, 121.9, 117.9, 115.3, 112.8, 77.3, 70.8, 53.2, 50.9, 46.7. UPLC-DAD-QTOF: $C_{23}H_{21}N_3O_4Br$ [M+H]+ calcd.: 482.0715, found: 482.0719.

(*S*)-3-Benzyl-3-((*S*)-1-(4-fluorophenyl)-2-nitroethyl)-1-methoxypyrrolo[1,2-*a*]pyrazin-4(3*H*)-one (3ac)

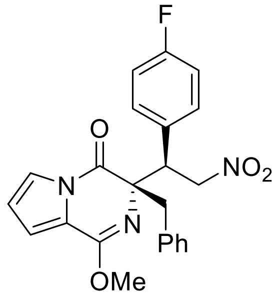

The title compound was prepared starting from (*S*)-3-benzyl-1-methoxypyrrolo[1,2-*a*]pyrazin-4(3*H*)-one **1a** according to the general procedure at –30 °C. The crude material was purified by flash column chromatography eluting 1:1 Hexane:CH_2Cl_2. Colourless solid. Yield: 38 mg, 0.09 mmol, 90%. m.p. 47-50 °C. $[\alpha]_D^{21}$= +36.2 (c=1, CH_2Cl_2). *dr* 97:3, 88% *ee*. ^{1}H NMR (300 MHz, $CDCl_3$) δ: 7.56 (ddd, *J* = 8.5, 5.2, 2.6 Hz, 2H), 7.32 (t, *J* = 2.4 Hz, 1H), 7.09 – 6.95 (m, 5H), 6.83 – 6.77 (m, 2H), 6.21 (d, *J* = 2.3 Hz, 2H), 4.85 – 4.70 (m, 1H), 4.55 – 4.45 (m, 2H), 3.97 (s, 3H), 3.18 (d, *J* = 12.5 Hz, 1H), 2.62 (d, *J* = 12.5 Hz, 1H). ^{13}C NMR (75 MHz, $CDCl_3$) δ: 170.7, 164.5, 161.2, 153.4, 133.9, 131.7, 131.6, 129.7, 127.9, 127.2, 121.9, 117.8, 115.6, 115.4, 115.3, 112.7, 77.5, 70.9, 53.2, 50.8, 46.6. UPLC-DAD-QTOF: $C_{23}H_{21}N_3O_4F$ [M+H]+ calcd.: 422.1516, found: 422.1513.

(*S*)-3-Benzyl-3-((*S*)-1-(2-chlorophenyl)-2-nitroethyl)-1-methoxypyrrolo[1,2-*a*]pyrazin-4(3*H*)-one (3ad)

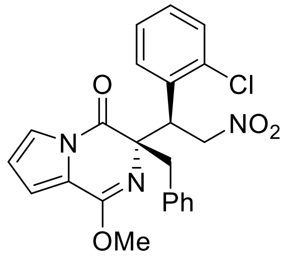

The title compound was prepared starting from (*S*)-3-benzyl-1-methoxypyrrolo[1,2-*a*]pyrazin-4(3*H*)-one **1a** according to the general procedure at –30 °C. The crude material was purified by flash column chromatography eluting 1:1 Hexane: CH_2Cl_2. Colourless solid. Yield: 37 mg, 0.084 mmol, 84%. m.p.= 156–159

°C. $[\alpha]_D^{21}$= +54.7 (c=1, CH_2Cl_2). *dr* >98:2, 86% *ee*. ^{1}H NMR (300 MHz, $CDCl_3$) δ: 7.87 – 7.75 (m, 1H), 7.50 – 7.44 (m, 1H), 7.39 – 7.31 (m, 1H), 7.28 (ddd, *J* = 6.5, 3.7, 2.1 Hz, 2H), 7.01 – 6.96 (m, 3H), 6.80 (dd, *J* = 7.2, 2.3 Hz, 2H), 6.22 (dd, *J* = 2.5, 0.7 Hz, 2H), 5.42 (dd, J = 10.7, 4.6 Hz, 1H), 4.76 (dd, *J* = 13.1, 10.9 Hz, 1H), 4.54 (ddd, *J* = 13.1, 4.6, 0.7 Hz, 1H), 3.97 (s, 3H), 3.40 (d, *J* = 12.5 Hz, 1H), 2.61 (d, *J* = 12.5 Hz, 1H). ^{13}C NMR (75 MHz, $CDCl_3$) δ: 170.5, 153.4, 136.7, 133.8, 133.6, 130.1, 129.8, 129.7, 129.4, 127.8, 127.2, 126.8, 121.9, 117.9, 115.2, 112.6, 77.1, 71.3, 53.2, 45.5, 45.4. UPLC-DAD-QTOF: $C_{24}H_{24}N_3O_5$ [M+H]+ calcd.: 434.1716, found: 434.1718.

(*S*)-3-Benzyl-3-((*S*)-1-(2,4-dibromo-5-methoxyphenyl)-2-nitroethyl)-1-methoxypyrrolo[1,2-*a*]pyrazin-4(3*H*)-one (3ae)

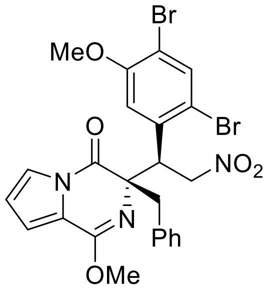

The title compound was prepared starting from (*S*)-3-benzyl-1-methoxypyrrolo[1,2-*a*]pyrazin-4(3*H*)-one **1a** according to the general procedure at –30 ºC. The crude material was purified by flash column chromatography eluting 1:1 Hexane:CH_2Cl_2. Colourless solid. Yield: 51 mg, 0.086 mmol, 86%. m.p. 200-203 °C. $[\alpha]_D^{21}$= +47.8(c=1, CH_2Cl_2). *dr* >98:2, 84% *ee*. ^{1}H NMR (300 MHz, $CDCl_3$) δ: 7.82 (s, 1H), 7.45 (d, *J* = 2.6 Hz, 2H), 7.39 – 7.31 (m, 1H), 7.04 – 6.94 (m, 3H), 6.79 (dd, *J* = 7.3, 2.3 Hz, 2H), 6.23 (d, *J* = 2.4 Hz, 2H), 5.34 (dd, *J* = 10.6, 4.7 Hz, 1H), 4.72 (dd, *J* = 13.0, 10.6 Hz, 1H), 4.53 (dd, *J* = 13.0, 4.7 Hz, 1H), 3.97 (s, 3H), 3.89 (s, 3H), 3.37 (d, *J* = 12.5 Hz, 1H), 2.63 (d, *J* = 12.5 Hz, 1H). ^{13}C NMR (75 MHz, $CDCl_3$) δ: 170.2, 155.4, 153.6, 136.8, 135.7, 133.4, 129.7, 127.8, 127.3, 121.8, 118.4, 118.0, 115.4, 113.0, 112.7, 77.2, 71.3, 56.5, 53.1, 48.2, 45.6. UPLC-DAD-QTOF: $C_{24}H_{22}N_3O_5Br_2$ [M+H]+ calcd.: 589.9926, found: 589.9927.

(*S*)-3-Benzyl-3-((*S*)-1-(furan-2-yl)-2-nitroethyl)-1-methoxypyrrolo[1,2-*a*]pyrazin-4(3*H*)-one (3af)

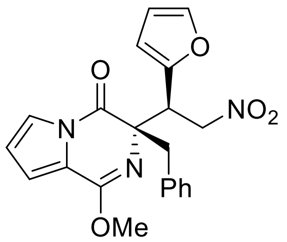

The title compound was prepared starting from (*S*)-3-benzyl-1-methoxypyrrolo[1,2-*a*]pyrazin-4(3*H*)-one **1a** according to the general procedure at –30 ºC. The crude material was purified by flash column chromatography eluting 1:1 Hexane:CH_2Cl_2. Orange solid. Yield: 35 mg, 0.89 mmol, 89%. m.p.= 99–102 °C. $[\alpha]_D^{21}$= +31.5 (c=1, CH_2Cl_2). *dr* >98:2, 98% *ee*. ^{1}H NMR (300 MHz, $CDCl_3$) δ: 7.32 (td, *J* = 1.9, 1.0 Hz, 2H), 7.05 – 7.00 (m, 3H), 6.92 – 6.86 (m, 2H), 6.40 (dd, *J* = 3.3, 0.8 Hz, 1H), 6.33 (dd, *J* = 3.3, 1.9 Hz, 1H), 6.24 – 6.21 (m, 2H), 4.83 (dd, *J* = 12.2, 9.9 Hz, 1H), 4.65 – 4.53 (m, 2H), 3.91 (s, 3H), 3.37 (d, *J* = 12.5 Hz, 1H), 2.85 (d, *J* = 12.5 Hz, 1H). ^{13}C NMR (75 MHz, $CDCl_3$) δ: 170.2, 153.2, 149.8, 142.6, 134.0, 129.9, 127.9, 127.2, 122.1, 117.8,

115.2, 112.5, 110.6, 109.9, 75.6, 70.6, 53.1, 46.1, 45.8. UPLC-DAD-QTOF: $C_{21}H_{20}N_3O_5$ [M+H]+ calcd.: 394.1403, found: 394.1403.

(*S*)-3-Benzyl-3-((*S*)-1-(furan-3-yl)-2-nitroethyl)-1-methoxypyrrolo[1,2-*a*]pyrazin-4(3*H*)-one (3ag)

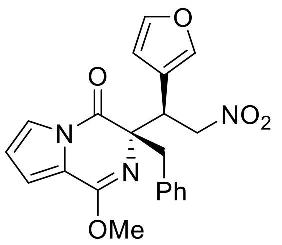

The title compound was prepared starting from (*S*)-3-benzyl-1-methoxypyrrolo[1,2-*a*]pyrazin-4(3*H*)-one **1a** according to the general procedure at –30 ºC. The crude material was purified by flash column chromatography eluting 1:1 Hexane:CH_2Cl_2. Orange solid. Yield: 33 mg, 0.085 mmol, 85%. m.p.= 124–127 °C. $[\alpha]_D^{21}$= +48.1 (c=1, CH_2Cl_2). *dr* >98:2, 91% *ee*. ^{1}H NMR (300 MHz, $CDCl_3$) δ: 7.58 (dd, *J* = 1.6, 0.8 Hz, 1H), 7.42 (t, *J* = 1.7 Hz, 1H), 7.32 (dd, *J* = 3.0, 1.5 Hz, 1H), 7.04 – 6.98 (m, 3H), 6.83 (dd, *J* = 6.3, 2.7 Hz, 2H), 6.68 (dd, *J* = 1.8, 0.9 Hz, 1H), 6.28 – 6.17 (m, 2H), 4.62 – 4.32 (m, 3H), 3.97 (s, 3H), 3.16 (d, *J* = 12.7 Hz, 1H), 2.89 (d, J = 12.6 Hz, 1H). ^{13}C NMR (75 MHz, $CDCl_3$) δ: 170.7, 153.6, 143.3, 142.3, 134.0, 129.7, 127.8, 127.2, 122.0, 119.6, 117.8, 115.3, 112.6, 110.8, 77.8, 70.7, 53.2, 46.5, 43.1. UPLC-DAD-QTOF: $C_{21}H_{20}N_3O_5$ [M+H]+ calcd.: 394.1403, found: 394.1405.

(*S*)-3-Benzyl-1-methoxy-3-((*S*)-2-nitro-1-(thiophen-2-yl)ethyl)pyrrolo[1,2-*a*]pyrazin-4(3*H*)-one (3ah)

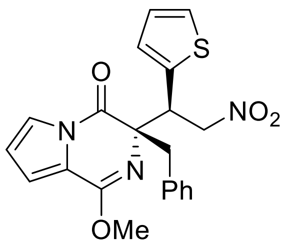

The title compound was prepared starting from (*S*)-3-benzyl-1-methoxypyrrolo[1,2-*a*]pyrazin-4(3*H*)-one **1a** according to the general procedure at –30 ºC. The crude material was purified by flash column chromatography eluting 1:1 Hexane:CH_2Cl_2. Orange solid. Yield: 35 mg, 0.086 mmol, 86%. m.p. 49-53 °C. $[\alpha]_D^{21}$= +41.6 (c=1, CH_2Cl_2). *dr* >98:2, 89% *ee*. ^{1}H NMR (300 MHz, $CDCl_3$) δ: 7.39 – 7.29 (m, 2H), 7.18 (dd, *J* = 3.4, 1.4 Hz, 1H), 7.04 – 6.97 (m, 4H), 6.82 (dq, *J* = 4.4, 1.9 Hz, 2H), 6.24 (td, *J* = 3.3, 1.7 Hz, 2H), 4.89 (dd, *J* = 10.4, 4.1 Hz, 1H), 4.56 (ddd, *J* = 12.1, 10.6, 1.5 Hz, 1H), 4.37 (ddd, *J* = 12.2, 4.2, 1.6 Hz, 1H), 4.06 (s, 3H), 3.17 (dd, *J* = 12.6, 1.6 Hz, 1H), 2.88 (dd, *J* = 12.6, 1.6 Hz, 1H). ^{13}C NMR (75 MHz, $CDCl_3$) δ: 170.5, 153.8, 137.0, 133.8, 129.8, 129.6, 127.8, 127.3, 127.2, 126.3, 122.0, 117.9, 115.3, 112.8, 79.2, 70.6, 53.9, 47.9, 46.5. UPLC-DAD-QTOF: $C_{21}H_{20}N_3O_4S$ [M+H]+ calcd.: 410.1175, found: 410.1173.

(*S*)-3-Benzyl-3-((*S*)-1-cyclohexyl-2-nitroethyl)-1-methoxypyrrolo[1,2-*a*]pyrazin-4(3*H*)-one (3ai)

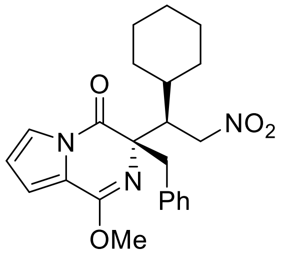

The title compound was prepared starting from (*S*)-3-benzyl-1-methoxypyrrolo[1,2-*a*]pyrazin-4(3*H*)-one **1a** according to the general procedure at –20 ºC. The crude material was purified by flash column chromatography eluting 1:1 Hexane:CH_2Cl_2. Colorless oil. Yield: 35 mg, 0.085 mmol, 85%. $[\alpha]_D^{21}$= +11.9 (c=1, CH_2Cl_2). *dr* >98:2, 93% *ee*. ^{1}H NMR (300 MHz, $CDCl_3$) δ: 7.27 (dd, *J* = 3.3, 1.6 Hz, 1H), 7.02 (q, *J* = 3.0 Hz, 3H), 6.95 – 6.86 (m, 2H), 6.28 – 6.16 (m, 2H), 4.78 (dd, *J* = 13.9, 5.0 Hz, 1H), 4.59 (ddd, *J* = 14.0, 6.3, 2.0 Hz, 1H), 3.89 (s, 3H), 3.38 (d, *J* = 12.2 Hz, 1H), 3.16 (d, *J* = 12.3 Hz, 1H), 1.94 (ddd, *J* = 14.5, 7.1, 2.9 Hz, 1H), 1.81 – 1.60 (m, 5H), 1.32 – 0.95 (m, 6H). ^{13}C NMR (75 MHz, $CDCl_3$) δ: 171.4, 152.9, 134.2, 129.9, 127.8, 127.2, 122.1, 117.6, 115.0, 112.2, 74.5, 70.9, 53.1, 51.2, 46.0, 38.1, 33.0, 28.4, 27.1, 26.7, 26.2. UPLC-DAD-QTOF: $C_{23}H_{28}N_3O_4$ [M+H]+ calcd.: 410.2080, found: 410.2074.

(*S*)-3-Isobutyl-1-methoxy-3-((*S*)-2-nitro-1-phenylethyl)pyrrolo[1,2-*a*]pyrazin-4(3*H*)-one (3ba)

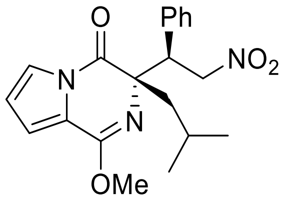

The title compound was prepared starting from (*S*)-3-isobutyl-1-methoxypyrrolo[1,2-*a*]pyrazin-4(3*H*)-one **1b** according to the general procedure at –20 ºC. The crude material was purified by flash column chromatography eluting 1:1 Hexane:CH_2Cl_2. Colorless solid. Yield: 31 mg, 0.084 mmol, 84%. *dr* 96:4, 89% *ee* (determined in the corresponding pyrrolopyrazinones **4ba**) . ^{1}H NMR (300 MHz, $CDCl_3$) δ: 7.46 (dq, *J* = 3.1, 1.6 Hz, 1H), 7.20 – 7.15 (m, 5H), 6.45 – 6.35 (m, 2H), 4.93 (dd, *J* = 12.9, 11.3 Hz, 1H), 4.83 – 4.71 (m, 1H), 4.06 (dt, *J* = 11.0, 3.6 Hz, 1H), 3.89 (s, 3H), 2.09 (dd, *J* = 13.2, 7.7 Hz, 1H), 1.76 (dd, *J* = 13.1, 4.7 Hz, 1H), 1.60 – 1.41 (m, 1H), 0.81 (d, *J* = 6.5 Hz, 3H), 0.59 (d, *J* = 6.6 Hz, 3H). ^{13}C NMR (75 MHz, $CDCl_3$) δ: 171.6, 152.5, 134.7, 129.2, 128.3, 128.1, 122.1, 117.9, 115.4, 112.7, 76.5, 69.8, 53.9, 53.1, 48.0, 24.9, 24.4, 22.8. The complete characterization of the adduct was made for the final pyrrolopyrazinones **4ba** (see section 2.4.1.).

(*S*)-3-((*S*)-1-(4-Bromophenyl)-2-nitroethyl)-3-isobutyl-1-methoxypyrrolo[1,2-*a*]pyrazin-4(3*H*)-one (3bb)

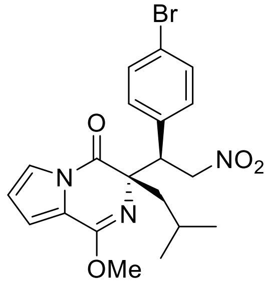

The title compound was prepared starting from (*S*)-3-isobutyl-1-methoxypyrrolo[1,2-*a*]pyrazin-4(3*H*)-one **1b** according to the general procedure at –20 ºC. The crude material was purified by flash column chromatography eluting 1:1 Hexane:CH_2Cl_2. Orange oil. Yield: 37 mg, 0.082 mmol, 82%. $[\alpha]_D^{21}$= -71.5 (c=1, CH_2Cl_2). *dr* 96:4, 89% *ee*. ^{1}H NMR (300 MHz, $CDCl_3$) δ: 7.47 (dt, *J* = 3.2, 0.7 Hz, 1H), 6.49 (dq, *J* = 3.4, 0.9 Hz, 1H), 6.42 (td, *J* = 3.3, 0.8 Hz, 1H), 4.84 (dd, *J* = 13.1, 11.4 Hz, 1H), 4.67 (dd, *J* = 13.1, 4.2 Hz, 1H), 4.05 (dd, *J* = 11.3, 4.3 Hz, 1H), 3.89 (s, 3H), 2.03 (dd, *J* = 13.2, 7.4 Hz, 1H), 1.68 (dd, *J* = 13.1, 5.0 Hz, 1H), 1.45 (ddd, *J* = 13.2, 9.3, 6.0 Hz, 1H), 0.78 (d, *J* = 6.7 Hz, 3H), 0.59 (d, *J* = 6.7 Hz, 3H). ^{13}C NMR (75 MHz, $CDCl_3$) δ: 171.8, 153.1, 134.4, 131.8, 131.4, 122.9, 122.4, 118.5, 116.1, 113.6, 78.0, 70.1, 53.6, 53.6, 48.6, 25.2, 24.8, 23.3. UPLC-DAD-QTOF: $C_{20}H_{23}N_3O_4Br$ [M+H]+ calcd.: 448.0872, found: 448.0873.

(*S*)-3-((*S*)-1-(Furan-2-yl)-2-nitroethyl)-3-isobutyl-1-methoxypyrrolo[1,2-*a*]pyrazin-4(3*H*)-one (3bf)

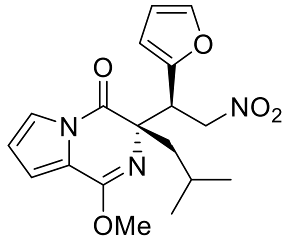

The title compound was prepared starting from (*S*)-3-isobutyl-1-methoxypyrrolo[1,2-*a*]pyrazin-4(3*H*)-one **1b** according to the general procedure at –30 ºC. The crude material was purified by flash column chromatography eluting 1:1 Hexane: CH_2Cl_2. Orange solid. Yield: 31 mg, 0.087 mmol, 87%. m.p.= 104–108 °C. $[\alpha]_D^{21}$= -80.3 (c=1, CH_2Cl_2). *dr* >98:2, 72% *ee*. ^{1}H NMR (300 MHz, $CDCl_3$) δ: 7.46 (dt, *J* = 3.2, 1.3 Hz, 1H), 7.10 (dt, *J* = 1.8, 0.9 Hz, 1H), 6.49 (dt, *J* = 3.4, 1.2 Hz, 1H), 6.40 (td, *J* = 3.3, 0.9 Hz, 1H), 6.19 – 6.13 (m, 1H), 6.13 – 6.10 (m, 1H), 4.97 – 4.77 (m, 2H), 4.11 (dd, *J* = 10.7, 4.1 Hz, 1H), 3.86 (s, 3H), 2.21 – 2.10 (m, 1H), 1.90 – 1.81 (m, 1H), 1.66 – 1.48 (m, 1H), 0.86 (dd, *J* = 6.7, 0.9 Hz, 3H), 0.62 (dd, *J* = 6.6, 0.9 Hz, 3H). ^{13}C NMR (75 MHz, $CDCl_3$) δ: 171.0, 152.7, 149.0, 142.5, 122.1, 118.2, 115.2, 112.7, 110.4, 109.9, 74.8, 69.0. UPLC-DAD-QTOF: $C_{18}H_{22}N_3O_5$ [M+H]+ calcd.: 360.1559, found: 360.1560.

(*S*)-1-Methoxy-3-(4-methoxybenzyl)-3-((*S*)-2-nitro-1-phenylethyl)pyrrolo[1,2-*a*]pyrazin-4(3*H*)-one (3ca)

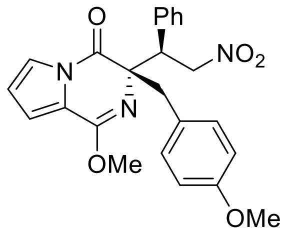

The title compound was prepared starting from (*S*)-1-methoxy-3-(4-methoxybenzyl)pyrrolo[1,2-*a*]pyrazin-4(3*H*)-one **1c** according to the general procedure at –20 ºC. The crude material was purified by flash column chromatography eluting 1:1 Hexane: CH_2Cl_2. Colorless oil. Yield: 38 mg, 0.088 mmol, 88%.

$[\alpha]_D^{21}$= +46.1 (c=1, CH_2Cl_2). *dr* >98:2, 84% *ee*. ^{1}H NMR (300 MHz, $CDCl_3$) δ: 7.56 – 7.52 (m, 2H), 6.79 – 6.67 (m, 2H), 6.58 – 6.46 (m, 2H), 6.27 – 6.17 (m, 2H), 4.82 (dd, *J* = 12.6, 10.7 Hz, 1H), 4.55 (dd, *J* = 12.7, 4.5 Hz, 1H), 4.46 (dd, *J* = 10.7, 4.5 Hz, 1H), 3.97 (s, 3H), 3.64 (s, 3H), 3.16 (d, *J* = 12.7 Hz, 1H), 2.60 (d, *J* = 12.7 Hz, 1H). ^{13}C NMR (75 MHz, $CDCl_3$) δ: 171.0, 158.8, 153.2, 135.4, 130.7, 130.0, 128.5, 128.4, 126.1, 122.0, 117.8, 115.2, 113.3, 112.5, 77.5, 71.1, 55.2, 53.2, 51.5, 45.8. UPLC-DAD-QTOF: $C_{24}H_{24}N_3O_5$ [M+H]+ calcd.: 434.1716, found: 434.1718.

(*S*)-3-((*S*)-1-(Furan-2-yl)-2-nitroethyl)-1-methoxy-3-(4-methoxybenzyl)pyrrolo[1,2-*a*]pyrazin-4(3*H*)-one (3cf)

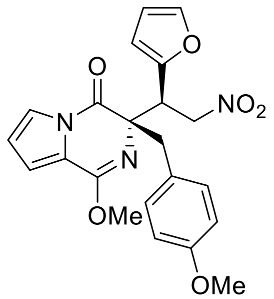

The title compound was prepared starting from (*S*)-1-methoxy-3-(4-methoxybenzyl)pyrrolo[1,2-*a*]pyrazin-4(3*H*)-one **1Cc** according to the general procedure at –20 ºC. The crude material was purified by flash column chromatography eluting 1:1 Hexane:CH_2Cl_2. Pale yellow oil. Yield: 39 mg, 0.090 mmol, 90%. $[\alpha]_D^{21}$= +47.5 (c=1, CH_2Cl_2). *dr* >98:2, 79% *ee*. ^{1}H NMR (300 MHz, $CDCl_3$) δ: 7.33 (dd, *J* = 3.1, 1.6 Hz, 1H), 7.31 (dd, *J* = 1.9, 0.7 Hz, 1H), 6.82 (dd, *J* = 9.0, 2.3 Hz, 2H), 6.59 – 6.54 (m, 2H), 6.38 (d, *J* = 3.1 Hz, 1H), 6.32 (dd, *J* = 3.3, 1.8 Hz, 1H), 6.27 – 6.22 (m, 2H), 4.82 (dd, *J* = 12.9, 10.7 Hz, 1H), 4.61 (dd, *J* = 13.0, 4.2 Hz, 1H), 4.53 (dd, *J* = 10.7, 4.2 Hz, 1H), 3.90 (s, 3H), 3.66 (s, 3H), 3.32 (d, *J* = 12.7 Hz, 1H), 2.80 (d, *J* = 12.7 Hz, 1H). ^{13}C NMR (75 MHz, $CDCl_3$) δ: 170.3, 158.8, 153.2, 149.8, 142.6, 130.9, 126.1, 122.1, 117.8, 115.2, 113.4, 112.5, 110.6, 109.8, 75.6, 70.6, 55.2, 53.1, 46.0, 44.9. UPLC-DAD-QTOF: $C_{22}H_{22}N_3O_6$ [M+H]+ calcd.: 424.1503, found: 424.1511.

(*S*)-1-Methoxy-3-(4-methoxybenzyl)-3-((*S*)-2-nitro-1-(thiophen-2-yl)ethyl)pyrrolo[1,2-*a*]pyrazin-4(3*H*)-one (3ch)

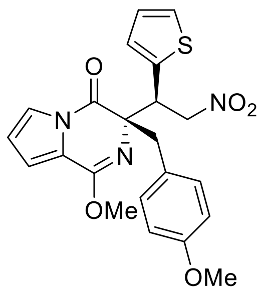

The title compound was prepared starting from (*S*)-1-methoxy-3-(4-methoxybenzyl)pyrrolo[1,2-*a*]pyrazin-4(3*H*)-one **1c** according to the general procedure at –20 ºC. The crude material was purified by flash column chromatography eluting 1:1 Hexane:CH_2Cl_2. Colourless solid. Yield: 39 mg, 0.088 mmol, 88%. m.p. 134-137 °C. $[\alpha]_D^{21}$= +42.2 (c=1, CH_2Cl_2). *dr* >98:2, 77% *ee*. ^{1}H NMR (300 MHz, $CDCl_3$) δ: 7.34 (dt, *J* = 4.1, 1.0 Hz, 2H), 7.17 (dd, *J* = 3.5, 1.3 Hz, 1H), 6.99 (dd, *J* = 5.2, 3.5 Hz, 1H), 6.74 (dd, *J* = 9.2, 2.6 Hz, 2H), 6.53 (dt, *J* = 9.3, 2.7 Hz, 2H), 6.28 (dd, *J* = 3.4, 1.5 Hz, 1H), 6.24 (t, *J* = 3.3 Hz, 1H), 4.86 (dd, *J* = 10.5, 4.2 Hz, 1H), 4.55 (dd, *J* = 12.2, 10.5 Hz, 1H), 4.36 (dd, *J* = 12.2, 4.2 Hz, 1H), 4.05 (s, 3H), 3.65 (s, 3H), 3.12 (d, *J* = 12.9 Hz, 1H), 2.82 (d, *J* = 12.8 Hz, 1H). ^{13}C NMR (75 MHz, $CDCl_3$) δ: 170.6, 158.9, 153.7,

137.1, 130.6, 129.7, 127.2, 126.2, 125.9, 122.1, 117.9, 115.3, 113.3, 112.8, 79.2, 70.7, 55.3, 53.9, 47.8, 45.7. UPLC-DAD-QTOF: $C_{22}H_{22}N_3O_5S$ [M+H]+ calcd.: 440.1275, found: 440.1284.

(*S*)-1-Methoxy-3-((*S*)-2-nitro-1-phenylethyl)-3-phenethylpyrrolo[1,2-*a*]pyrazin-4(3*H*)-one (3da)

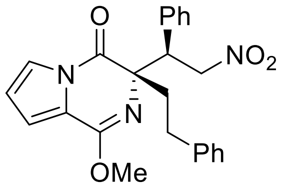

The title compound was prepared starting from 1-methoxy-3-phenethylpyrrolo[1,2-*a*]pyrazin-4(3*H*)-one **1d** according to the general procedure at –30 ºC. The crude material was purified by flash column chromatography eluting 1:1 Hexane:CH_2Cl_2. Yield: 35 mg, 0.084 mmol, 84%. m.p. 46-50 °C. $[\alpha]_D^{21}$= -10.6 (c=1, CH_2Cl_2). *dr* 94:6, 84% *ee*. ^{1}H NMR (300 MHz, $CDCl_3$) δ: 7.46 (dd, *J* = 3.2, 1.5 Hz, 1H), 7.33 – 7.28 (m, 2H), 7.24 – 7.10 (m, 6H), 7.01 – 6.91 (m, 2H), 6.52 (dd, *J* = 3.4, 1.5 Hz, 1H), 6.42 (t, *J* = 3.3 Hz, 1H), 4.86 (dd, *J* = 12.9, 10.9 Hz, 1H), 4.66 (dd, *J* = 12.9, 4.5 Hz, 1H), 4.20 (dd, *J* = 10.9, 4.5 Hz, 1H), 3.93 (s, 3H), 2.45 – 2.16 (m, 4H), 2.01 – 1.87 (m, 1H). ^{13}C NMR (75 MHz, $CDCl_3$) δ: 171.1, 153.1, 140.4, 134.9, 129.5, 128.5, 128.4, 128.4, 128.3, 122.2, 118.1, 115.5, 113.1, 76.9, 70.0, 53.3, 52.4, 41.5, 30.4. UPLC-DAD-QTOF: $C_{24}H_{24}N_3O_4$ [M+H]+ calcd.: 418.1767, found: 418.1763.

(*S*)-3-((*S*)-1-(Furan-3-yl)-2-nitroethyl)-1-methoxy-3-phenethylpyrrolo[1,2-*a*]pyrazin-4(3*H*)-one (3dg)

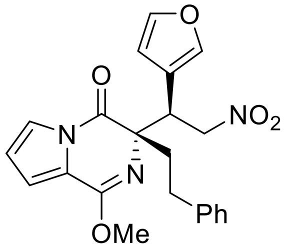

The title compound was prepared starting from 1-methoxy-3-phenethylpyrrolo[1,2-*a*]pyrazin-4(3*H*)-one **1d** according to the general procedure at –30 ºC. The crude material was purified by flash column chromatography eluting 1:1 Hexane: CH_2Cl_2. Orange oil. Yield: 35 mg, 0.085 mmol, 85%. $[\alpha]_D^{21}$= -5.56 (c=1, CH_2Cl_2). *dr* 97:3, 92% *ee*. ^{1}H NMR (300 MHz, $CDCl_3$) δ: 7.48 (dd, *J* = 3.2, 1.4 Hz, 1H), 7.42 – 7.39 (m, 1H), 7.30 (t, *J* = 1.7 Hz, 1H), 7.17 (tdd, *J* = 8.4, 6.3, 4.8 Hz, 3H), 7.01 – 6.94 (m, 2H), 6.63 (dd, *J* = 3.4, 1.4 Hz, 1H), 6.49 – 6.43 (m, 2H), 4.58 (dd, *J* = 12.4, 10.9 Hz, 1H), 4.45 (dd, *J* = 12.4, 4.3 Hz, 1H), 4.21 (dd, *J* = 10.8, 4.2 Hz, 1H), 3.95 (s, 3H), 2.45 – 2.18 (m, 4H), 2.03 (td, *J* = 12.1, 7.4 Hz, 1H). ^{13}C NMR (75 MHz, $CDCl_3$) δ: 171.0, 153.4, 143.2, 142.0, 140.3, 128.5, 128.4, 126.3, 122.2, 119.2, 118.3, 115.6, 113.4, 110.3, 77.3, 69.6, 53.3, 43.8, 41.6, 30.2. UPLC-DAD-QTOF: $C_{22}H_{22}N_3O_5$ [M+H]+ calcd.: 408.1559, found: 408.1558.

(*S*)-3-((*S*)-1-Cyclohexyl-2-nitroethyl)-1-methoxy-3-phenethylpyrrolo[1,2-*a*]pyrazin-4(3*H*)-one (3di)

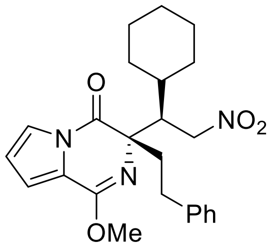

The title compound was prepared starting from 1-methoxy-3-phenethylpyrrolo[1,2-*a*]pyrazin-4(3*H*)-one **1d** according to the general procedure at –30 ^{o}C. The crude material was purified by flash column chromatography eluting 1:1 Hexane:CH_2Cl_2. Yellow oil. Yield: 36 mg, 0.084 mmol, 84%. $[\alpha]_D^{21}$= +17.6 (c=1, CH_2Cl_2). *dr* >98:2, 93% *ee*. ^{1}H NMR (300 MHz, $CDCl_3$) δ: 7.46 (dd, *J* = 3.2, 1.5 Hz, 1H), 7.27 – 7.13 (m, 3H), 7.09 – 7.02 (m, 2H), 6.67 (dd, *J* = 3.4, 1.5 Hz, 1H), 6.48 (t, *J* = 3.3 Hz, 1H), 4.67 (dd, *J* = 13.9, 5.3 Hz, 1H), 4.49 (dd, *J* = 13.9, 6.2 Hz, 1H), 3.89 (s, 3H), 2.98 (td, *J* = 5.8, 2.1 Hz, 1H), 2.52 – 2.33 (m, 2H), 2.31 – 2.12 (m, 2H), 1.85 – 1.65 (m, 2H), 1.60 (q, *J* = 4.3 Hz, 4H), 1.23 – 0.81 (m, 5H), ^{13}C NMR (75 MHz, $CDCl_3$) δ: 171.7, 153.0, 140.7, 128.6, 128.5, 126.3, 122.3, 118.2, 115.5, 113.0, 74.0, 69.7, 53.2, 51.2, 41.5, 37.6, 33.2, 30.3, 28.3, 27.1, 26.6, 26.2. UPLC-DAD-QTOF: $C_{24}H_{30}N_3O_4$ [M+H]+ calcd.: 424.2236, found: 424.2231.

(*S*)-3-((1*H*-Indol-3-yl)methyl)-1-methoxy-3-((*S*)-2-nitro-1-phenylethyl)pyrrolo[1,2-*a*]pyrazin-4(3*H*)-one (3ea)

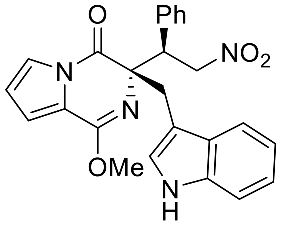

The title compound was prepared starting from (*S*)-3-((1*H*-indol-3-yl)methyl)-1-methoxypyrrolo[1,2-*a*]pyrazin-4(3*H*)-one **1e** according to the general procedure at –30 ^{o}C. The crude material was purified by flash column chromatography eluting 1:1 Hexane:CH_2Cl_2. Orange foam. Yield: 38 mg, 0.085 mmol, 85%. *dr* > 98:2, 48% *ee*. ^{1}H NMR (300 MHz, $CDCl_3$) δ: 7.78 (s, 1H), 7.65 – 7.59 (m, 2H), 7.45 – 7.31 (m, 4H), 7.19 (dd, *J* = 3.0, 1.5 Hz, 1H), 7.13 (dd, *J* = 7.3, 1.4 Hz, 1H), 7.10 – 6.96 (m, 2H), 6.66 (d, *J* = 2.5 Hz, 1H), 6.13 – 6.07 (m, 2H), 4.85 (dd, *J* = 13.6, 11.6 Hz, 1H), 4.57 (ddd, *J* = 11.7, 8.3, 4.5 Hz, 2H), 3.91 (s, 3H), 3.61 (dd, *J* = 7.6, 5.8 Hz, 1H), 3.42 (d, *J* = 13.5 Hz, 1H), 2.88 (d, *J* = 13.8 Hz, 1H). ^{13}C NMR (75 MHz, $CDCl_3$) δ: 171.6, 153.1, 135.7, 135.6, 128.5, 128.4, 122.9, 122.0, 119.3, 119.3, 117.7, 114.7, 112.0, 110.8, 108.9, 77.7, 71.3, 53.0, 51.3, 36.7. UPLC-DAD-QTOF: $C_{25}H_{23}N_4O_4$ [M+H]+ calcd.: 443.1719, found: 443.1714.

(*S*)-3-Allyl-1-methoxy-3-((*S*)-2-nitro-1-phenylethyl)pyrrolo[1,2-*a*]pyrazin-4(3*H*)-one (3fa)

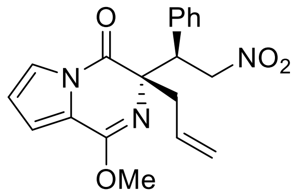

The title compound was prepared starting from 3-allyl-1-methoxypyrrolo[1,2-*a*]pyrazin-4(3*H*)-one **1f** according to the general procedure at –30 ºC. The crude material was purified by flash column chromatography eluting 1:1 Hexane:CH_2Cl_2. Colourless solid. Yield: 30 mg, 0.084 mmol, 84%, m.p.= 116–122 °C. $[\alpha]_D^{21}$= -17.9 (c=1, CH_2Cl_2), *dr* 96:4, 85% *ee.* ^{1}H NMR (300 MHz, $CDCl_3$) δ: 7.48 (dt, *J* = 3.1, 1.3 Hz, 1H), 7.45 – 7.39 (m, 2H), 7.27 (dd, *J* = 4.5, 2.4 Hz, 3H), 6.54 (dd, *J* = 3.4, 1.4 Hz, 1H), 6.43 (dd, *J* = 3.9, 2.7 Hz, 1H), 5.43 – 5.27 (m, 1H), 5.02 – 4.86 (m, 2H), 4.81 (dd, *J* = 12.8, 10.8 Hz, 1H), 4.56 (ddd, *J* = 12.8, 4.6, 1.2 Hz, 1H), 4.27 (dd, *J* = 10.7, 4.5 Hz, 1H), 3.94 (s, 3H), 2.64 (dd, *J* = 12.8, 8.1 Hz, 1H), 2.25 (ddt, *J* = 12.9, 6.8, 1.2 Hz, 1H). ^{13}C NMR (75 MHz, $CDCl_3$) δ: 170.8, 153.1, 135.1, 130.6, 129.8, 128.4, 122.2, 120.3, 118.1, 115.5, 113.0, 77.0, 70.1, 53.3, 51.4, 44.6. UPLC-DAD-QTOF: $C_{19}H_{20}N_3O_4$ [M+H]+ calcd.: 354.1454, found: 354.1461.

(*R*)-3-((*S*)-1-Cyclohexyl-2-nitroethyl)-1-methoxy-3-phenylpyrrolo[1,2-*a*]pyrazin-4(3*H*)-one (3gi)

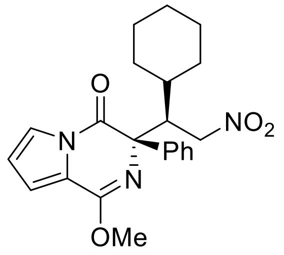

The title compound was prepared starting from (*S*)-1-methoxy-3-phenylpyrrolo[1,2-*a*]pyrazin-4(3*H*)-one **1g** according to the general procedure at –30 ºC. The crude material was purified by flash column chromatography eluting 1:1 Hexane:CH_2Cl_2. Colourless solid. Yield: 36 mg, 0.092 mmol, 92%. m.p.= 153–154 °C. $[\alpha]_D^{21}$= +17.6 (c=1, CH_2Cl_2). *dr* >98:2, 72% *ee*. ^{1}H NMR (300 MHz, $CDCl_3$) δ: 7.66 – 7.54 (m, 2H), 7.43 (dd, *J* = 3.2, 1.5 Hz, 1H), 7.39 – 7.25 (m, 3H), 6.65 (dd, *J* = 3.4, 1.4 Hz, 1H), 6.42 (t, *J* = 3.3 Hz, 1H), 4.54 (dd, *J* = 14.4, 7.7 Hz, 1H), 4.29 (dd, *J* = 14.4, 3.5 Hz, 1H), 4.04 (s, 3H), 3.76 (dt, *J* = 7.7, 3.3 Hz, 1H), 1.80 – 1.52 (m, 5H), 1.46 – 1.31 (m, 1H), 1.18 – 1.02 (m, 5H). ^{13}C NMR (75 MHz, $CDCl_3$) δ: 170.1, 153.3, 138.8, 129.0, 128.9, 126.8, 121.9, 119.2, 115.1, 113.1, 74.9, 73.4, 53.3, 51.0, 40.8, 33.6, 29.5, 27.1, 26.7, 26.1. UPLC-DAD-QTOF: $C_{22}H_{26}N_3O_4$ [M+H]+ calcd.: 396.1923, found: 396.1916.

(*S*)-3-Hexyl-1-methoxy-3-((*S*)-2-nitro-1-phenylethyl)pyrrolo[1,2-*a*]pyrazin-4(3*H*)-one (3ha)

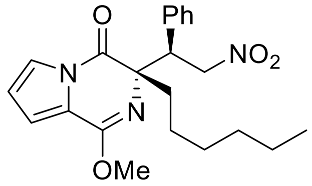

The title compound was prepared starting from **1h** according to the general procedure at –30 ºC. The crude material was purified by flash column chromatography eluting 1:1 Hexane:CH_2Cl_2. Colourless foam. Yield: 35 mg, 0.089 mmol,

89%. *dr* 92:8, 75% *ee* (determined in the corresponding pyrrolopyrazinones **4ha**). Data for **3ha**: ^{1}H NMR (300 MHz, $CDCl_3$) δ: 7.49 (dd, *J* = 3.2, 1.5 Hz, 1H), 7.35 – 7.29 (m, 2H), 7.25 – 7.21 (m, 2H), 7.07 (dd, *J* = 3.5, 2.5 Hz, 1H), 6.51 (dd, *J* = 3.4, 1.5 Hz, 1H), 6.42 (t, *J* = 3.3 Hz, 1H), 4.84 (dd, *J* = 12.9, 10.9 Hz, 1H), 4.62 (dd, *J* = 12.9, 4.5 Hz, 1H), 4.18 (dd, *J* = 10.8, 4.5 Hz, 1H), 3.92 (s, 3H), 2.01 (ddd, *J* = 12.7, 11.5, 4.6 Hz, 1H), 2.01 (ddd, *J* = 12.7, 11.5, 4.6 Hz, 1H), 1.63 – 1.46 (m, 1H), 1.31 – 1.05 (m, 8H), 0.79 (t, *J* = 6.9 Hz, 3H). The complete characterization of the adduct was made for the final pyrrolopyrazinone **4ha** (see section 2.4.1.).

2.4. Elaboration of adducts

2.4.1. Preparation of diketopyrrolo piperazines 4

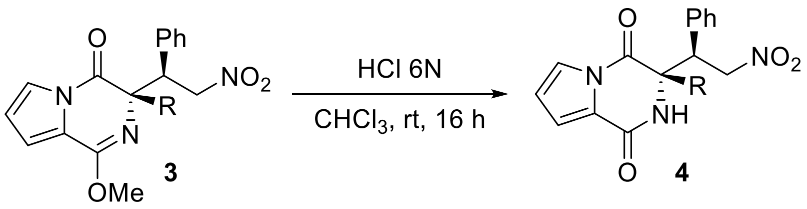

To a solution of the corresponding Michael adduct **3** (0.07-0.5 mmol) in $CHCl_3$ (1 mL) was added HCl 6N (1 mL) and the reaction mixture was stirred overnight at room temperature. Then, it was diluted with CH_2Cl_2 and water, extracted with CH_2Cl_2 (3 x 25 mL), dried over $MgSO_4$, filtrated and concentrated under vacuum to produce pure diketopyrrolo piperazines **4**.

(*S*)-3-Benzyl-3-((*S*)-2-nitro-1-phenylethyl)-2,3-dihydropyrrolo[1,2-*a*]pyrazine-1,4-dione (4aa)

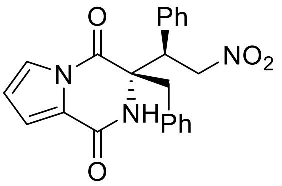

Colourless solid. Yield: 181 mg, 0.46 mmol, 93%. m.p.= 136-138 °C. $[\alpha]_D^{21}$= -9.31 (c=1, CH_2Cl_2). ^{1}H NMR (300 MHz, $CDCl_3$) δ: 7.57 (s, 1H), 7.41 (dd, *J* = 3.3, 1.6 Hz, 1H), 7.37 – 7.25 (m, 4H), 7.14 – 7.09 (m, 2H), 7.06 – 7.00 (m, 2H), 6.70 (dd, *J* = 3.6, 1.6 Hz, 1H), 6.33 (t, *J* = 3.3 Hz, 1H), 5.14 (dd, *J* = 13.3, 11.1 Hz, 1H), 4.89 (dd, *J* = 13.3, 4.3 Hz, 1H), 4.38 (dd, *J* = 11.0, 4.3 Hz, 1H), 3.49 (d, *J* = 13.2 Hz, 1H), 2.83 (d, *J* = 13.2 Hz, 1H). ^{13}C NMR (75 MHz, $CDCl_3$) δ: 165.7, 157.3, 133.0, 132.7, 130.1, 129.3, 129.3, 129.2, 128.5, 127.9, 124.6, 119.4, 118.9, 116.1, 75.8, 68.6, 52.3, 46.2. UPLC-DAD-QTOF: $C_{22}H_{20}N_3O_4$ [M+H]+ calcd.: 390.1454, found: 390.1452.

(*S*)-3-Isobutyl-3-((*S*)-2-nitro-1-phenylethyl)-2,3-dihydropyrrolo[1,2-*a*]pyrazine-1,4-dione (4ba)

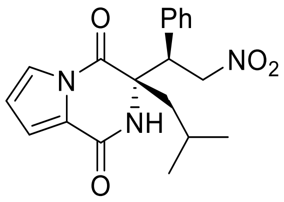

Colorless solid. Yield: 25 mg, 0.071 mmol, 71% (from **1b**). m.p.= 71-74 °C. $[\alpha]_D^{21}$= -62.3 (c=1, CH_2Cl_2). *dr* 96:4, 89% *ee*. ^{1}H NMR (300 MHz, $CDCl_3$) δ: 8.06 – 7.76 (m, 1H), 7.48 (dd, *J* = 3.2, 1.5 Hz, 1H), 7.26 – 7.05 (m, 5H), 6.86 (dd, *J* = 3.3, 1.3 Hz, 1H), 6.43 (t, *J* = 3.3 Hz, 1H), 5.09 (t, *J* = 12.5 Hz, 1H), 4.95 – 4.86 (m, 1H), 4.00 (dd, *J* = 11.1, 4.5 Hz, 1H), 2.31 – 2.22 (m, 1H), 1.81 – 1.63 (m, 2H), 0.87 (d, *J* = 6.5 Hz, 3H), 0.71 (d, *J* = 6.5 Hz, 3H). ^{13}C NMR (75 MHz, $CDCl_3$) δ: 166.1, 157.4, 132.9, 129.1, 128.8, 128.8, 124.7, 119.5, 118.9, 116.2, 75.2, 67.4, 54.9, 46.6, 29.8, 25.2, 24.0, 22.7. UPLC-DAD-QTOF: $C_{19}H_{22}N_3O_4$ [M+H]+ calcd.: 356.1610, found: 356.1602; $C_{19}H_{21}N_3O_4Na$ [M+Na]+ calcd.: 378.1430, found: 378.1423.

(*S*)-3-Allyl-3-((*S*)-2-nitro-1-phenylethyl)-2,3-dihydropyrrolo[1,2-*a*]pyrazine-1,4-dione (4fa)

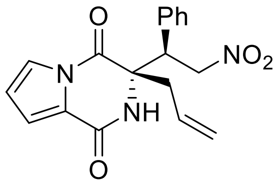

Colourless solid. Yield: 153 mg, 0.45 mmol, 90%. m.p.= 140-143 °C. $[\alpha]_D^{21}$= -7.53 (c=1, CH_2Cl_2). ^{1}H NMR (300 MHz, $CDCl_3$) δ: 7.55 (dd, *J* = 3.2, 1.5 Hz, 1H), 7.38 – 7.33 (m, 3H), 7.28 – 7.24 (m, 2H), 7.03 (dd, *J* = 3.5, 1.5 Hz, 1H), 6.53 (t, *J* = 3.4 Hz, 1H), 6.11 (s, 1H), 5.57 (dddd, *J* = 16.9, 10.1, 7.7, 6.9 Hz, 1H), 5.23 – 5.12 (m, 2H), 5.03 (dd, *J* = 13.3, 11.0 Hz, 1H), 4.74 (dd, *J* = 13.3, 4.5 Hz, 1H), 4.20 (dd, *J* = 10.9, 4.5 Hz, 1H), 2.89 (dd, *J* = 13.7, 7.7 Hz, 1H), 2.32 (dd, *J* = 13.7, 6.9 Hz, 1H). ^{13}C NMR (75 MHz, $CDCl_3$) δ: 165.5, 157.2, 132.8, 129.3, 129.2, 129.1, 128.9, 124.8, 122.6, 119.7, 119.2, 116.3, 75.3, 67.4, 52.4, 43.6. UPLC-DAD-QTOF: $C_{22}H_{20}N_3O_4$ [M+H]+ calcd.: 340.1292, found: 340.1290.

***(S*)-3-Hexyl-3-((*S*)-2-nitro-1-phenylethyl)-2,3-dihydropyrrolo[1,2-*a*]pyrazine-1,4-dione (4ha)**

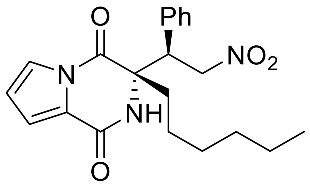

Colourless solid. Yield: 34 mg, 0.088 mmol, 88%. m.p.= 60-65 °C. $[\alpha]_D^{21}$= -37.6. (c=1, CH_2Cl_2). *dr* 92:8, 75% *ee*. ^{1}H NMR (300 MHz, $CDCl_3$) δ: 7.54 (dd, *J* = 3.2, 1.5 Hz, 1H), 7.49 (s, 1H), 7.35 – 7.17 (m, 5H), 6.95 (dd, *J* = 3.5, 1.5 Hz, 1H), 6.50 (t, *J* = 3.3 Hz, 1H), 5.07 (dd, *J* = 13.3, 10.9 Hz, 1H), 4.84 (dd, *J* = 13.5, 4.6 Hz, 1H), 4.11 (dd, *J* = 10.9, 4.5 Hz, 1H), 2.34 – 2.18 (m, 1H), 1.63 (td, *J* = 12.9, 12.5, 3.8 Hz, 1H), 1.36 – 1.04 (m, 8H), 0.80 (d, *J* = 7.1 Hz, 3H). ^{13}C NMR (75 MHz, $CDCl_3$) δ: 166.0, 157.3, 133.0, 129.5, 129.2, 129.1, 128.8, 127.2, 124.8, 119.6, 119.1, 116.3, 75.4, 67.9, 53.3, 39.2, 31.5, 29.0, 23.9, 22.5, 14.0. UPLC-DAD-QTOF: $C_{21}H_{26}N_3O_4$ [M+H]+ calcd.: 384.1923, found: 384.1923. $C_{21}H_{25}N_3O_4Na$ [M+Na]+ calcd.: 406.1743, found: 406.1738.

2.4.2. Preparation of primary amine 5 from 4aa

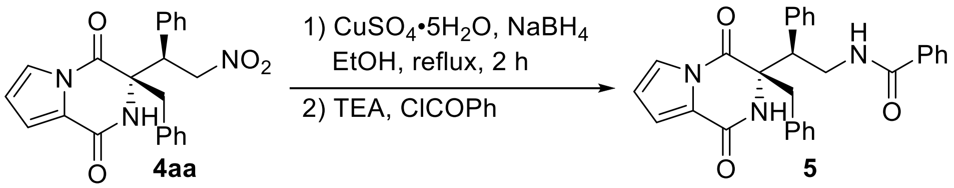

Step 1:[23] The diketopiperazine **4aa** (0.1 mmol) was dissolved in ethanol (0.5 mL) and a solution of $CuSO_4$ (0.5 mL, 2M aqueous solution, 10 mol %) was added. After the reaction mixture was cooled to 0 °C, $NaBH_4$ (0.5 mmol, 5 equiv.) was added portionwise and the reaction mixture was stirred at reflux. After 2 hours the reaction mixture was diluted with EtOAc and the organic layer washed with water, dried over $MgSO_4$, filtrated and evaporated under vacuum. The crude was used in the next step without any purification.

Step 2:[24] To a solution of the resulting amine (0.1 mmol) and Et_3N (0.11 mmol, 1.1 equiv.) in CH_2Cl_2 (1 mL) was added benzoyl chloride (1.1 equiv.) dropwise and the reaction was left stirring overnight. The reaction was quenched with water and extracted with CH_2Cl_2, dried over $MgSO_4$, filtrated and evaporated under vacuum. The crude product was purified by flash column chromatography (eluting with Hexane/EtOAc 95/5 to 70/30) to afford an orange foam. $[\alpha]_D^{21}$= -21.76 (c=1, CH_2Cl_2). Yield: 19.5 mg, 0.042 mmol, 42% (2 steps).

***N*-((*S*)-2-((*S*)-3-benzyl-1,4-dioxo-1,2,3,4-tetrahydropyrrolo[1,2-*a*]pyrazin-3-yl)-2-phenylethyl)benzamide (5).** ^{1}H NMR (300 MHz, $CDCl_3$) δ: 9.68 (s, 1H), 8.07 – 7.99 (m, 3H), 7.61 – 7.49 (m, 1H), 7.38 (td, *J* = 7.4, 4.7 Hz, 2H), 7.32 – 7.18 (m, 7H), 7.15 – 7.03 (m, 2H), 6.76 (td, *J* = 2.7, 1.2 Hz, 1H), 6.17 (dq, *J* = 2.7, 1.5 Hz, 1H), 5.97 (q, *J* = 2.7 Hz, 1H), 5.90 (s, 1H), 3.95 (dd, *J* = 7.2, 4.2 Hz, 1H), 3.78 (dd, *J* = 8.1, 4.3 Hz, 1H), 3.72 (d, *J* = 7.6 Hz, 1H), 3.34 (d, *J* = 13.4 Hz, 1H). ^{13}C NMR (75 MHz, $CDCl_3$) δ: 168.8, 163.9, 161.0, 138.1, 134.7, 134.5, 133.5, 131.0, 130.5, 130.2, 128.8, 128.7, 128.6, 128.5, 128.5, 127.7, 126.6, 125.1, 122.2, 110.2, 109.8, 63.1, 53.0, 46.5, 42.1. UPLC-DAD-QTOF: $C_{29}H_{26}N_3O_3$ [M+H]+ calcd.: 464.1969, found: 464. 1969. $C_{29}H_{25}N_3O_3Na$ [M+Na]+ calcd.: 486.1788, found: 486.1796.

[23] Yoo, S.-E.; Lee, S.-H. *Synlett* **1990**, 419-420.

[24] Michael, F. E.; Cochran, B. M. *J. Am. Chem. Soc.* **2006**, *128*, 4246-4247.

2.4.3. Preparation of spiro compound 6 by intramolecular silyl nitronate olefin cycloaddition (ISOC) of 4fa.[25]

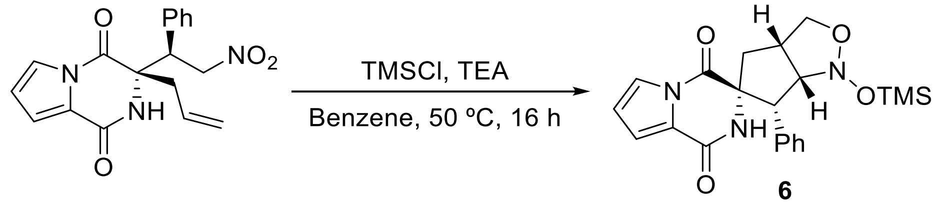

To a solution of the pyrrolo diketopiperazine **4fa** (102 mg, 0.3 mmol, 1 equiv.) in benzene (2.5 mL) under inert atmosphere were added freshly distilled Et_3N (0.250 mL, 1.8 mmol, 6 equiv.) and freshly distilled TMSCl (0.190 mL, 1.5 mmol, 5 equiv.). After addition was complete the mixture was warmed to 50 °C and stirred for 16 hours. Afterwards, the reaction mixture was cooled 0 °C, quenched with water (10 mL) and extracted with CH_2Cl_2 (2 x 15 mL). The combination of organic layer was dried over $MgSO_4$ and concentrated under reduced pressure. The crude product was purified by silica flash chromatography (eluting with Hexane/EtOAc 50/50 to 20/80) to produce the corresponding *N*-trimethylsilyloxyisoxazoline **6**. Yield: 104 mg, 0.25 mmol, 84%. m.p.= 151-154 °C.^{1}H NMR (300 MHz, $CDCl_3$) δ: 8.13 – 7.95 (m, 1H), 7.28 (dd, *J* = 3.2, 1.5 Hz, 1H), 7.21 – 7.07 (m, 5H), 6.83 (dd, *J* = 3.5, 1.4 Hz, 1H), 6.26 (t, *J* = 3.3 Hz, 1H), 4.65 (dd, *J* = 10.3, 8.4 Hz, 1H), 4.49 (dd, *J* = 8.6, 7.0 Hz, 1H), 3.83 (dd, *J* = 8.5, 1.4 Hz, 1H), 3.70 (dt, *J* = 15.8, 8.0 Hz, 1H), 3.27 (dd, *J* = 10.3, 1.8 Hz, 1H), 2.87 (dd, *J* = 13.4, 9.0 Hz, 1H), 1.97 (ddd, *J* = 13.7, 8.5, 1.7 Hz, 1H), -0.03 (s, 9H). ^{13}C NMR (75 MHz, $CDCl_3$) δ: 166.7, 157.7, 134.0, 128.6, 128.1, 128.0, 125.0, 119.1, 118.3, 115.5, 82.6, 73.4, 73.0, 60.9, 44.3, 41.9, -0.7. UPLC-DAD-QTOF: $C_{29}H_{26}N_3O_3$ [M+H]+ calcd.: 412.1687, found: 412.1697. $C_{29}H_{25}N_3O_3Na$ [M+Na]+ calcd.: 434.1507, found: 434.1516.

[25] Hassner, A.; Friedman, O.; Dehaen, W. *Liebigs Ann./Recueil* **1997**, 587-594.

2. Computational details

Catalyst **C7** structure was optimized using density functional theory (DFT) as implemented in Gaussian 16,[26] with B3LYP[27] as functional and 6-311G(d,p) as basis set, introducing solvation factors with the IEF-PCM[28] method (toluene as solvent). The stationary point of **C7** was characterized by frequency calculations, verifying that it did not contain any imaginary frequency. The electronic energy of the minimized structure was -1423.174659 Hartrees.

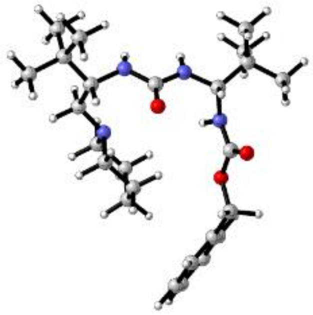

Standard orientation

Center Number	Atomic Number	Atomic Type	Coordinates (Angstroms) X	Y	Z
1	8	0	2.504933	-1.210033	-0.255259
2	6	0	1.534376	-2.068396	-0.670827
3	8	0	1.562579	-2.670517	-1.723543
4	7	0	0.559801	-2.156093	0.275593

[26] Gaussian 16, Revision A.03, Frisch, M. J.; Trucks, G. W.; Schlegel, H. B.; Scuseria, G. E.; Robb, M. A.; Cheeseman, J. R.; Scalmani, G.; Barone, V.; Petersson, G. A.; Nakatsuji, H.; Li, X.; Caricato, M.; Marenich, A. V.; Bloino, J.; Janesko, B. G.; Gomperts, R.; Mennucci, B.; Hratchian, H. P.; Ortiz, J. V.; Izmaylov, A. F.; Sonnenberg, J. L.; Williams-Young, D.; Ding, F.; Lipparini, F.; Egidi, F.; Goings, J.; Peng, B.; Petrone, A.; Henderson, T.; Ranasinghe, D.; Zakrzewski, V. G.; Gao, J.; Rega, N.; Zheng, G.; Liang, W.; Hada, M.; Ehara, M.; Toyota, K.; Fukuda, R.; Hasegawa, J.; Ishida, M.; Nakajima, T.; Honda, Y.; Kitao, O.; Nakai, H.; Vreven, T.; Throssell, K.; Montgomery, J. A., Jr.; Peralta, J. E.; Ogliaro, F.; Bearpark, M. J.; Heyd, J. J.; Brothers, E. N.; Kudin, K. N.; Staroverov, V. N.; Keith, T. A.; Kobayashi, R.; Normand, J.; Raghavachari, K.; Rendell, A. P.; Burant, J. C.; Iyengar, S. S.; Tomasi, J.; Cossi, M.; Millam, J. M.; Klene, M.; Adamo, C.; Cammi, R.; Ochterski, J. W.; Martin, R. L.; Morokuma, K.; Farkas, O.; Foresman, J. B.; Fox, D. J. Gaussian, Inc., Wallingford CT, **2016.**

[27] a) C. Lee, W. Yang, R. G. Parr, *Phys. Rev. B* 1988, **37**, 785-789; b) A. D. Becke, *J. Chem. Phys*. 1993, **98**, 5648-5652; c) W. Khon, A. D. Becke, G. R. Parr, *J. Phys. Chem*. 1996, **100**, 12974-12980.

[28] a) E. Cances, B. Mennucci and J. Tomasi, *J. Chem. Phys.*, **1997**, *107*, 3032-3041; b) M.Cossi, V. Barone, B. Mennucci and J. Tomasi, *Chem. Phys. Lett.*, **1998**, *286*, 253-260; c) J. Tomasi, B. Mennucci and E. Cances, *J. Mol.Struc.: THEOCHEM*, **1999**, *464*, 211-226.

5	1	0	0.595491	-1.470901	1.015444
6	6	0	-0.764903	-2.710653	-0.047809
7	1	0	-0.803137	-2.788325	-1.134651
8	6	0	-0.985307	-4.125426	0.558637
9	6	0	-2.342582	-4.664173	0.074027
10	6	0	-0.947236	-4.086025	2.096787
11	6	0	0.136337	-5.050252	0.053766
12	1	0	-3.170929	-4.039219	0.414556
13	1	0	-2.377793	-4.701765	-1.018730
14	1	0	-2.502512	-5.677975	0.451238
15	1	0	-1.782356	-3.520685	2.524037
16	1	0	-1.017390	-5.100143	2.498745
17	1	0	-0.011815	-3.648263	2.455018
18	1	0	0.188034	-5.047954	-1.037018
19	1	0	1.109964	-4.733241	0.432304
20	1	0	-0.044582	-6.073937	0.392845
21	7	0	-1.794117	-1.785973	0.363710
22	1	0	-2.131190	-1.846530	1.310948
23	7	0	-3.025901	0.167156	0.185510
24	1	0	-3.182406	0.041275	1.175210
25	6	0	3.597218	-1.004481	-1.181551
26	1	0	4.166277	-1.935629	-1.260435
27	1	0	3.196051	-0.769748	-2.168292
28	6	0	4.439895	0.119097	-0.645139
29	6	0	4.955492	0.055436	0.653570

30	6	0	4.707435	1.242952	-1.427159
31	6	0	5.719547	1.101340	1.161616
32	1	0	4.738071	-0.810376	1.268891
33	6	0	5.480395	2.289004	-0.923794
34	1	0	4.298968	1.306843	-2.429965
35	6	0	5.984923	2.221736	0.372417
36	1	0	6.108223	1.045142	2.172239
37	1	0	5.677032	3.158964	-1.540021
38	1	0	6.578741	3.037673	0.768537
39	6	0	-2.089671	-0.663871	-0.389732
40	6	0	-4.694012	1.918212	-0.427033
41	6	0	-5.431969	1.828757	0.921372
42	6	0	-5.344635	0.938910	-1.422476
43	6	0	-4.828539	3.346324	-0.989778
44	1	0	-4.236208	3.466360	-1.902247
45	1	0	-5.873128	3.552588	-1.239838
46	1	0	-4.506834	4.105968	-0.274005
47	1	0	-4.859659	0.998033	-2.401395
48	1	0	-5.262798	-0.090905	-1.070532
49	1	0	-6.404380	1.176702	-1.553584
50	1	0	-5.388947	0.816500	1.333929
51	1	0	-5.022076	2.521865	1.661010
52	1	0	-6.488307	2.079340	0.788910
53	8	0	-1.597698	-0.476612	-1.494285
54	6	0	-2.355815	2.491371	0.598841

55	1	0	-2.754352	2.454791	1.617951
56	1	0	-2.474140	3.530111	0.249987
57	6	0	-0.230835	2.444191	-0.581367
58	6	0	-0.281964	2.704704	1.827664
59	6	0	1.219389	1.965446	-0.535822
60	1	0	-0.257674	3.538644	-0.758444
61	1	0	-0.739151	1.956627	-1.412988
62	6	0	1.162542	2.216616	1.954155
63	1	0	-0.285010	3.812309	1.770091
64	1	0	-0.851757	2.430599	2.721125
65	6	0	1.956107	2.528441	0.681934
66	1	0	1.725779	2.254879	-1.461707
67	1	0	1.227133	0.874031	-0.493494
68	1	0	1.627894	2.679282	2.830537
69	1	0	1.156296	1.133090	2.121722
70	1	0	2.965925	2.118958	0.743868
71	1	0	2.055861	3.616987	0.578094
72	7	0	-0.944779	2.114267	0.660974
73	1	0	-2.771435	1.547766	-1.296833
74	6	0	-3.186074	1.541084	-0.288778

----

3. X-Ray Crystallographic data of 3ae

Solvent System: Dichloromethane/*n*-Hexane; Crystallization Method: Slow evaporation of the solvent at room temperature.

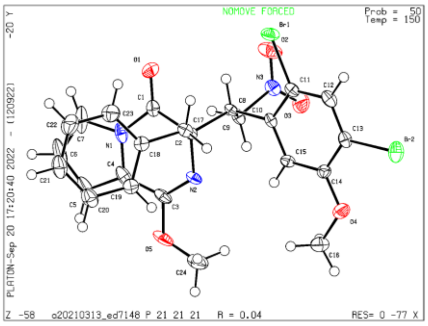

X-ray structure of **3ae** with 50% thermal ellipsoids probability

Crystal data and structure refinement for 3ae: CCDC 2210448

Empirical formula	$C_{24}H_{21}Br_2N_3O_5$
Formula weight	591.26
Temperature/K	149.93(16)
Crystal system	orthorhombic
Space group	P212121
a/Å	9.91489(13)
b/Å	14.37037(19)
c/Å	16.7111(2)
α/°	90.0
β/°	90.0
γ/°	90.0
Volume/$Å^3$	2381.01(5)
Z	4
$\rho_{calc}g/cm^3$	1.649
μ/mm^{-1}	4.672
F(000)	1184.0
Crystal size/mm^3	0.477 × 0.214 × 0.139
Radiation	CuKα (λ = 1.54184)
2Θ range for data collection/°	8.114 to 137.962
Index ranges	-12 ≤ h ≤ 12, -17 ≤ k ≤ 17, -17 ≤ l ≤ 20
Reflections collected	21755
Independent reflections	4409 [Rint = 0.0520, Rsigma = 0.0371]
Data/restraints/parameters	4409/0/309
Goodness-of-fit on	F2 1.077
Final R indexes [I>=2σ (I)]	R_1 = 0.0393, wR_2 = 0.0977
Final R indexes [all data]	R_1 = 0.0417, wR_2 = 0.0995
Largest diff. peak/hole /	e $Å^{-3}$ 0.44/-0.52
Flack parameter	-0.042(13)
Bijvoet Pairs Covarage	100%
Hooft y	-0.044(9)
P3 false ≤10^{-99}	

4. ^{1}H and ^{13}C NMR spectra for selected compounds

Benzyl ((*S*)-2,2-dimethyl-1-(3-((1*S*,2*S*)-2-(piperidin-1-yl)cyclohexyl)ureido)propyl)carbamate (C4)

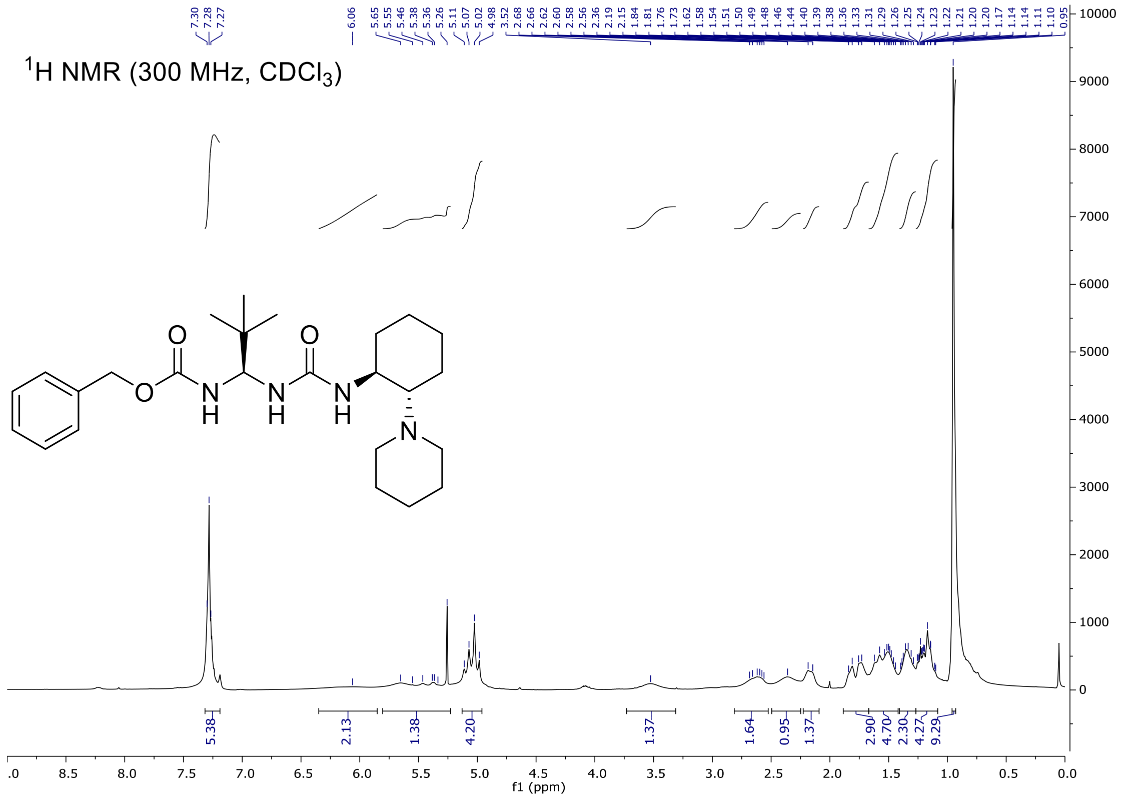

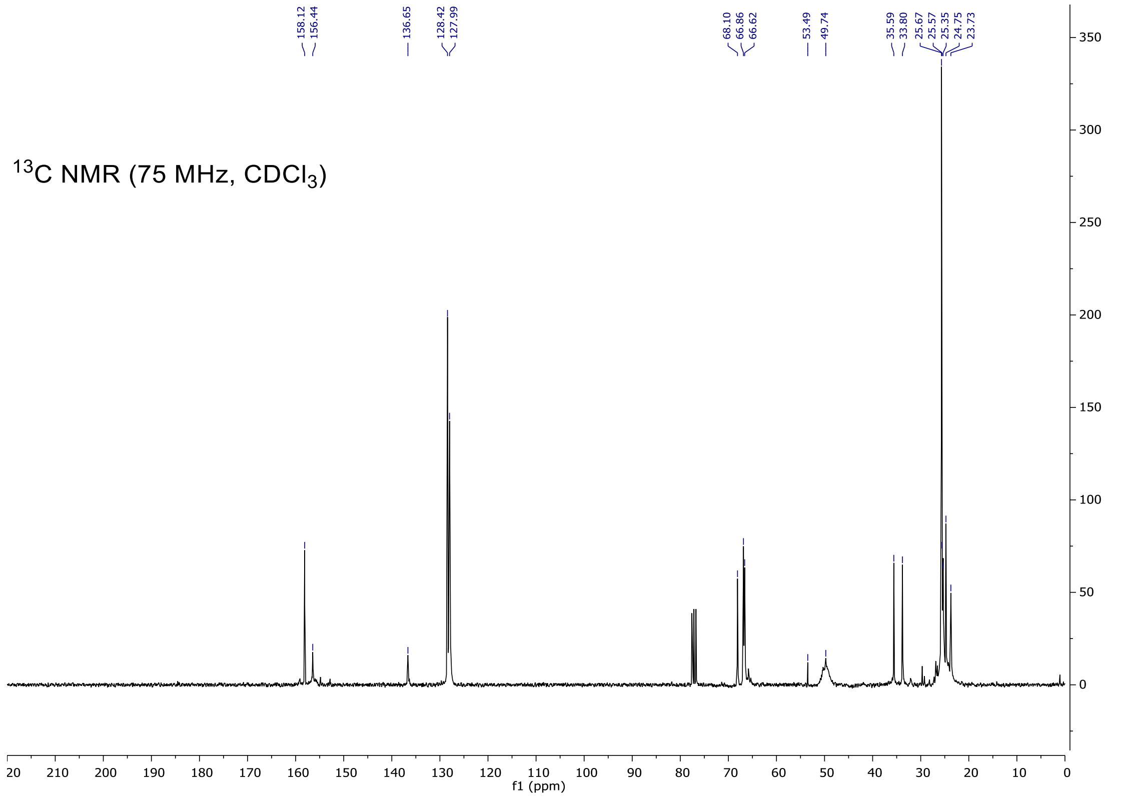

3,5-Bis(trifluoromethyl)benzyl ((1*S*)-2,2-dimethyl-1-(3-((2*S*)-2-(piperidin-1-yl)cyclohexyl)ureido)propyl)carbamate (C5)

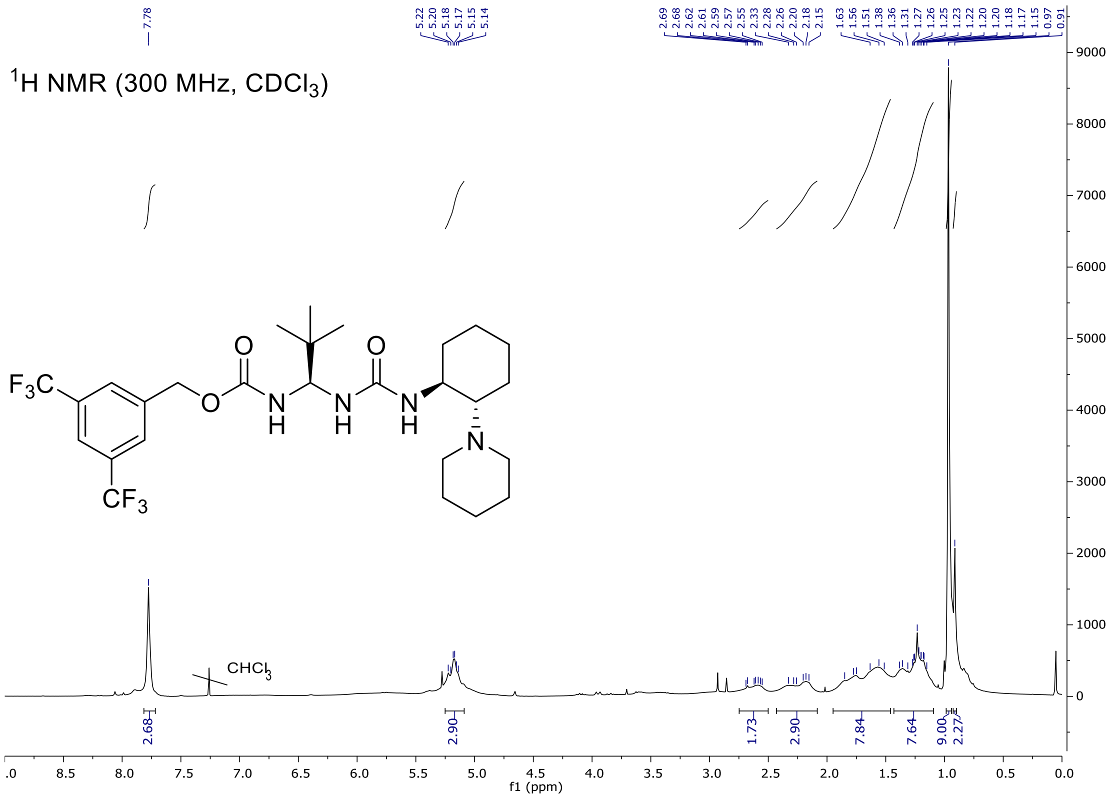

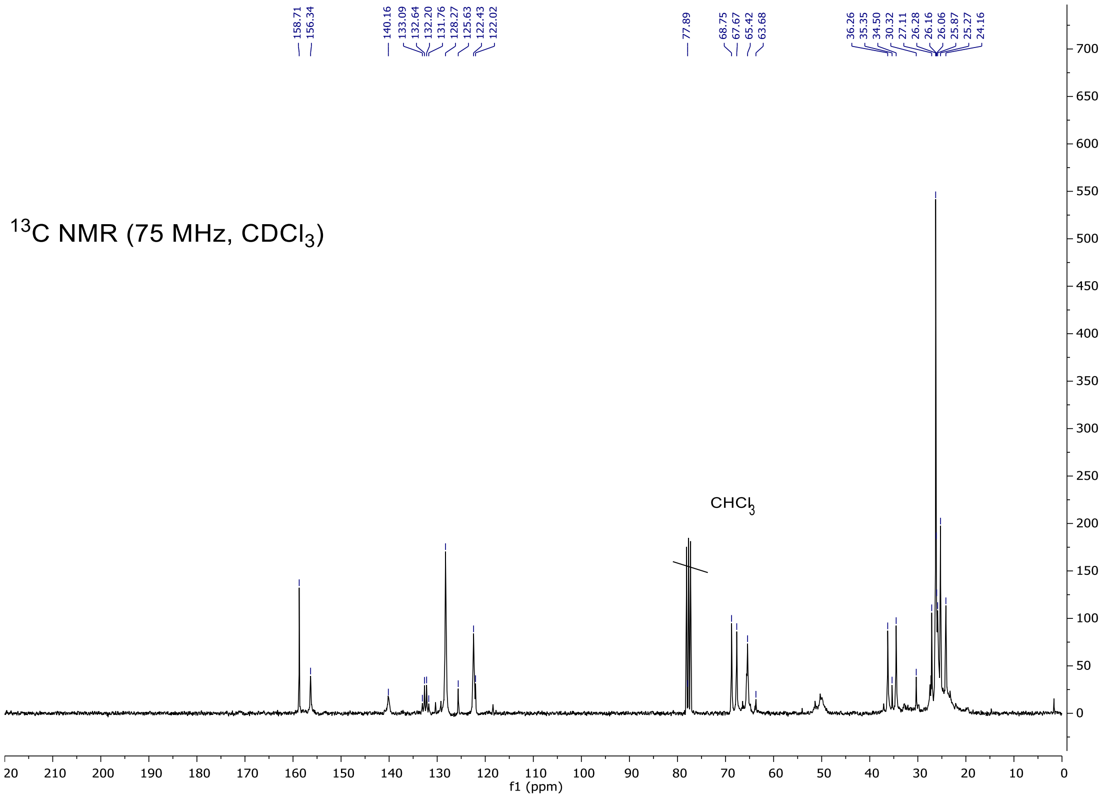

Benzyl ((S)-1-(3-((1S,2S)-2-(diisobutylamino)cyclohexyl)ureido)-2,2-dimethylpropyl)carbamate (C6)

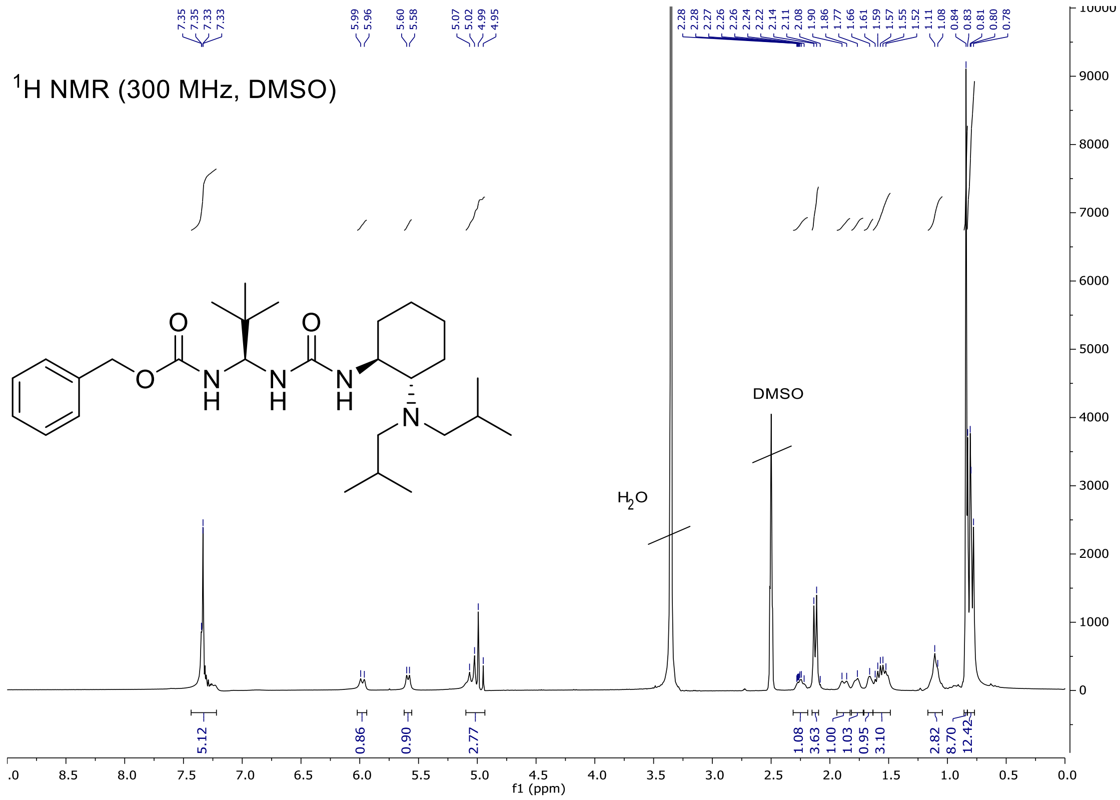

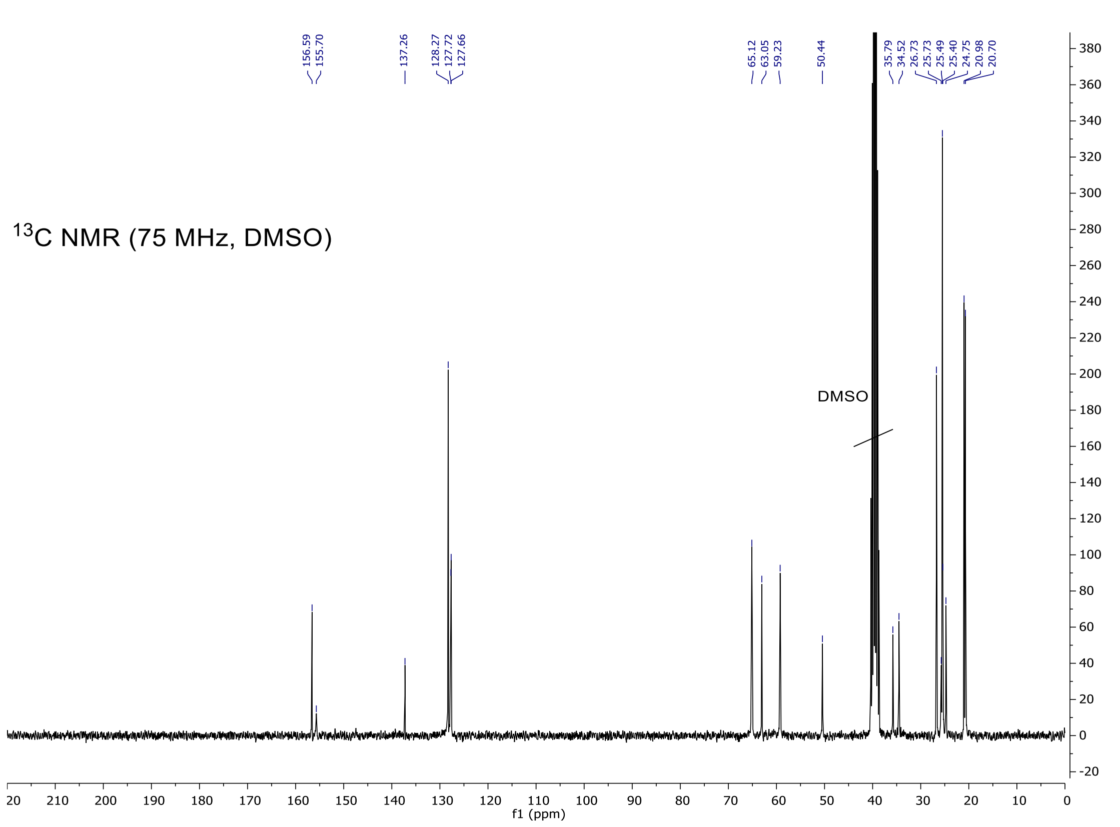

Benzyl ((S)-1-(3-((S)-3,3-dimethyl-1-(piperidin-1-yl)butan-2-yl)ureido)-2,2-dimethylpropyl)carbamate (C7)

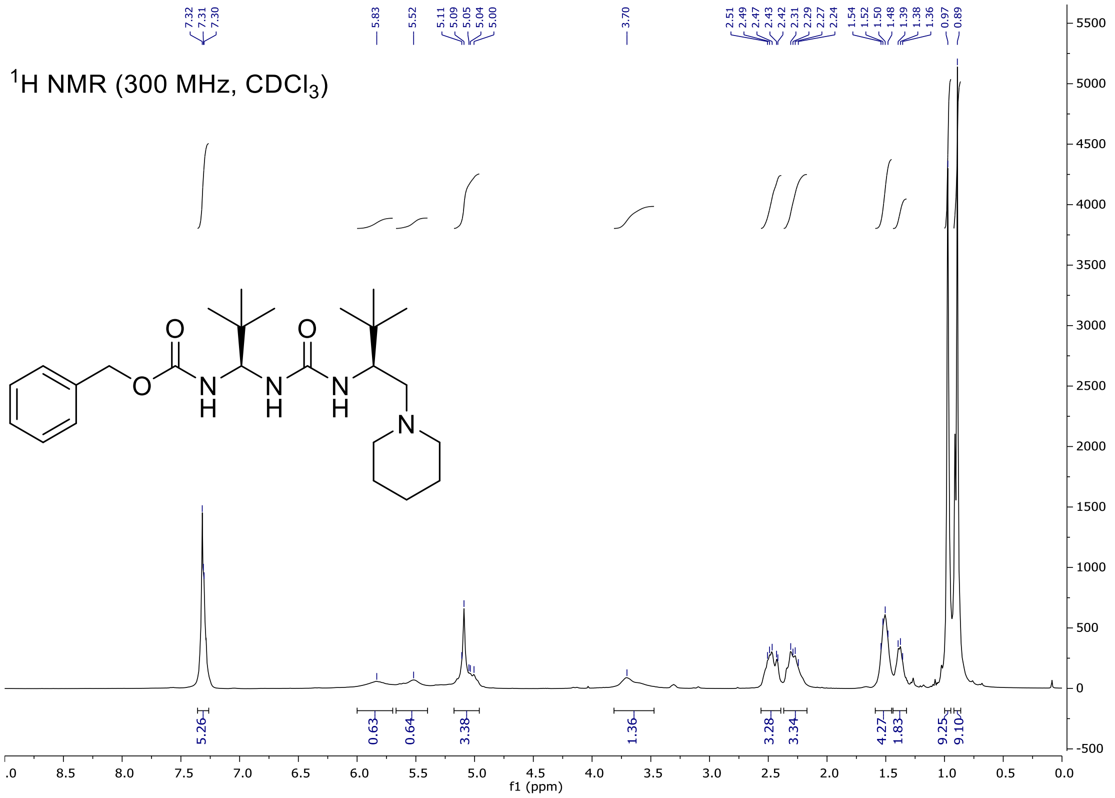

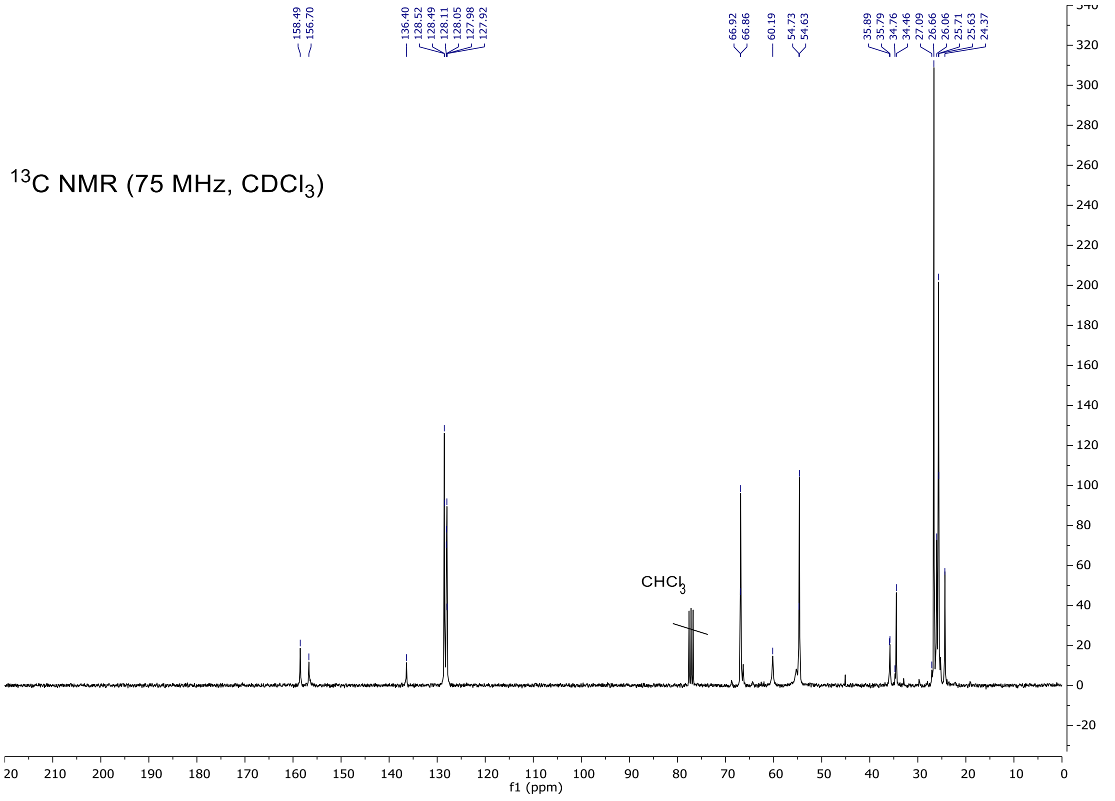

Naphthalen-1-ylmethyl ((S)-2,2-dimethyl-1-(3-((1R,2R)-2-(piperidin-1-yl)cyclohexyl)ureido)propyl)carbamate (diast-C1)

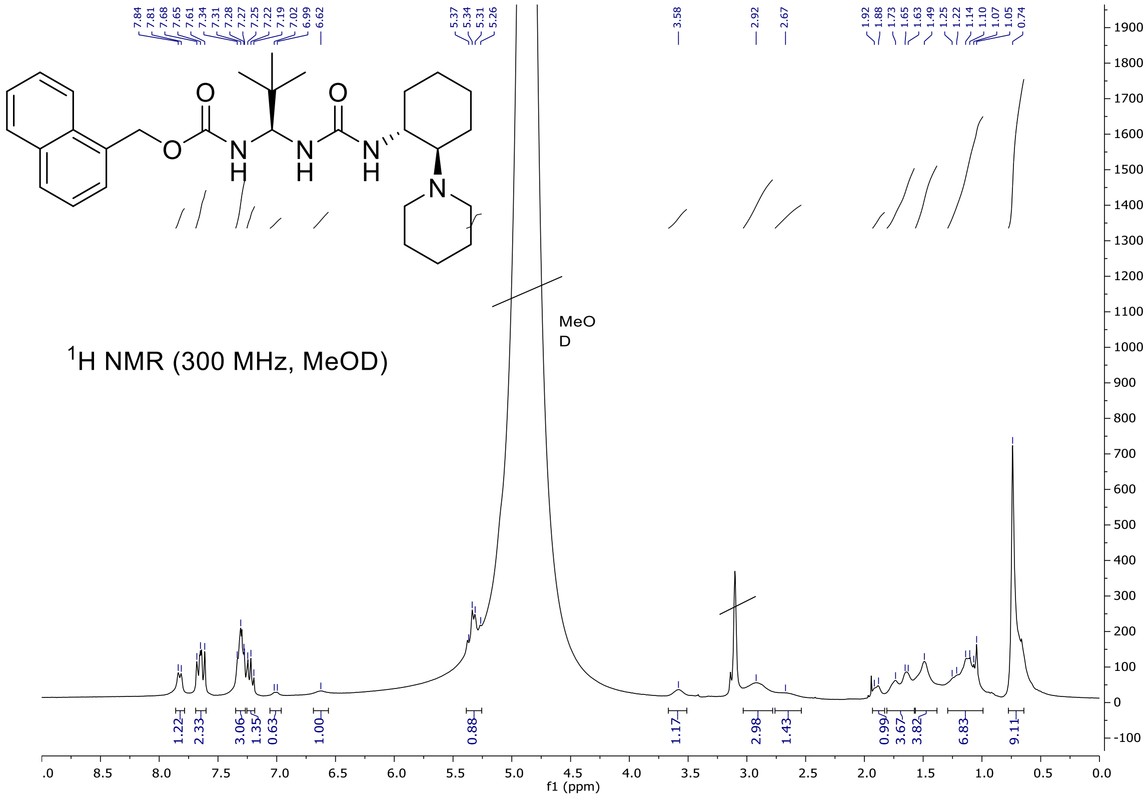

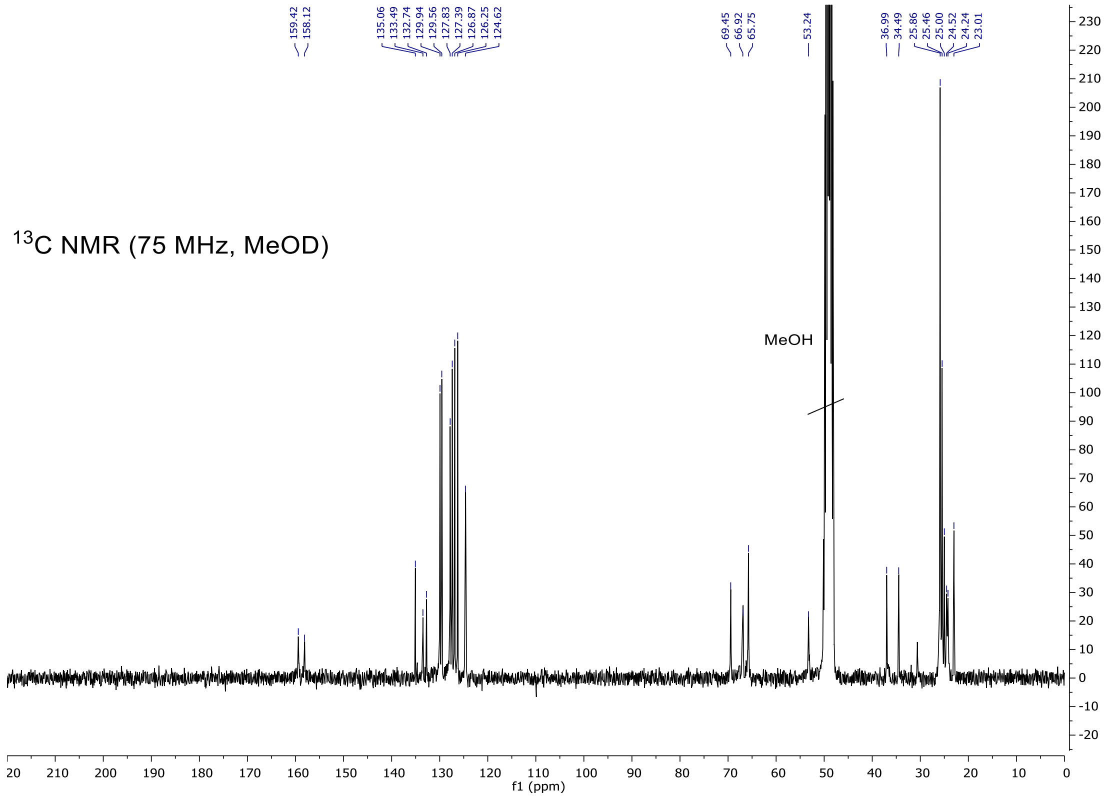

Naphthalen-1-ylmethyl ((S)-1-(3-((S)-(6-methoxyquinolin-4-yl)((1S,2S,4S,5R)-5-vinylquinuclidin-2-yl)methyl)ureido)-2,2-dimethylpropyl)carbamate (C8)

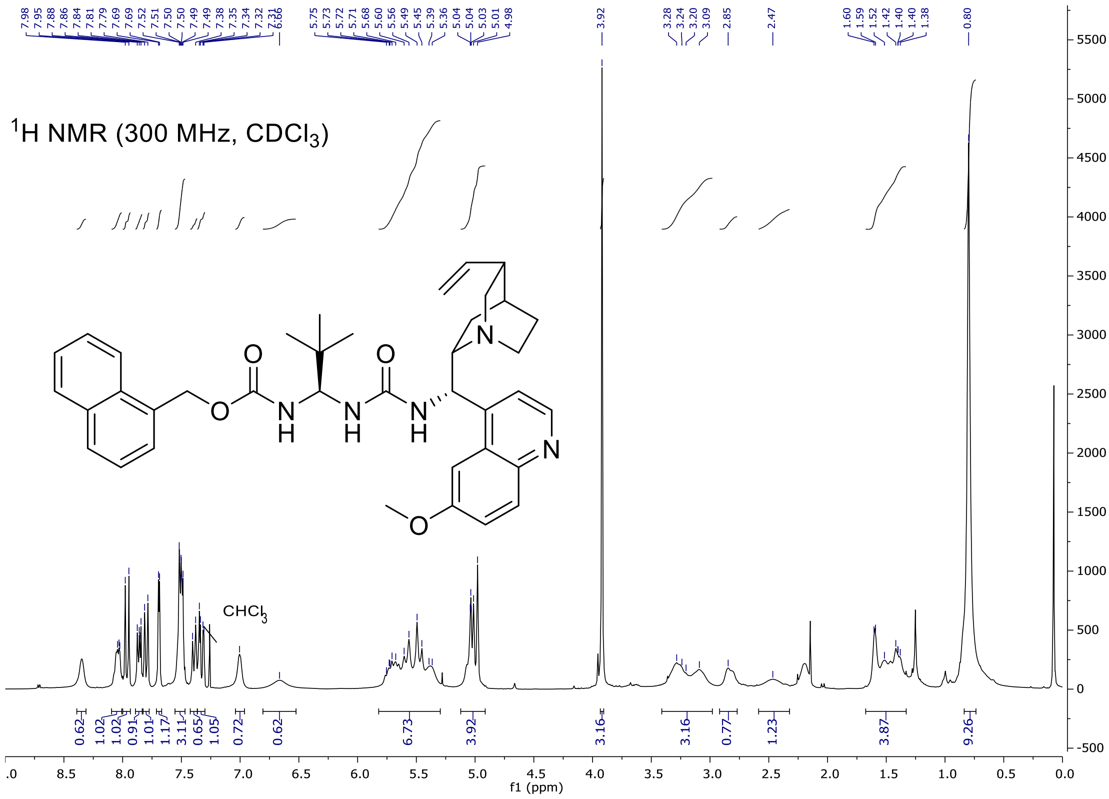

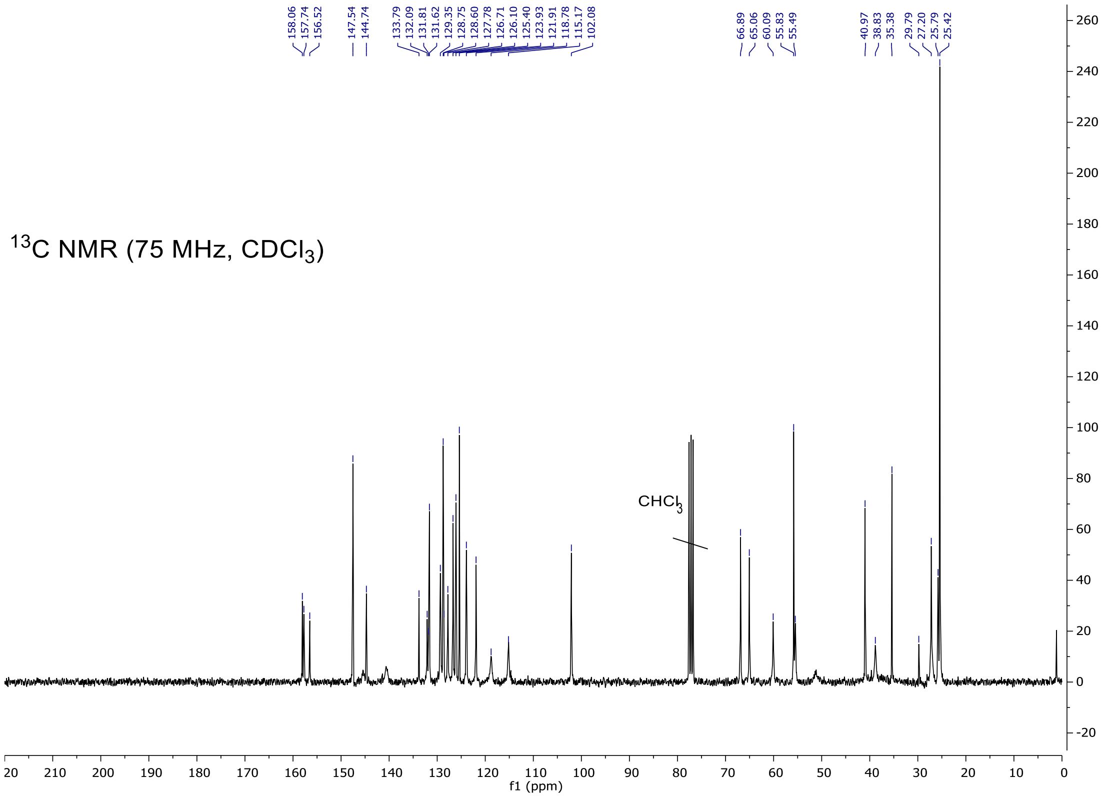

Methyl (1*H*-pyrrole-2-carbonyl)-*L*-phenylalaninate

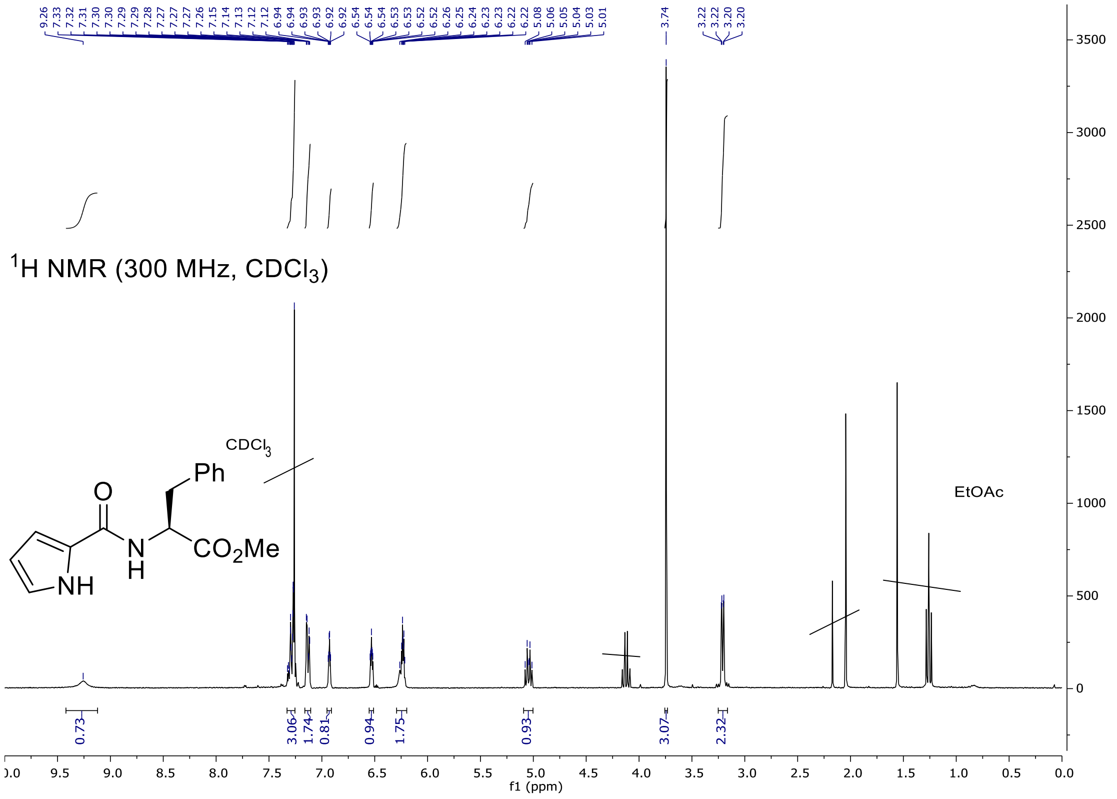

Methyl (1*H*-pyrrole-2-carbonyl)-*L*-leucinate

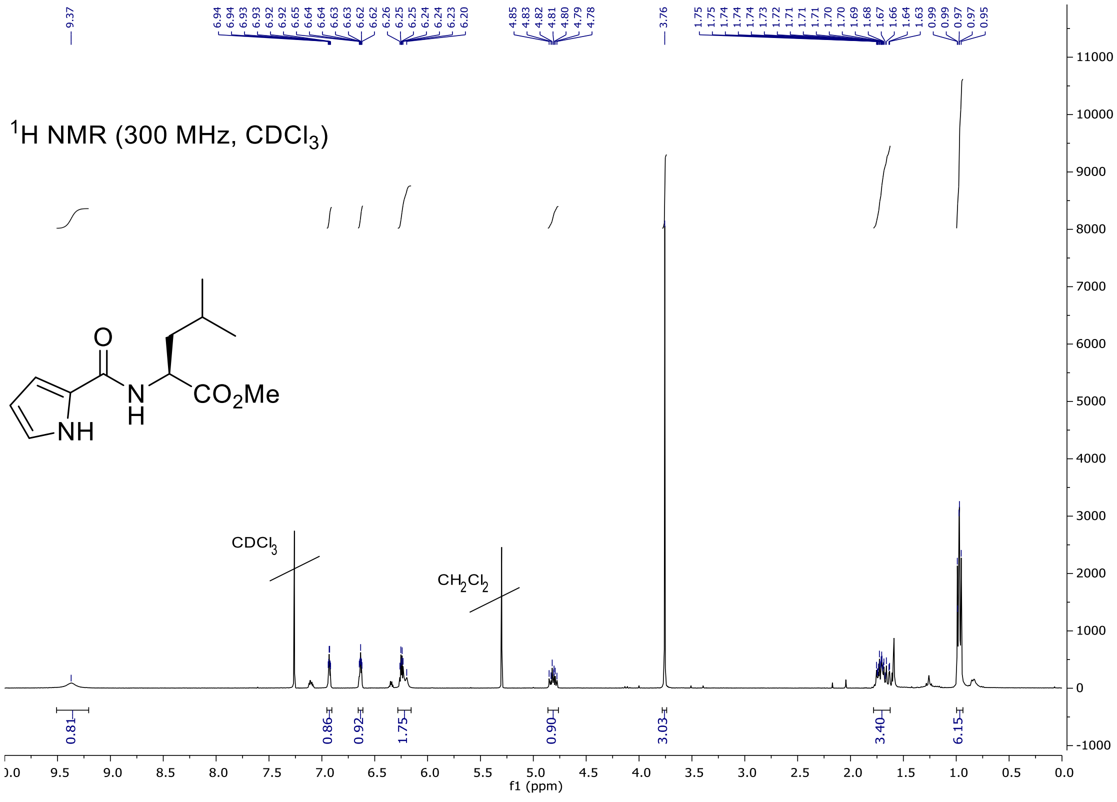

Methyl (*S*)-3-(4-methoxyphenyl)-2-(1*H*-pyrrole-2-carboxamido)propanoate

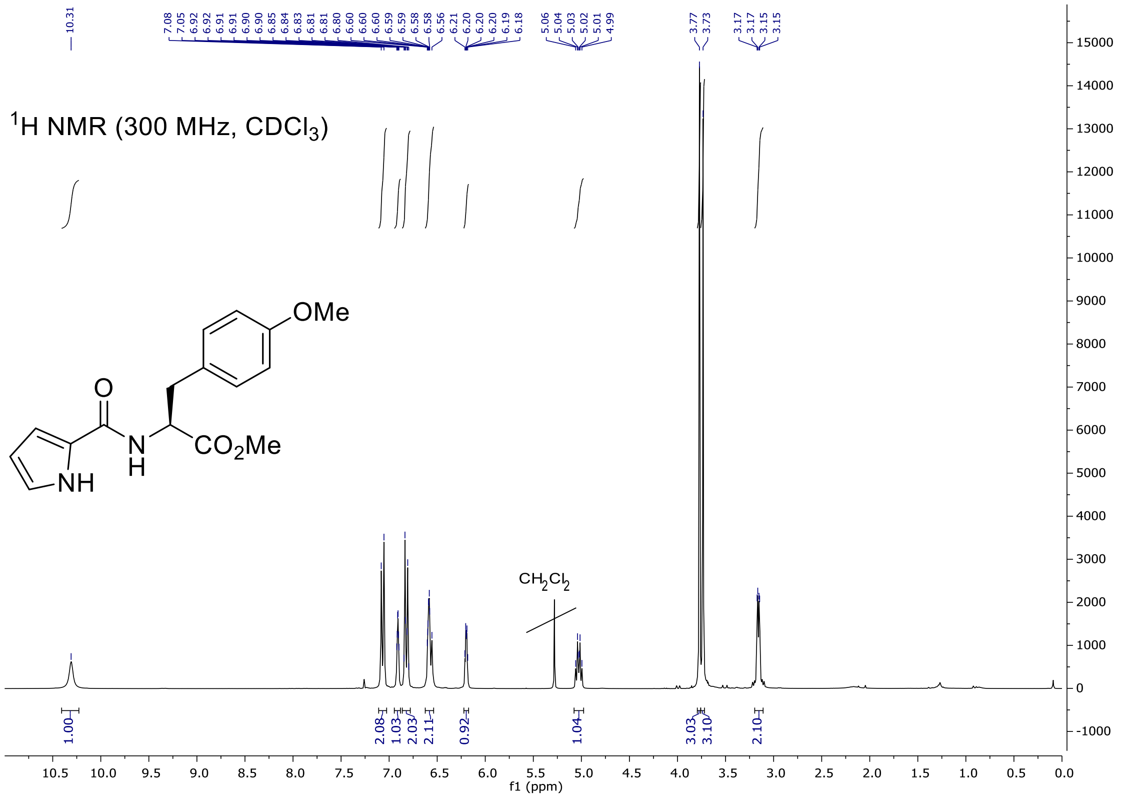

Methyl (1*H*-pyrrole-2-carbonyl)-*L*-tryptophanate

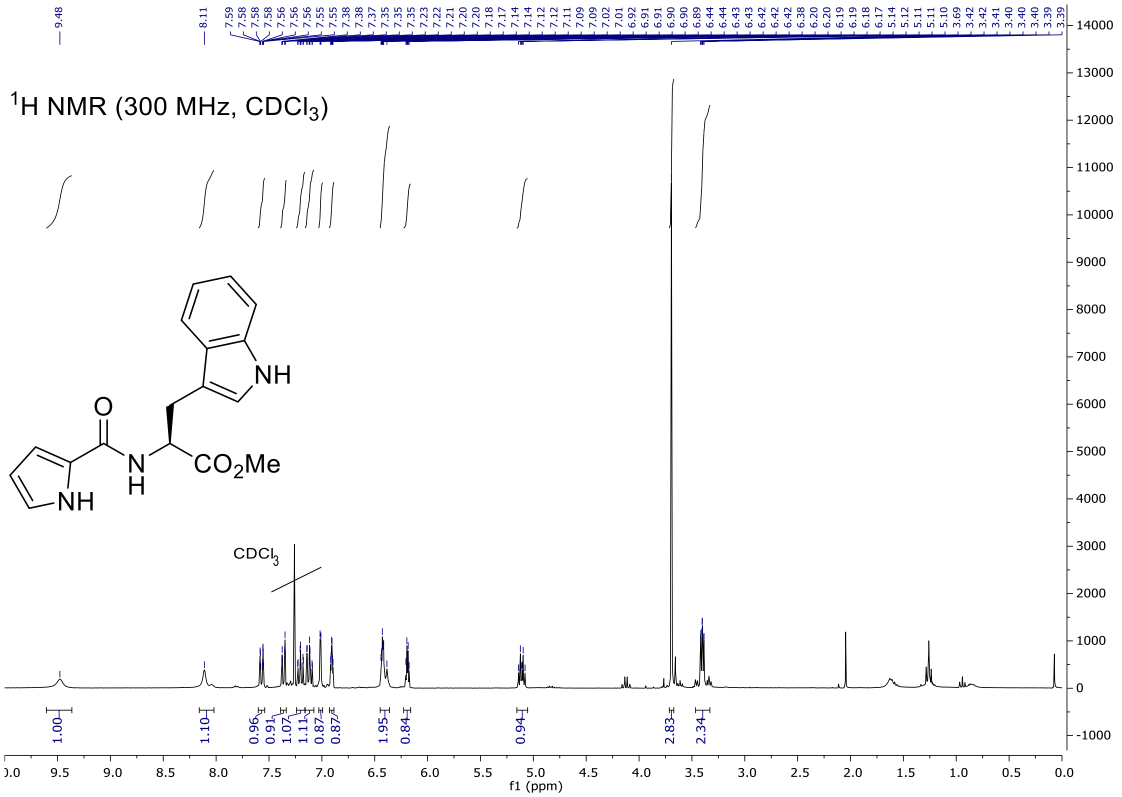

Methyl 4-phenyl-2-(1*H*-pyrrole-2-carboxamido)butanoate

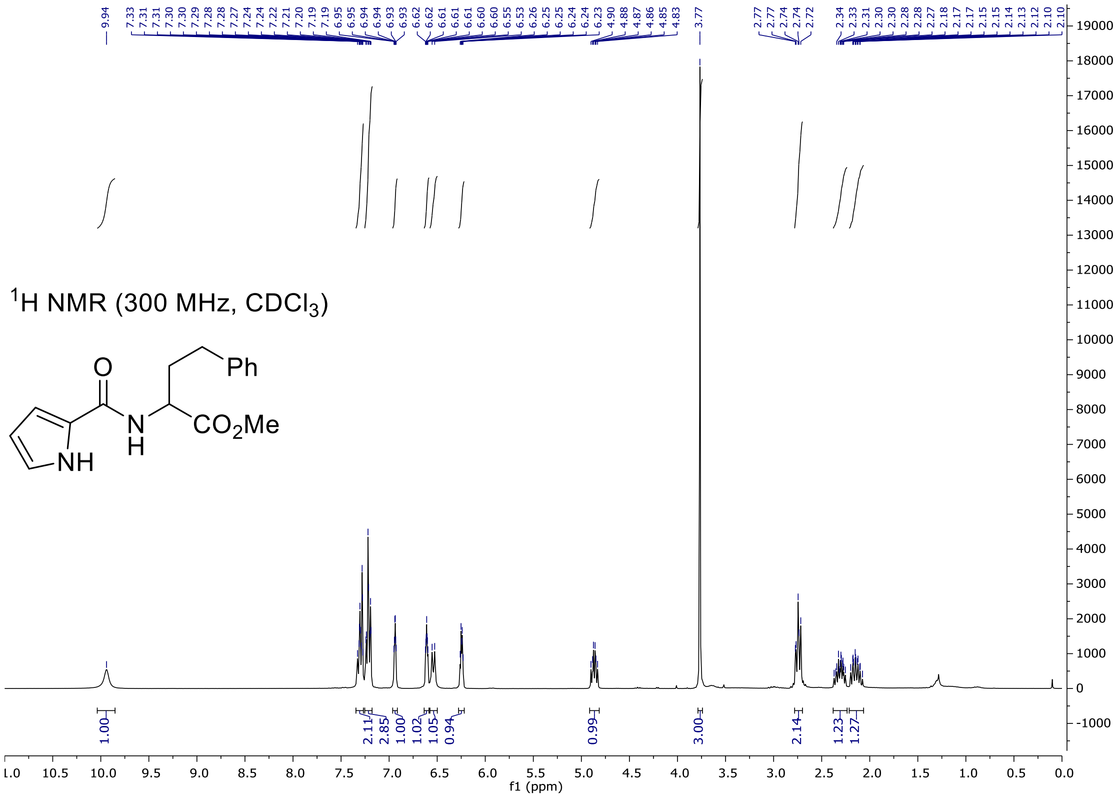

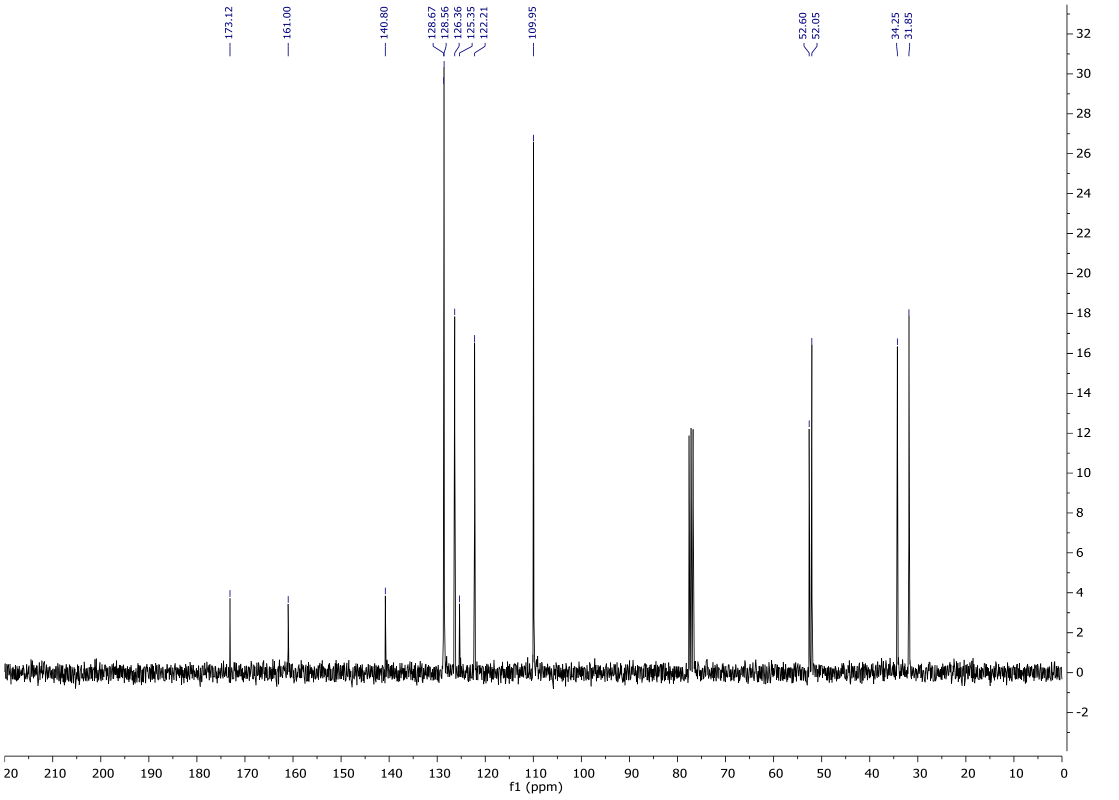

Methyl 2-(1*H*-pyrrole-2-carboxamido)pent-4-enoate

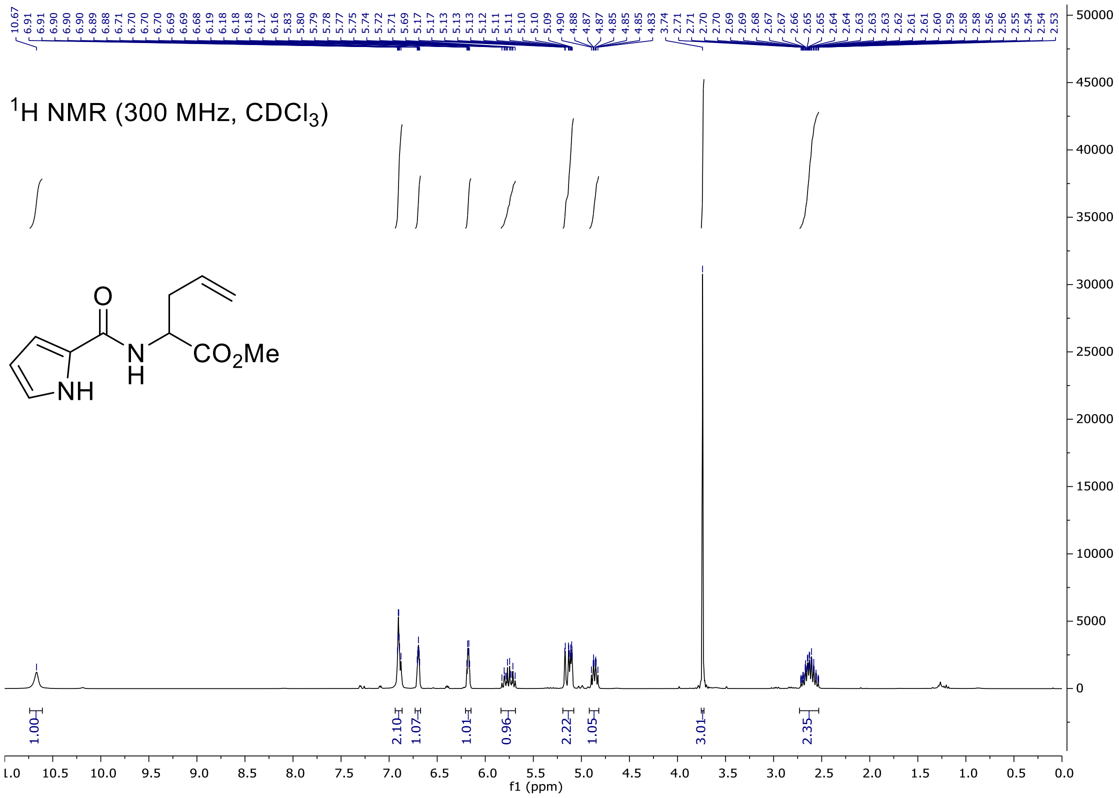

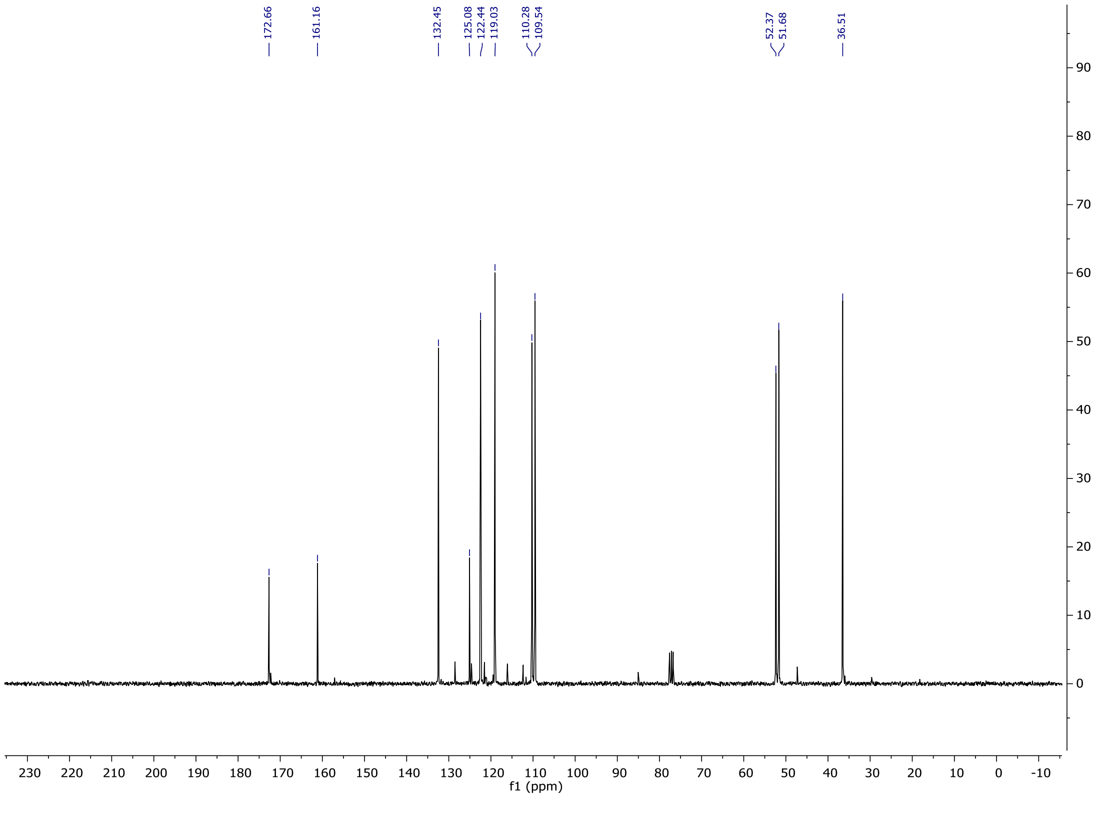

Methyl (*S*)-2-phenyl-2-(1*H*-pyrrole-2-carboxamido)acetate

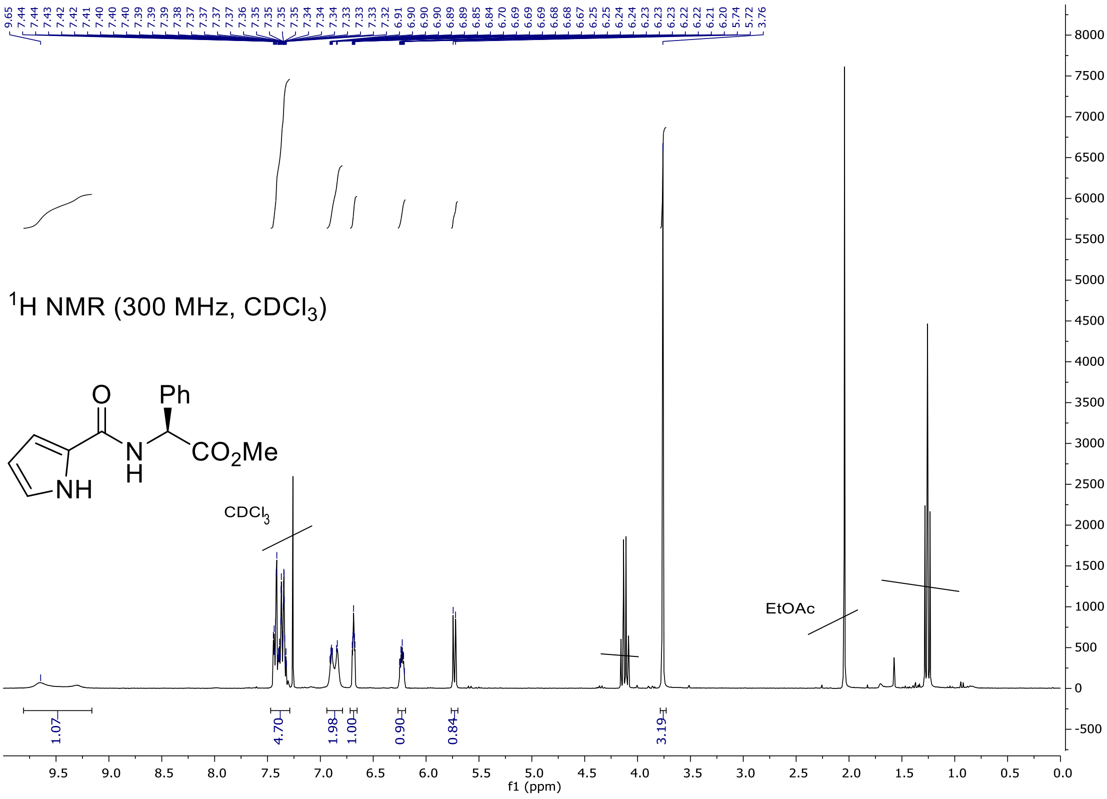

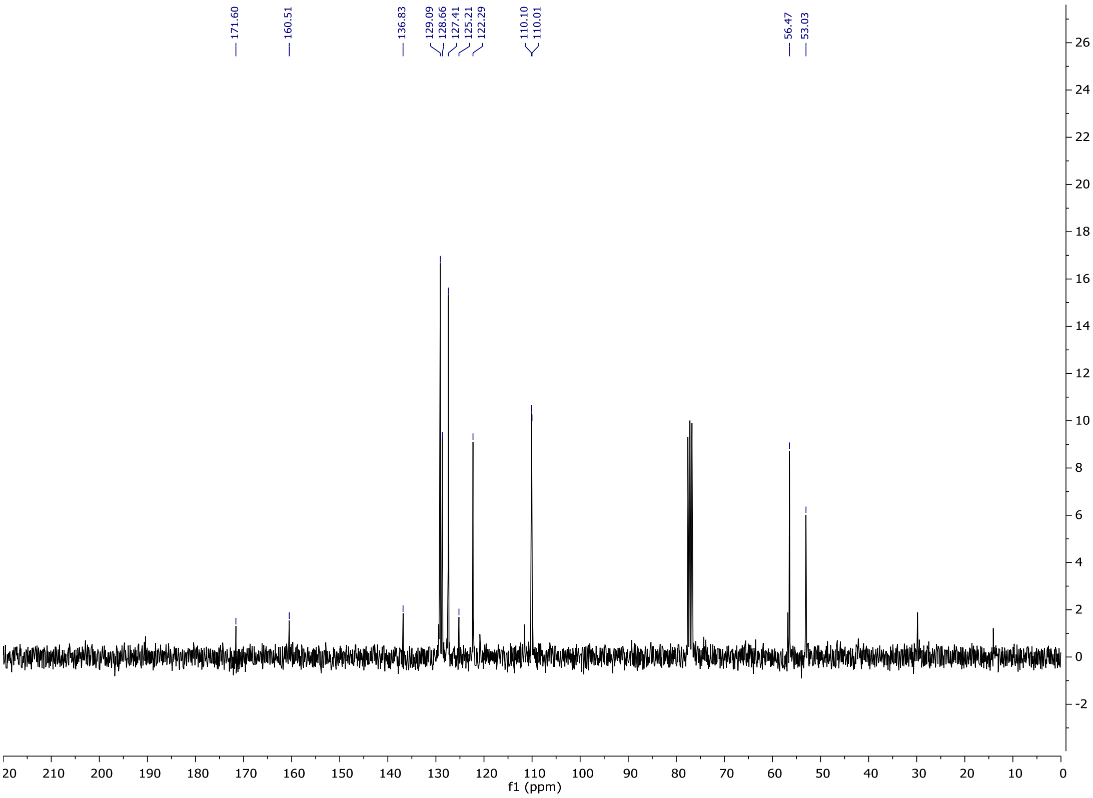

Methyl 2-(1*H*-pyrrole-2-carboxamido)octanoate

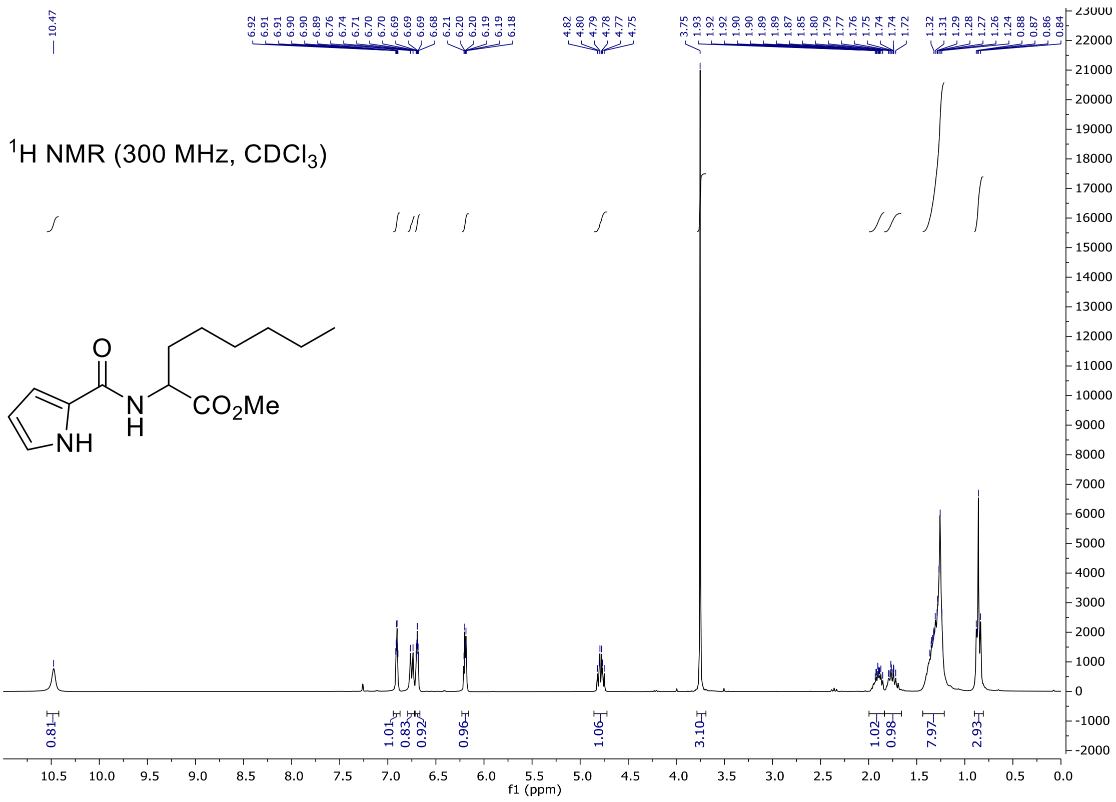

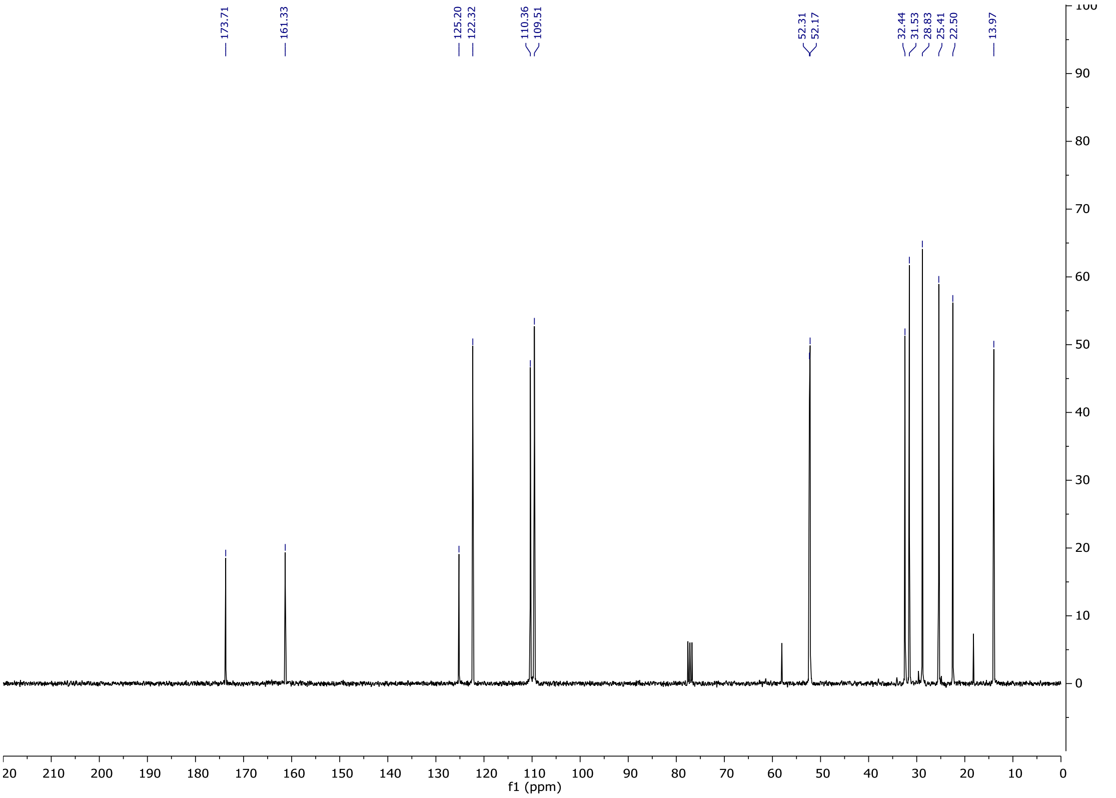

(*S*)-3-Benzyl-1-methoxypyrrolo[1,2-*a*]pyrazin-4(3*H*)-one (1a)

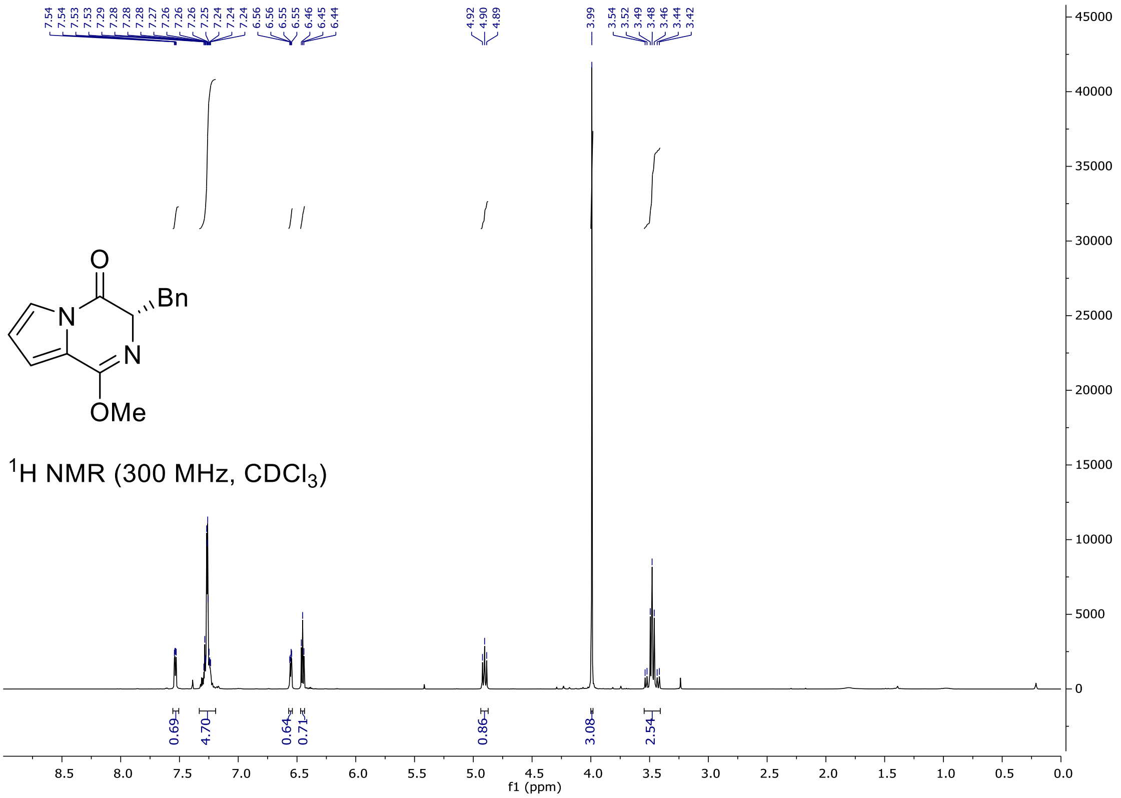

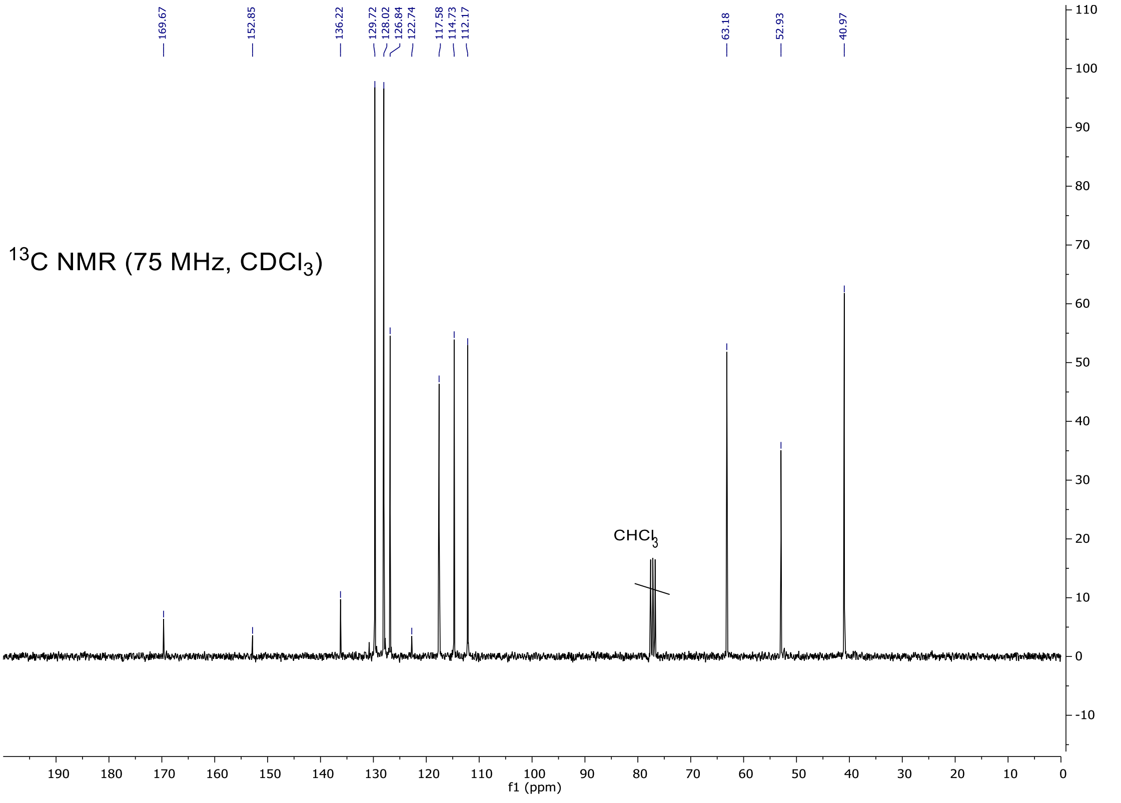

(*S*)-3-Isobutyl-1-methoxypyrrolo[1,2-*a*]pyrazin-4(3*H*)-one (1b)

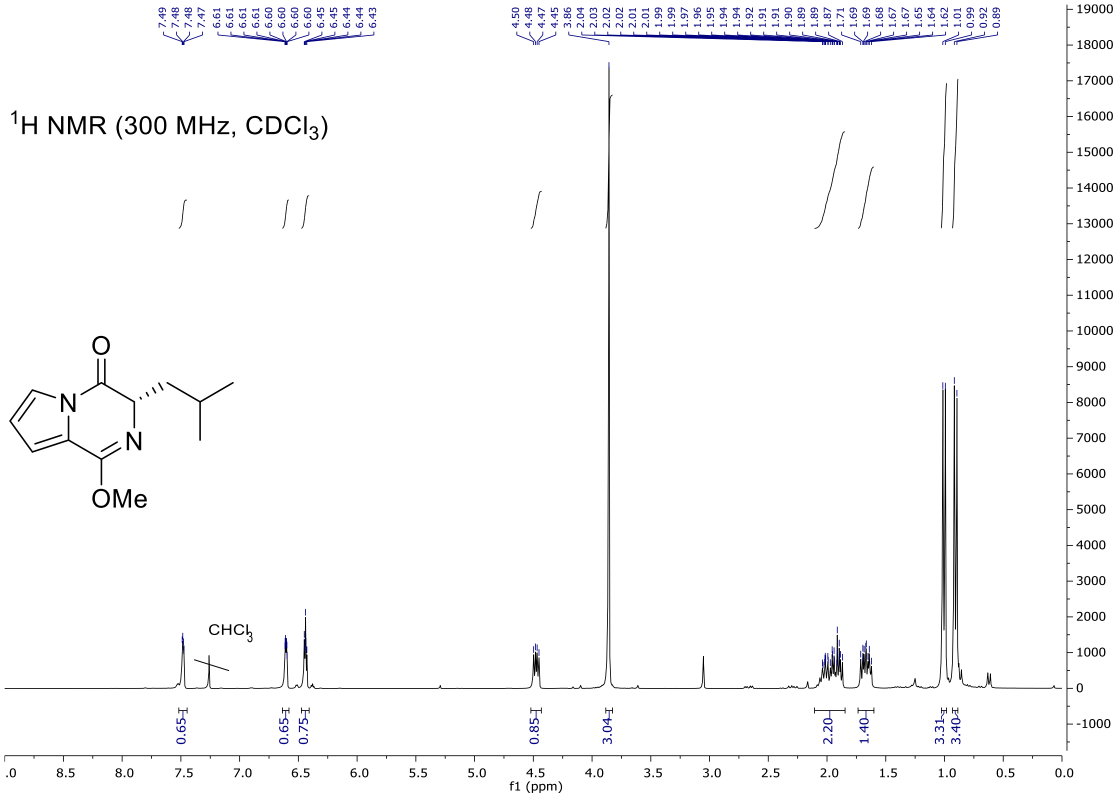

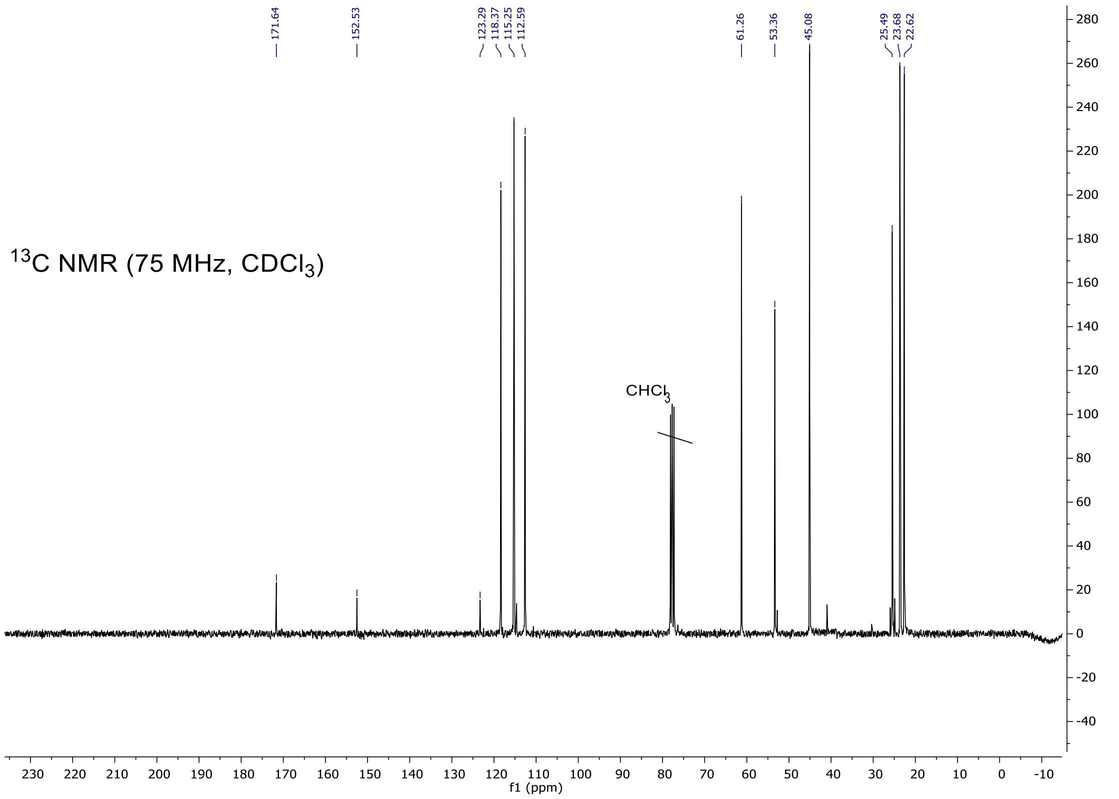

(*S*)-1-Methoxy-3-(4-methoxybenzyl)pyrrolo[1,2-*a*]pyrazin-4(3*H*)-one (1c)

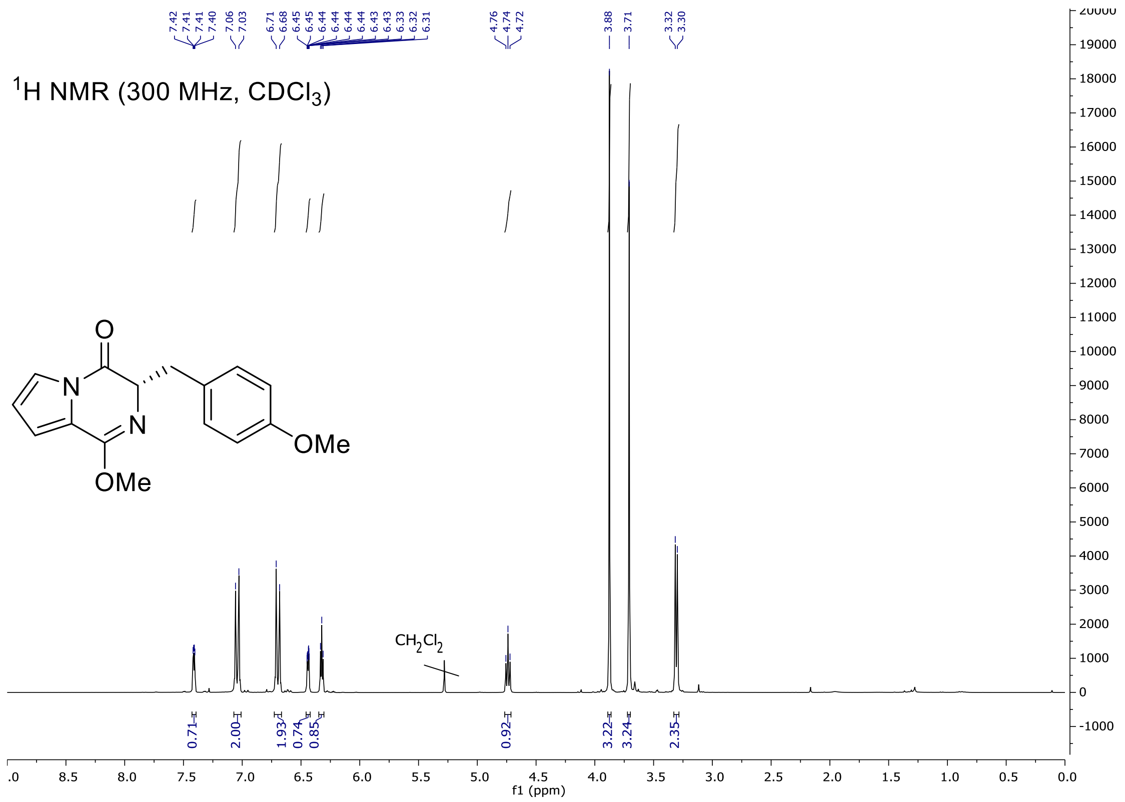

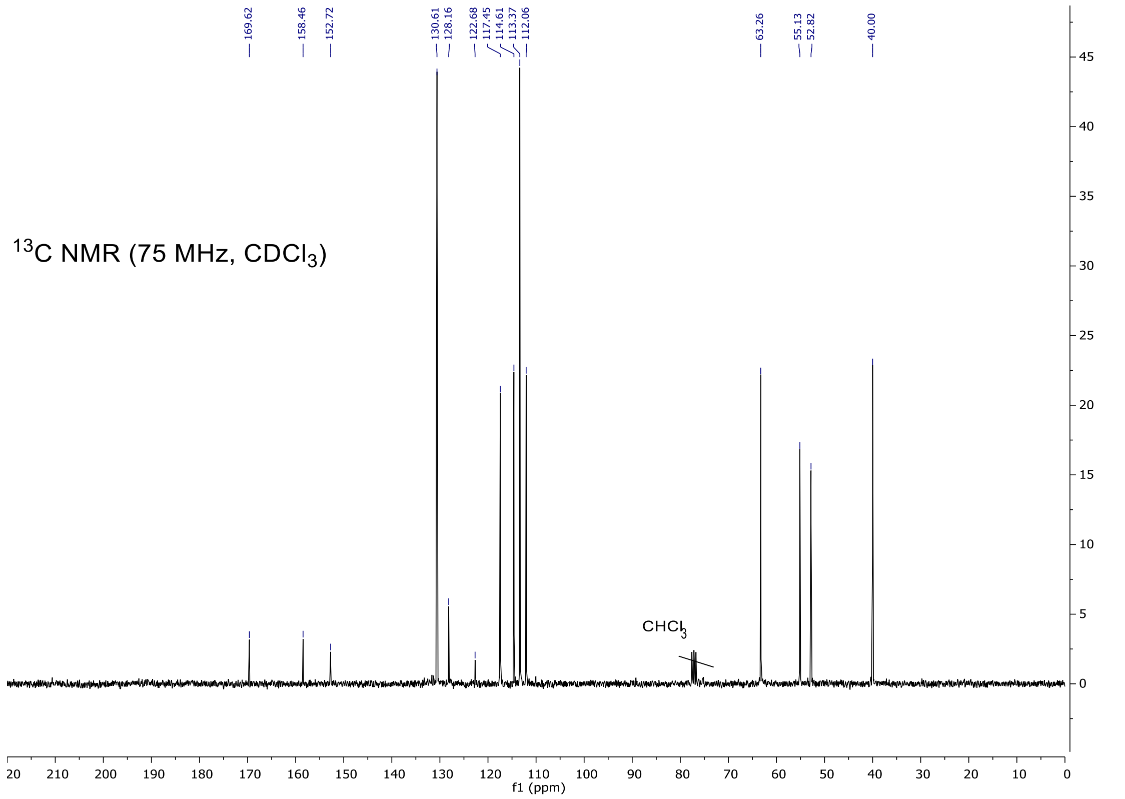

1-Methoxy-3-phenethylpyrrolo[1,2-*a*]pyrazin-4(3*H*)-one (1d)

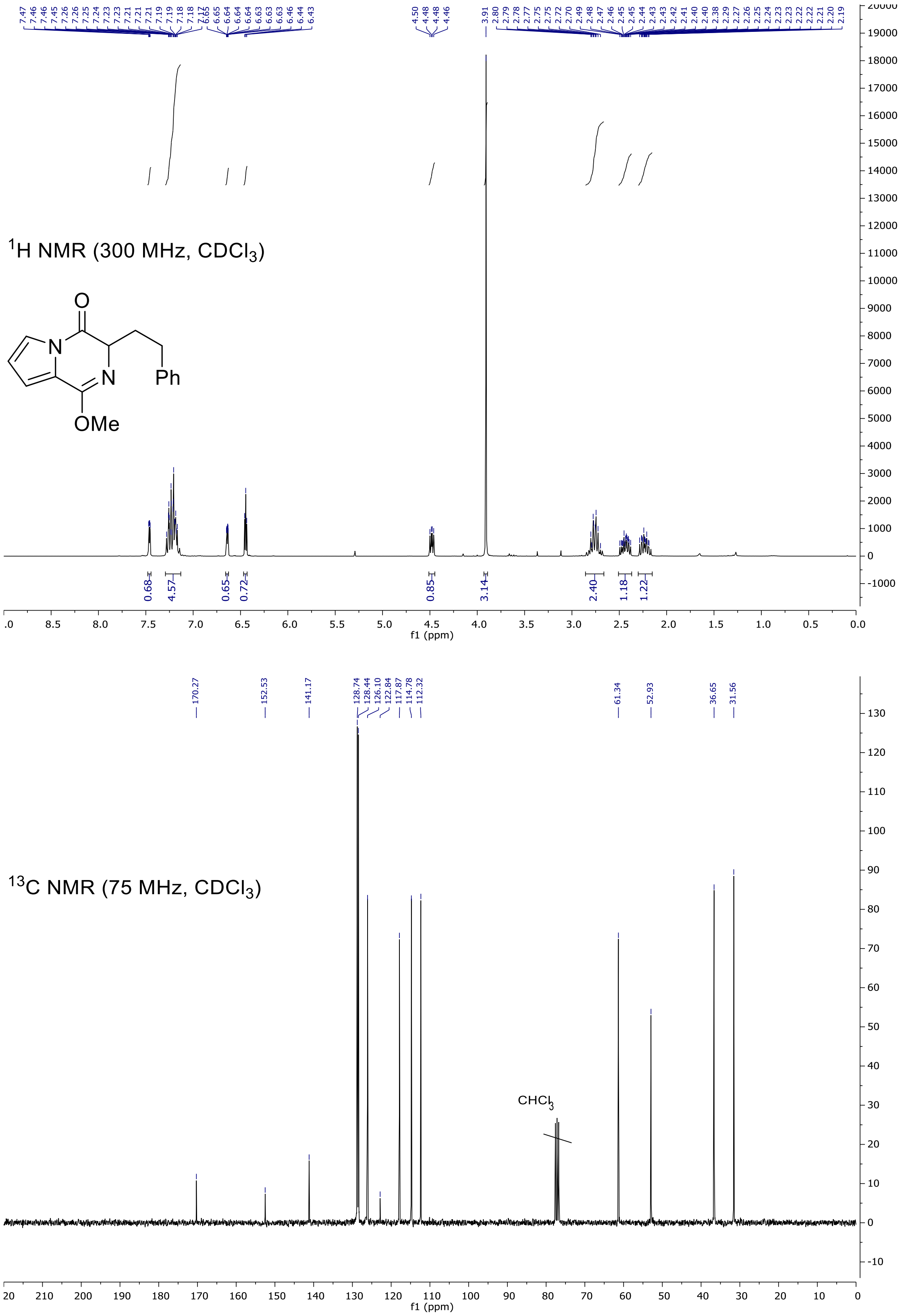

(*S*)-3-((1*H*-Indol-3-yl)methyl)-1-methoxypyrrolo[1,2-*a*]pyrazin-4(3*H*)-one (1e)

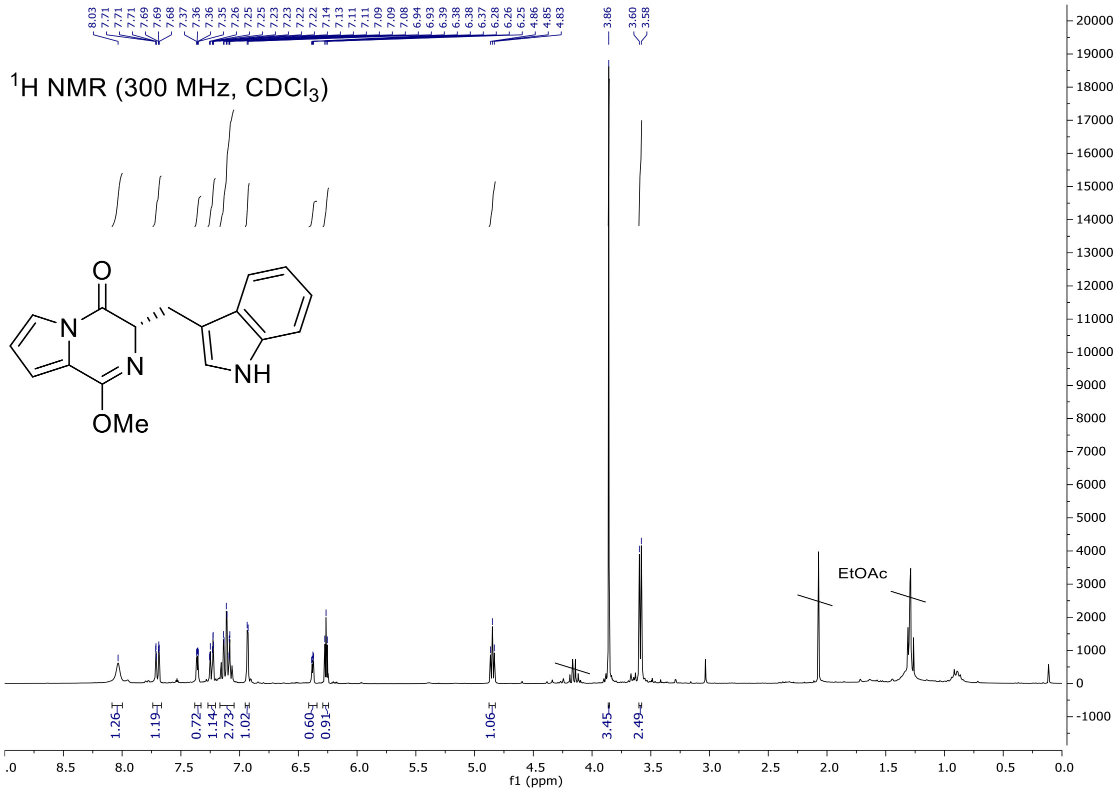

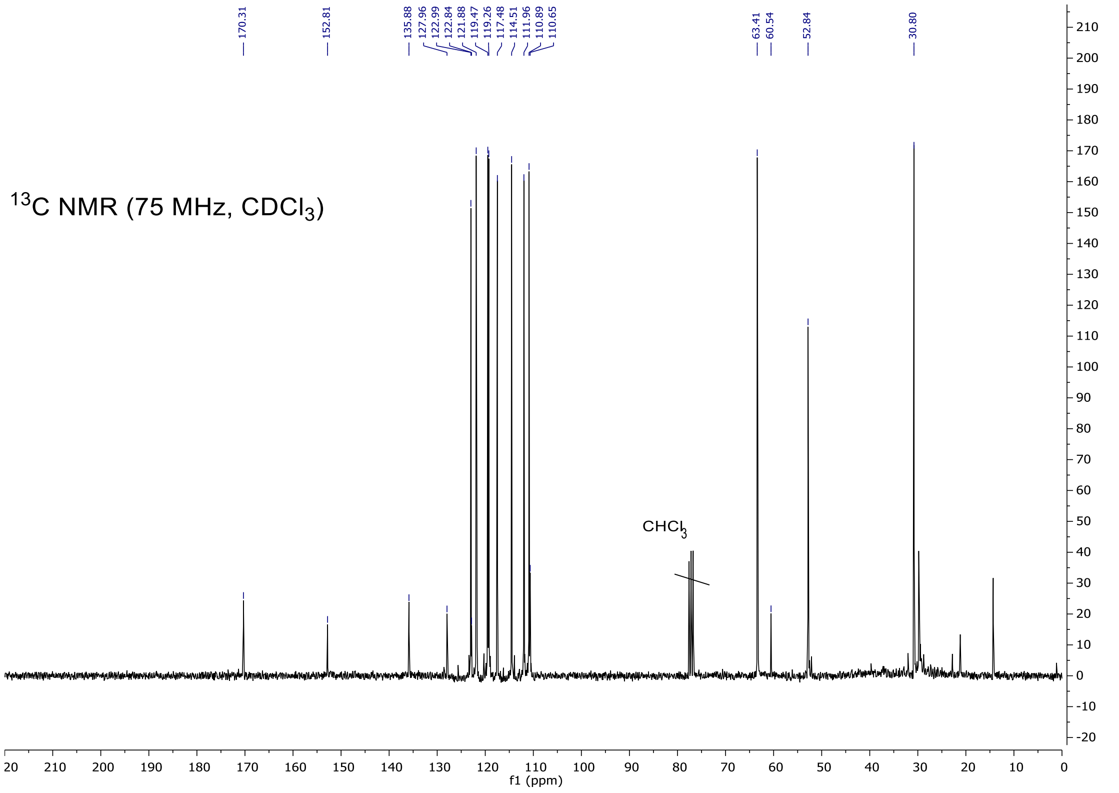

3-Allyl-1-methoxypyrrolo[1,2-*a*]pyrazin-4(3*H*)-one (1f)

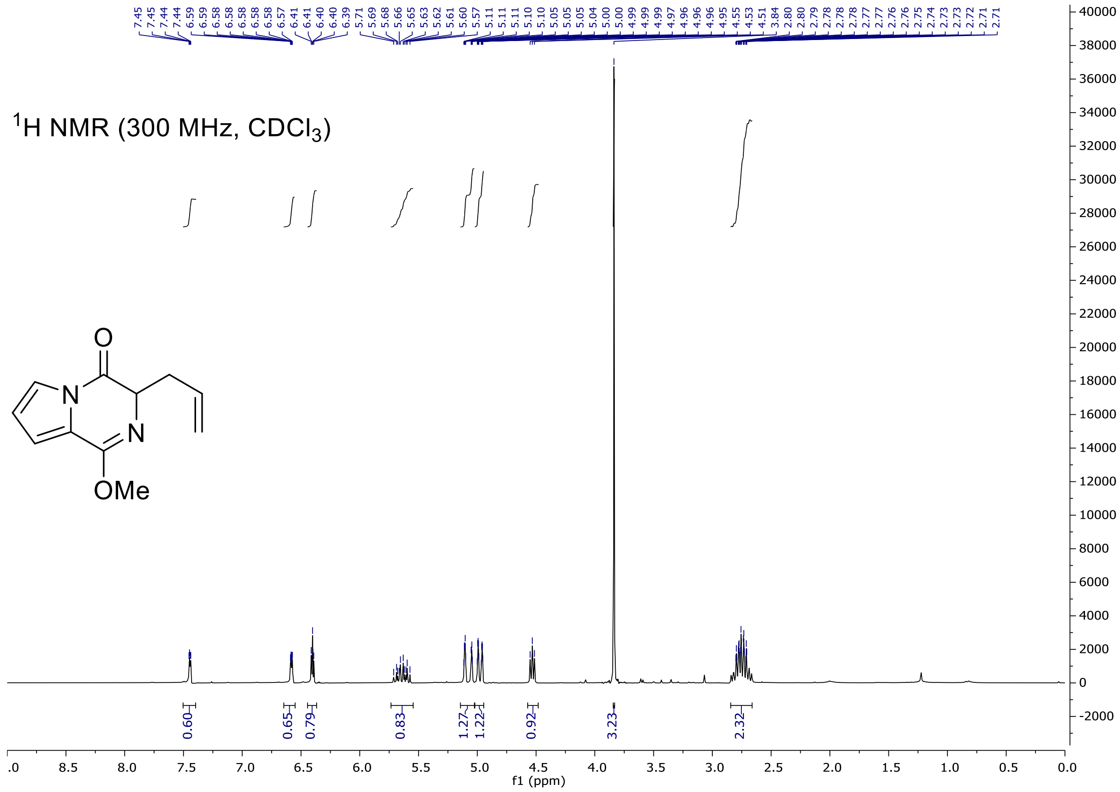

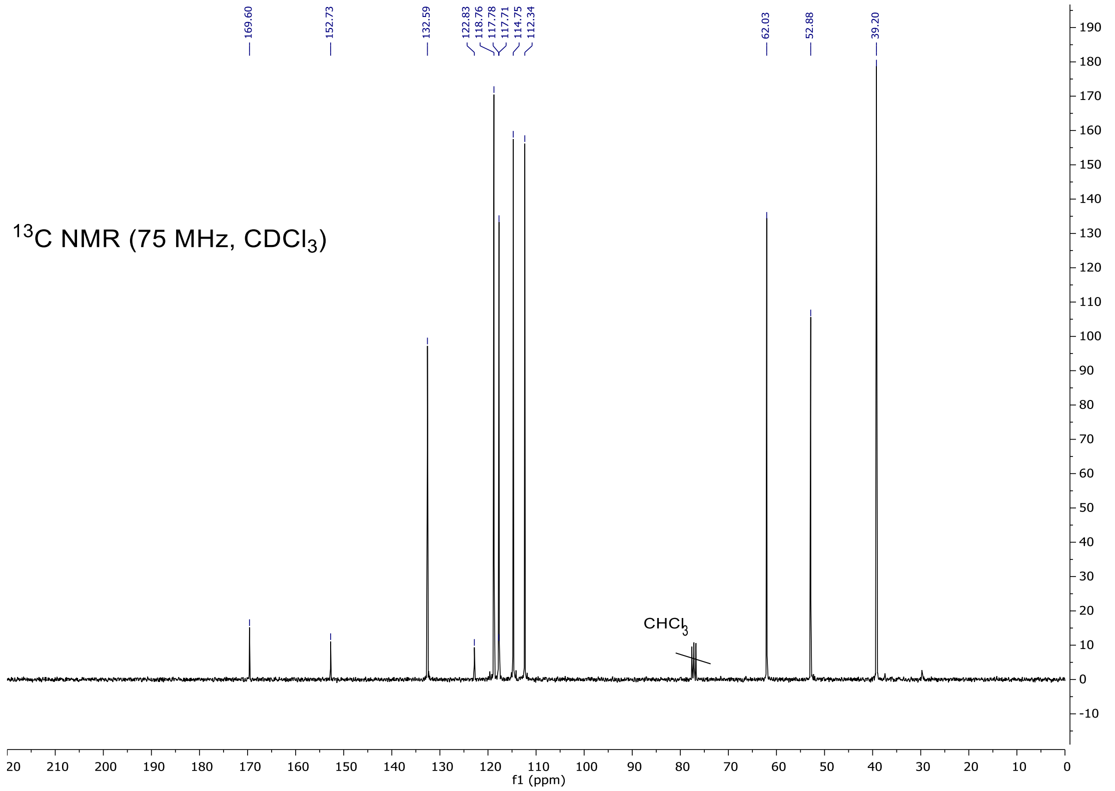

(*S*)-1-Methoxy-3-phenylpyrrolo[1,2-*a*]pyrazin-4(3*H*)-one (1g)

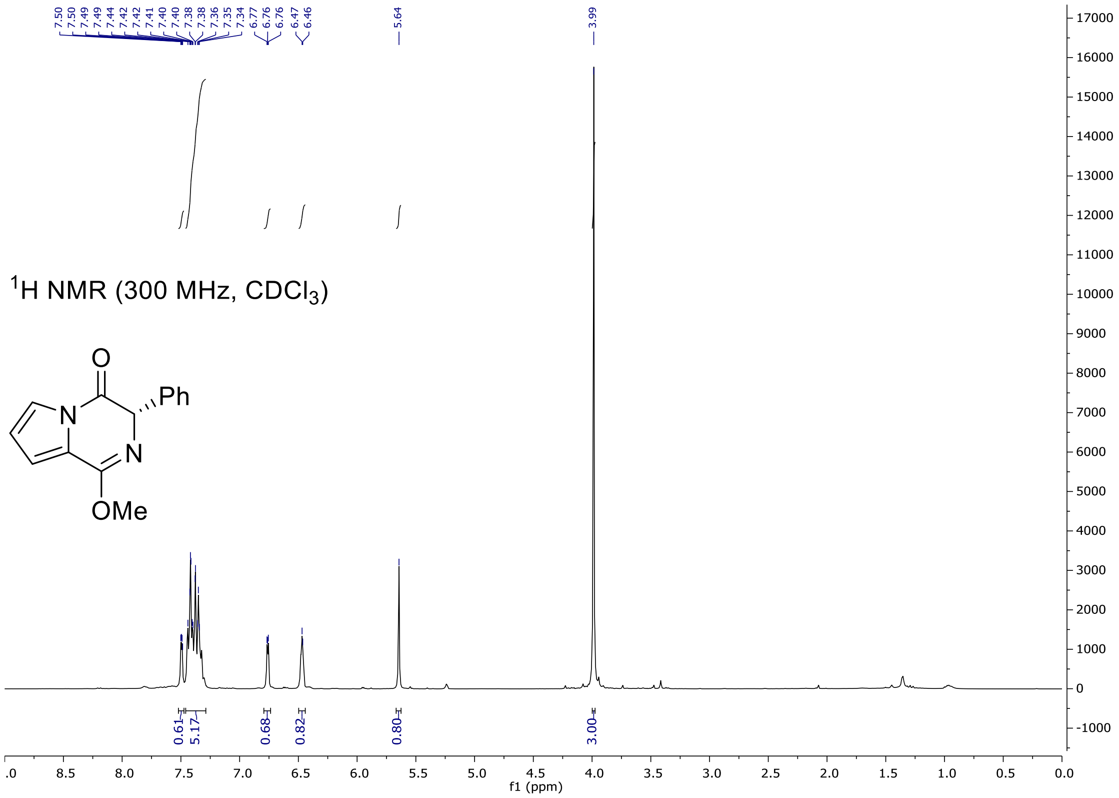

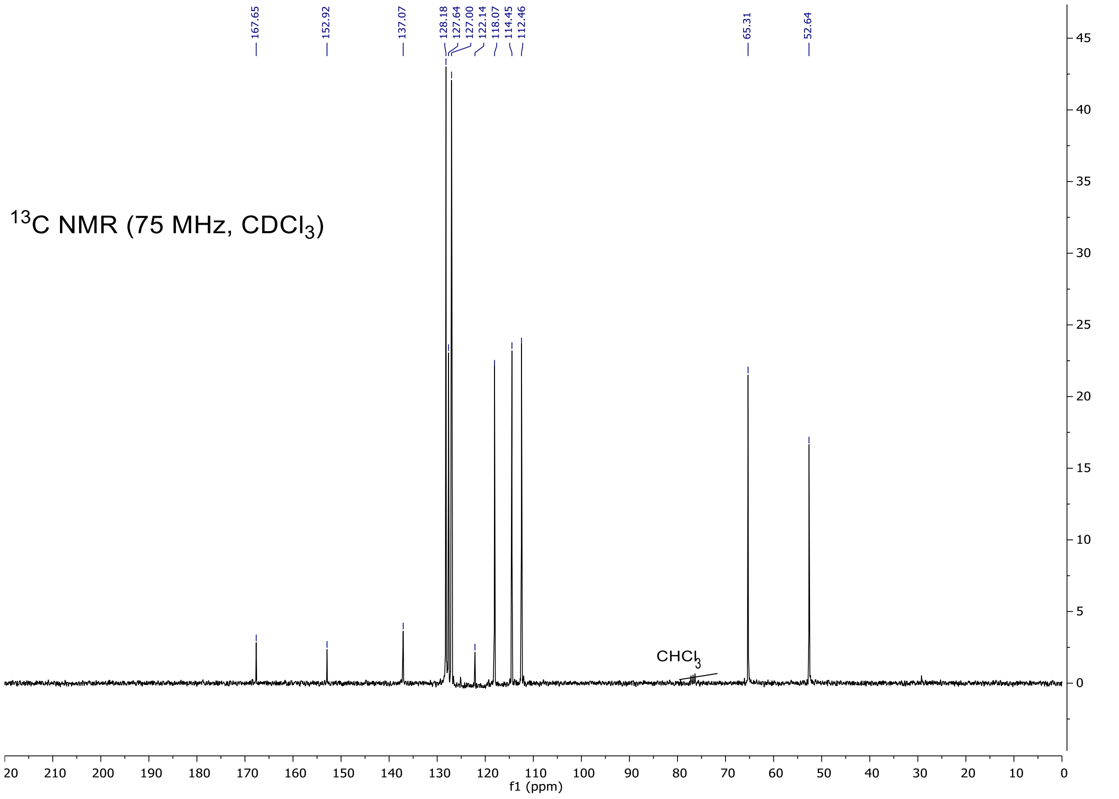

3-Hexyl-1-methoxypyrrolo[1,2-a]pyrazin-4(3H)-one (1h)

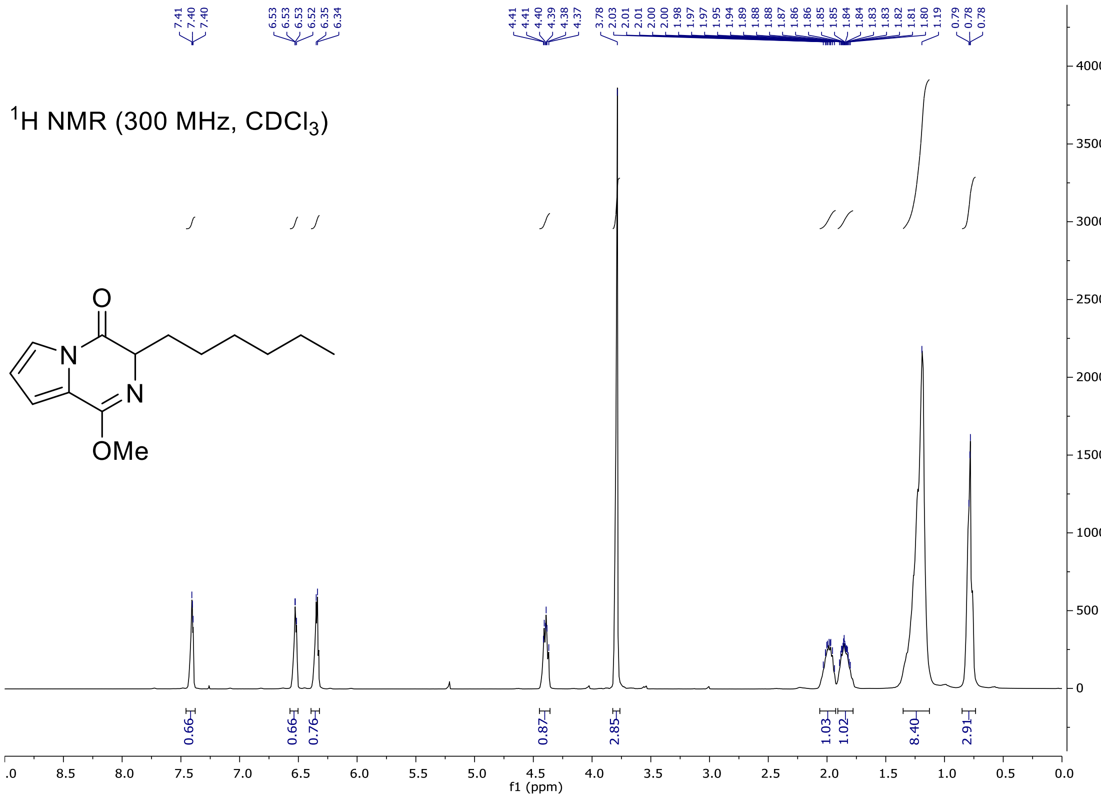

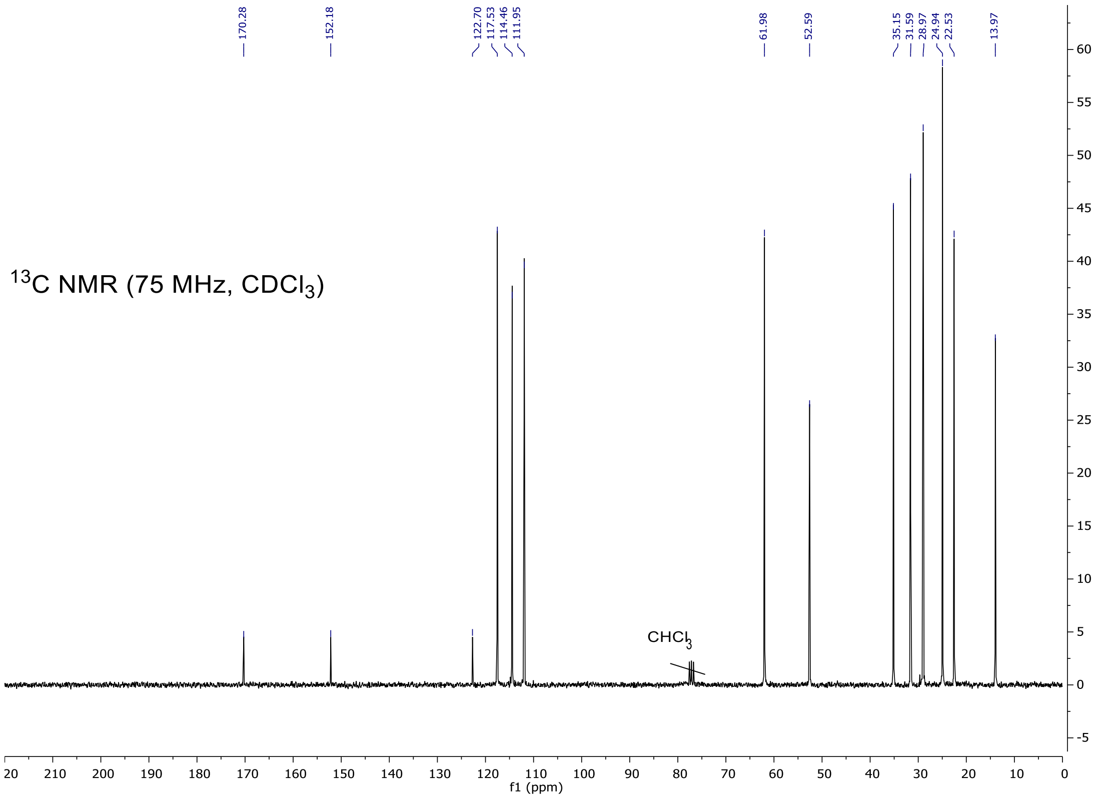

(*S*)-3-Benzyl-1-methoxy-3-((*S*)-2-nitro-1-phenylethyl)pyrrolo[1,2-*a*]pyrazin-4(3*H*)-one (3aa)

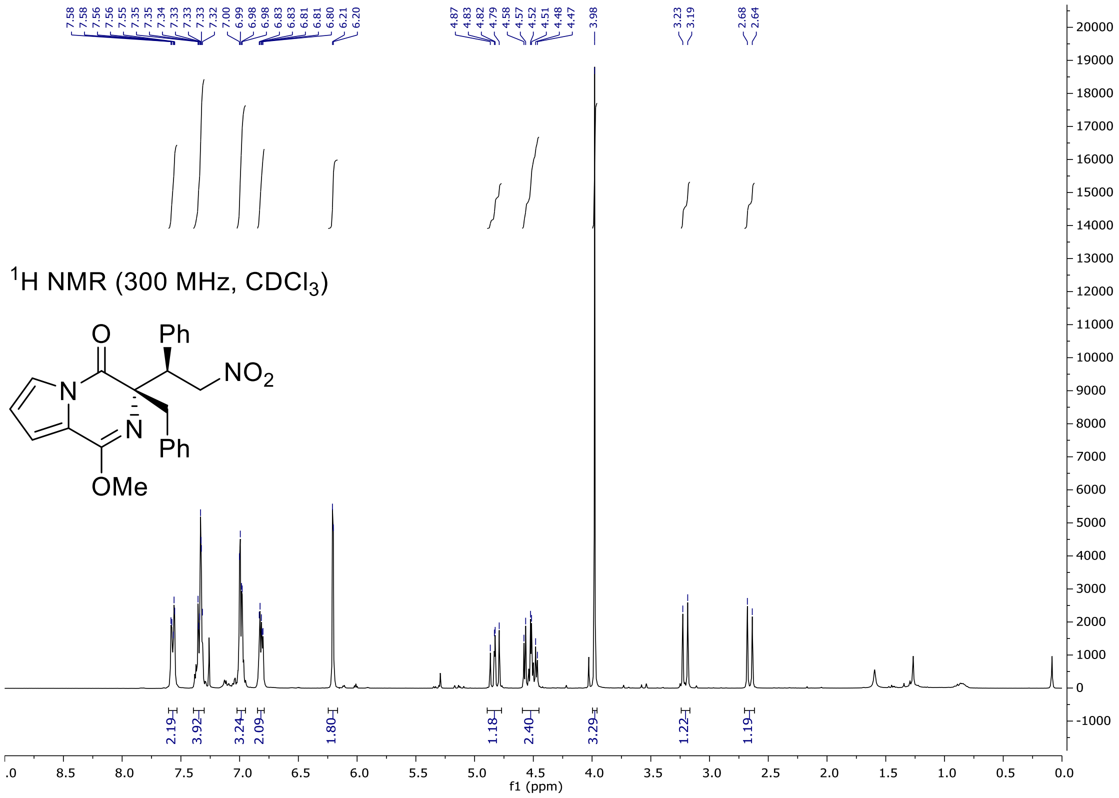

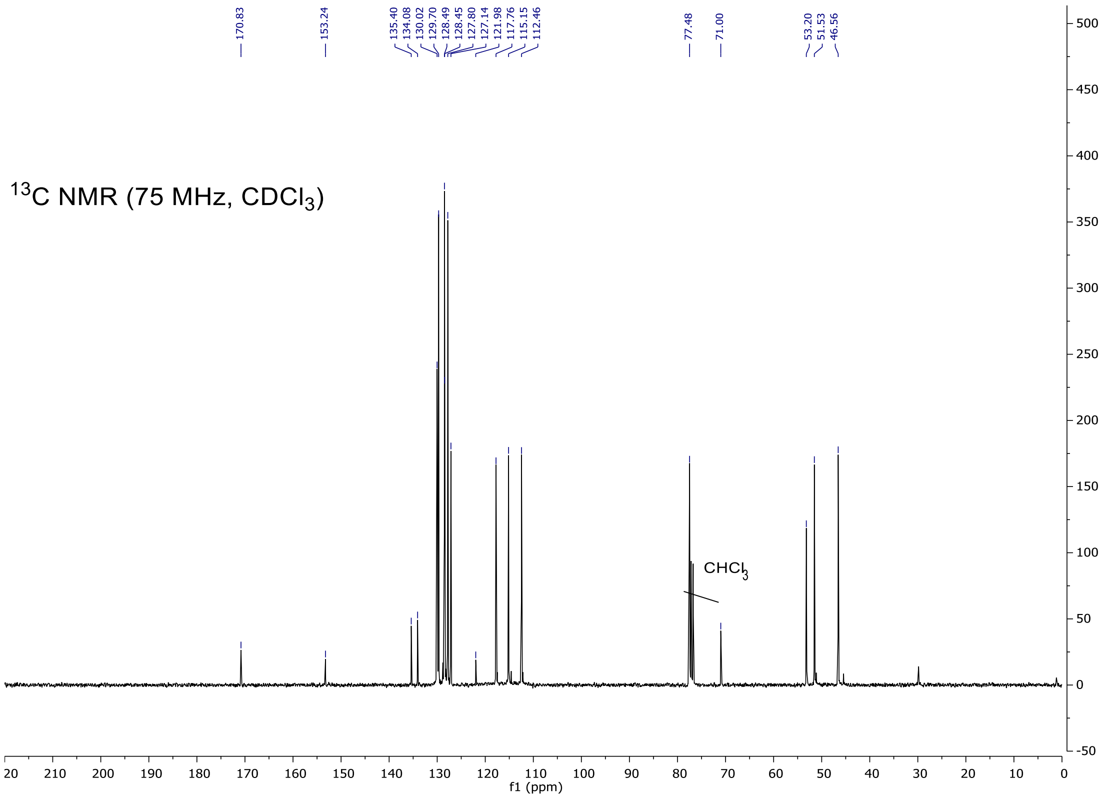

(*S*)-3-Benzyl-3-((*S*)-1-(4-bromophenyl)-2-nitroethyl)-1-methoxypyrrolo[1,2-*a*]pyrazin-4(3*H*)-one (3ab)

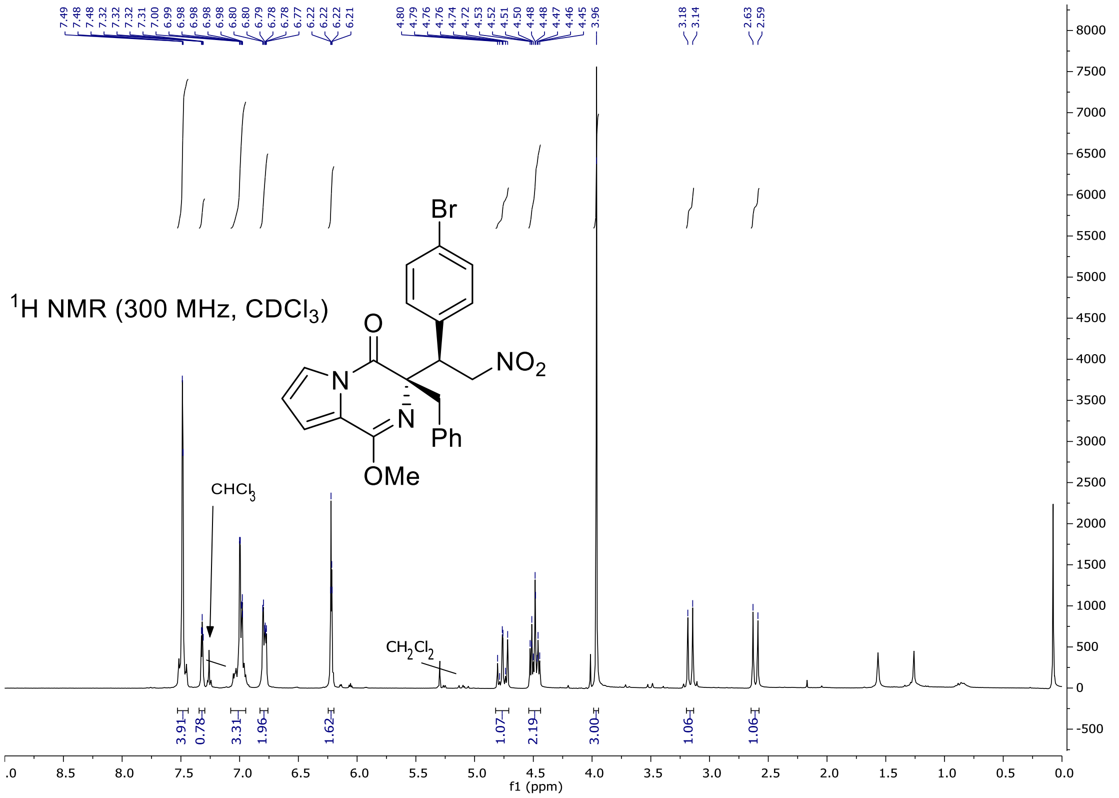

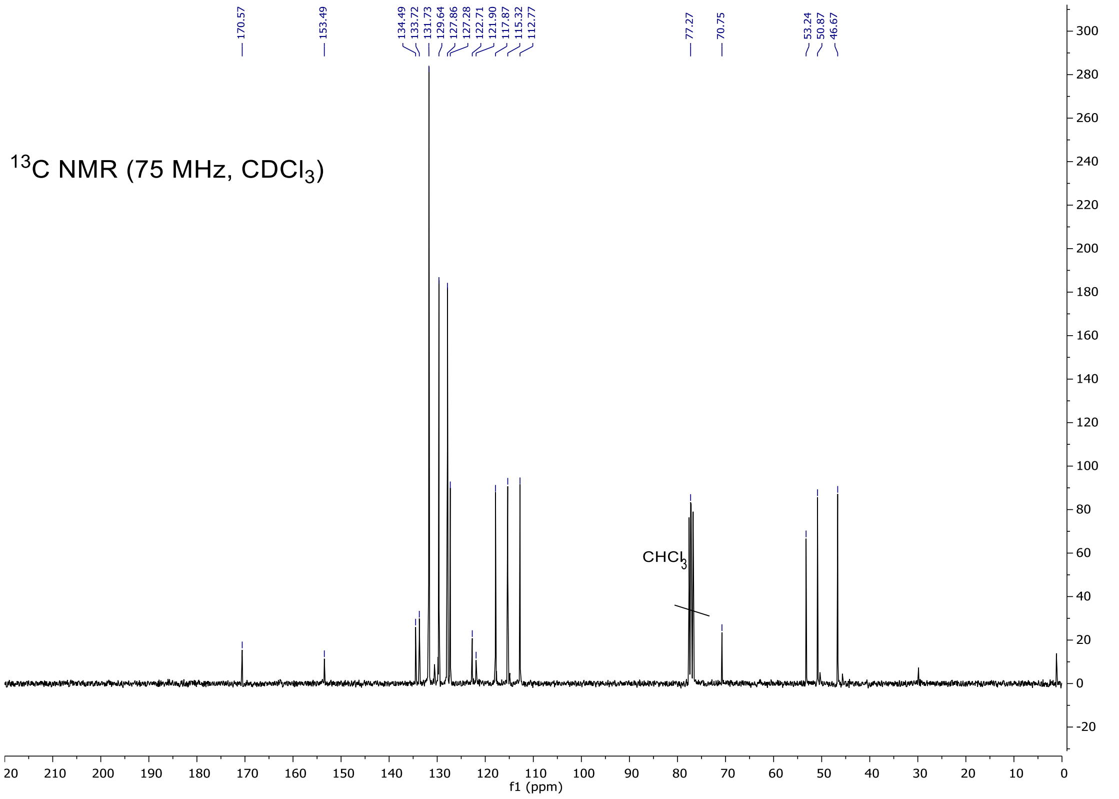

(*S*)-3-Benzyl-3-((*S*)-1-(4-fluorophenyl)-2-nitroethyl)-1-methoxypyrrolo[1,2-*a*]pyrazin-4(3*H*)-one (3ac)

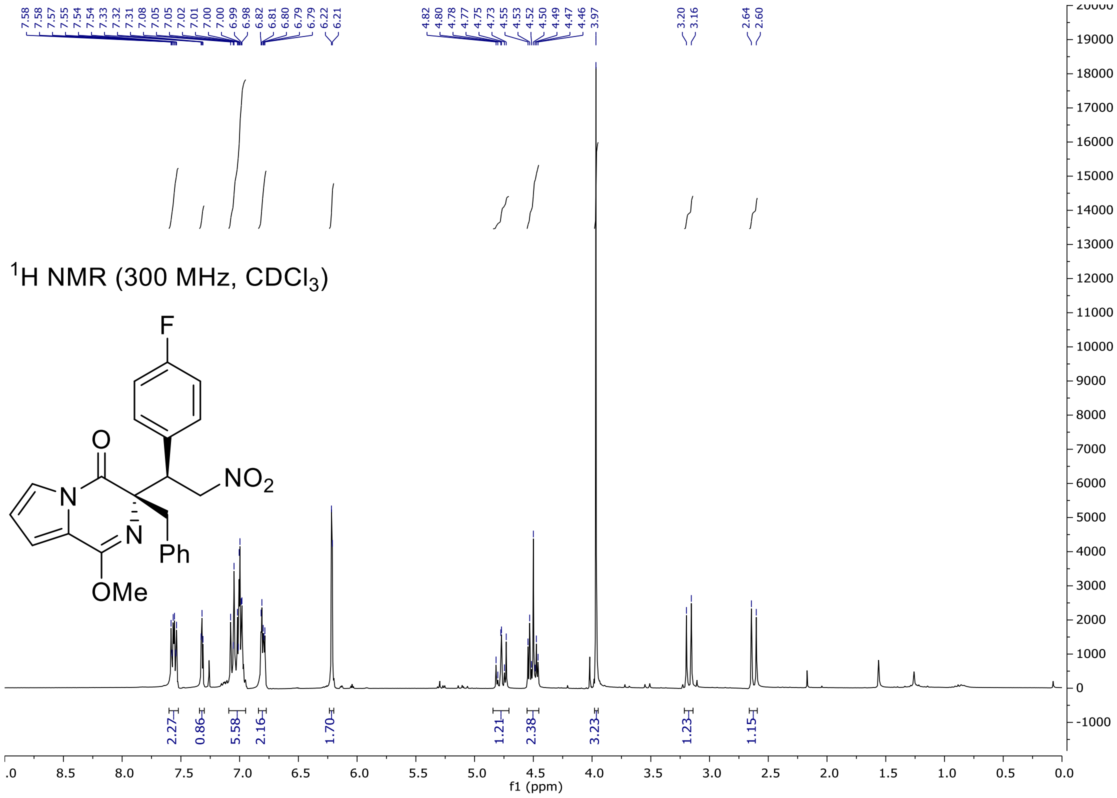

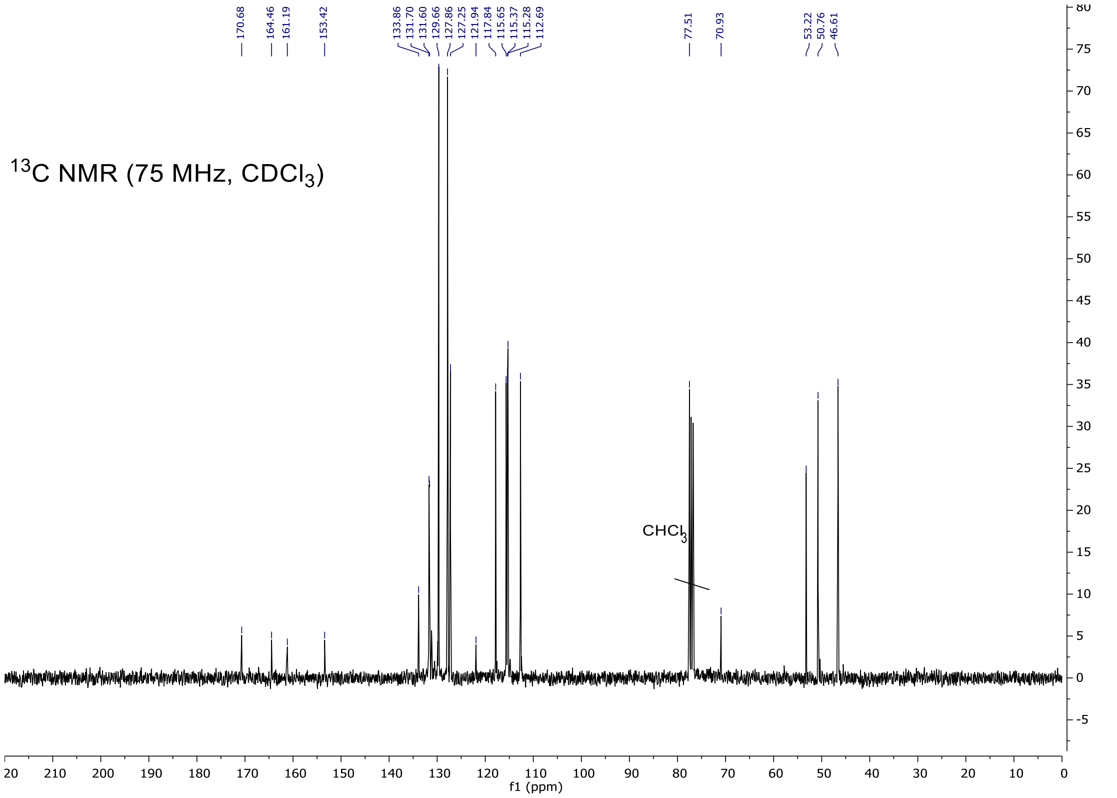

(*S*)-3-Benzyl-3-((*S*)-1-(2-chlorophenyl)-2-nitroethyl)-1-methoxypyrrolo[1,2-*a*]pyrazin-4(3*H*)-one (3ad)

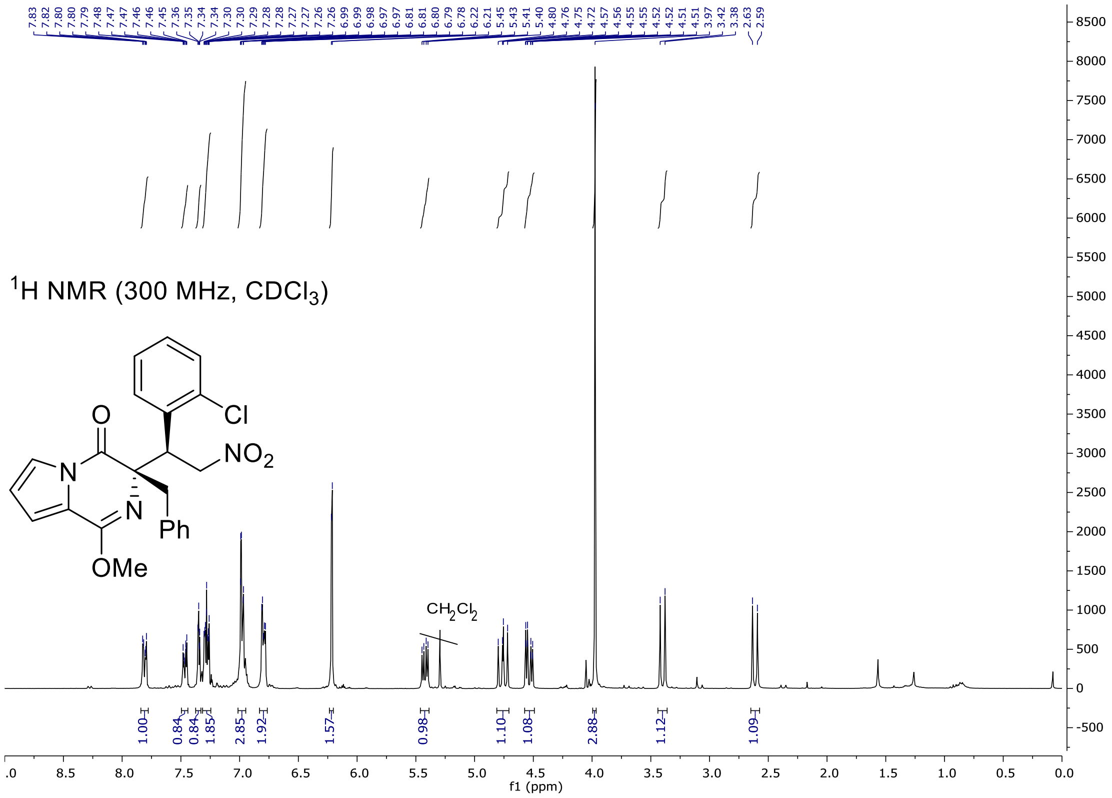

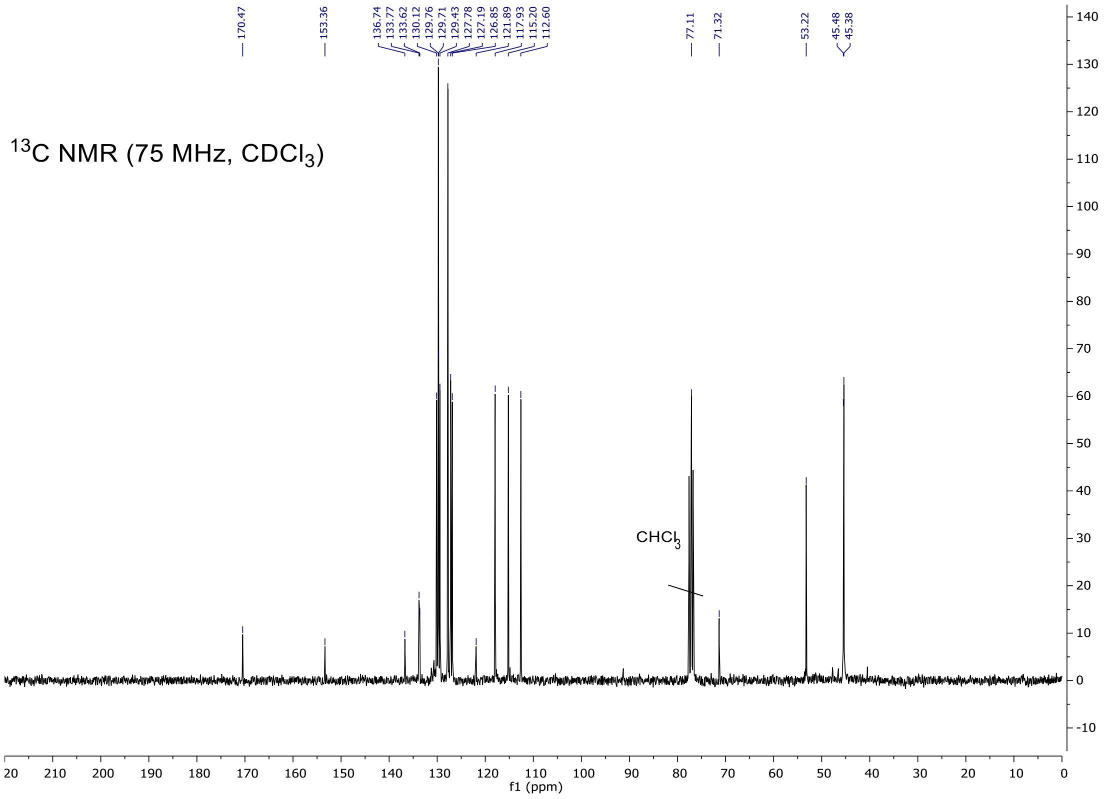

(*S*)-3-Benzyl-3-((*S*)-1-(2,4-dibromo-5-methoxyphenyl)-2-nitroethyl)-1-methoxypyrrolo[1,2-*a*]pyrazin-4(3*H*)-one (3ae)

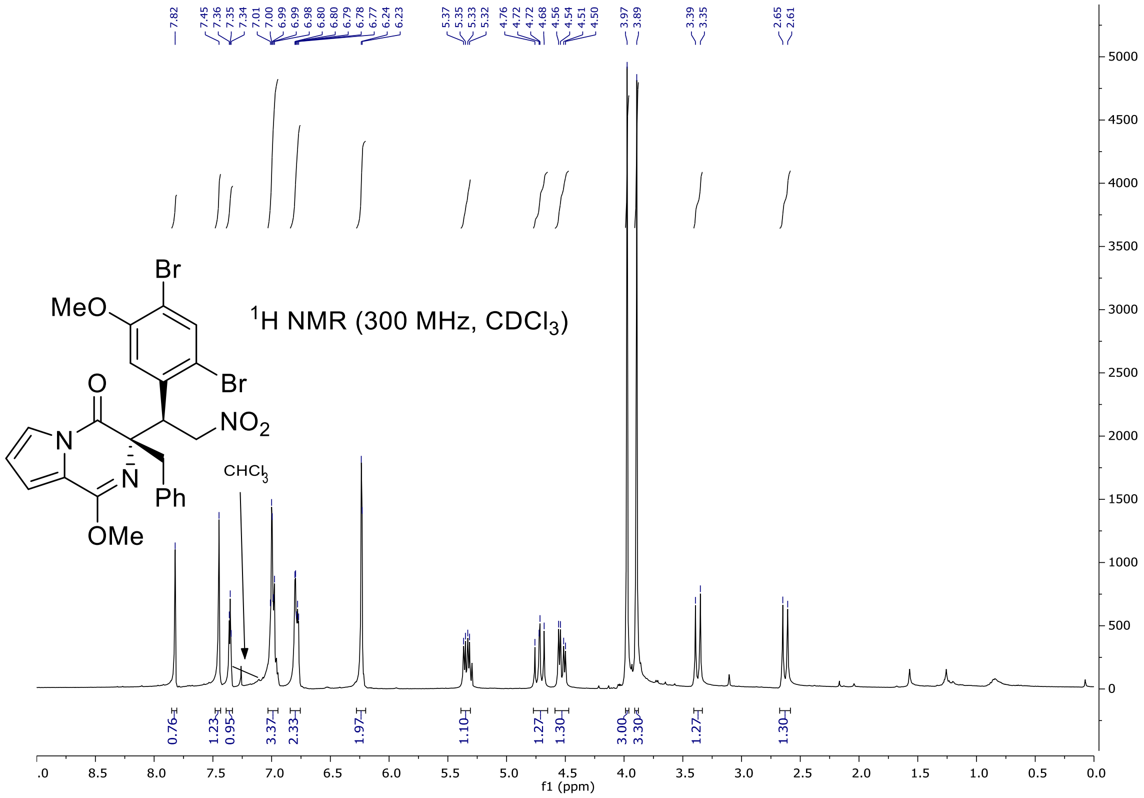

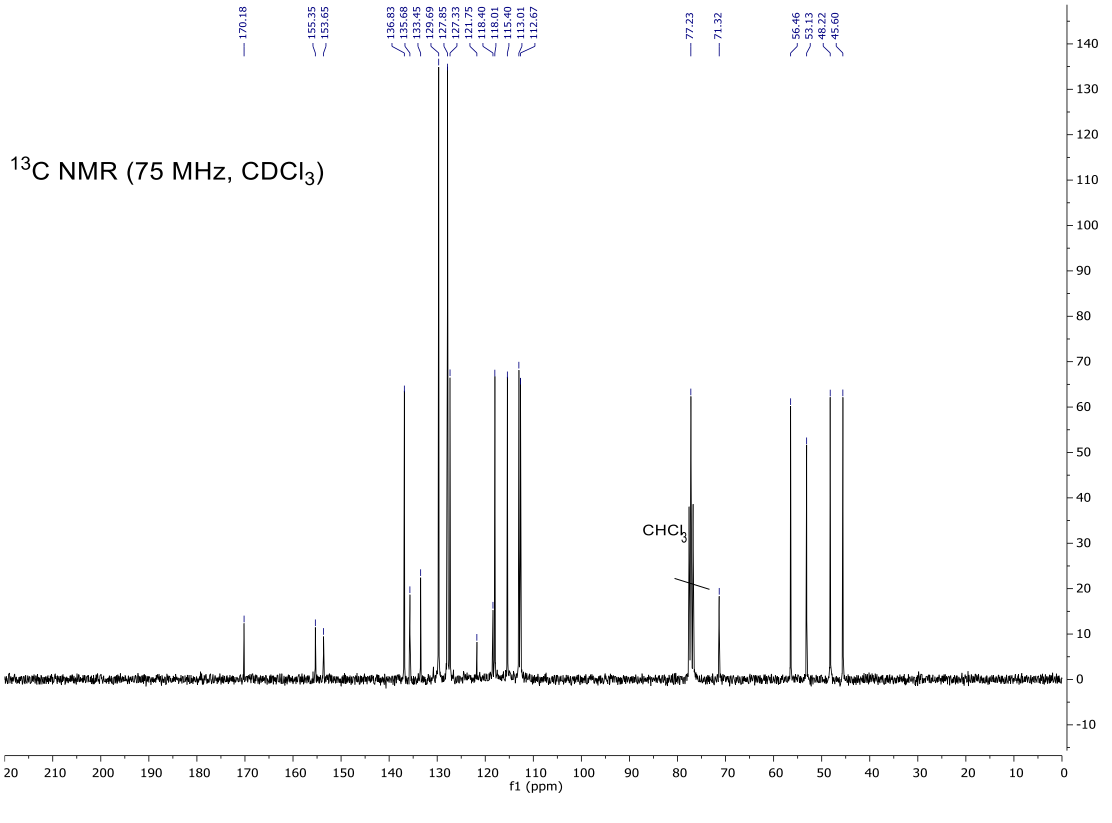

(*S*)-3-Benzyl-3-((*S*)-1-(furan-2-yl)-2-nitroethyl)-1-methoxypyrrolo[1,2-*a*]pyrazin-4(3*H*)-one (3af)

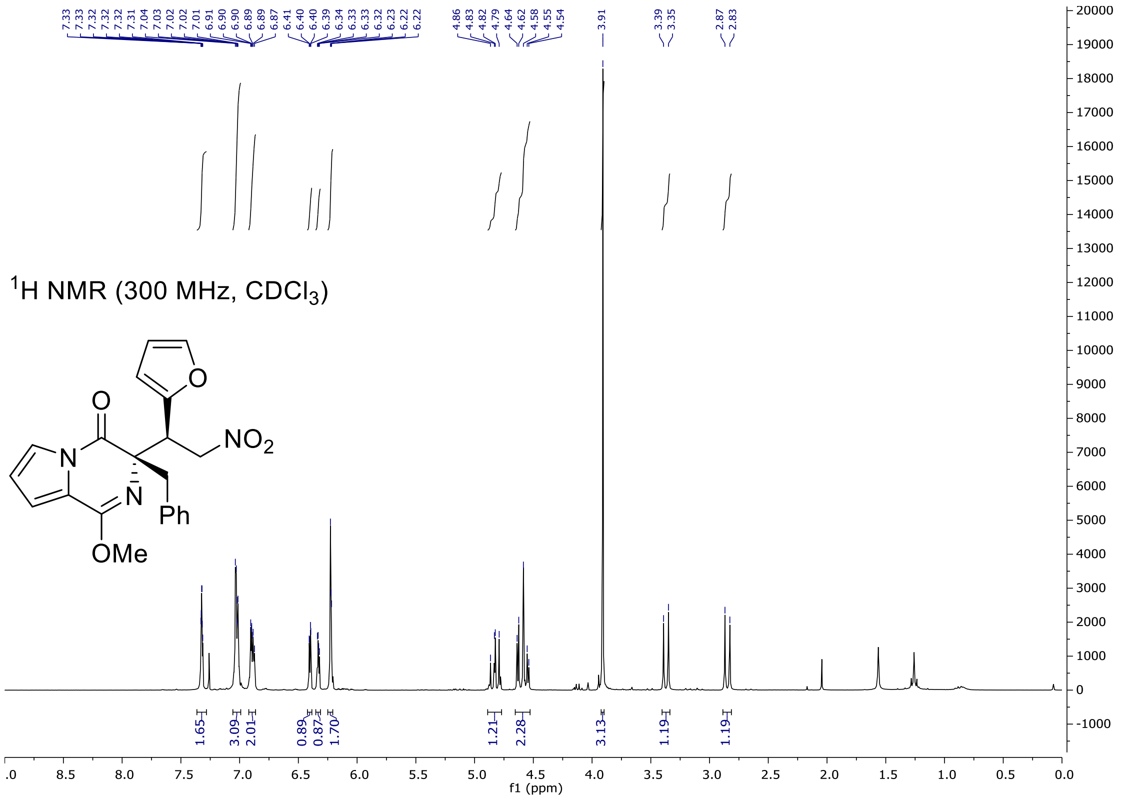

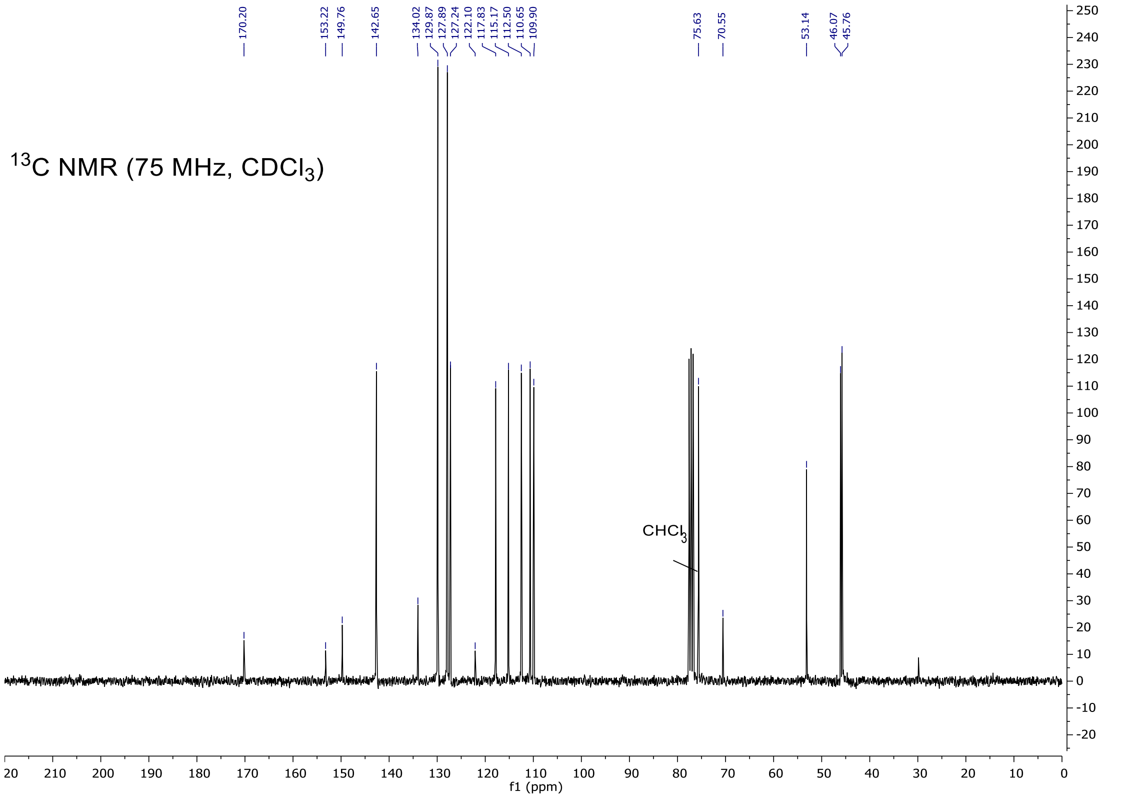

(*S*)-3-Benzyl-3-((*S*)-1-(furan-3-yl)-2-nitroethyl)-1-methoxypyrrolo[1,2-*a*]pyrazin-4(3*H*)-one (3ag)

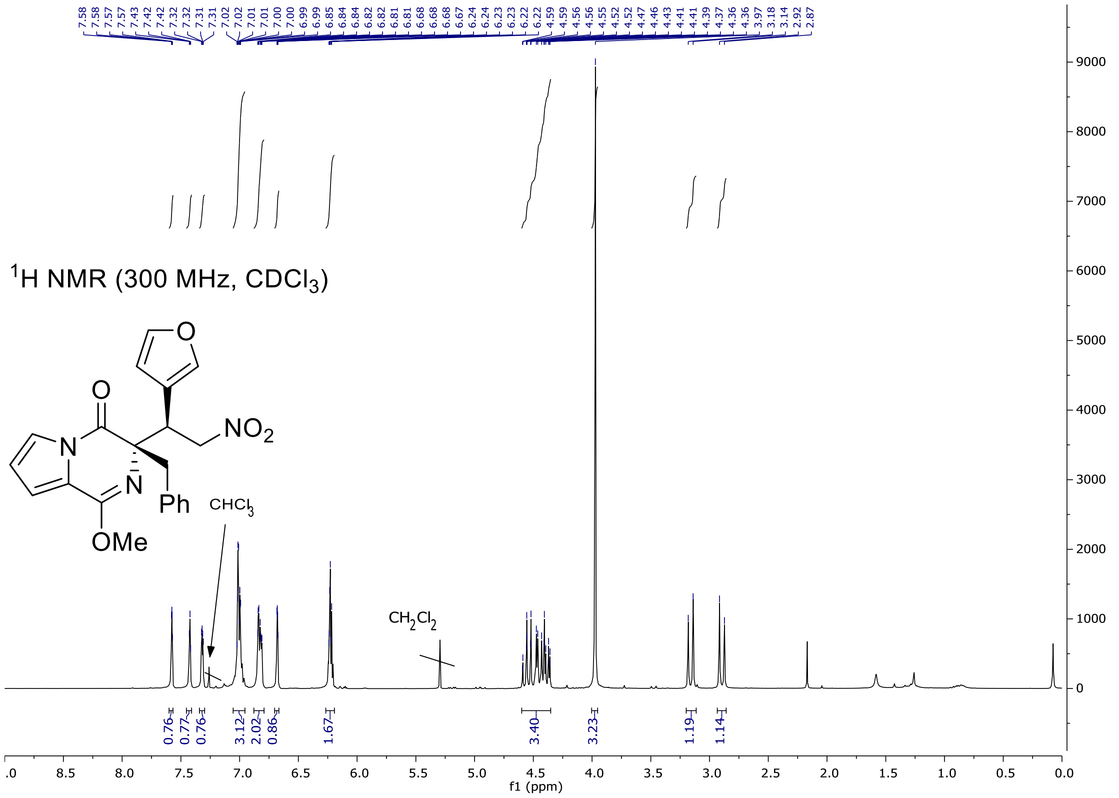

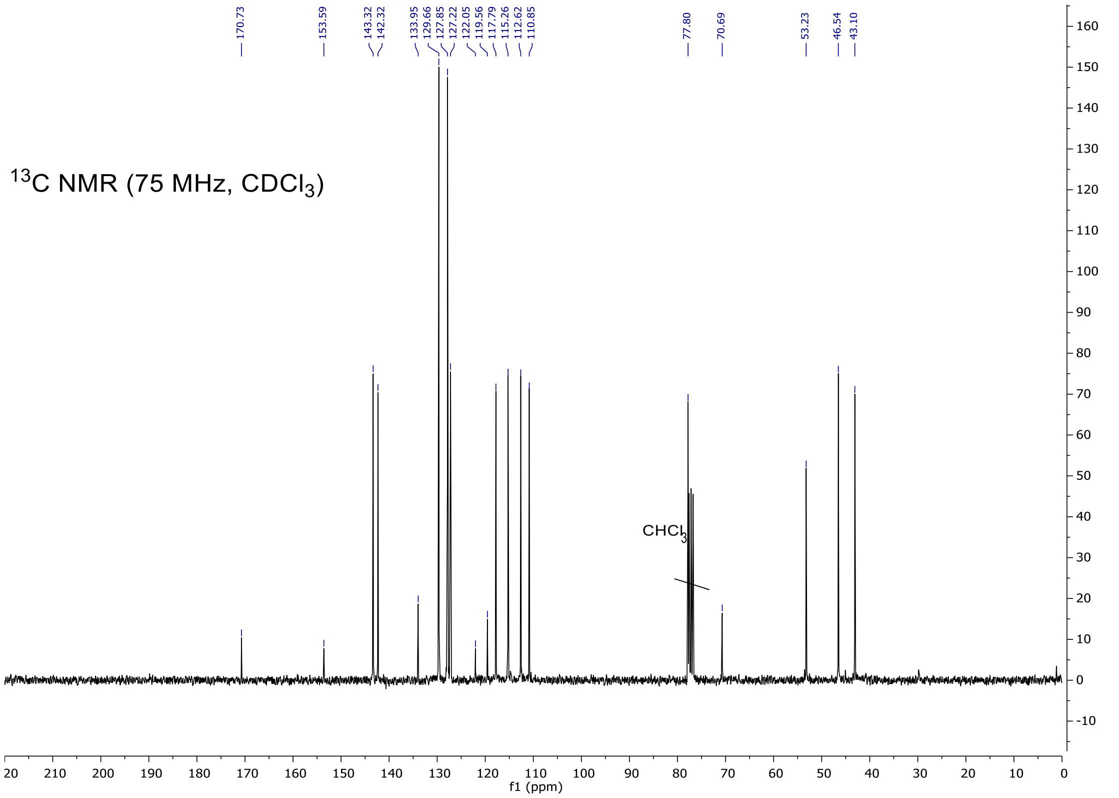

(*S*)-3-Benzyl-1-methoxy-3-((*S*)-2-nitro-1-(thiophen-2-yl)ethyl)pyrrolo[1,2-*a*]pyrazin-4(3*H*)-one (3ah)

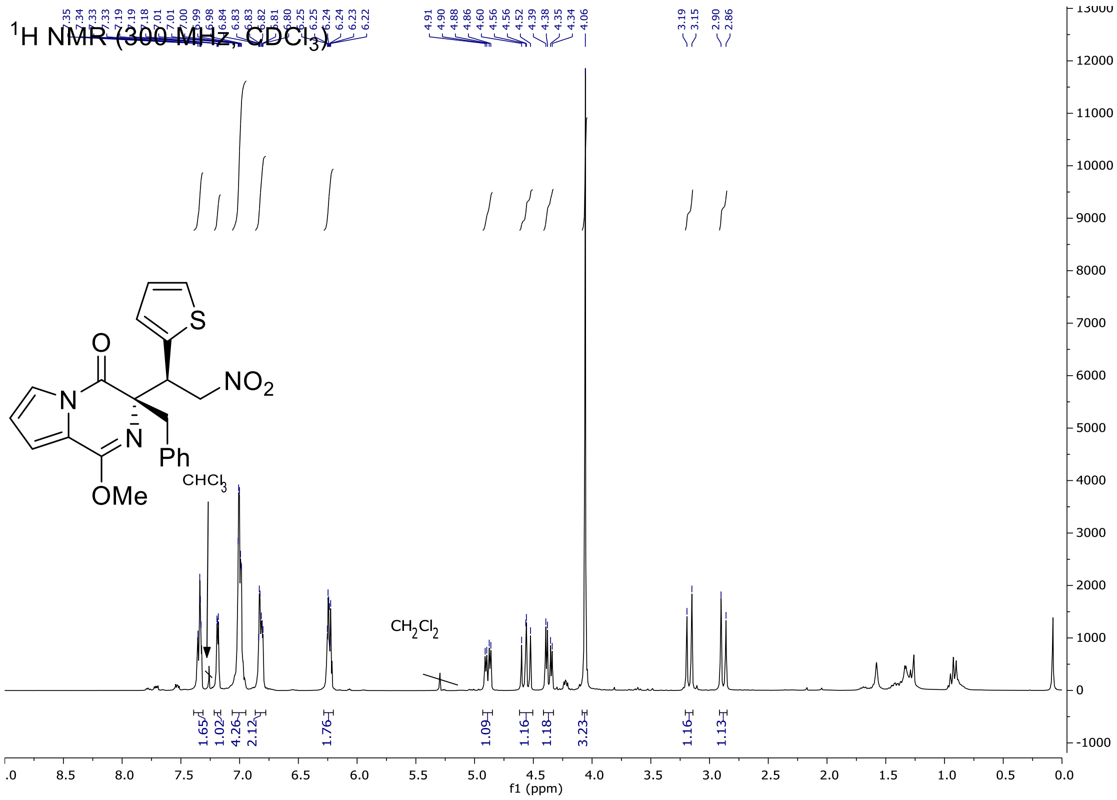

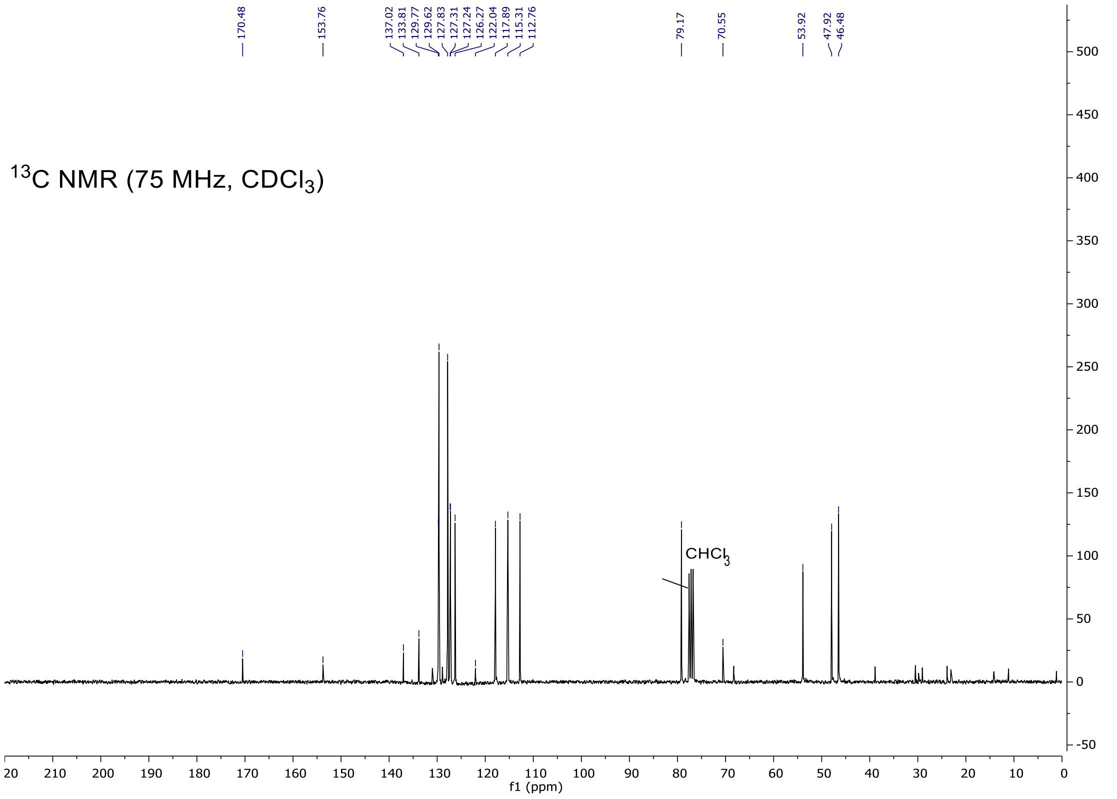

(*S*)-3-Benzyl-3-((*S*)-1-cyclohexyl-2-nitroethyl)-1-methoxypyrrolo[1,2-a]pyrazin-4(3H)-one (3ai)

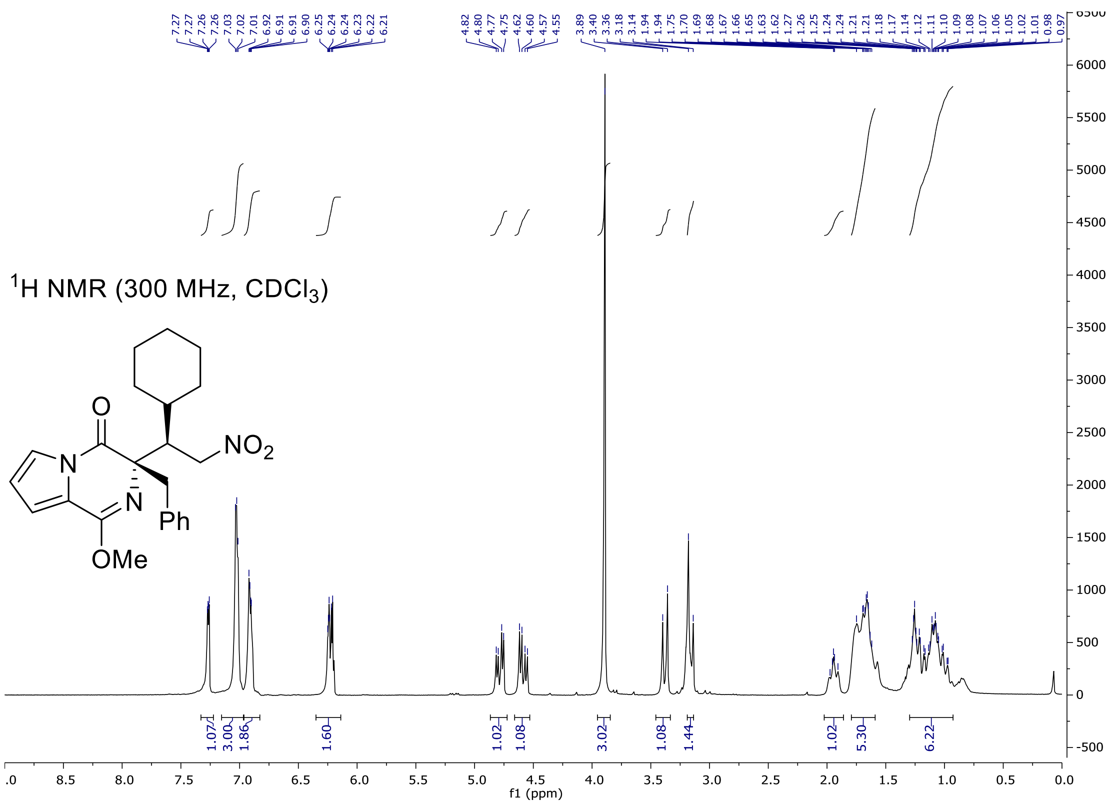

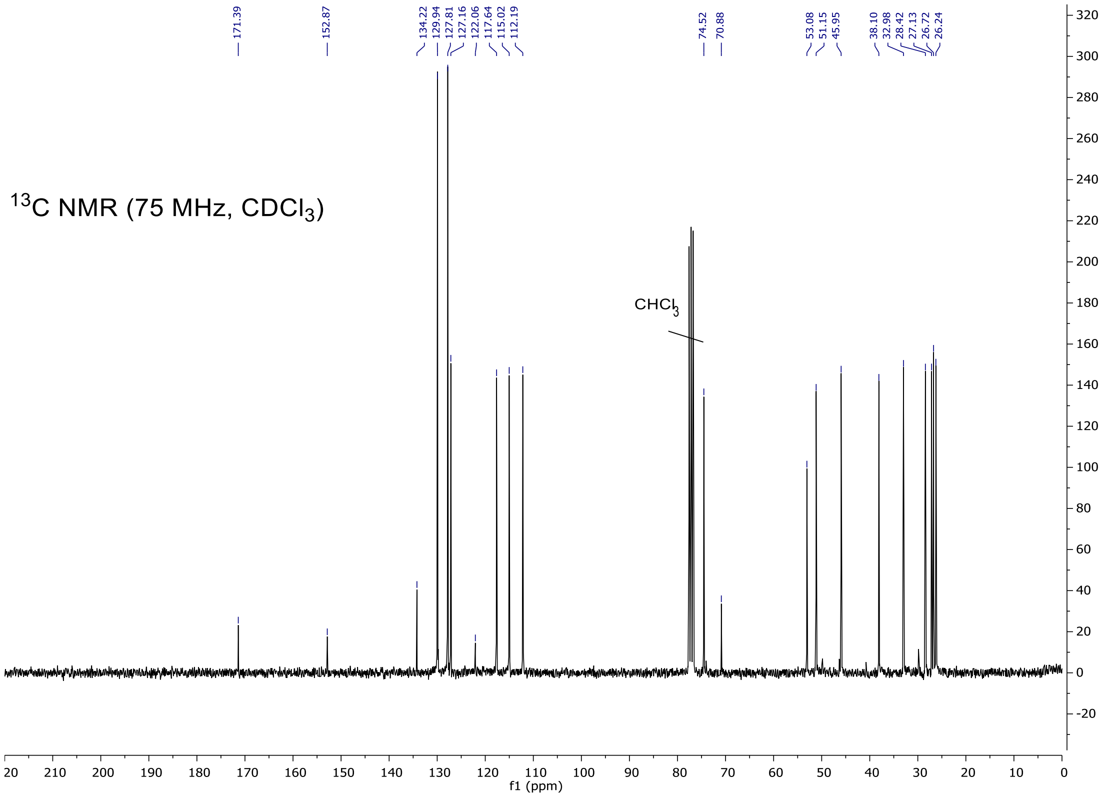

(*S*)-3-Isobutyl-1-methoxy-3-((*S*)-2-nitro-1-phenylethyl)pyrrolo[1,2-*a*]pyrazin-4(3*H*)-one (3ba)

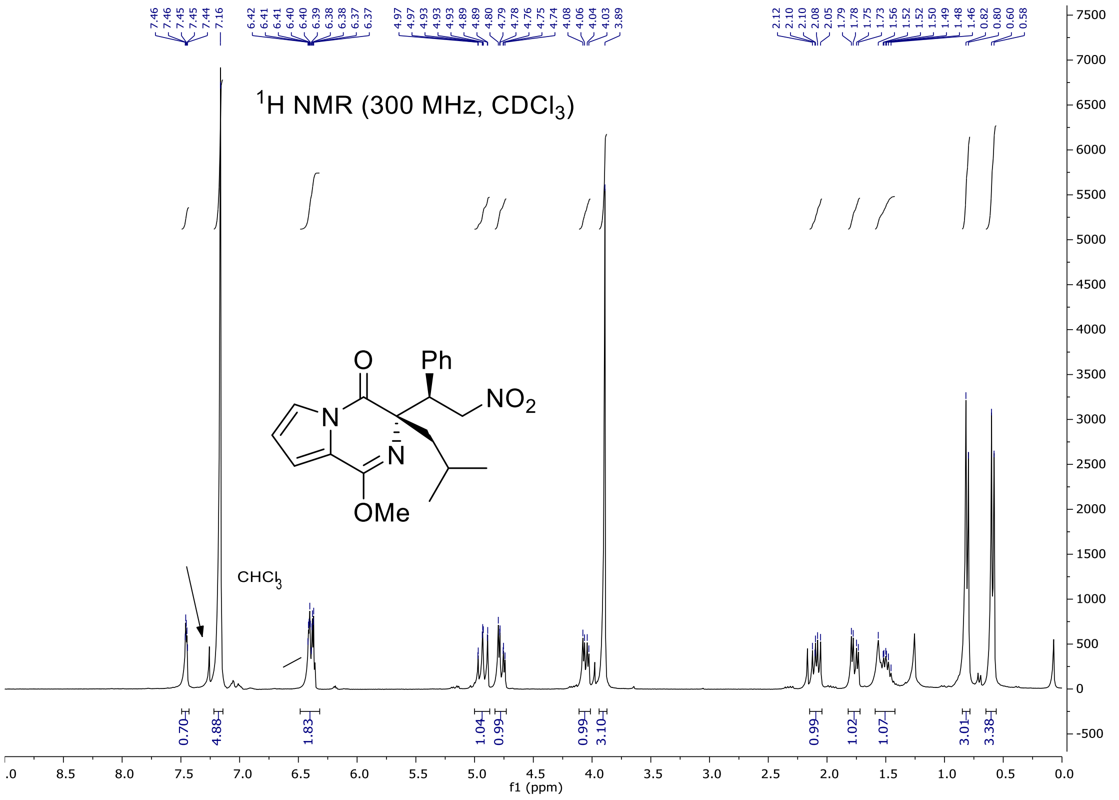

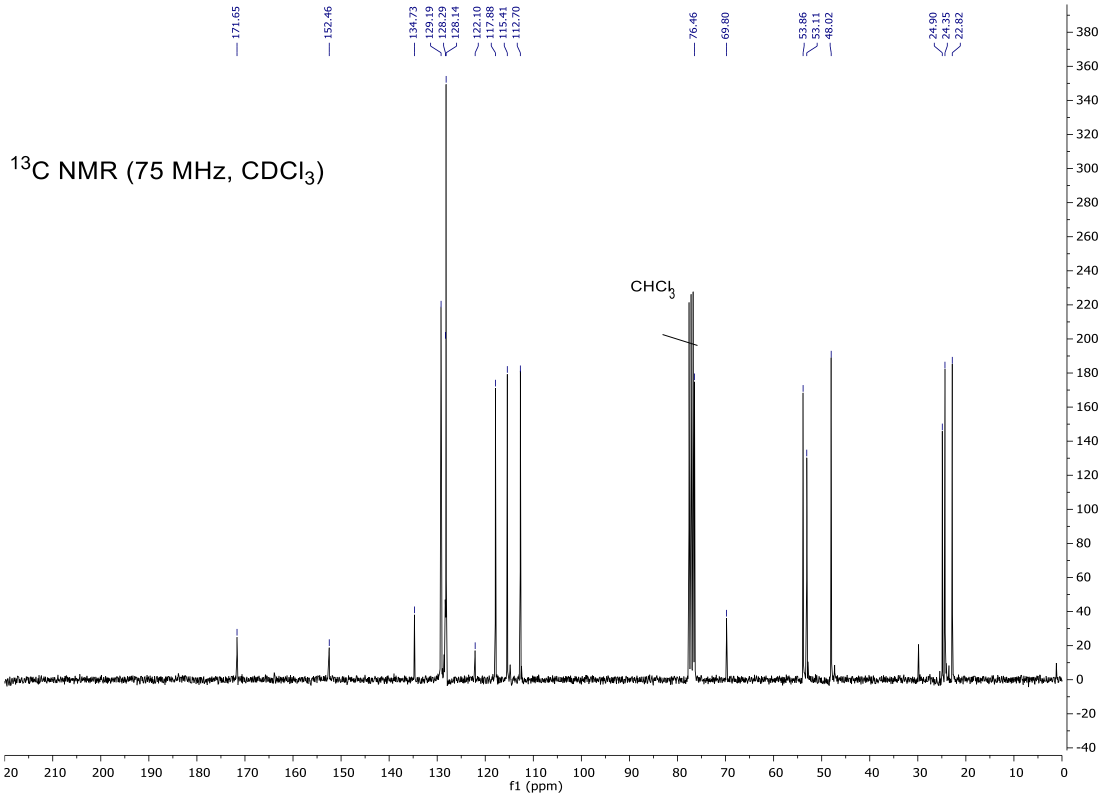

(*S*)-3-((*S*)-1-(4-Bromophenyl)-2-nitroethyl)-3-isobutyl-1-methoxypyrrolo[1,2-*a*]pyrazin-4(3*H*)-one (3bb)

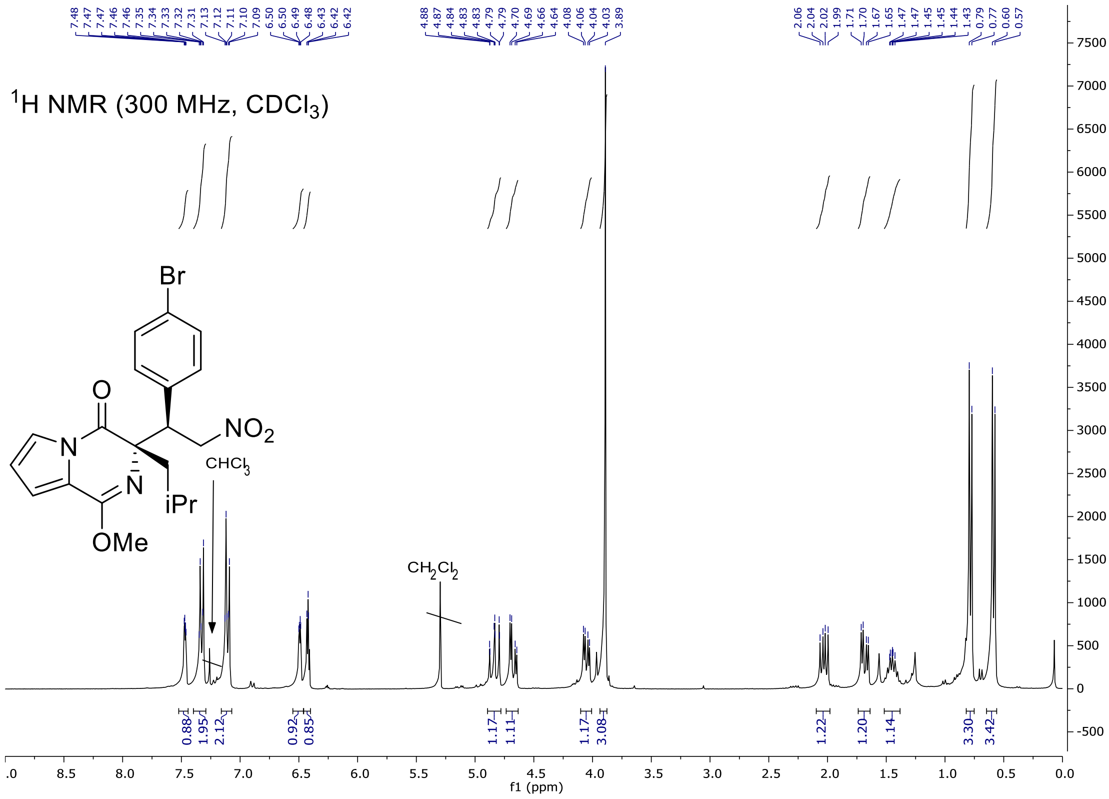

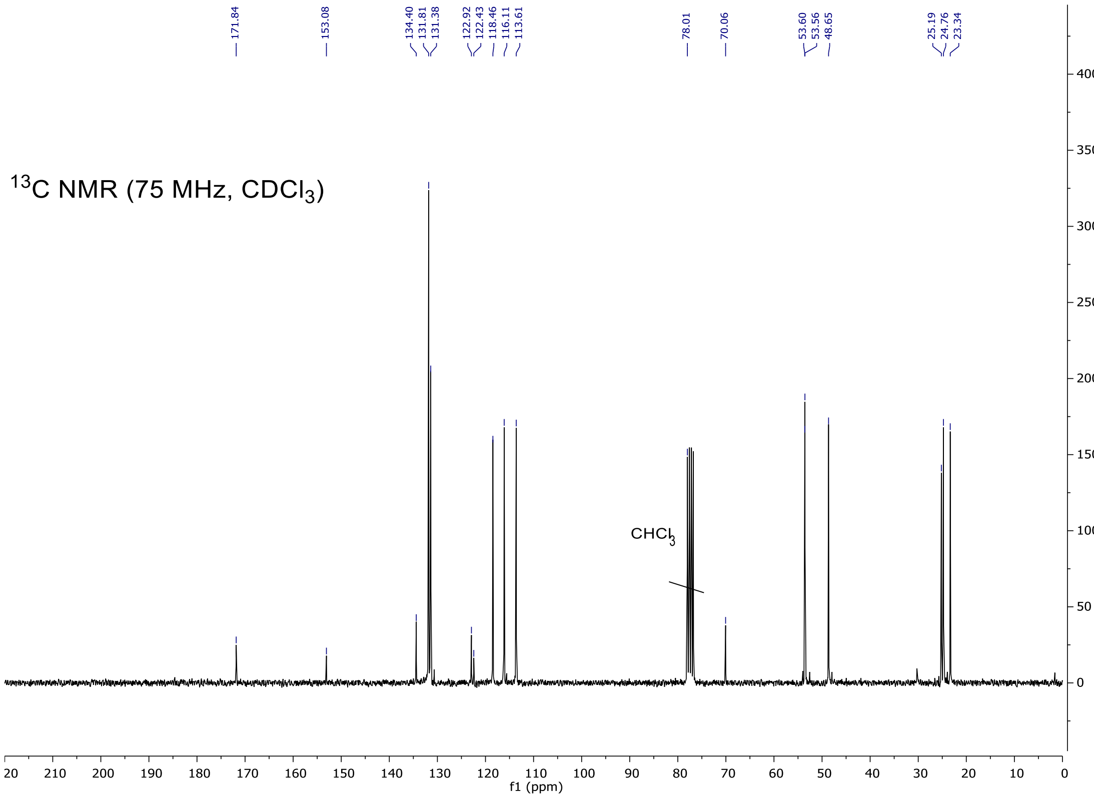

(*S*)-3-((*S*)-1-(Furan-2-yl)-2-nitroethyl)-3-isobutyl-1-methoxypyrrolo[1,2-*a*]pyrazin-4(3*H*)-one (3bf)

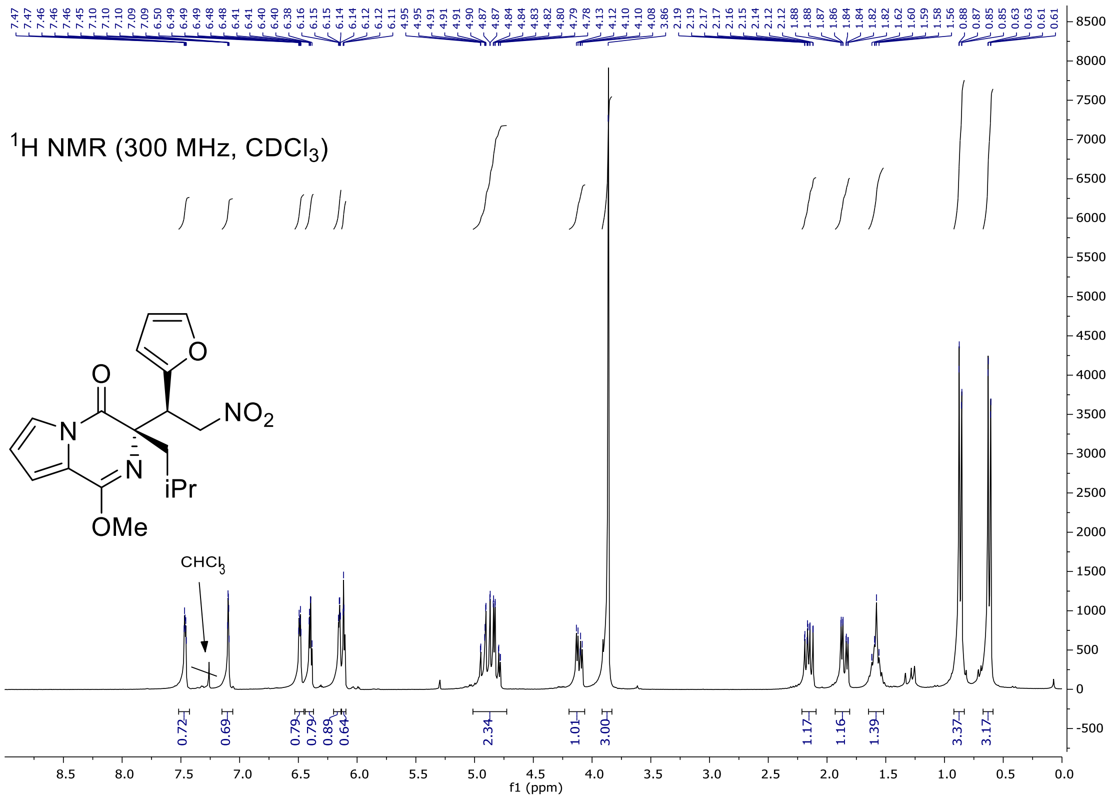

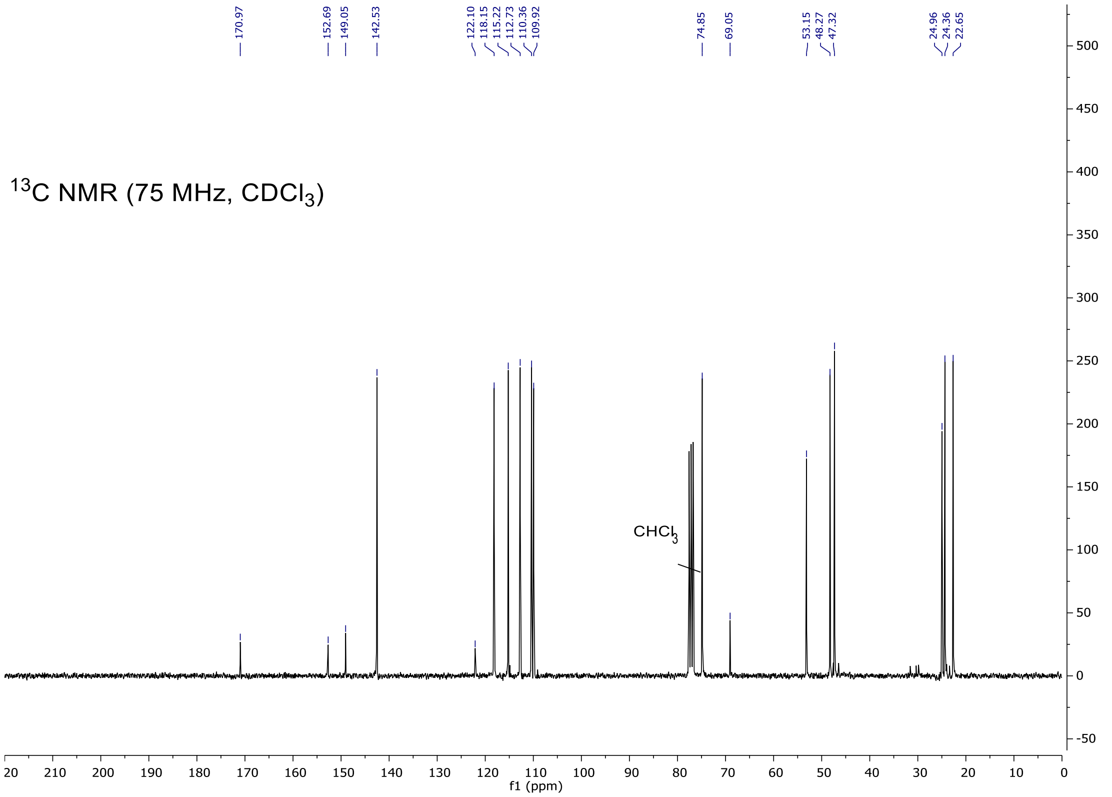

(*S*)-1-Methoxy-3-(4-methoxybenzyl)-3-((*S*)-2-nitro-1-phenylethyl)pyrrolo[1,2-*a*]pyrazin-4(3*H*)-one (3ca)

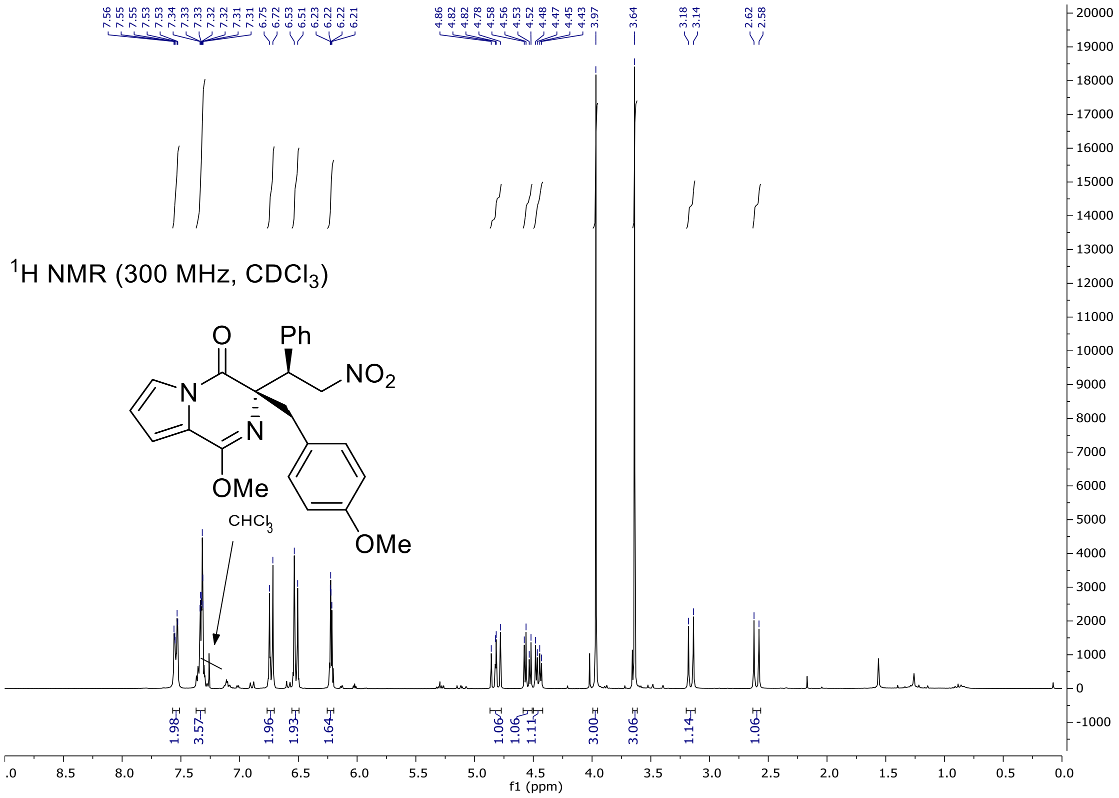

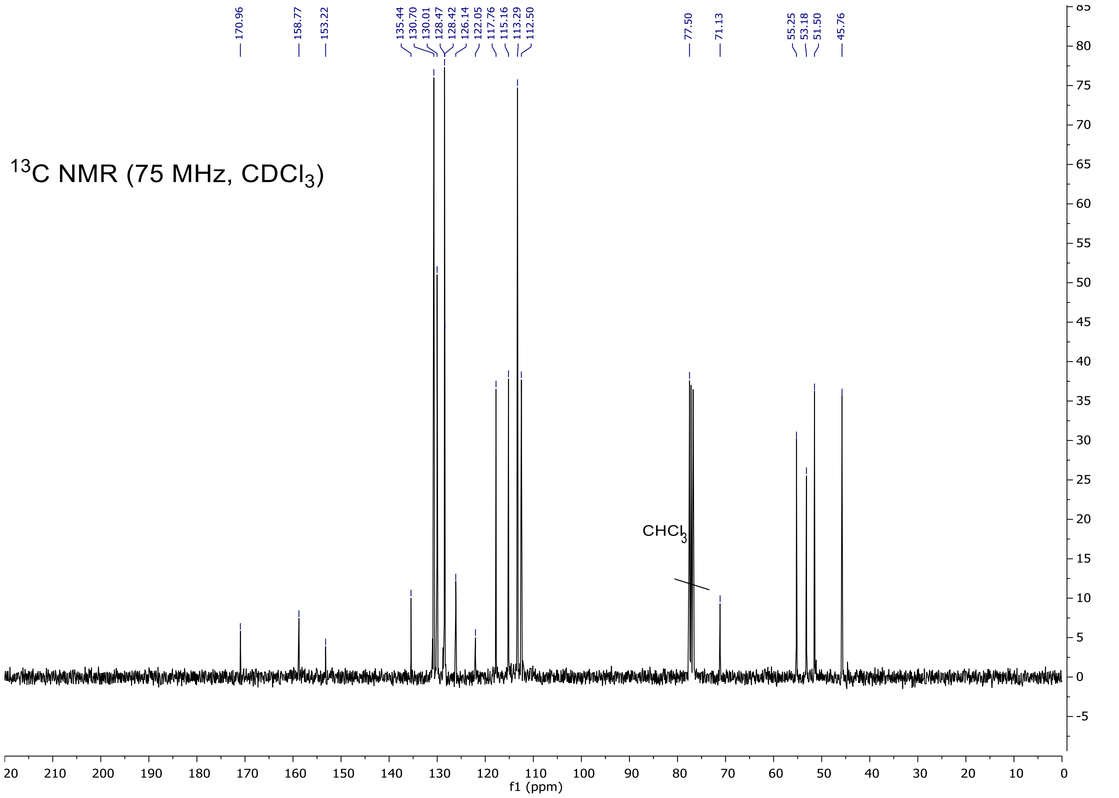

(*S*)-3-((*S*)-1-(Furan-2-yl)-2-nitroethyl)-1-methoxy-3-(4-methoxybenzyl)pyrrolo[1,2-*a*]pyrazin-4(3*H*)-one (3cf)

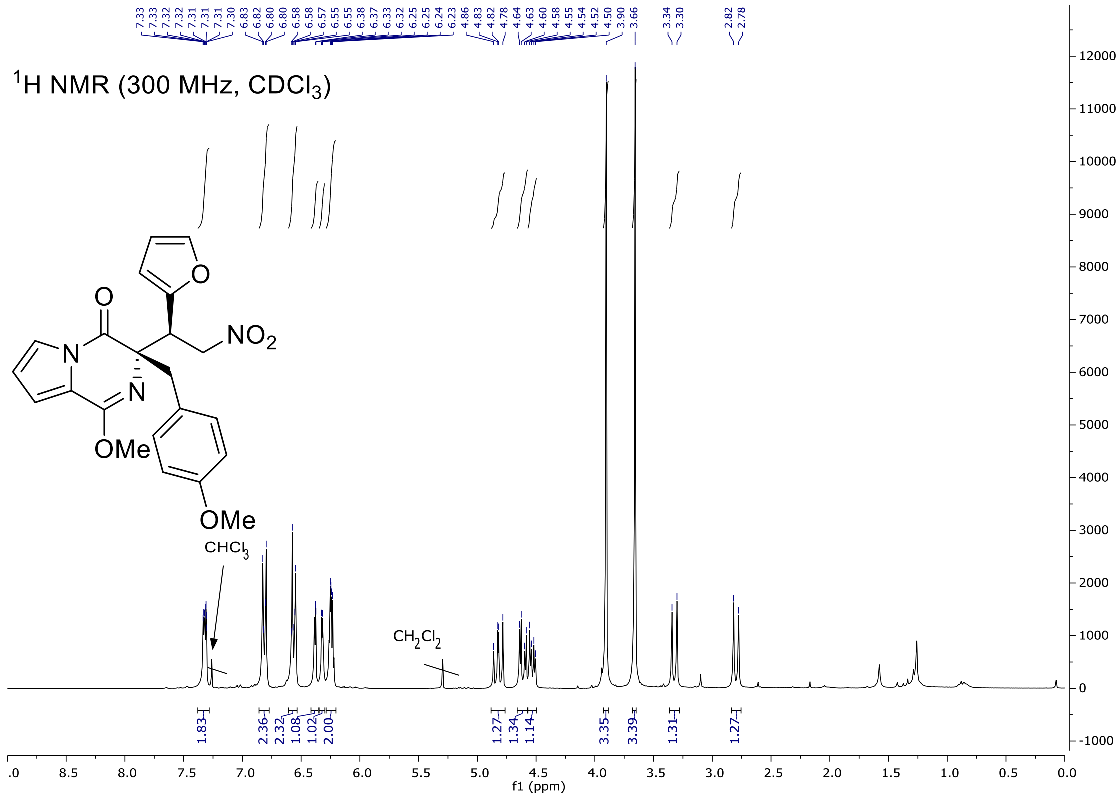

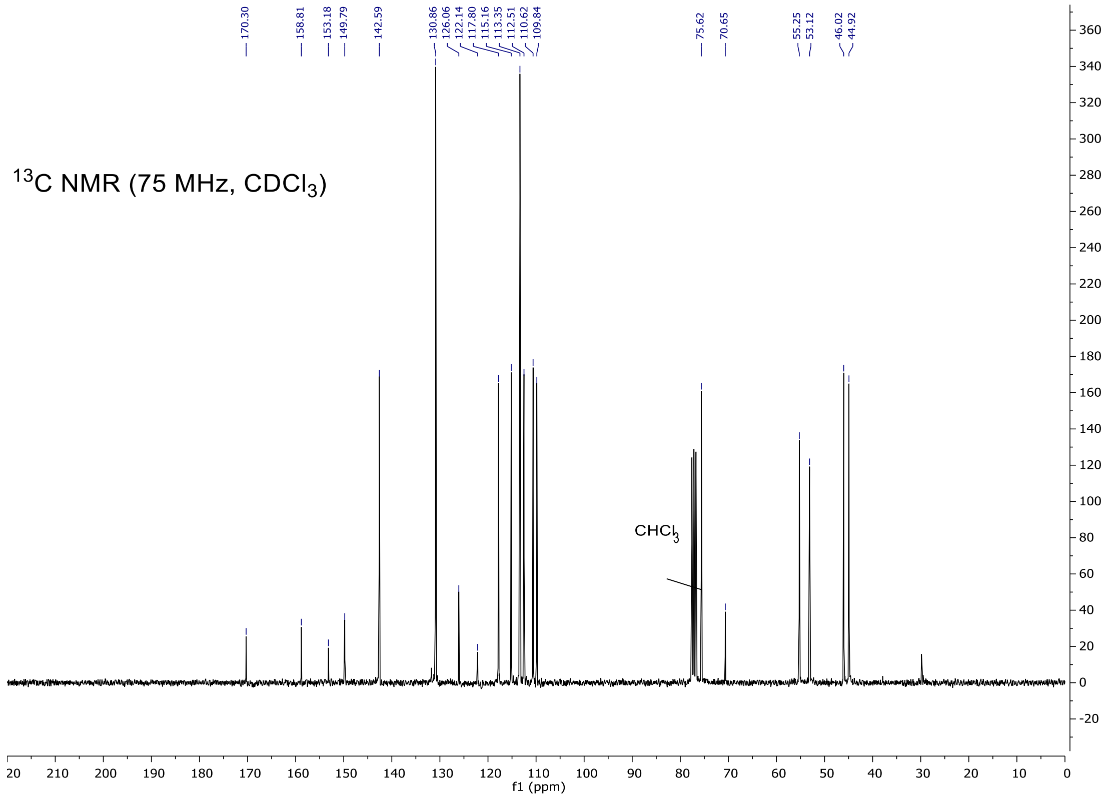

(*S*)-1-Methoxy-3-(4-methoxybenzyl)-3-((*S*)-2-nitro-1-(thiophen-2-yl)ethyl)pyrrolo[1,2-*a*]pyrazin-4(3*H*)-one (3ch)

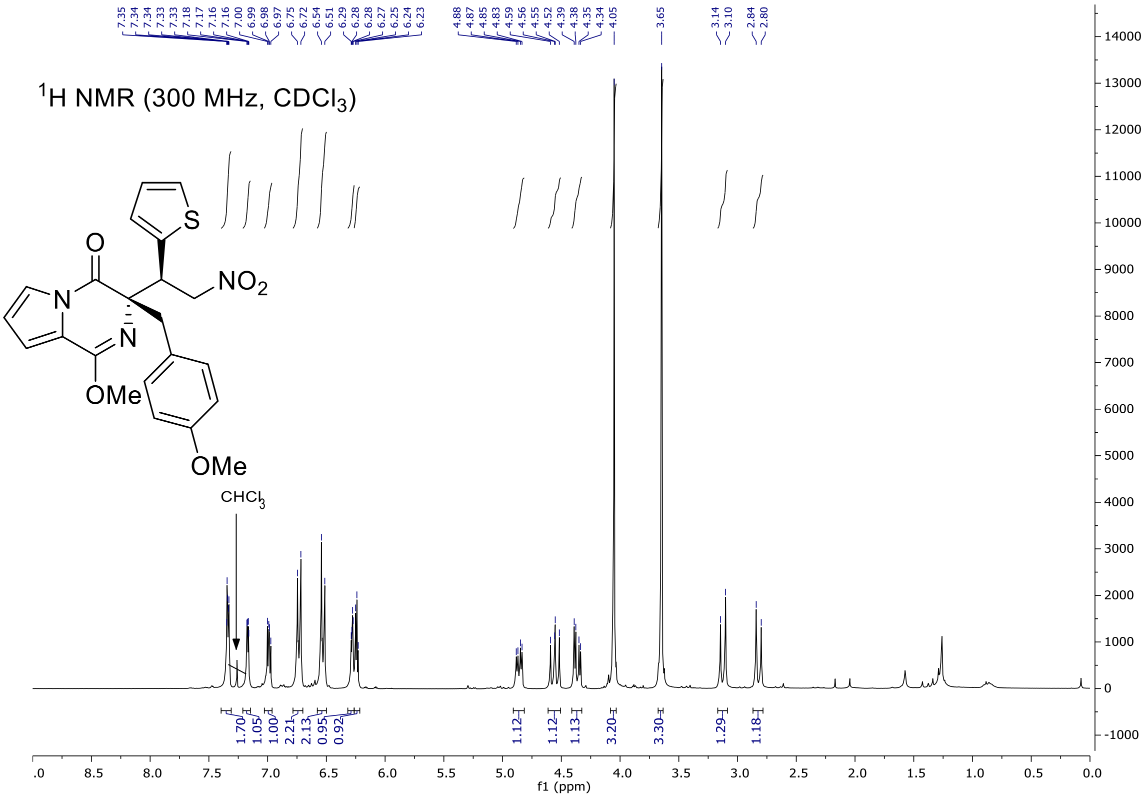

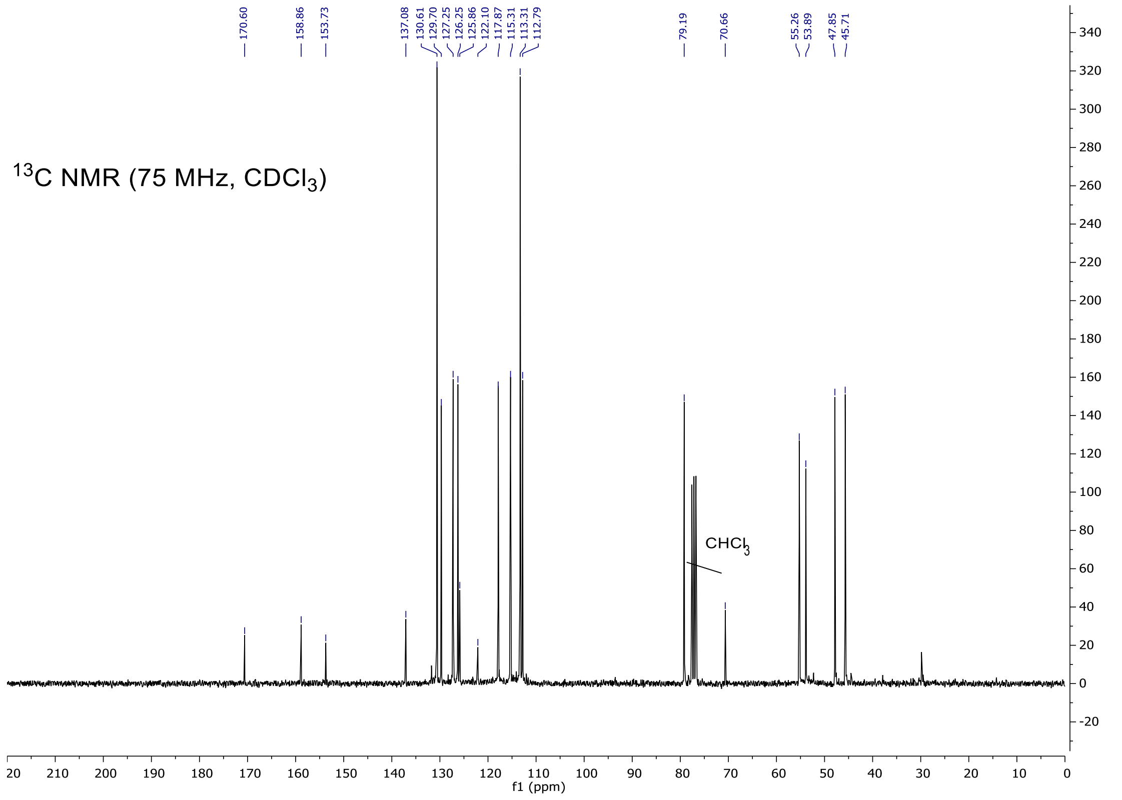

(*S*)-1-Methoxy-3-((*S*)-2-nitro-1-phenylethyl)-3-phenethylpyrrolo[1,2-*a*]pyrazin-4(3*H*)-one (3da)

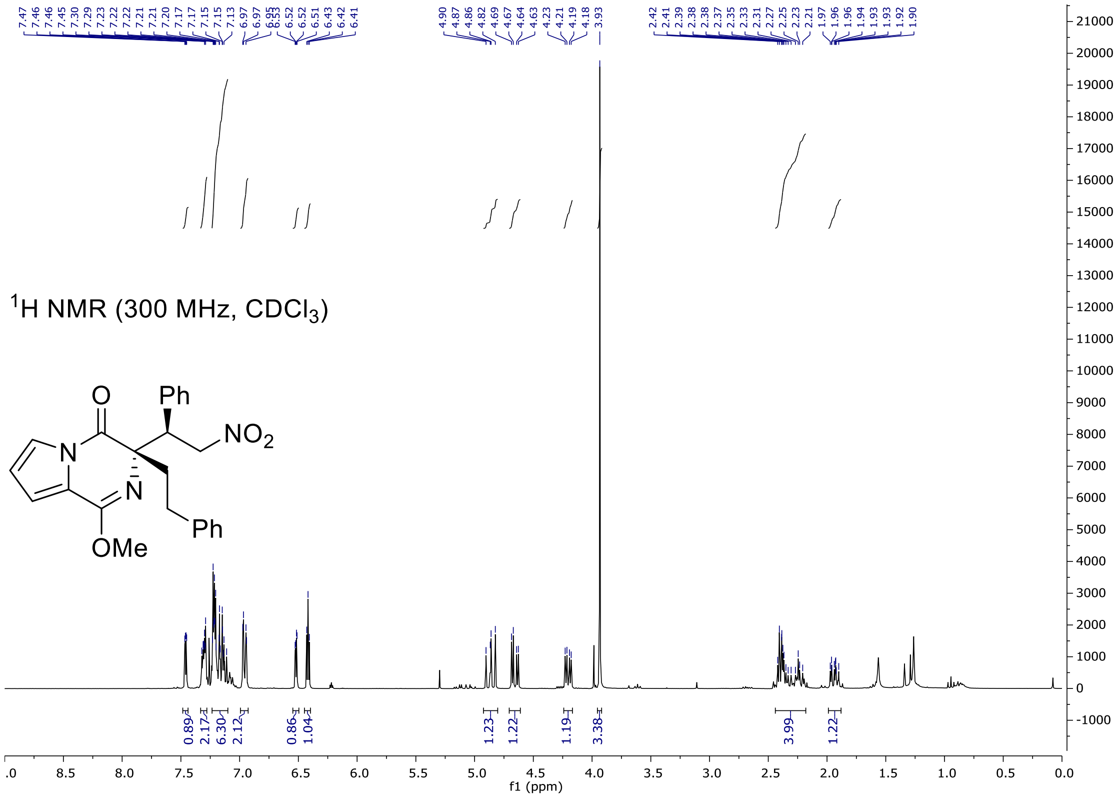

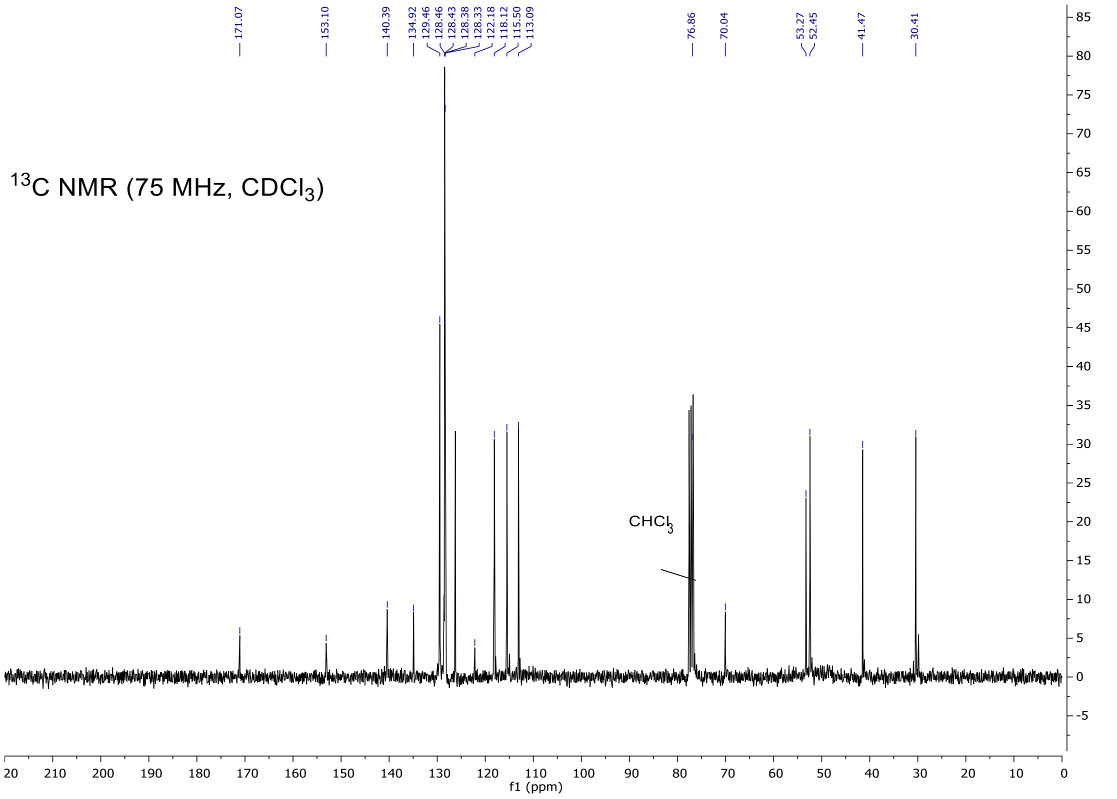

(*S*)-3-((*S*)-1-(Furan-3-yl)-2-nitroethyl)-1-methoxy-3-phenethylpyrrolo[1,2-*a*]pyrazin-4(3*H*)-one (3dg)

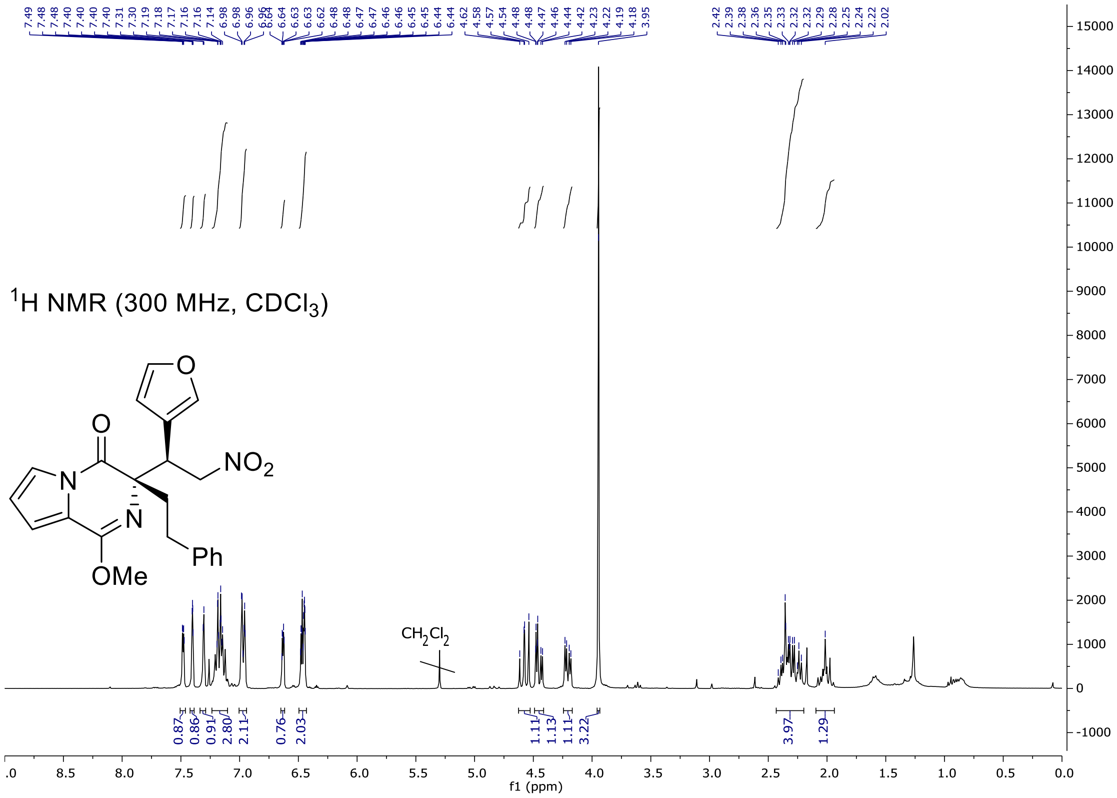

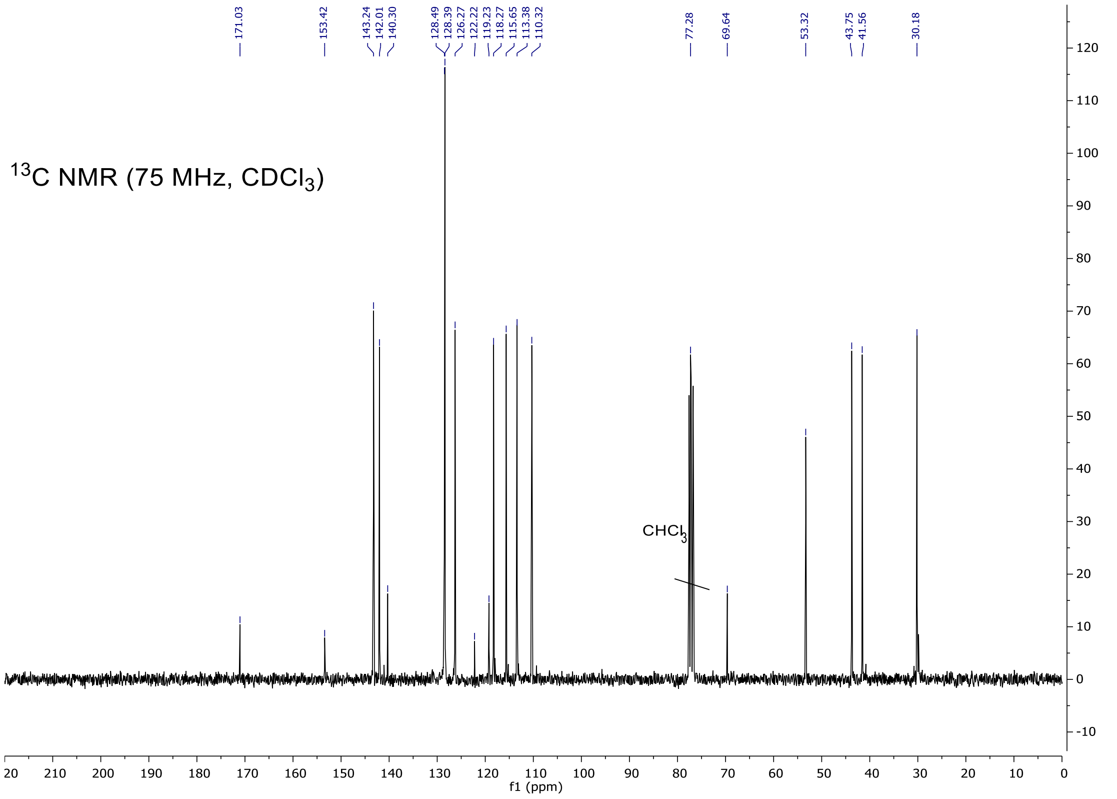

(*S*)-3-((*S*)-1-Cyclohexyl-2-nitroethyl)-1-methoxy-3-phenethylpyrrolo[1,2-*a*]pyrazin-4(3*H*)-one (3di)

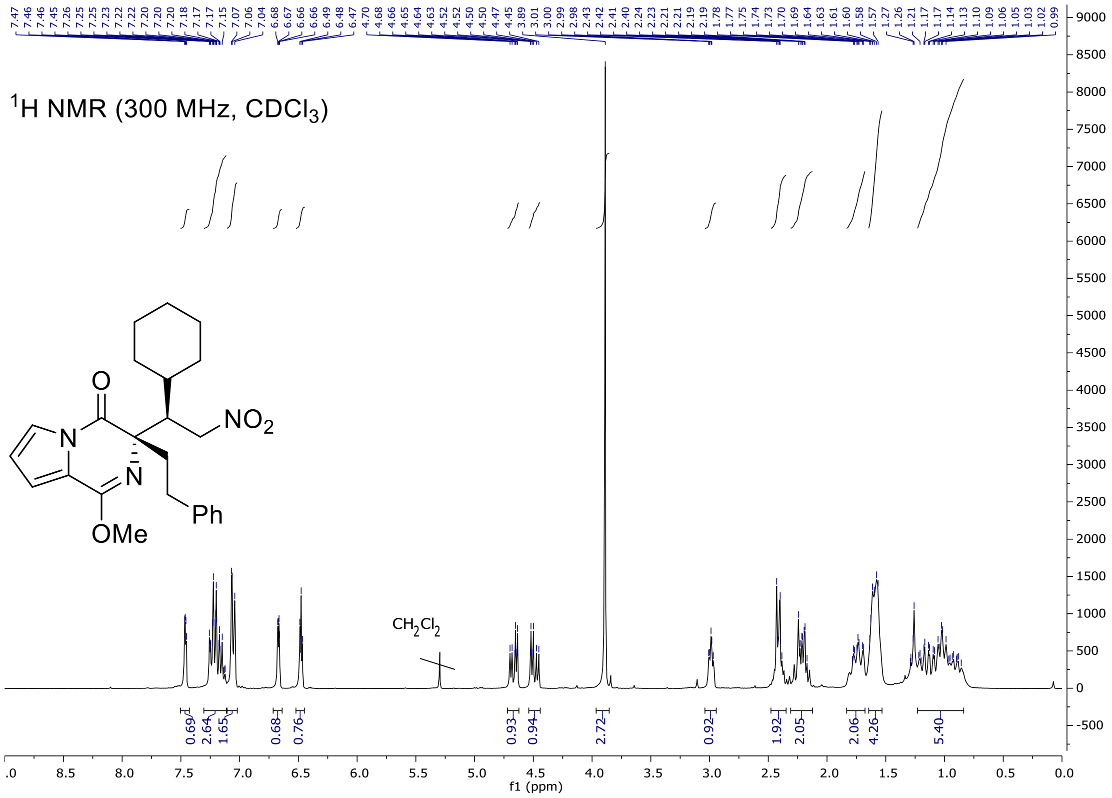

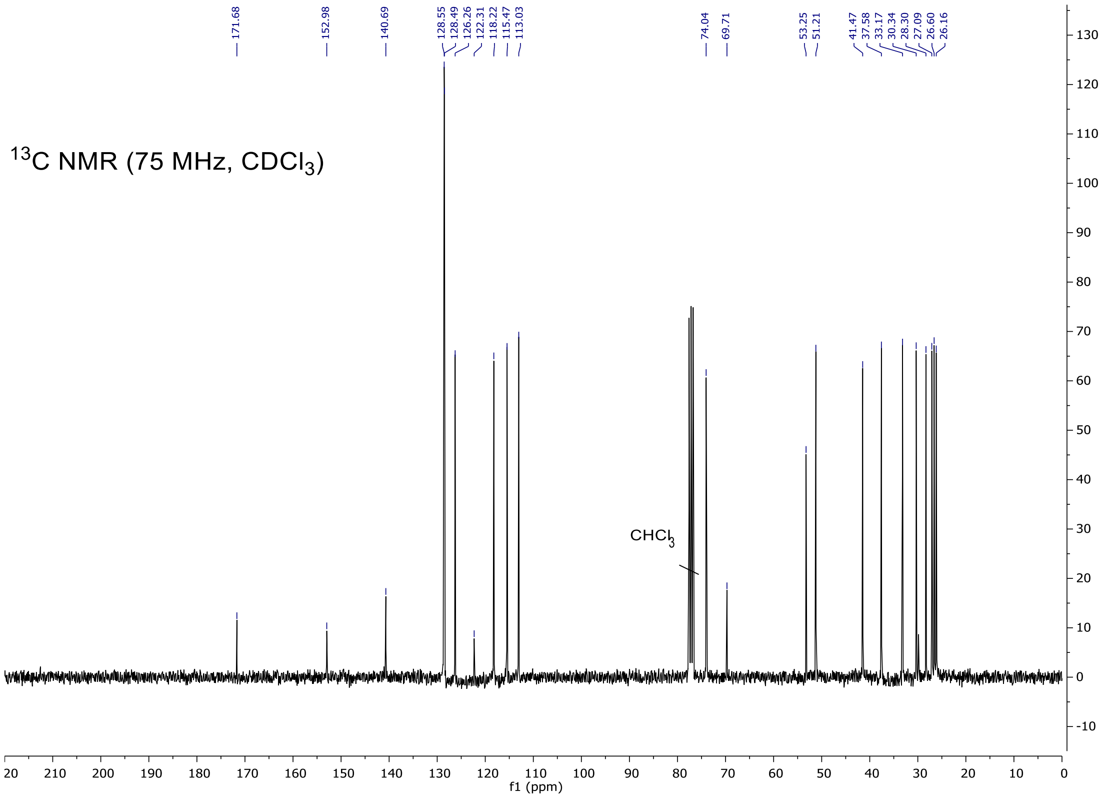

(*S*)-3-((1*H*-Indol-3-yl)methyl)-1-methoxy-3-((*S*)-2-nitro-1-phenylethyl)pyrrolo[1,2-*a*]pyrazin-4(3*H*)-one (3ea)

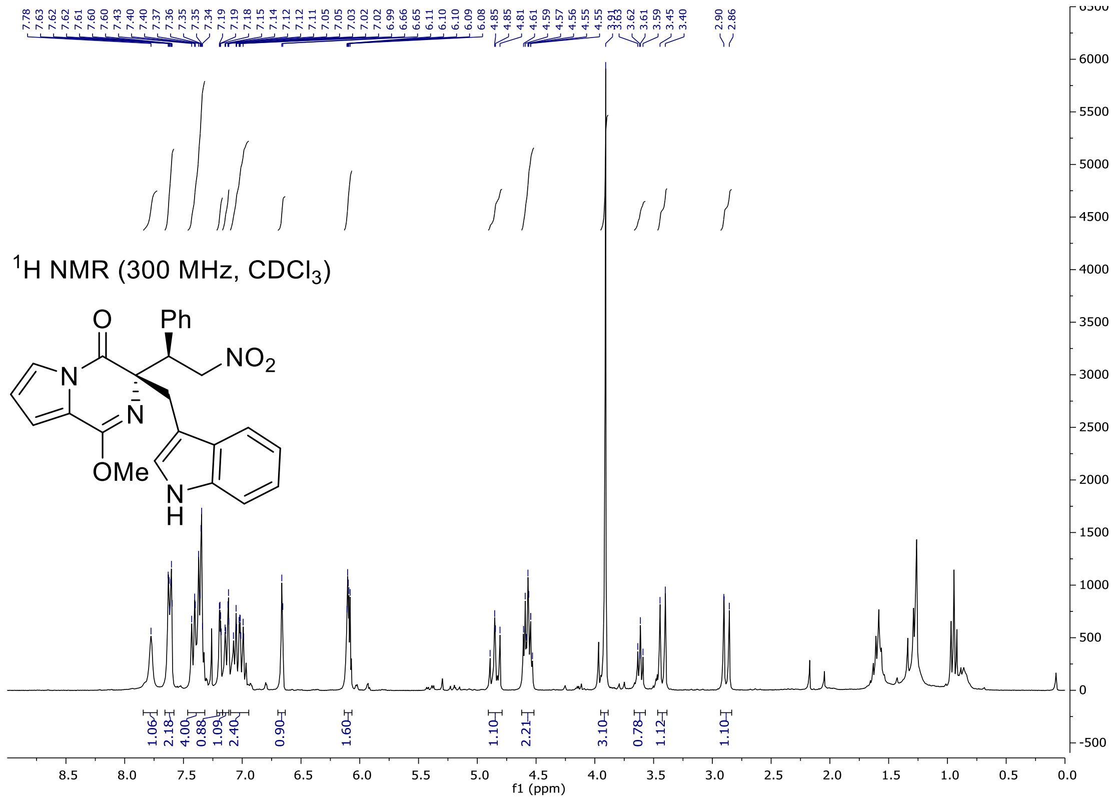

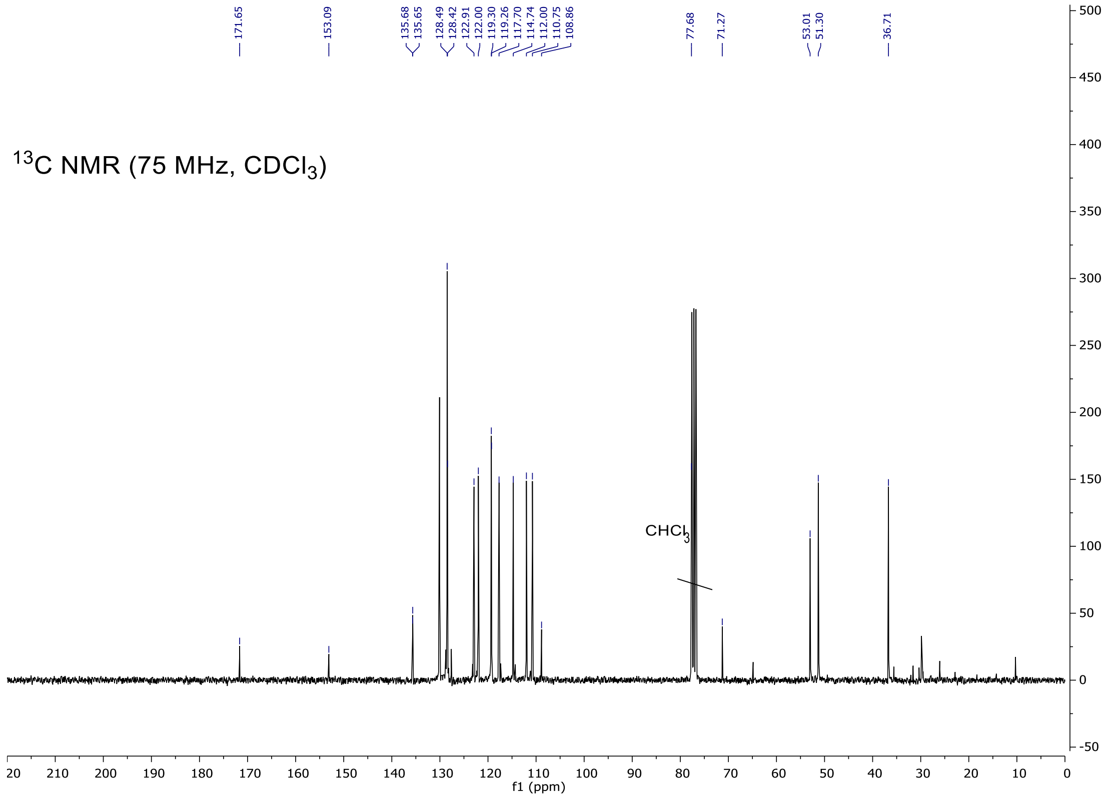

(*S*)-3-Allyl-1-methoxy-3-((*S*)-2-nitro-1-phenylethyl)pyrrolo[1,2-*a*]pyrazin-4(3*H*)-one (3fa)

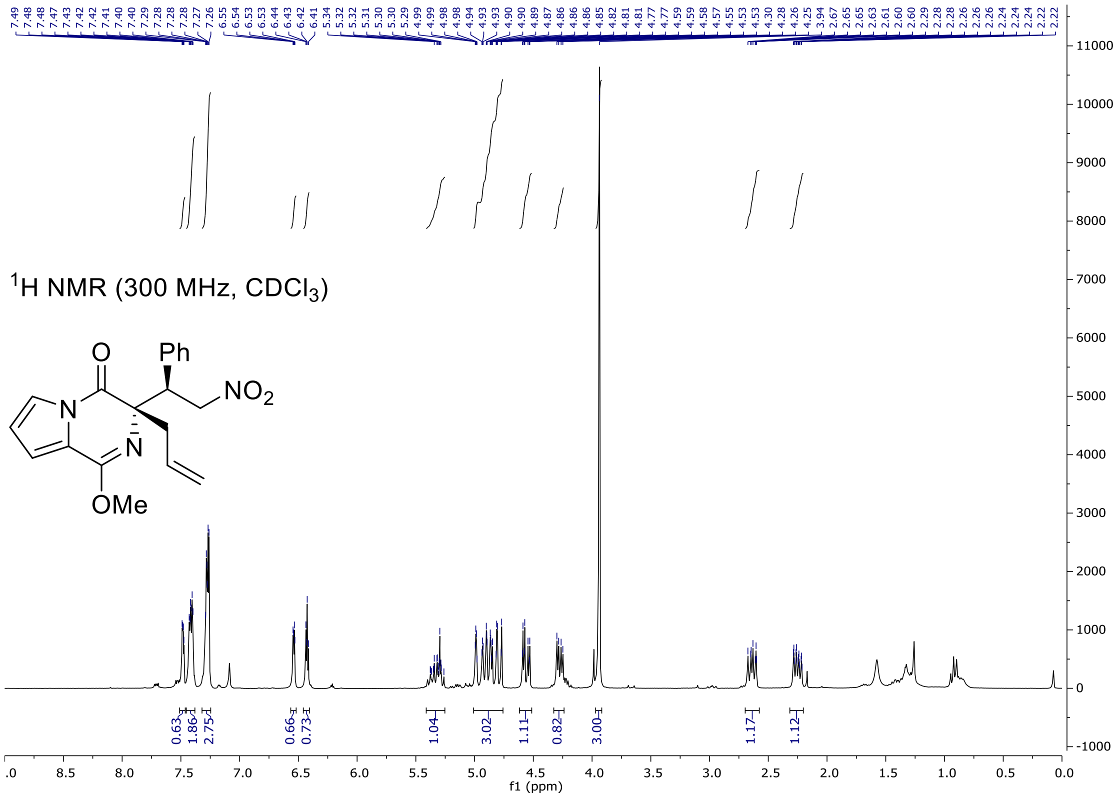

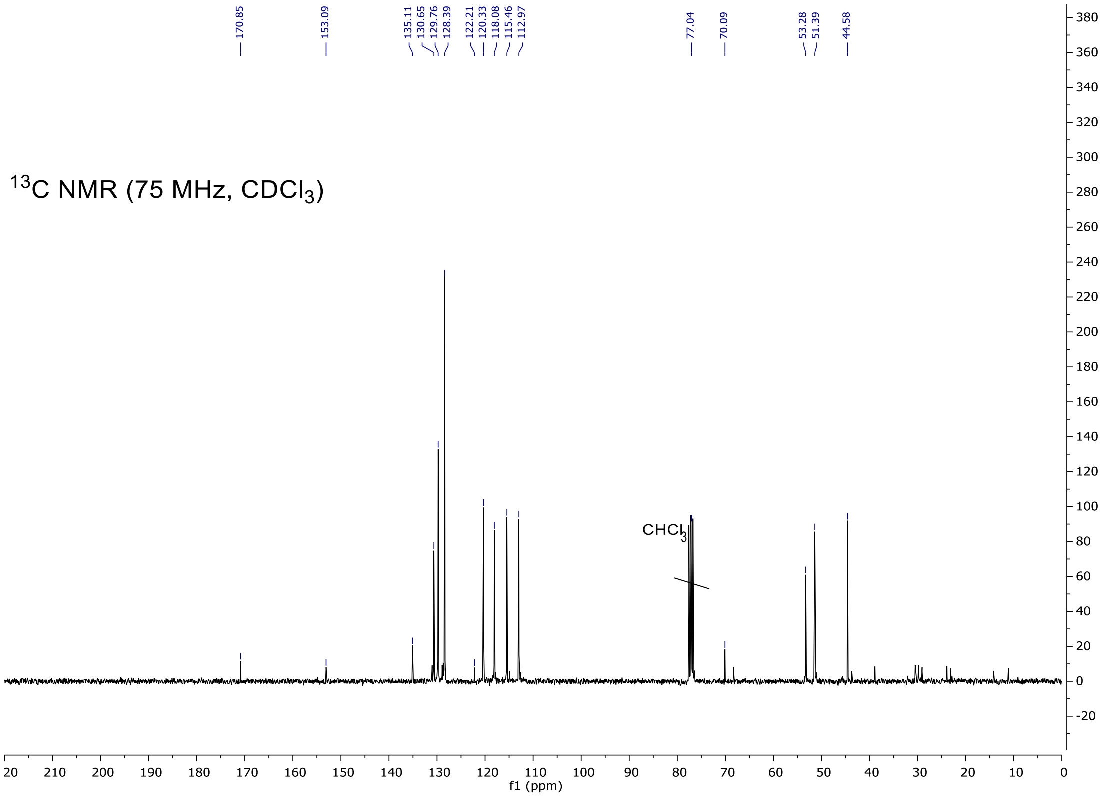

(*R*)-3-((*S*)-1-Cyclohexyl-2-nitroethyl)-1-methoxy-3-phenylpyrrolo[1,2-*a*]pyrazin-4(3*H*)-one (3gi)

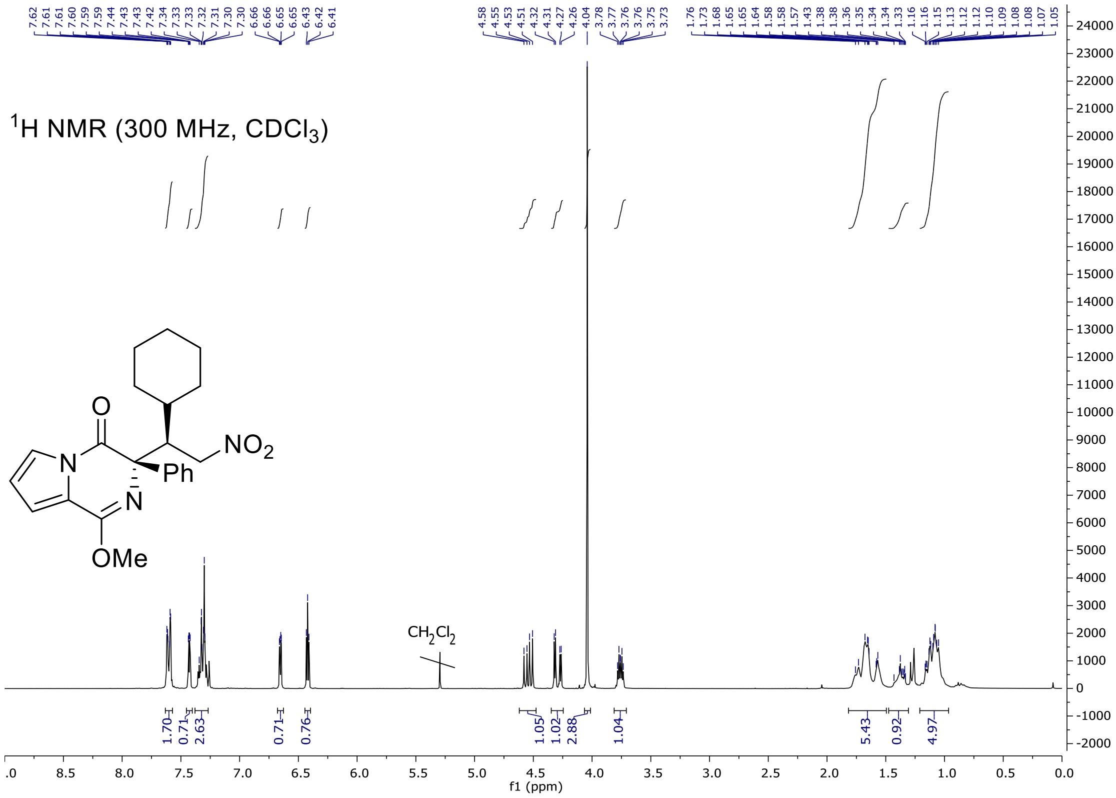

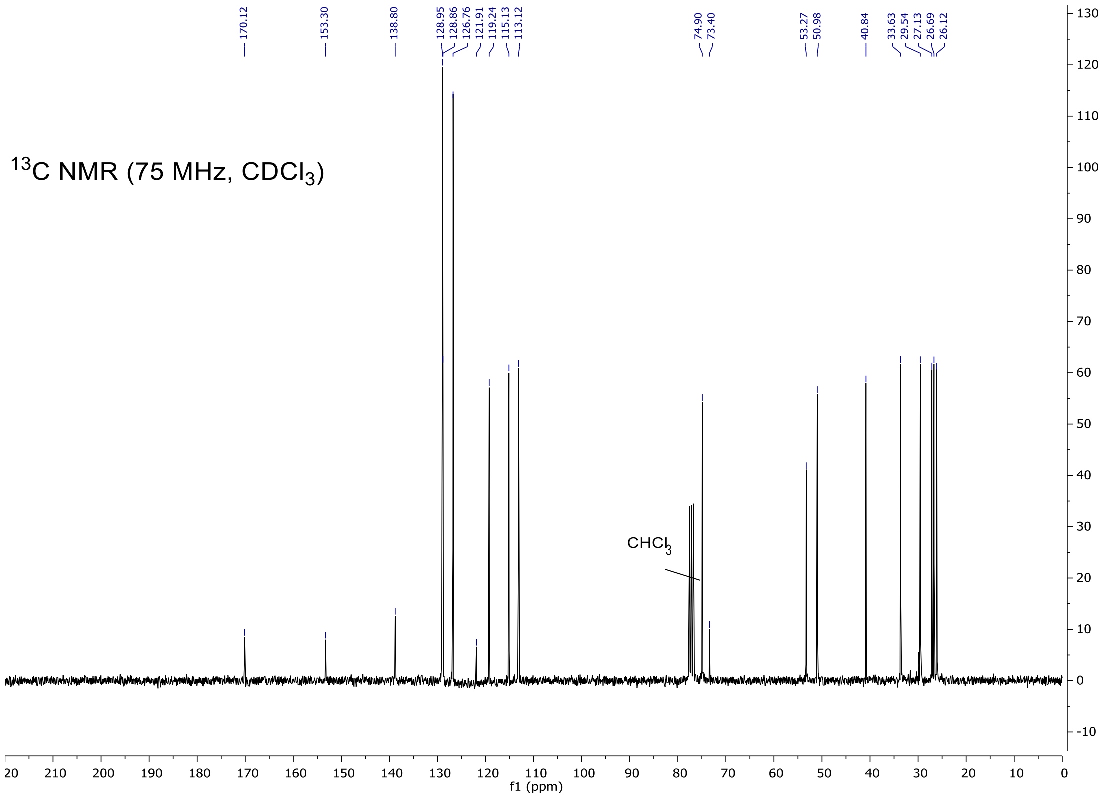

(*S*)-3-Hexyl-1-methoxy-3-((*S*)-2-nitro-1-phenylethyl)pyrrolo[1,2-*a*]pyrazin-4(3*H*)-one (3ha)

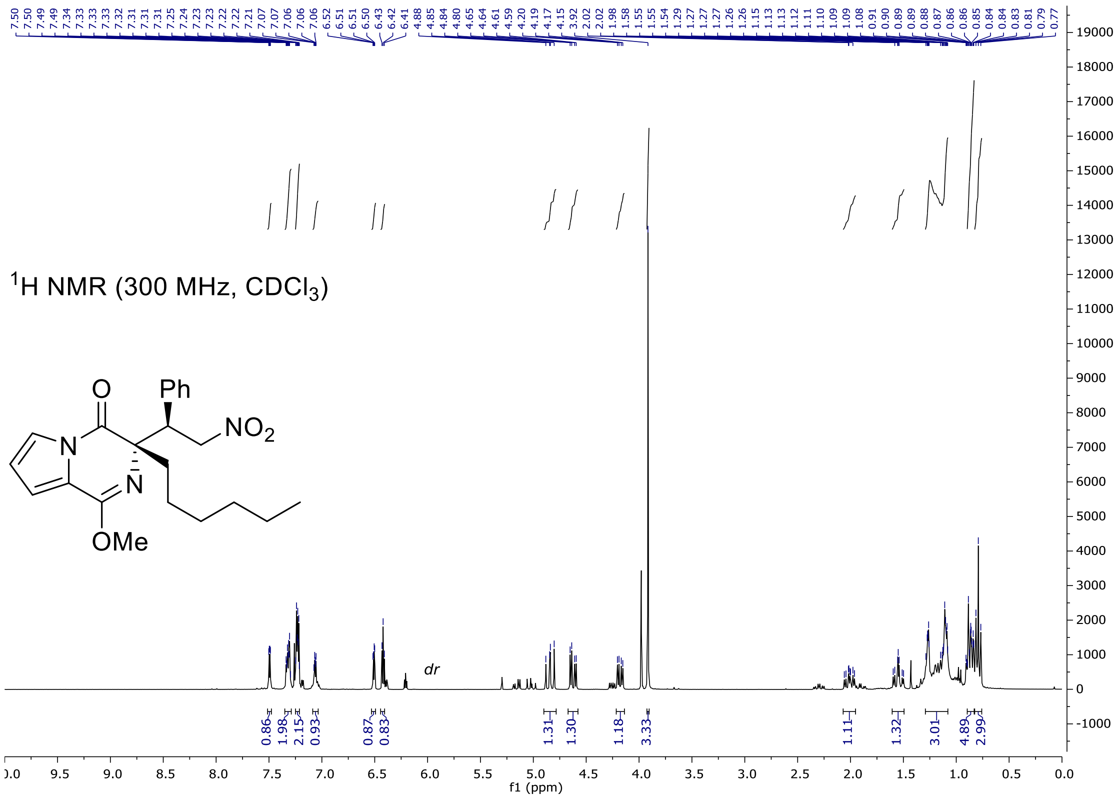

(*S*)-3-Benzyl-3-((*S*)-2-nitro-1-phenylethyl)-2,3-dihydropyrrolo[1,2-*a*]pyrazine-1,4-dione (4aa)

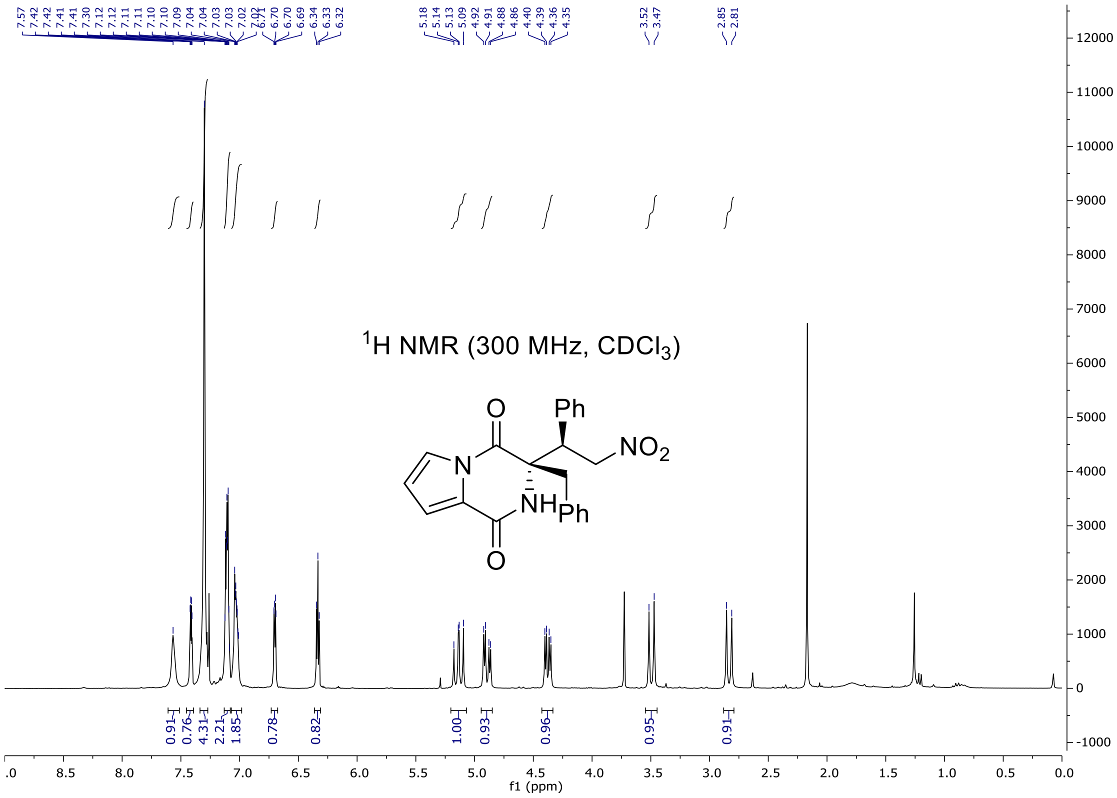

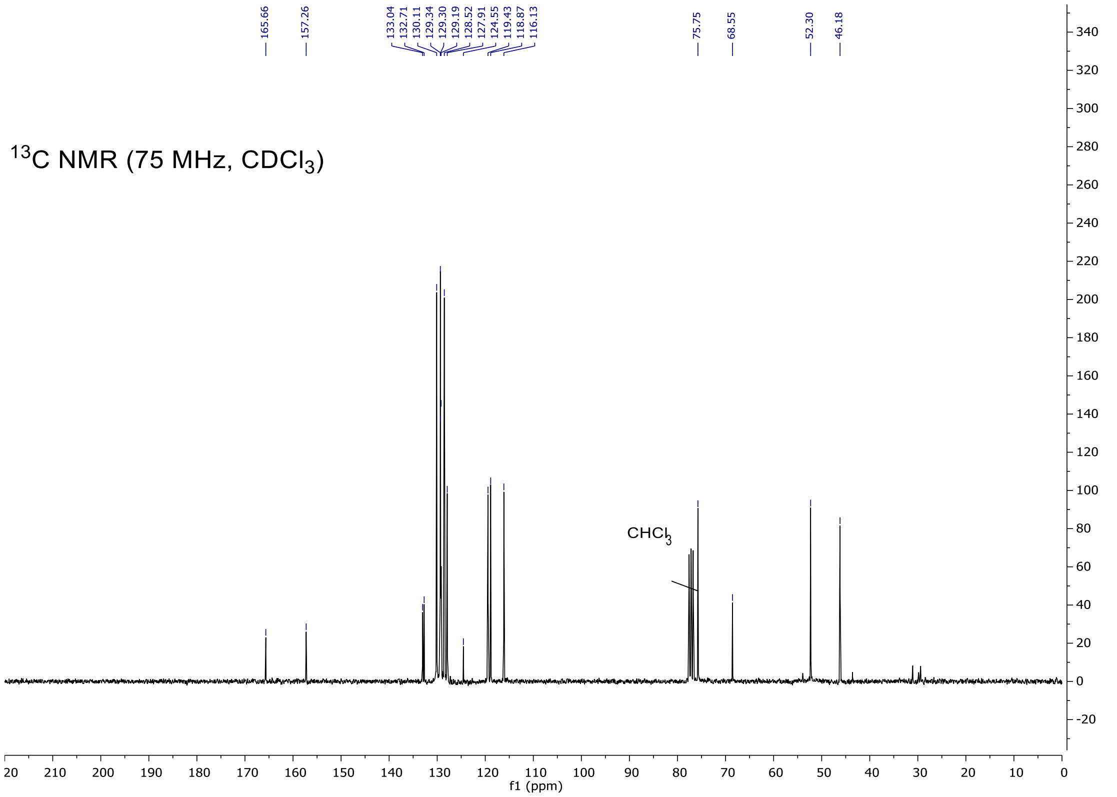

(*S*)-3-Isobutyl-3-((*S*)-2-nitro-1-phenylethyl)-2,3-dihydropyrrolo[1,2-*a*]pyrazine-1,4-dione (4ba)

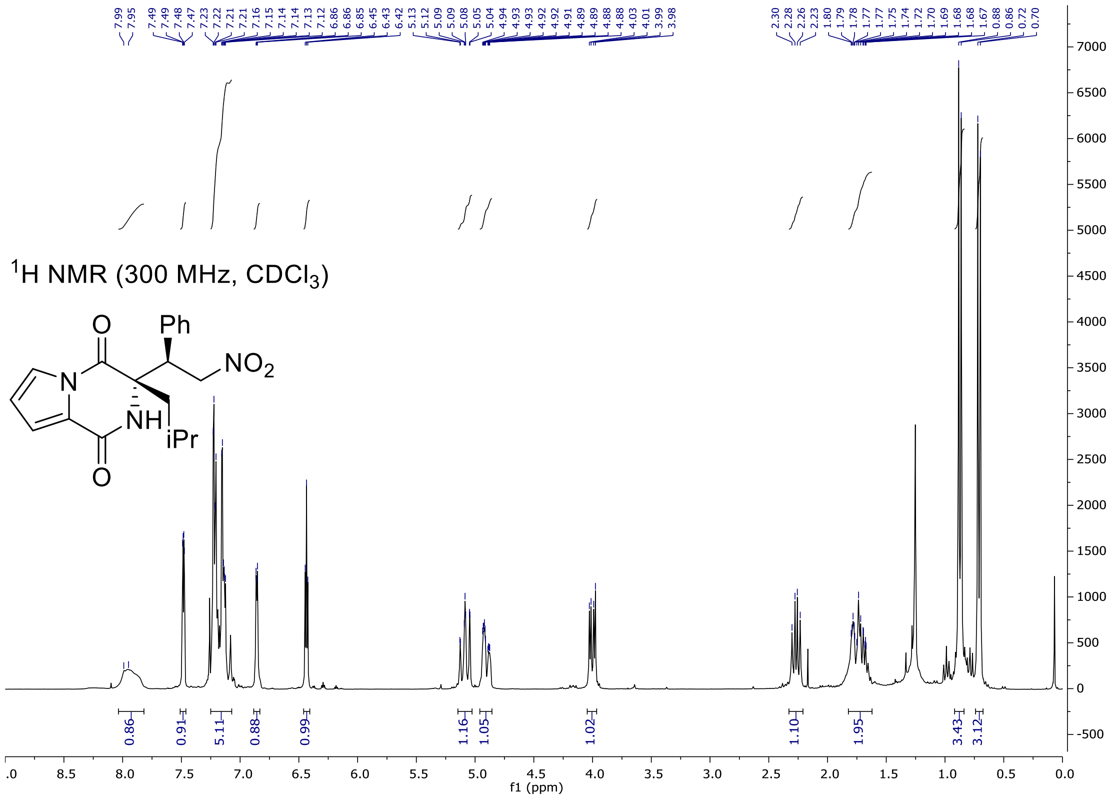

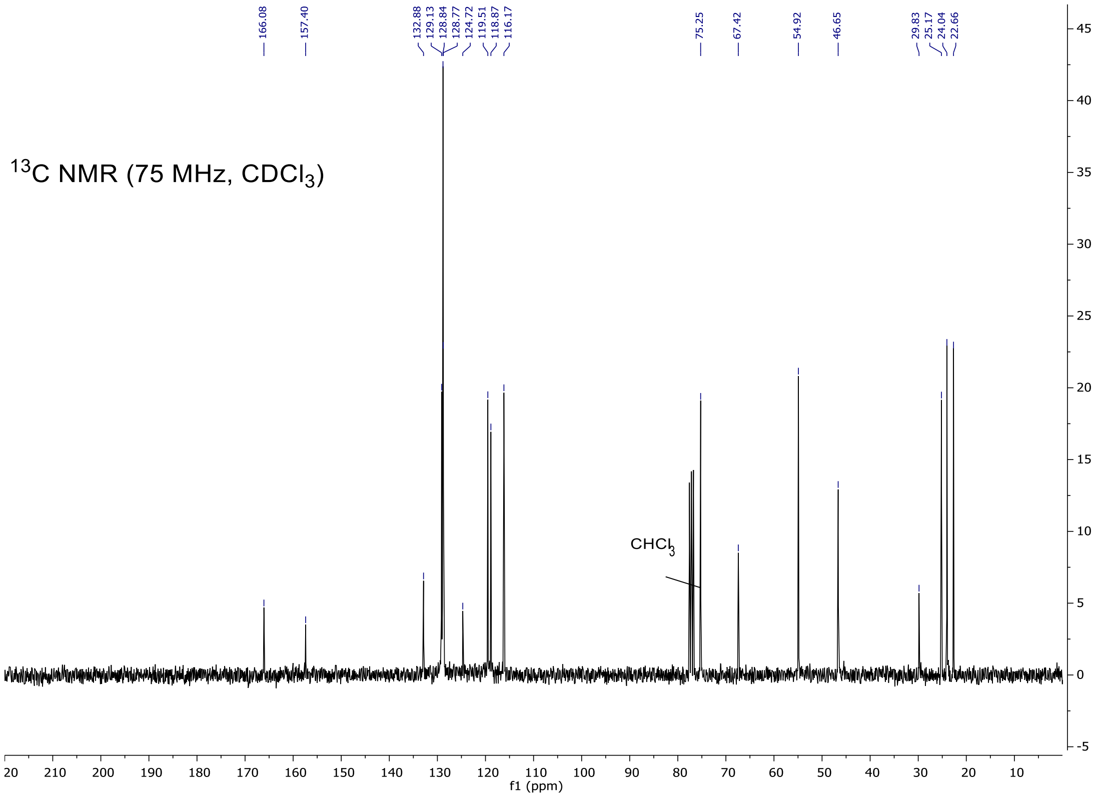

(*S*)-3-allyl-3-((*S*)-2-nitro-1-phenylethyl)-2,3-dihydropyrrolo[1,2-*a*]pyrazine-1,4-dione (4fa)

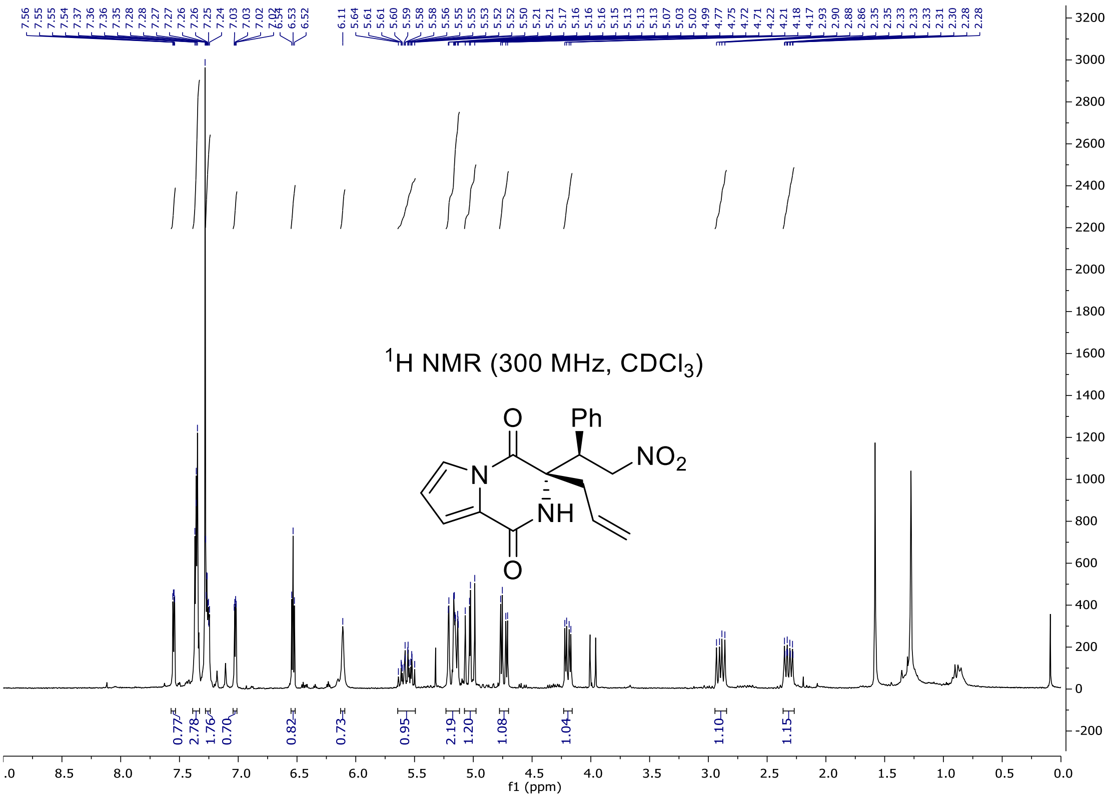

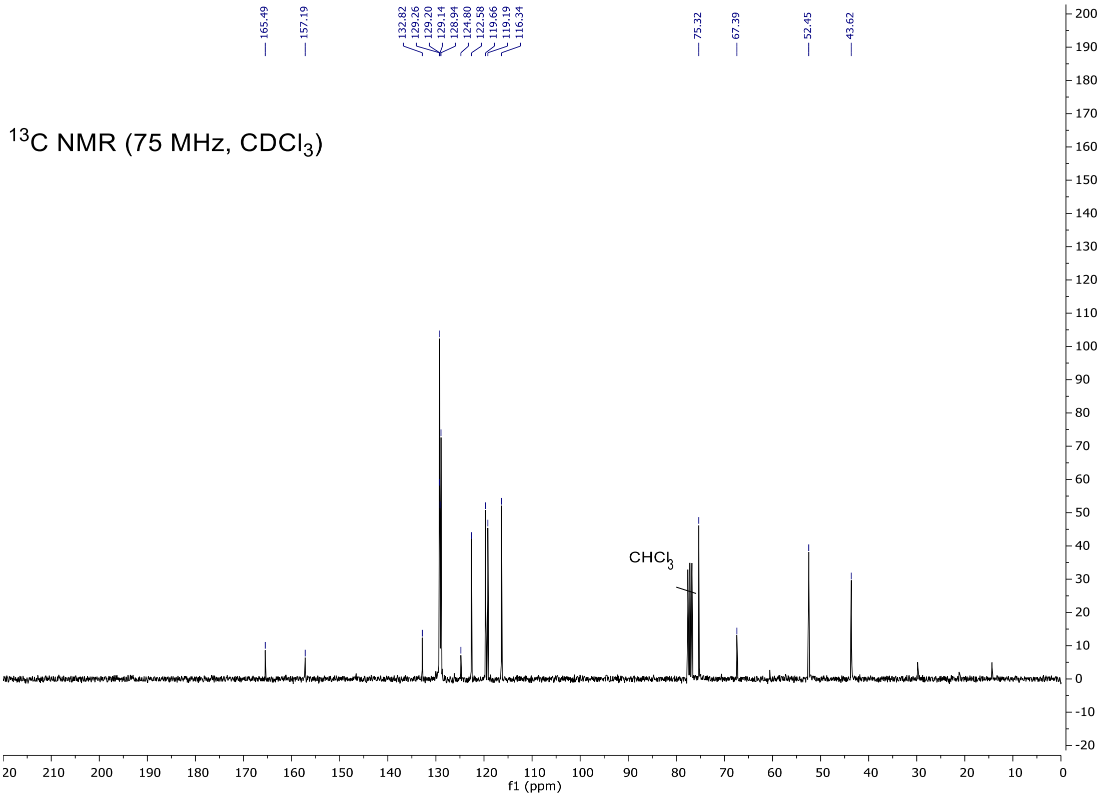

(*S*)-3-Hexyl-3-((*S*)-2-nitro-1-phenylethyl)-2,3-dihydropyrrolo[1,2-*a*]pyrazine-1,4-dione (4ha)

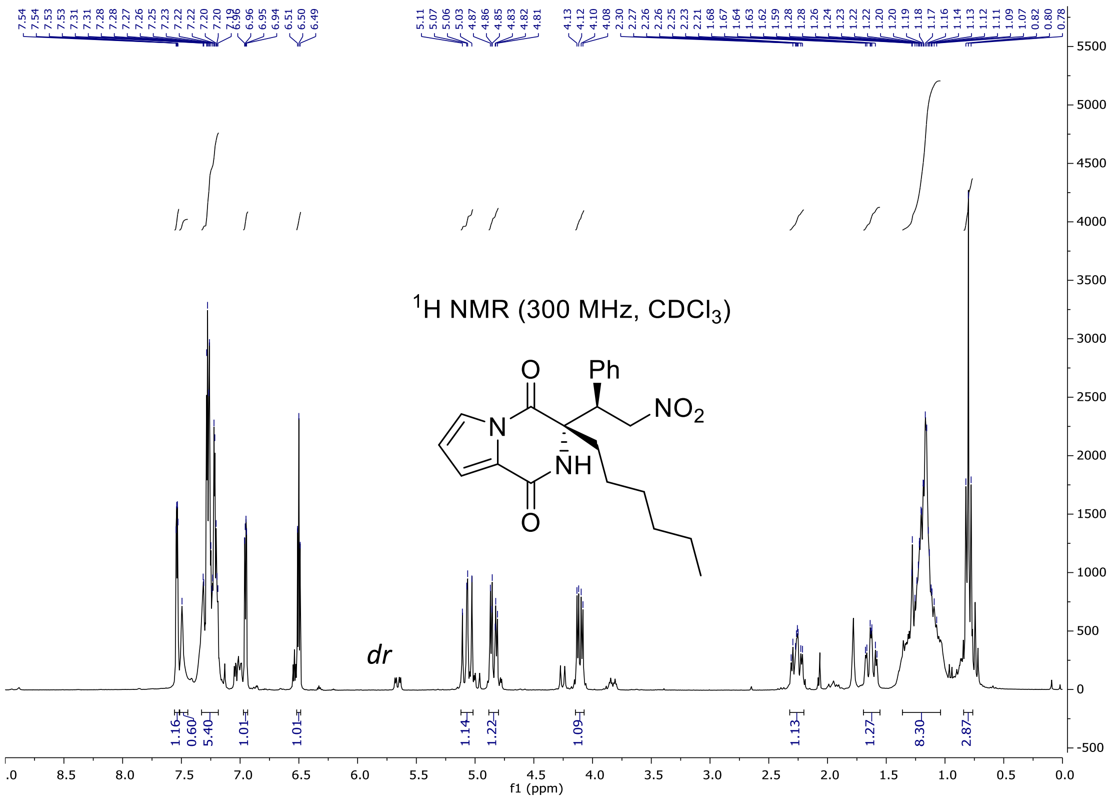

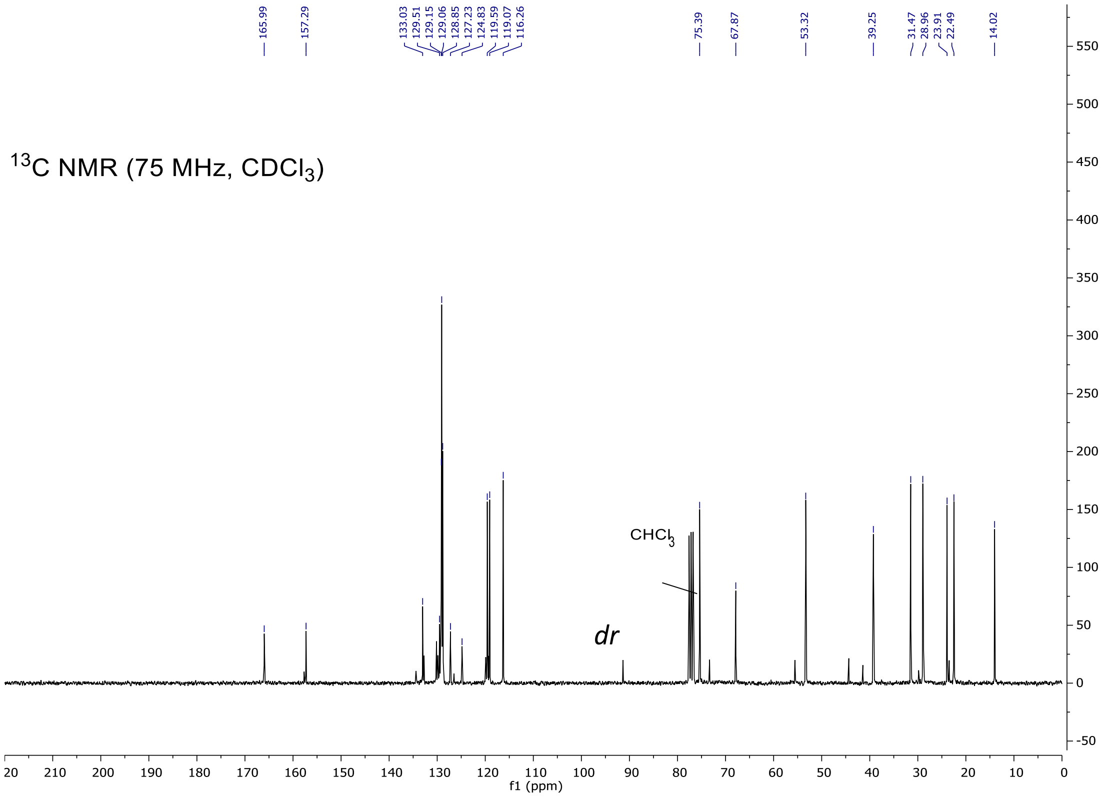

***N*-((*R*)-2-((*S*)-3-Benzyl-1,4-dioxo-1,2,3,4-tetrahydropyrrolo[1,2-*a*]pyrazin-3-yl)-2-phenylethyl)benzamide (5)**

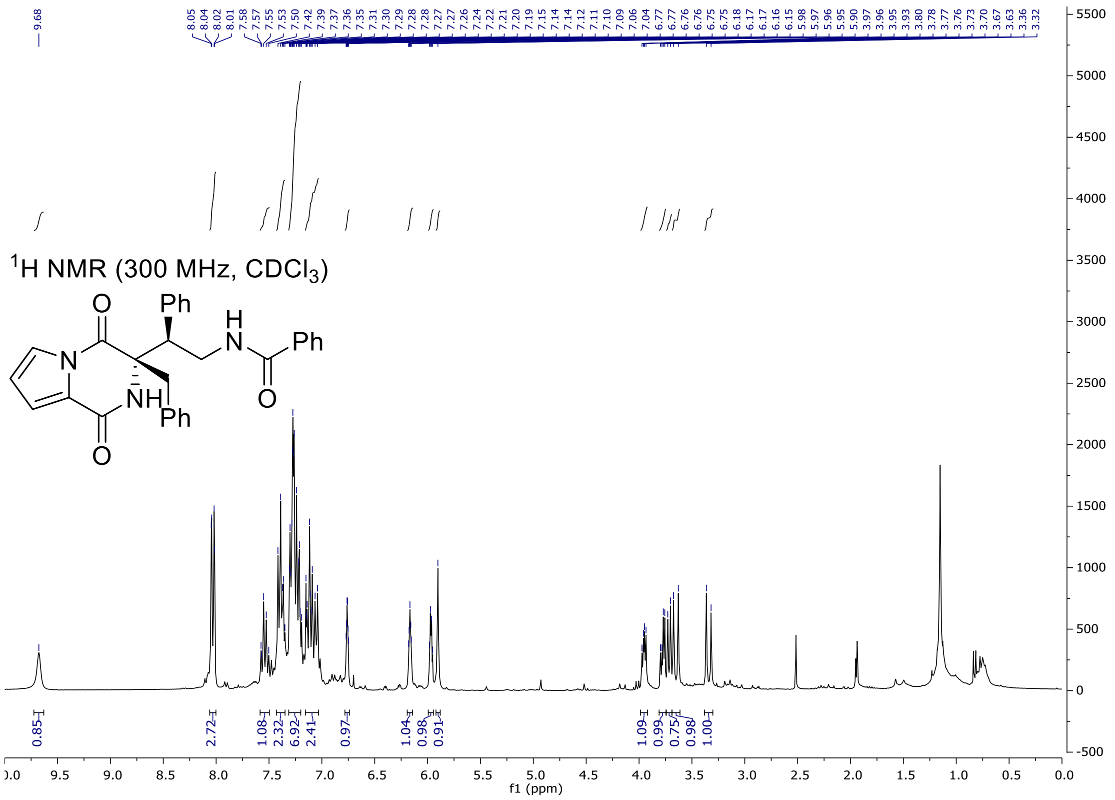

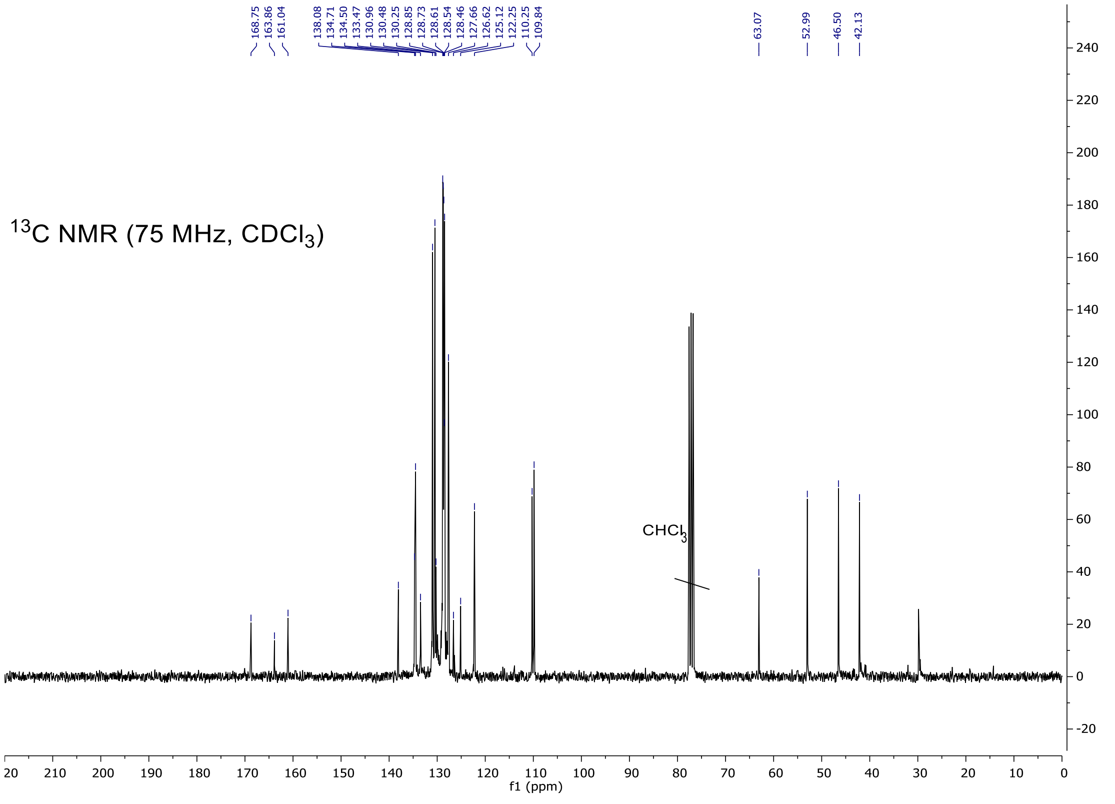

COSY
(300 MHz, $CDCl_3$)

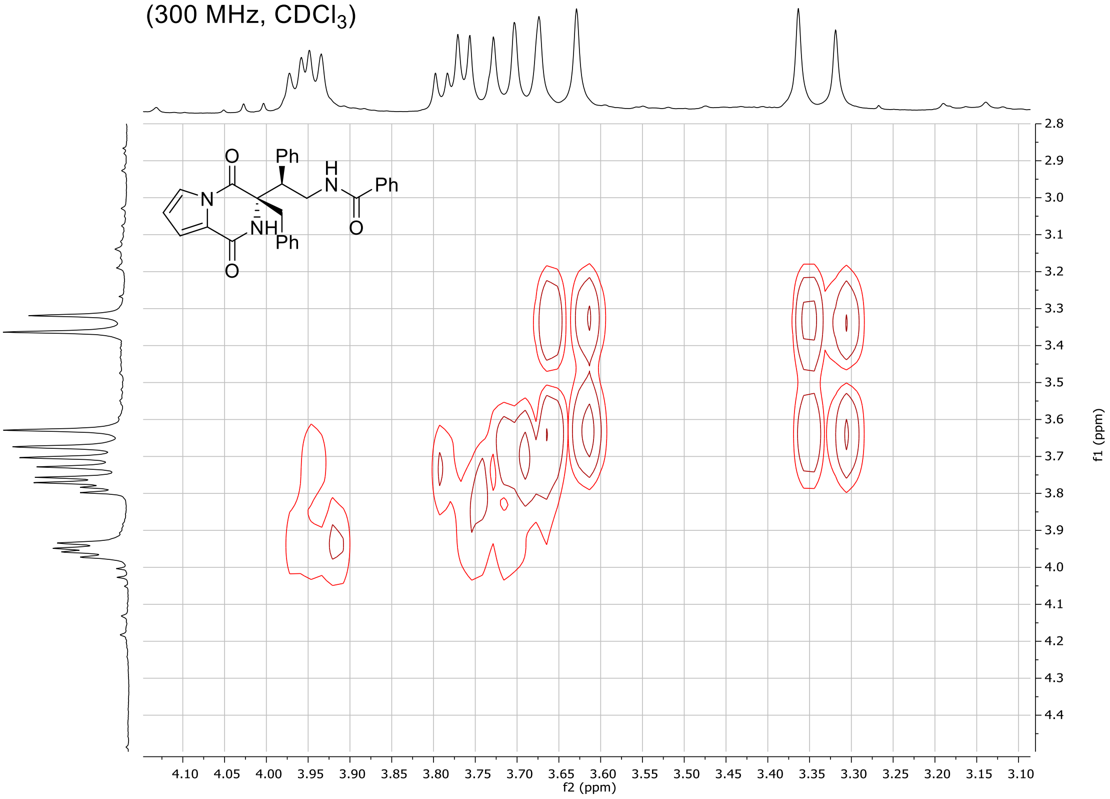

HSQC
(300 MHz, $CDCl_3$)

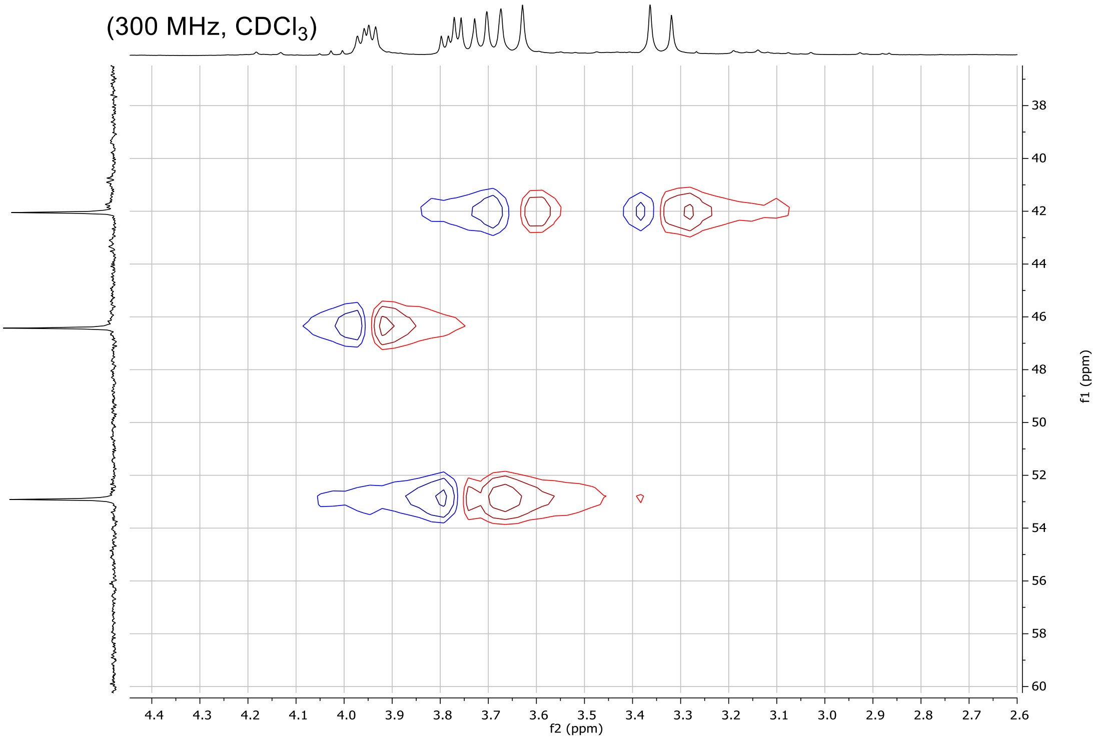

(3a*S*,5*R*,6*S*,6a*R*)-6-phenyl-1-((trimethylsilyl)oxy)-3a,4,6,6a-tetrahydro-1*H*,3*H*,4'*H*-spiro[cyclopenta[*c*]isoxazole-5,3'-pyrrolo[1,2-*a*]pyrazine]-1',4'(2'*H*)-dione (6)

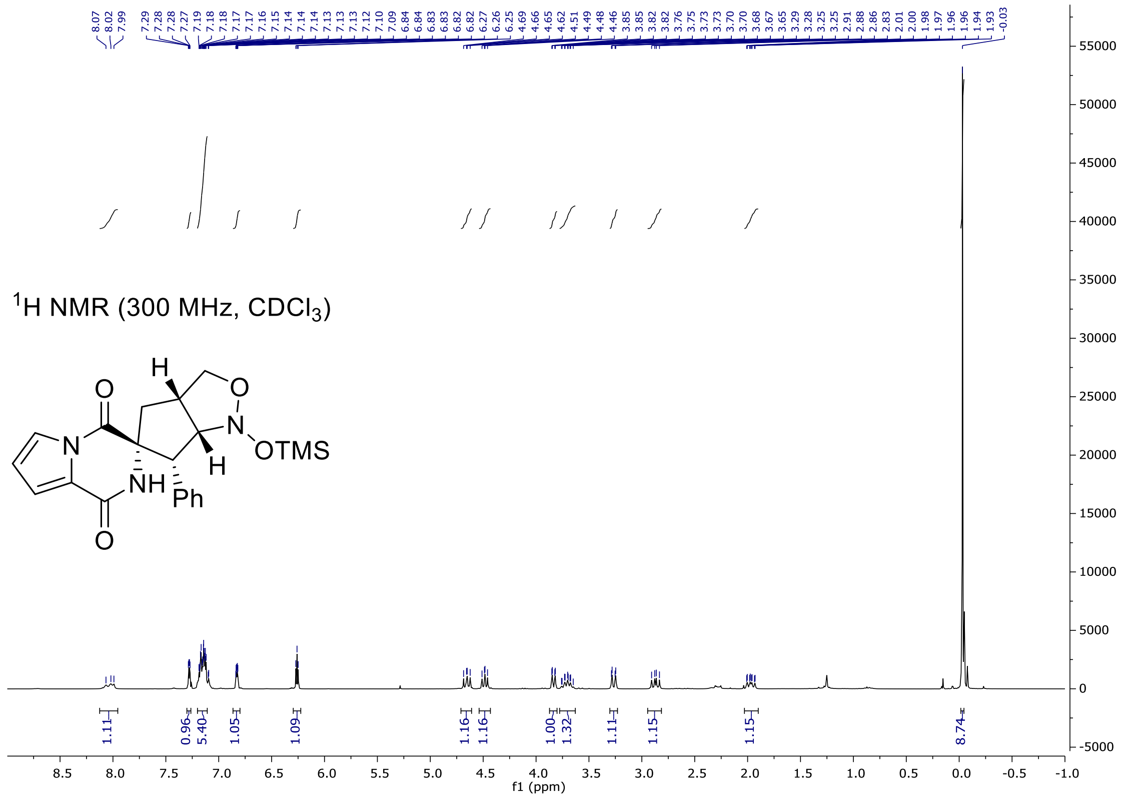

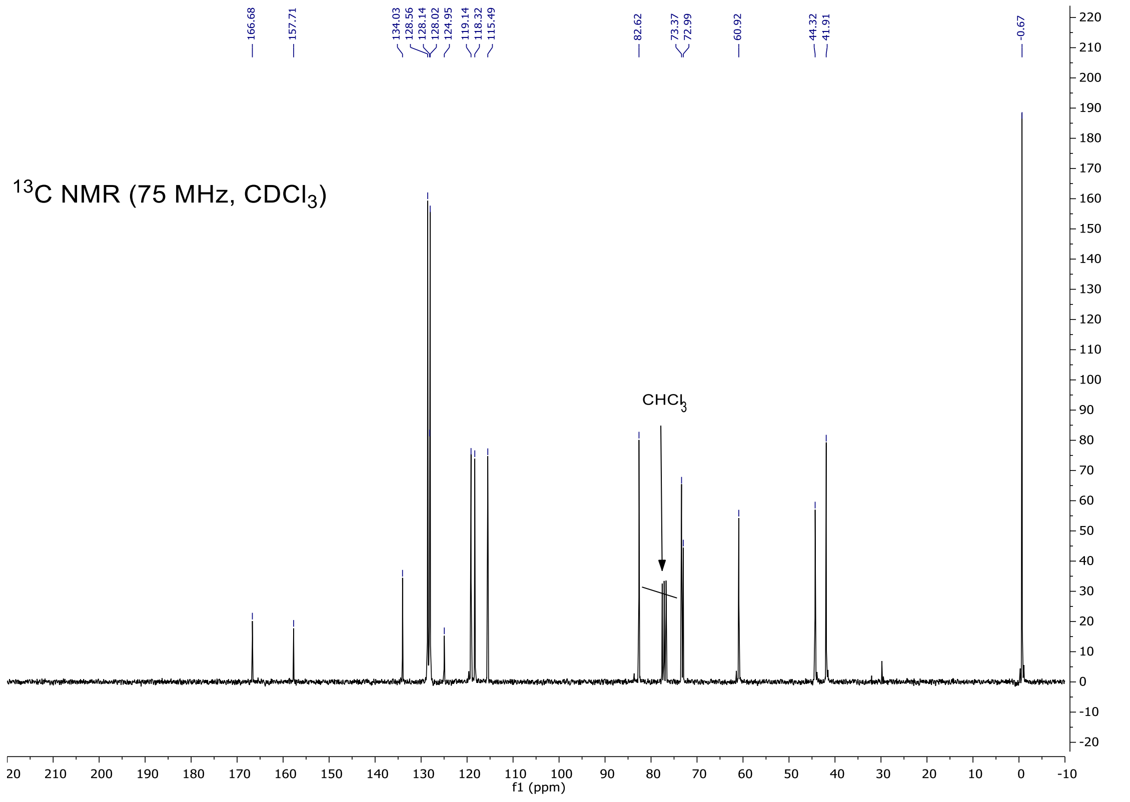

COSY
(500 MHz, $CDCl_3$)

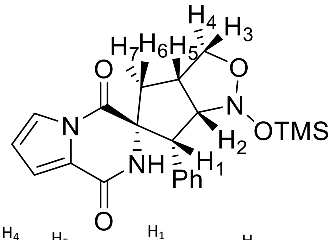

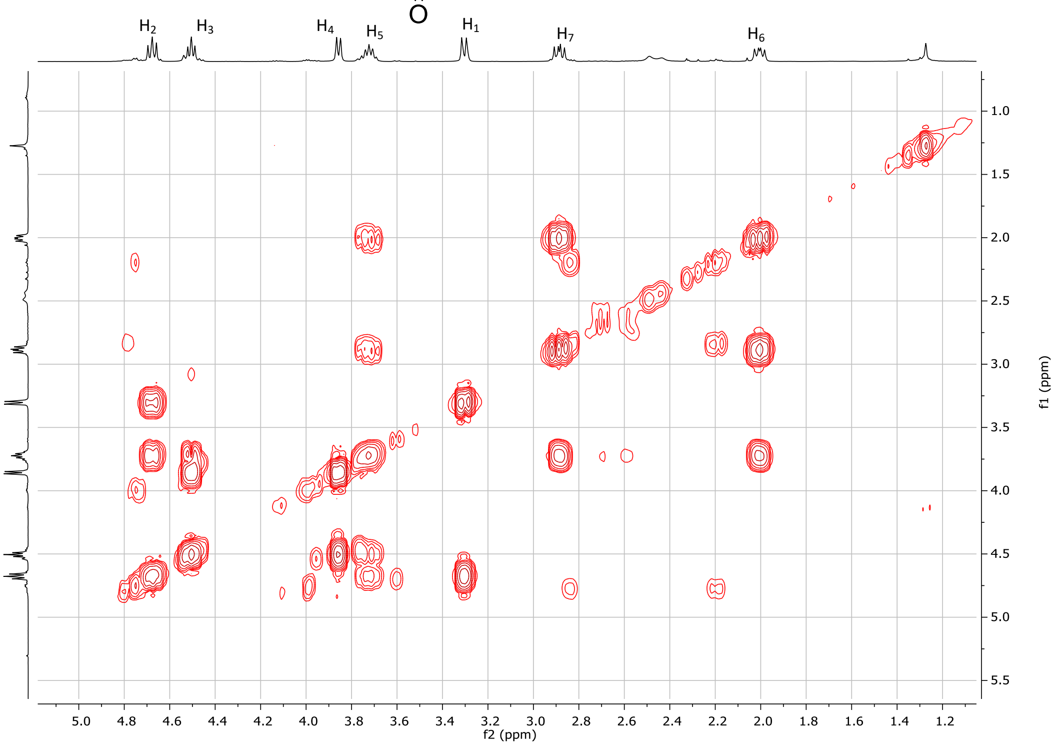

DEPT
(500 MHz, $CDCl_3$)

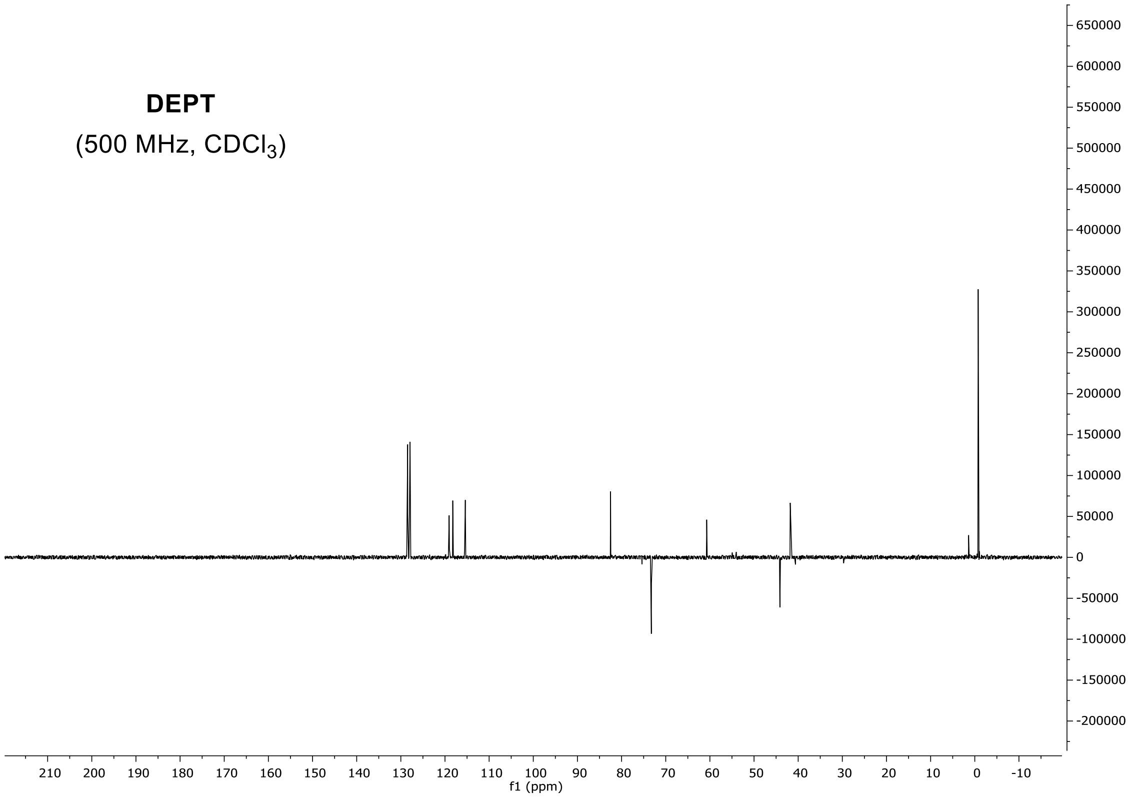

HSQC

(500 MHz, $CDCl_3$)

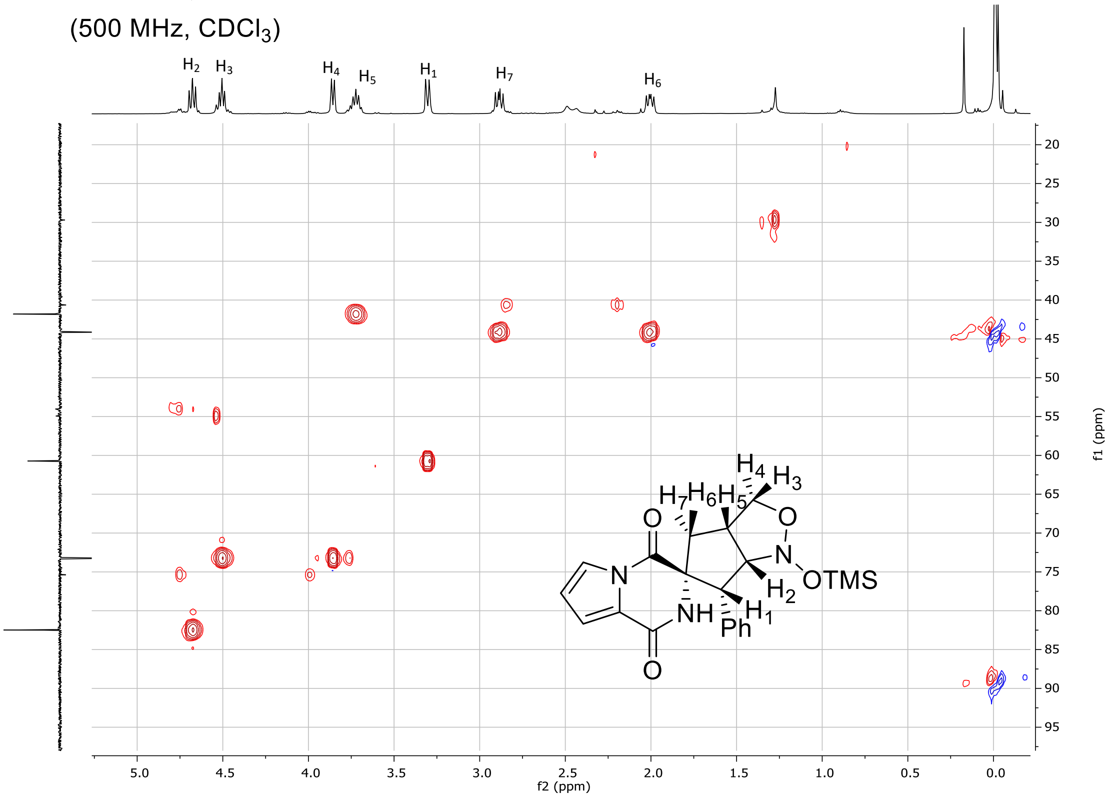

HMBC

(500 MHz, $CDCl_3$)

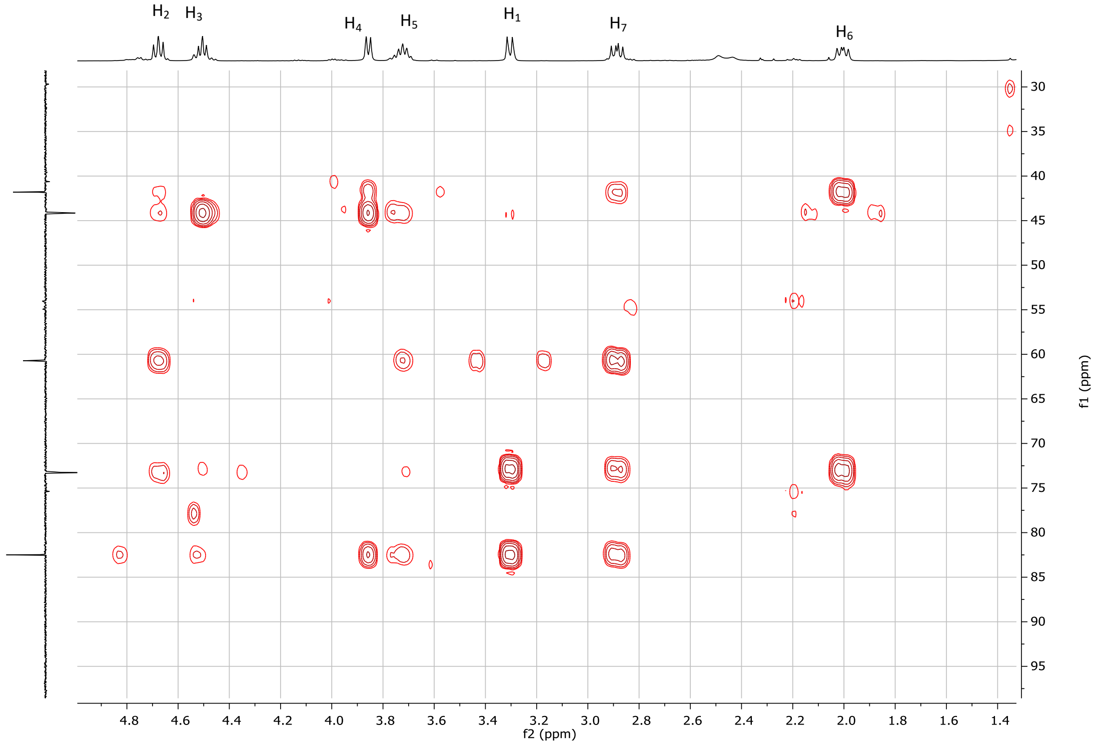

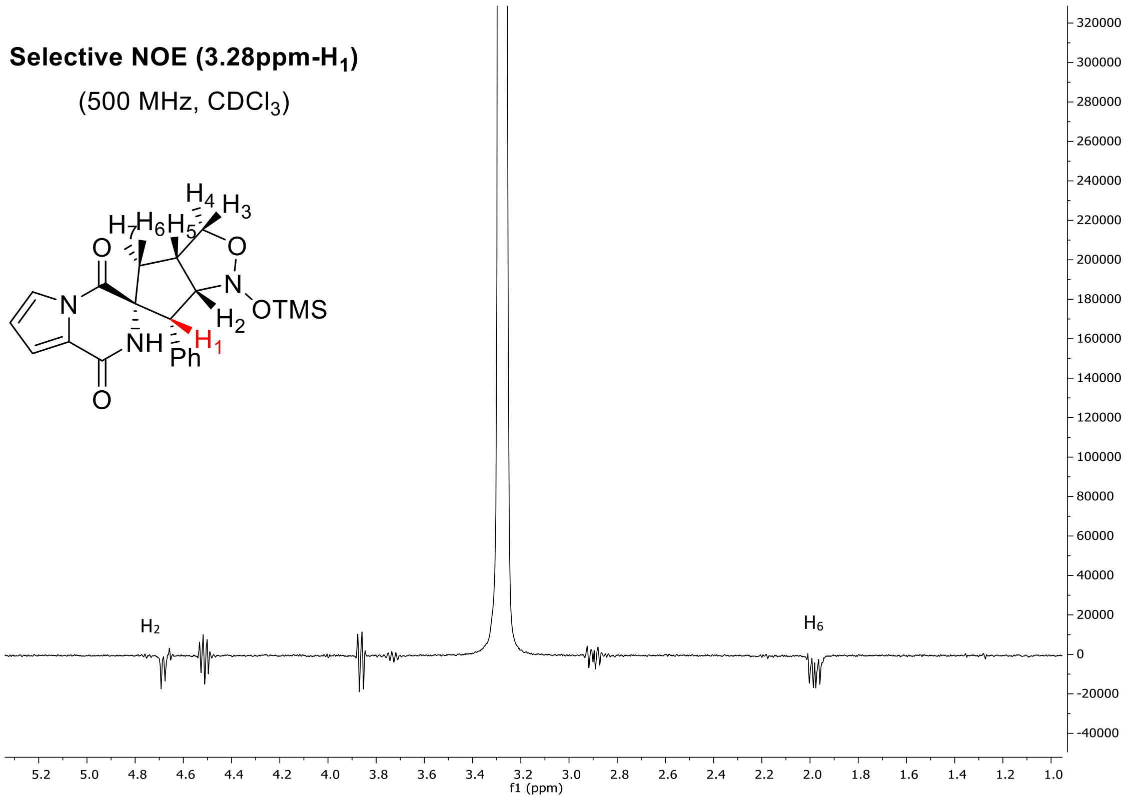
Selective NOE (3.28ppm-H1)
(500 MHz, CDCl3)
H4 H3
H7 H6 H5
O
N
OTMS
H2
NH
Ph
H1
H2
H6
f1 (ppm)

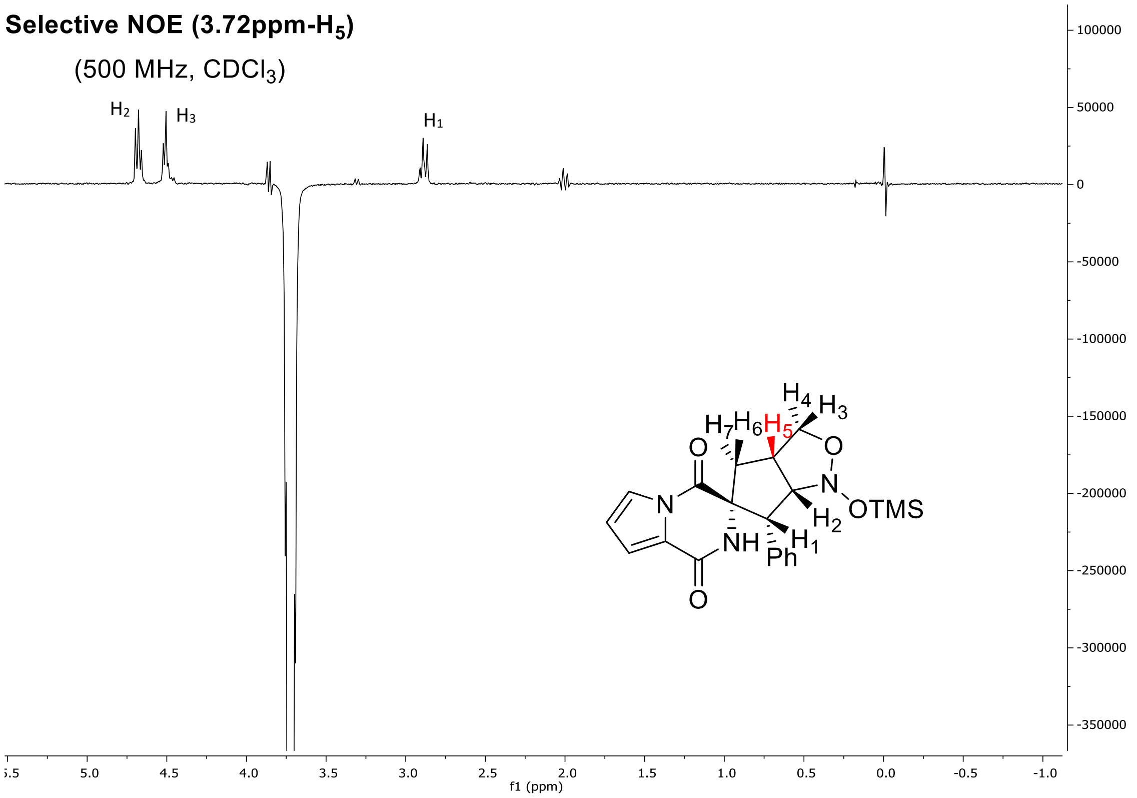
Selective NOE (3.72ppm-H5)
(500 MHz, CDCl3)
H2
H3
H1
H4 H3
H7 H6 H5
O
N
OTMS
H2
NH
Ph
H1
f1 (ppm)

4. HPLC chromatograms for selected compounds

(*S*)-3-Benzyl-1-methoxy-3-((*S*)-2-nitro-1-phenylethyl)pyrrolo[1,2-*a*]pyrazin-4(3*H*)-one (3aa)

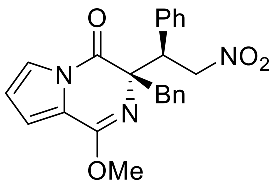

The enantiomeric purity was determined by HPLC analysis (Daicel Chiralpak ID, hexane/isopropanol 90/10, flow rate = 1 mL/min, retention times: 10.8 min (anti), 11.8 min (syn, major.), 12.4 min (syn, minor.) and 16.8 min (anti). Processed chanel Descr.: PDA 210.0 nm).

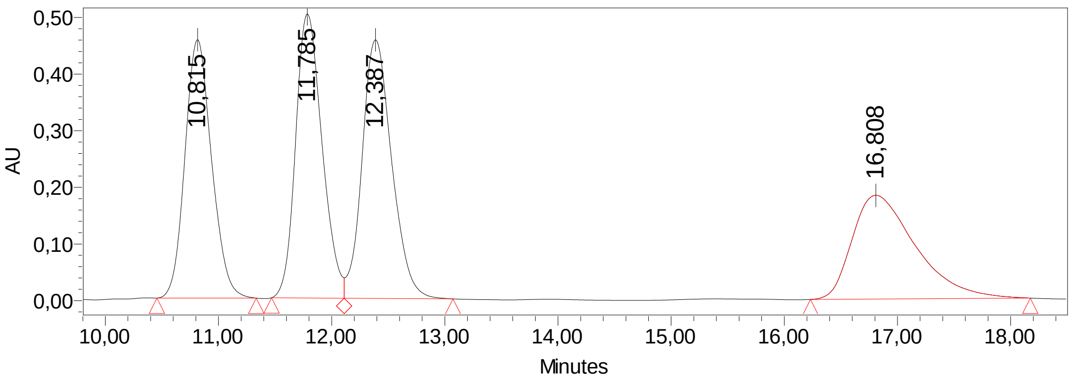

	Retention time	% Area
1	10,815	24,06
2	11,785	26,22
3	12,387	26,79
4	16,808	22,94

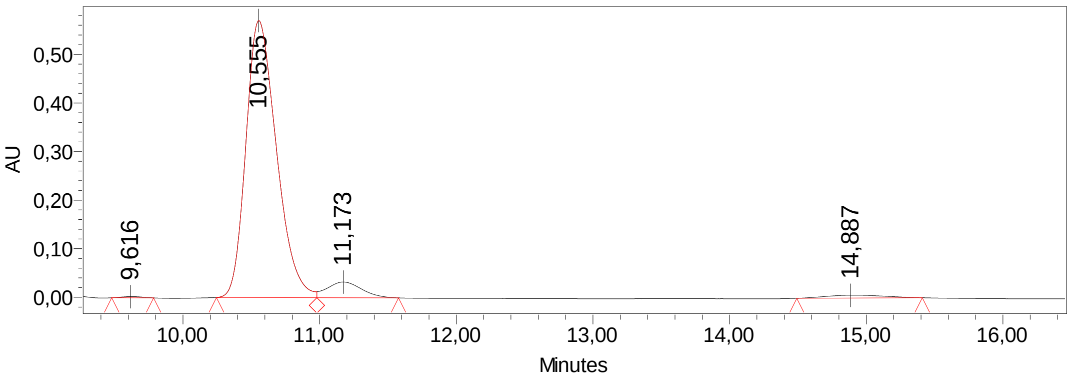

	Retention time	% Area
1	9,616	0,28
2	10,555	91,95
3	11,173	5,93
4	14,887	1,84

(*S*)-3-Benzyl-3-((*S*)-1-(4-bromophenyl)-2-nitroethyl)-1-methoxypyrrolo[1,2-*a*]pyrazin-4(3*H*)-one (3ab)

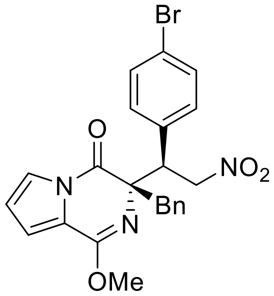

The enantiomeric purity was determined by HPLC analysis (Lux amylose-3, hexane/isopropanol 90/10, flow rate = 1 mL/min, retention times: 13.4 min (anti), 16.0 min (syn, major.), 17.6 min (anti) and 26.0 min (syn, minor.). Processed chanel Descr.: PDA 210.0 nm).

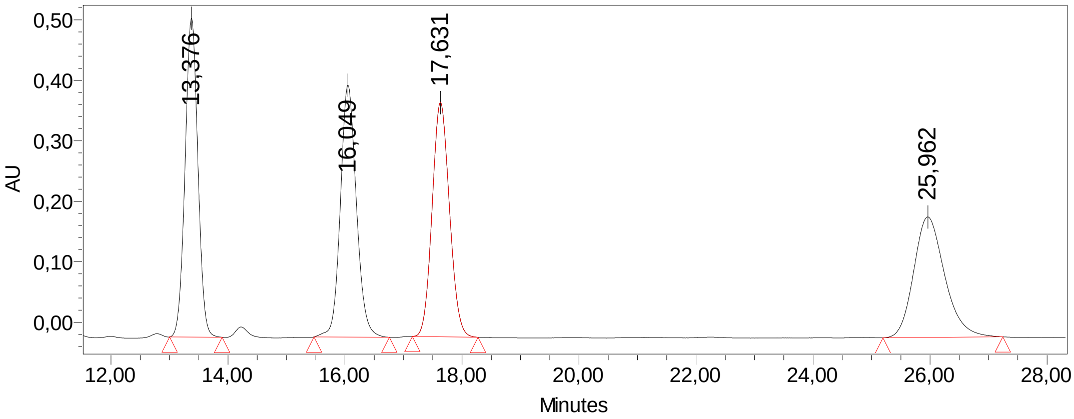

	Retention time	% Area
1	13,376	25,65
2	16,049	25,37
3	17,631	25,53
4	25,962	23,45

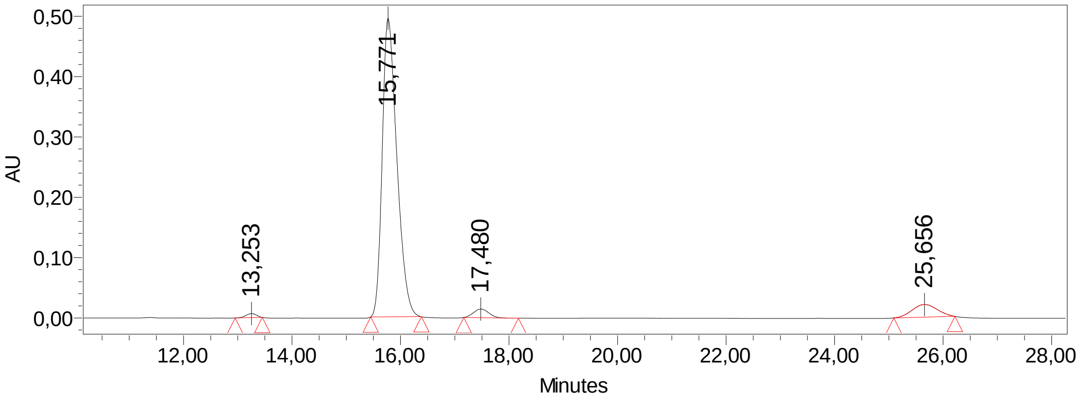

	Retention time	% Area
1	13,253	0,90
2	15,771	89,80
3	17,480	2,73
4	25,656	6,57

(*S*)-3-Benzyl-3-((*S*)-1-(4-fluorophenyl)-2-nitroethyl)-1-methoxypyrrolo[1,2-*a*]pyrazin-4(3*H*)-one (3ac)

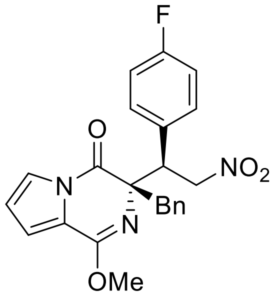

The enantiomeric purity was determined by HPLC analysis (Lux amylose-3), hexane/isopropanol 90/10, flow rate = 1 mL/min, retention times: 10.8 min (anti),12.4 min (syn, major.), 14.7 min (anti) and 19.0 min (syn, minor.). Processed chanel Descr.: PDA 210.0 nm).

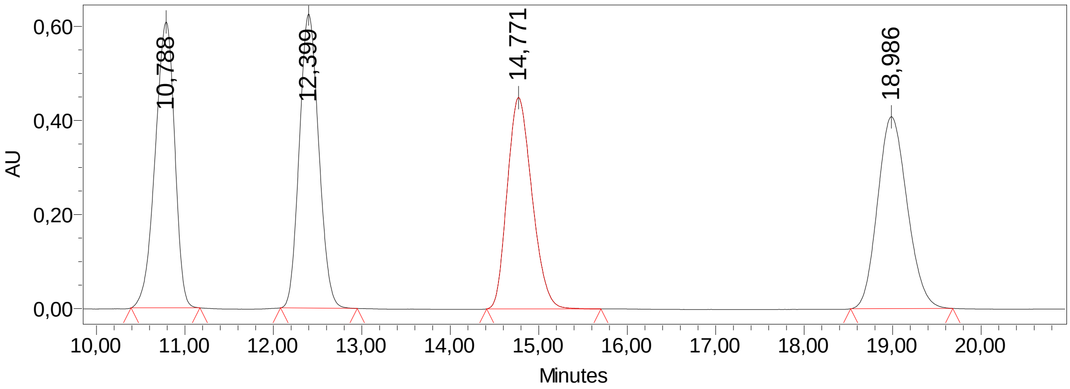

	Retention time	% Area
1	10,788	25,10
2	12,399	25,67
3	14,771	23,61
4	18,986	25,62

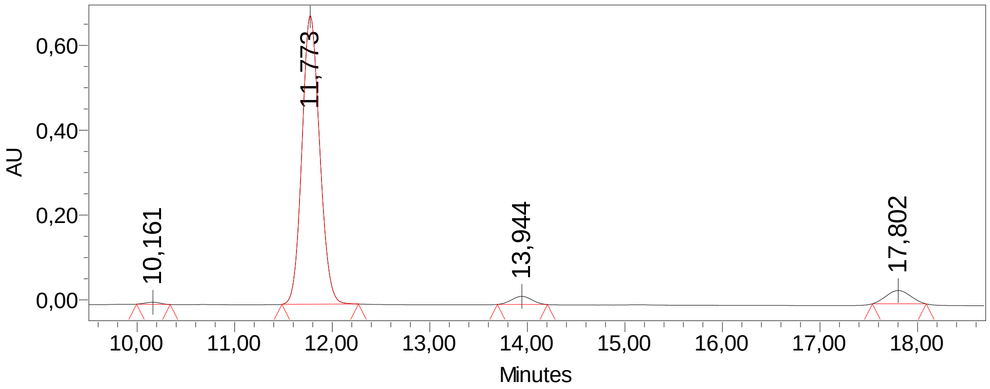

	Retention time	% Area
1	10,161	0,56
2	11,773	90,86
3	13,944	2,90
4	17,802	5,68

(*S*)-3-Benzyl-3-((*S*)-1-(2-chlorophenyl)-2-nitroethyl)-1-methoxypyrrolo[1,2-*a*]pyrazin-4(3*H*)-one (3ad)

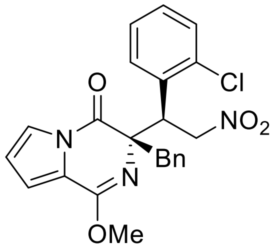

The enantiomeric purity was determined by HPLC analysis (Lux amylose-3), hexane/isopropanol 90/10, flow rate = 1 mL/min, retention times: 13.6 min (minor.) and 15.1 min (major.) Processed chanel Descr.: PDA 210.0 nm).

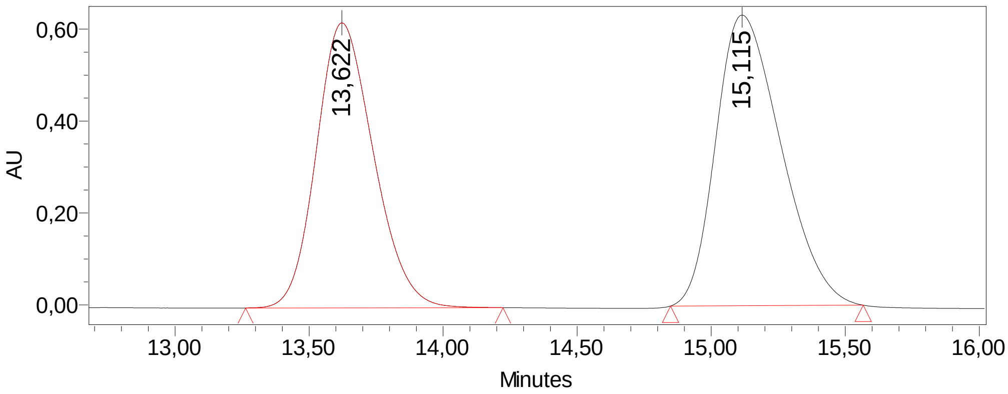

	Retention time	% Area
1	13,622	46,23
2	15,115	53,77

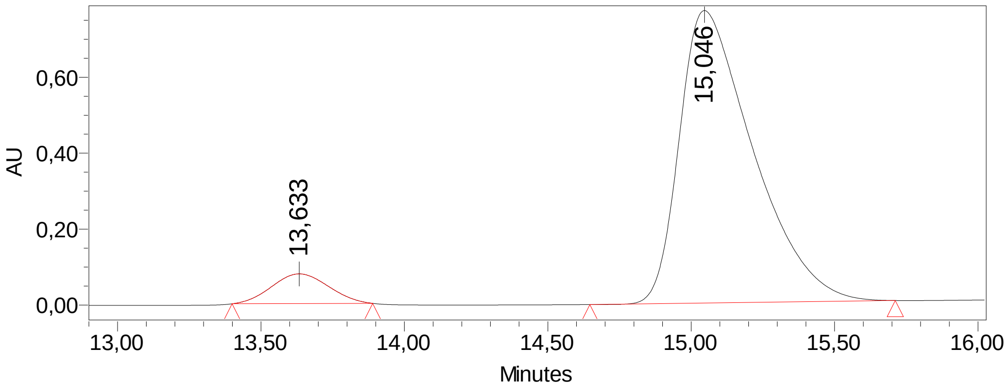

	Retention time	% Area
1	13,633	7,28
2	15,046	92,72

(*S*)-3-Benzyl-3-((*S*)-1-(2,4-dibromo-5-methoxyphenyl)-2-nitroethyl)-1-methoxypyrrolo[1,2-*a*]pyrazin-4(3*H*)-one (3ae)

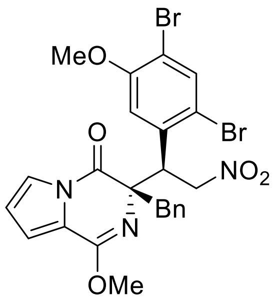

The enantiomeric purity was determined by HPLC analysis (Lux amylose-3), hexane/isopropanol 90/10, flow rate = 1 mL/min, retention times: 16.2 min (syn, minor.) 16.8 min (anti), 17.5 (syn, mayor. and 19.9 min (anti). Processed chanel Descr.: PDA 210.0 nm).

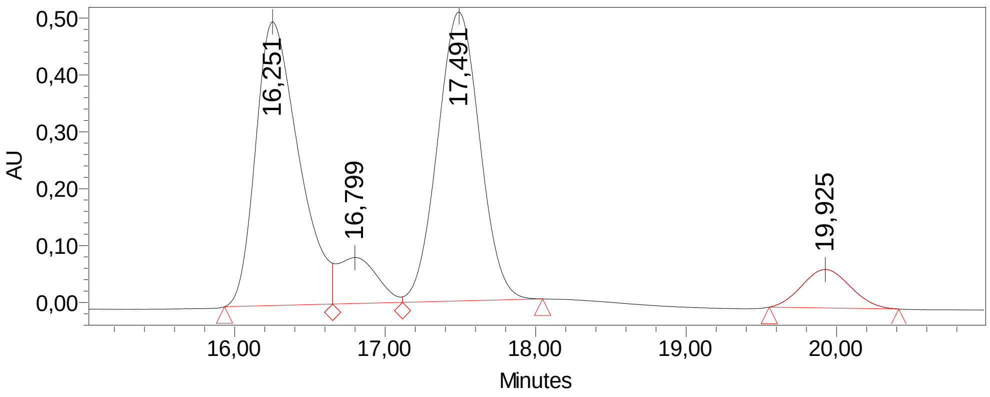

	Retention time	% Area
1	16,251	43,41
2	16,799	6,61
3	17,491	43,35
4	19,925	6,64

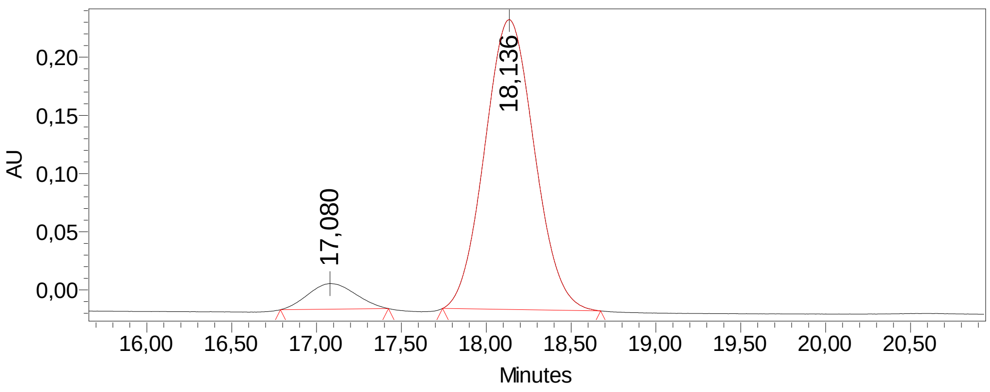

	Retention time	% Area
1	17,080	7,72
2	18,136	92,28

(*S*)-3-Benzyl-3-((*S*)-1-(furan-2-yl)-2-nitroethyl)-1-methoxypyrrolo[1,2-*a*]pyrazin-4(3*H*)-one (3af)

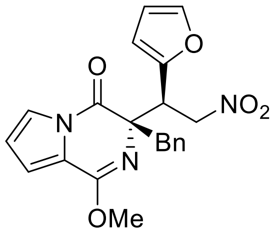

The enantiomeric purity was determined by HPLC analysis (Daicel Chiralpak IC), hexane/isopropanol 90/10, flow rate = 1 mL/min, retention times: 12.3 min (major.) and 25.5 min (minor.). Processed chanel Descr.: PDA 210.0 nm).

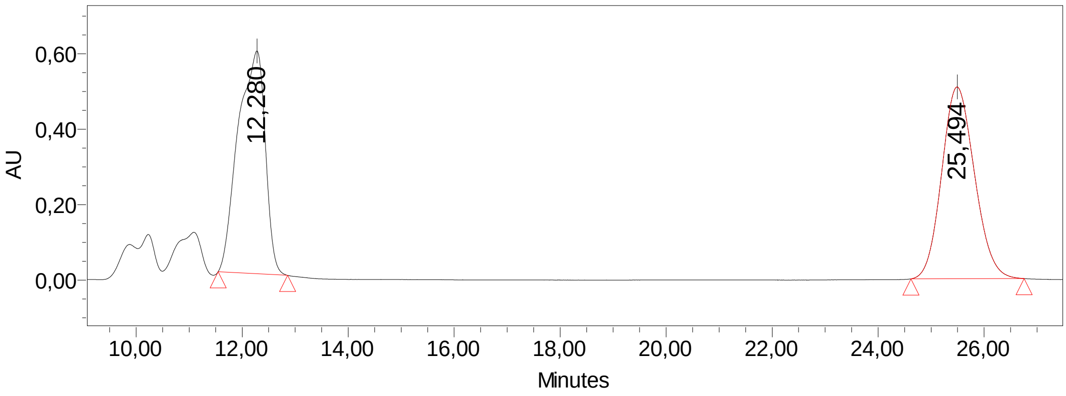

	Retention time	% Area
1	12,280	48,95
2	25,494	51,05

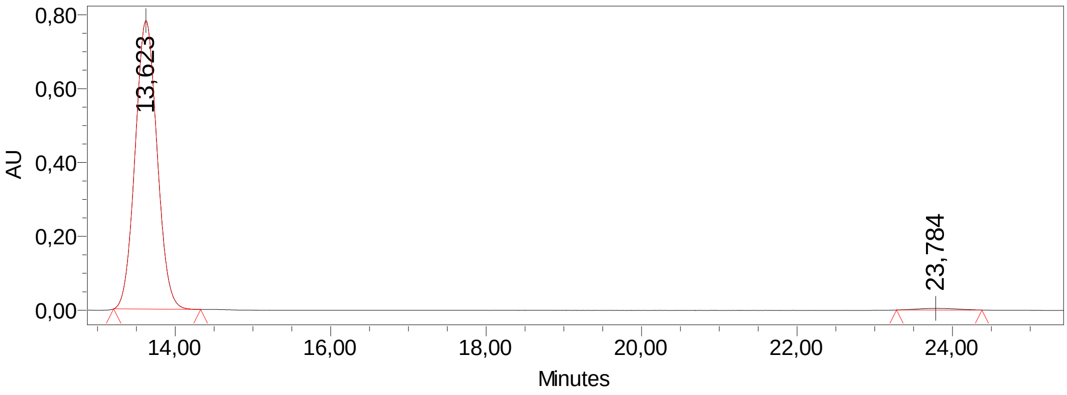

	Retention time	% Area
1	13,623	98,98
2	23,784	1,02

(*S*)-3-Benzyl-3-((*S*)-1-(furan-3-yl)-2-nitroethyl)-1-methoxypyrrolo[1,2-*a*]pyrazin-4(3*H*)-one (3ag)

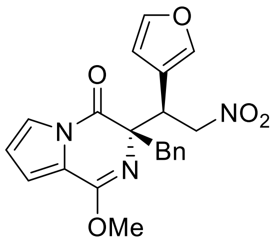

The enantiomeric purity was determined by HPLC analysis (Lux amylose-3), hexane/isopropanol 90/10, flow rate = 1 mL/min, retention times: 10.3 min (anti), 11.6 min (syn, major.) and 13.3 min (syn, minor.) and 14.1 min (anti). Processed chanel Descr.: PDA 210.0 nm).

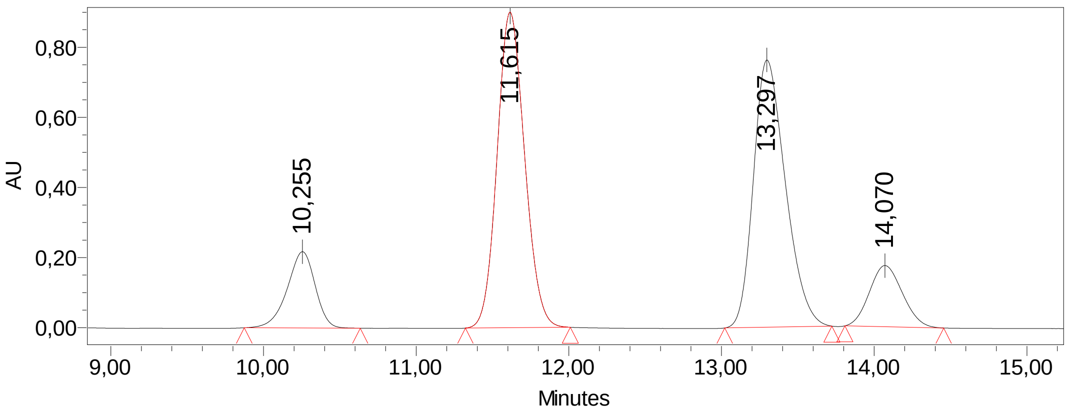

	Retention time	% Area
1	10,255	9,98
2	11,615	40,58
3	13,297	40,00
4	14,070	9,43

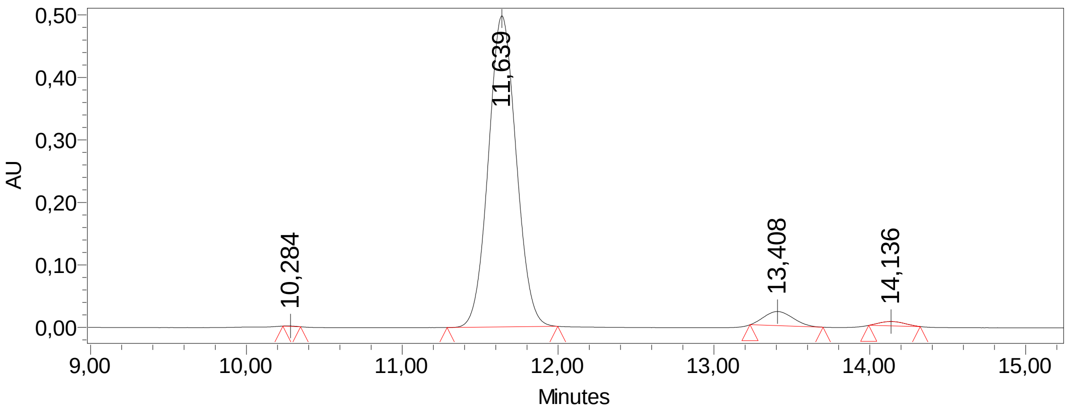

	Retention time	% Area
1	10,284	0,04
2	11,639	94,31
3	13,408	4,45
4	14,136	1,20

(*S*)-3-Benzyl-1-methoxy-3-((*S*)-2-nitro-1-(thiophen-2-yl)ethyl)pyrrolo[1,2-*a*]pyrazin-4(3*H*)-one (3ah)

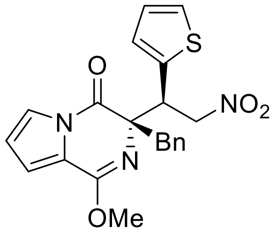

The enantiomeric purity was determined by HPLC analysis (Lux amylose-3), hexane/isopropanol 90/10, flow rate = 1 mL/min, retention times: 11.5 min (syn, major.), 11.8 min (anti), 15.6 min (anti) and 17.1 min (syn, minor.). Processed chanel Descr.: PDA 210.0 nm).

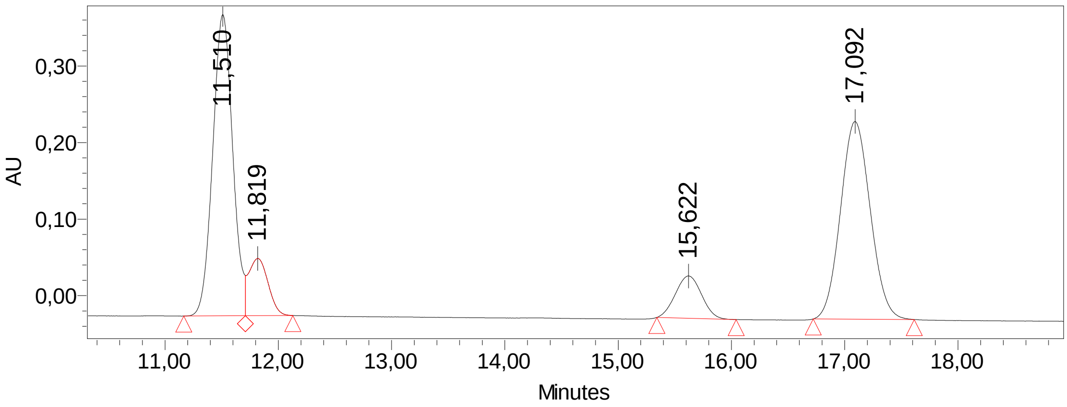

	Retention time	% Area
1	11,510	42,64
2	11,819	7,93
3	15,622	7,82
4	17,092	41,61

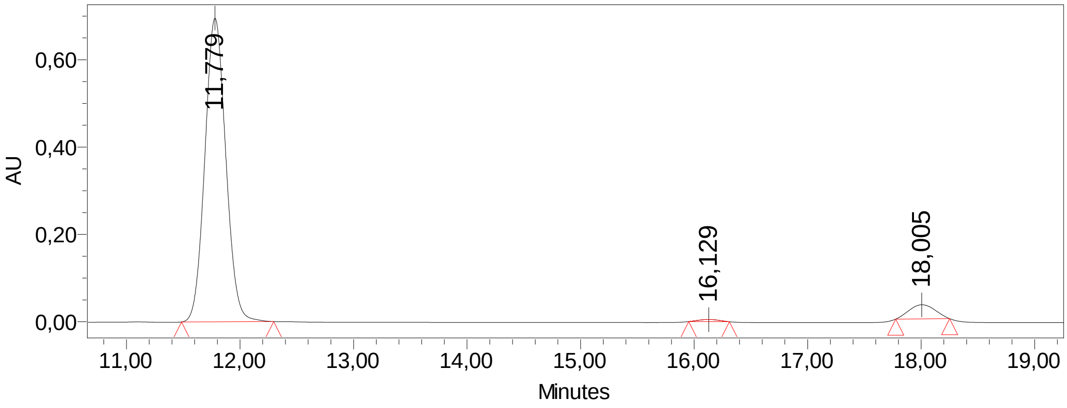

	Retention time	% Area
1	11,779	93,92
2	16,129	0,64
3	18,005	5,44

(*S*)-3-Benzyl-3-((*S*)-1-cyclohexyl-2-nitroethyl)-1-methoxypyrrolo[1,2-*a*]pyrazin-4(3*H*)-one (3ai)

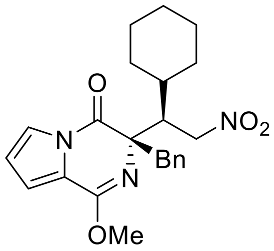

The enantiomeric purity was determined by HPLC analysis (Lux amylose-3), hexane/isopropanol 90/10, flow rate = 1 mL/min, retention times: 16.2 min (major.) and 28.7 min (min.). Processed chanel Descr.: PDA 210.0 nm).

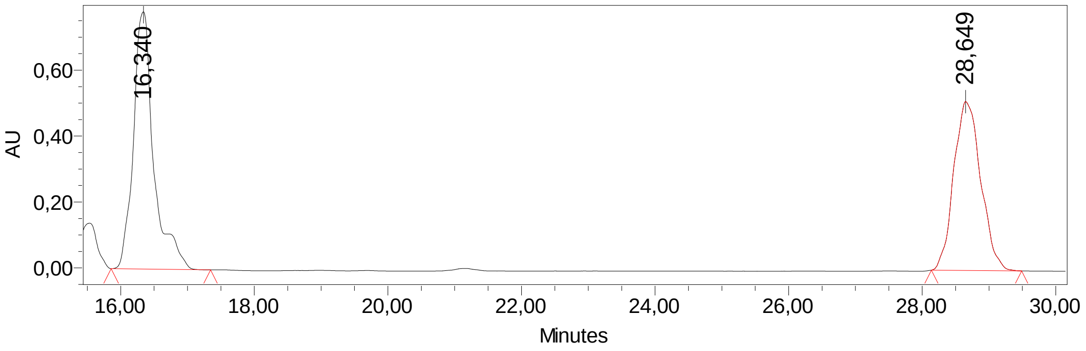

	Retention time	% Area
1	16,340	53,31
2	28,649	46,69

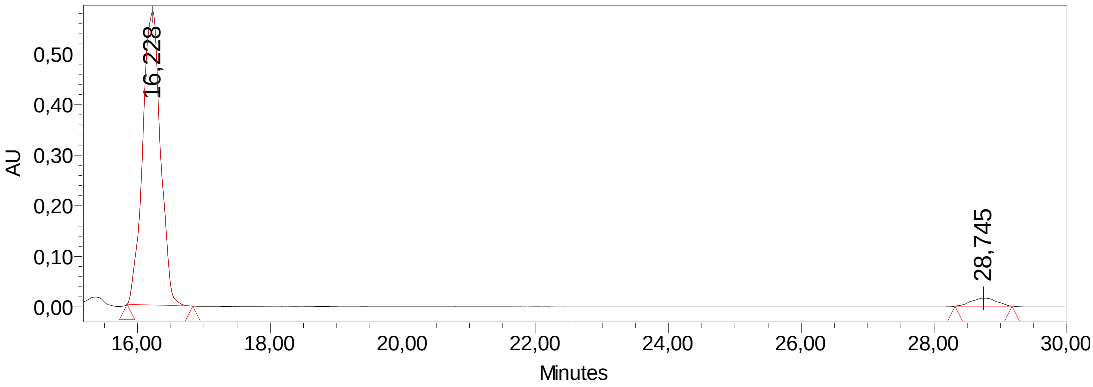

	Retention time	% Area
1	16,228	96,47
2	28,745	3,53

(*S*)-3-Isobutyl-3-((*S*)-2-nitro-1-phenylethyl)-2,3-dihydropyrrolo[1,2-*a*]pyrazine-1,4-dione (3ba)

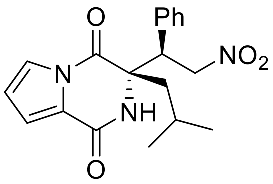

The enantiomeric purity was determined by HPLC analysis (Daicel Chiralpak ID), hexane/isopropanol 90/10, flow rate = 1 mL/min, retention times: 8.7 min (anti), 9.6 min (syn, major.), 10.5 min (anti) and 11.2 min (syn, minor.). Processed chanel Descr.: PDA 210.0 nm).

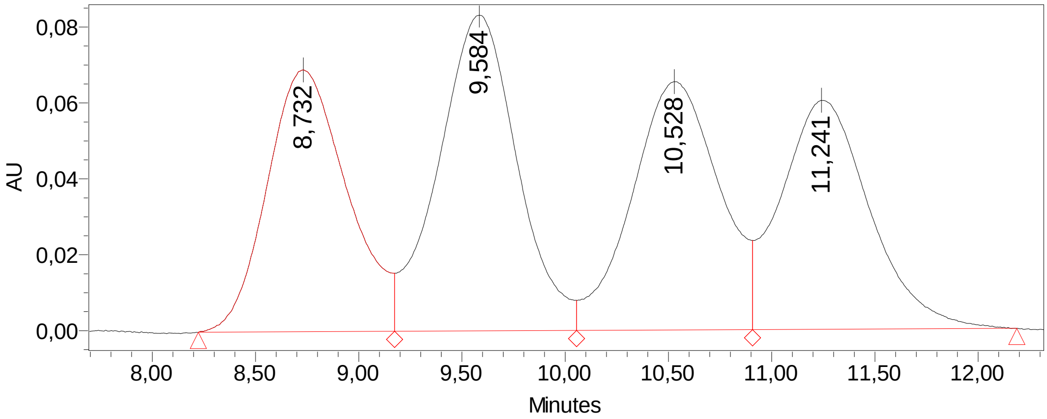

	Retention time	% Area
1	8,732	23,82
2	9,584	28,29
3	10,528	24,67
4	11,241	23,22

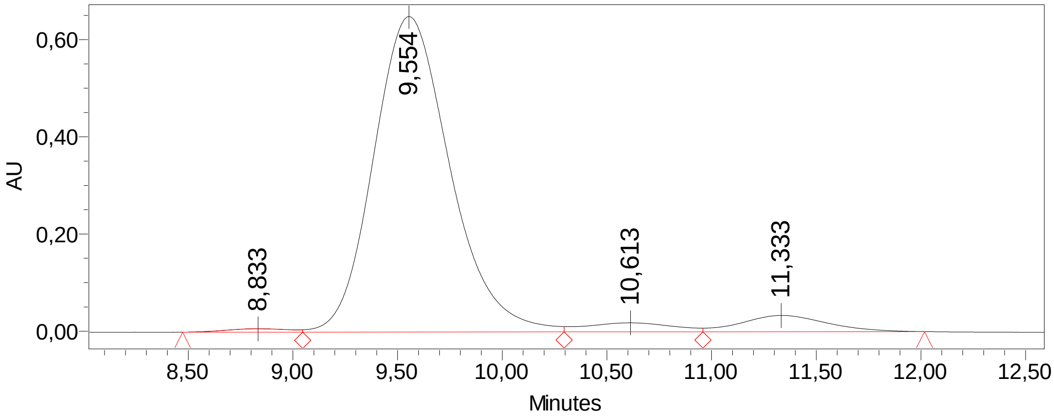

	Retention time	% Area
1	8,833	0,80
2	9,554	90,96
3	10,613	2,99
4	11,333	5,26

(*S*)-3-((*S*)-1-(4-Bromophenyl)-2-nitroethyl)-3-isobutyl-1-methoxypyrrolo[1,2-*a*]pyrazin-4(3*H*)-one (3bb)

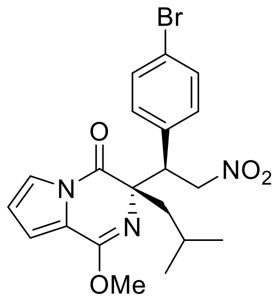

The enantiomeric purity was determined by HPLC analysis (Daicel Chiralpak OD-H), hexane/isopropanol 98/2, flow rate = 1 mL/min, retention times: 9.7 min (anti), 12.5 min (syn, minor.), 22.1 min (anti) and 27.0 min (syn, major.) Processed chanel Descr.: PDA 210.0 nm).

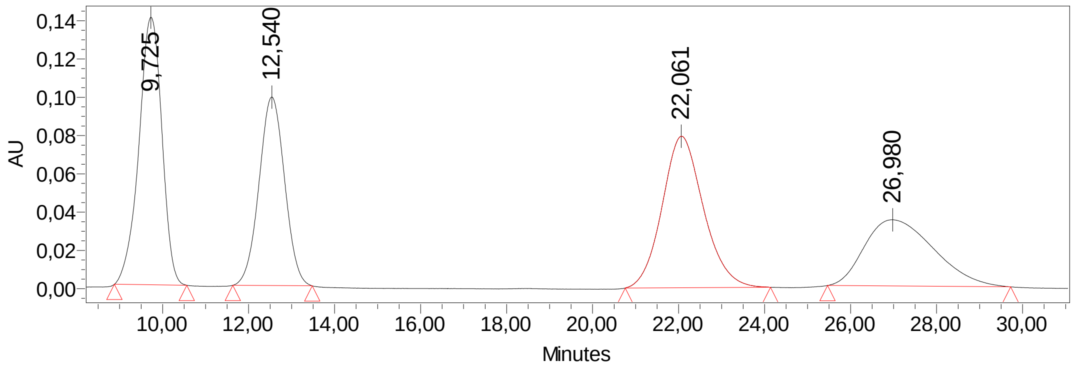

	Retention time	% Area
1	9,725	28,22
2	12,540	22,08
3	22,061	28,66
4	26,980	21,04

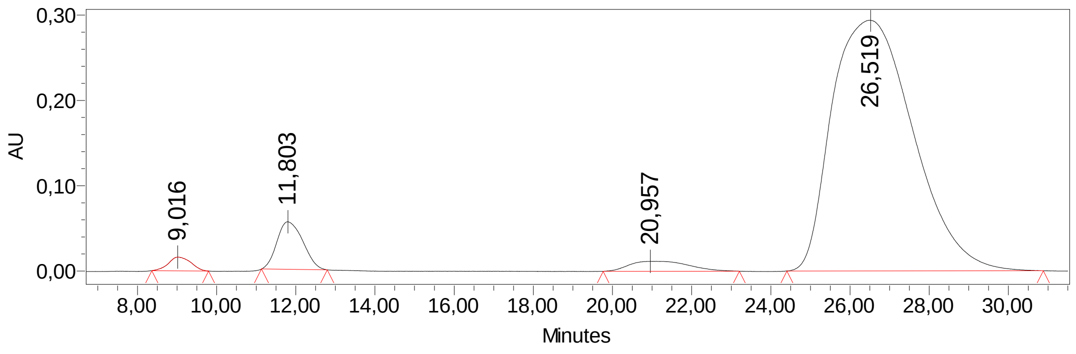

	Retention time	% Area
1	9,016	1,37
2	11,803	5,46
3	20,957	2,63
4	26,519	90,54

(*S*)-3-((*S*)-1-(Furan-2-yl)-2-nitroethyl)-3-isobutyl-1-methoxypyrrolo[1,2-*a*]pyrazin-4(3*H*)-one (3bf)

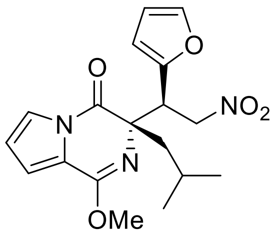

The enantiomeric purity was determined by HPLC analysis (Daicel Chiralpak OD-H), hexane/isopropanol 98/2, flow rate = 1 mL/min, retention times: 6.8 min (anti), 7.6 min (syn, minor.), 8.4 min (syn, major.). Processed chanel Descr.: PDA 210.0 nm).

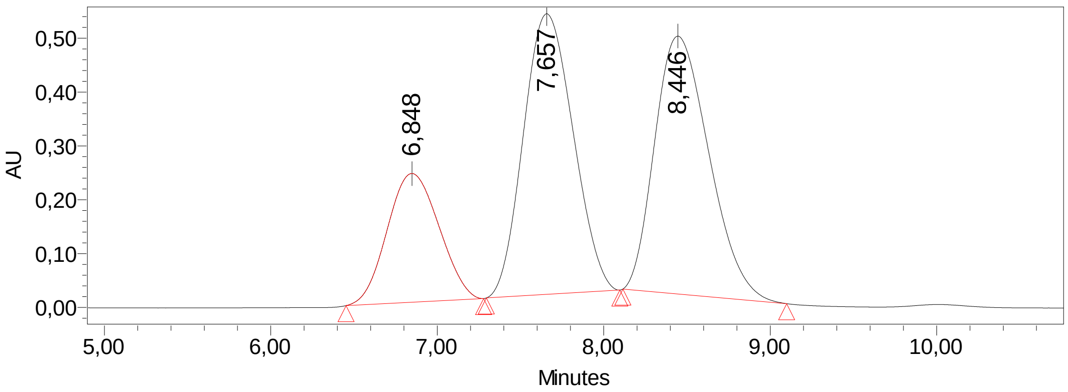

	Retention time	% Area
1	6,848	19,22
2	7,657	40,32
3	8,446	40,46

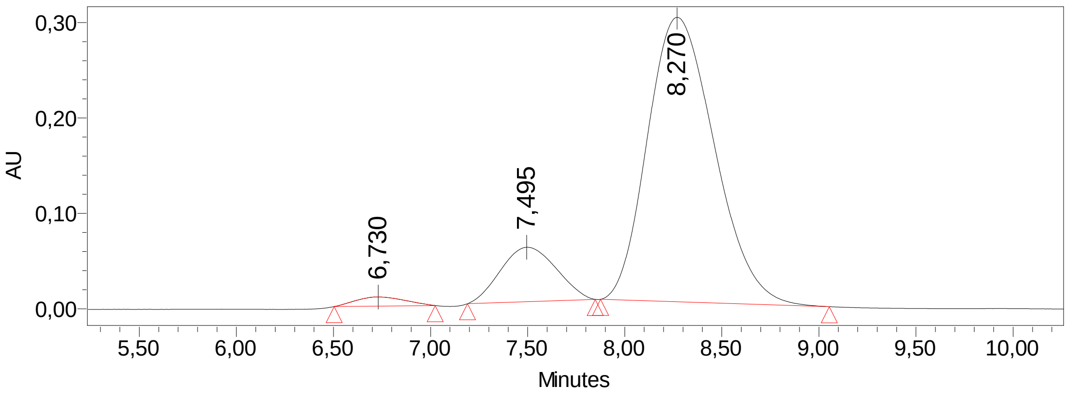

	Retention time	% Area
1	6,730	2,00
2	7,495	13,44
3	8,270	84,56

(*S*)-1-Methoxy-3-(4-methoxybenzyl)-3-((*S*)-2-nitro-1-phenylethyl)pyrrolo[1,2-*a*]pyrazin-4(3*H*)-one (3ca)

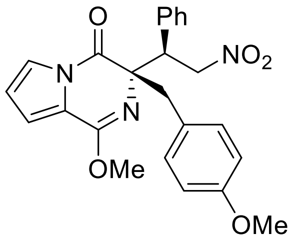

The enantiomeric purity was determined by HPLC analysis (Daicel Chiralpak ID), hexane/isopropanol 90/10, flow rate = 1 mL/min, retention times: 23.5 min (syn, major.), 25.4 min (anti), 28.6 min (syn, minor.) and 45.2 min (anti). Processed chanel Descr.: PDA 210.0 nm).

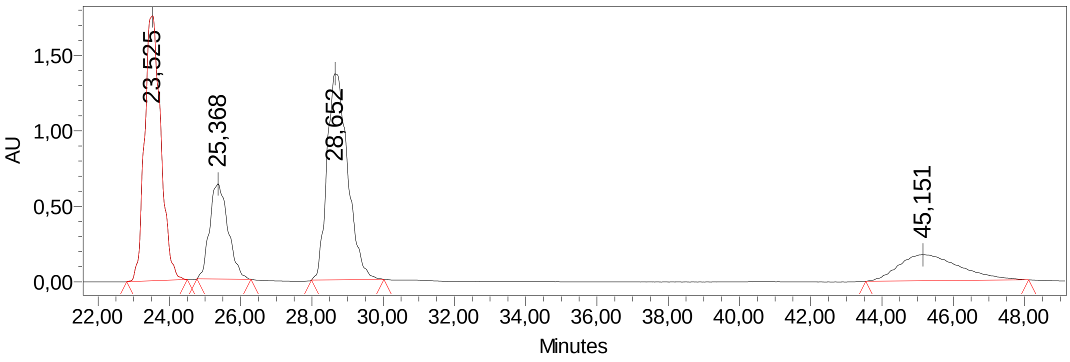

	Retention time	% Area
1	23,525	36,07
2	25,368	14,71
3	28,652	35,89
4	45,151	13,34

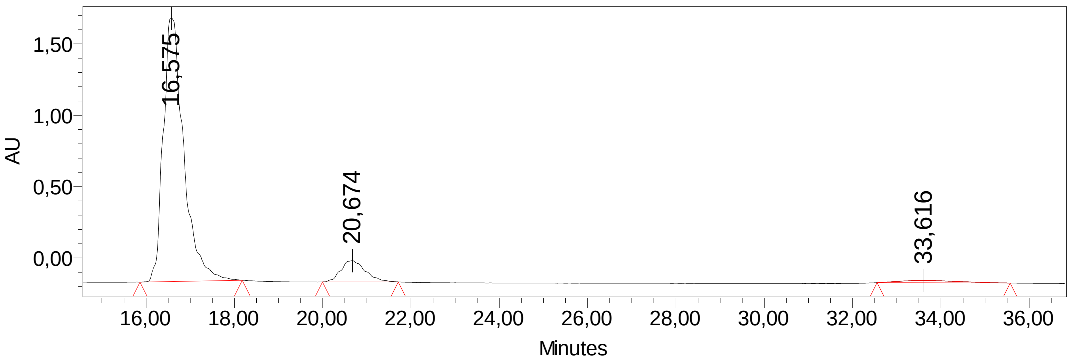

	Retention time	% Area
1	16,575	89,12
2	20,674	8,62
3	33,616	2,26

(*S*)-3-((*S*)-1-(Furan-2-yl)-2-nitroethyl)-1-methoxy-3-(4-methoxybenzyl)pyrrolo[1,2-*a*]pyrazin-4(3*H*)-one (3cf)

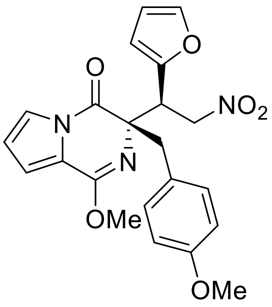

The enantiomeric purity was determined by HPLC analysis (Daicel Chiralpak ID), hexane/isopropanol 90/10, flow rate = 0.5 mL/min, retention times: 25.1 min (syn, major.), 28.4 min (anti), 33.8 min (syn, minor.) and 40.8 min (anti). Processed chanel Descr.: PDA 210.0 nm).

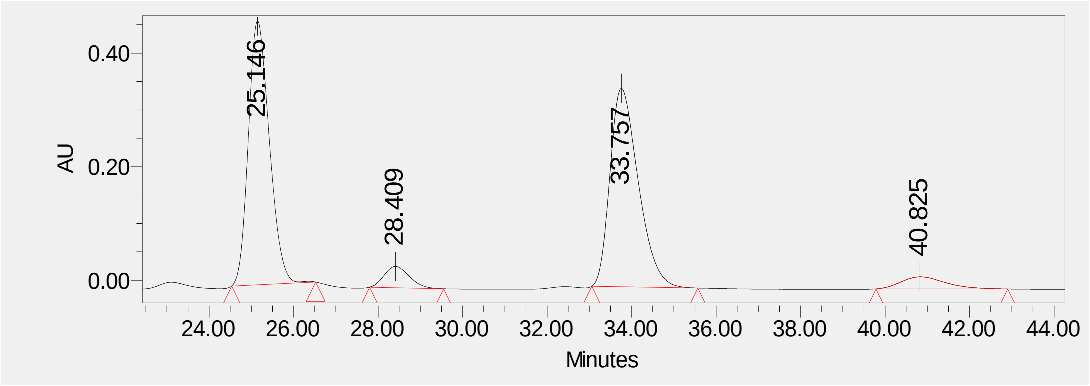

	Retention time	% Area
1	25.146	45.35
2	28.409	4.38
3	33.757	45.87
4	40.825	4.40

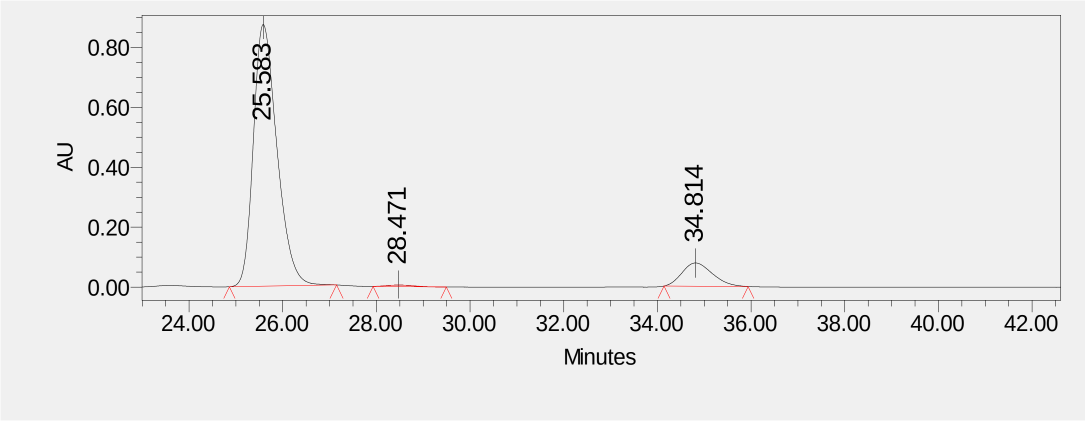

	Retention time	% Area
1	25.583	89.23
2	28.471	0.58
3	34.814	10.20

(*S*)-1-Methoxy-3-(4-methoxybenzyl)-3-((*S*)-2-nitro-1-(thiophen-2-yl)ethyl)pyrrolo[1,2-*a*]pyrazin-4(3*H*)-one (3ch)

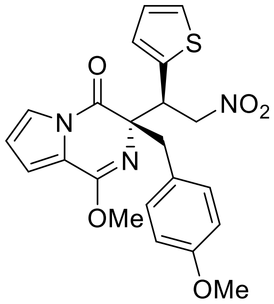

The enantiomeric purity was determined by HPLC analysis (Daicel Chiralpak ID), hexane/isopropanol 90/10, flow rate = 0.5 mL/min, retention times: 23.6 min (syn, major.), 26.0 min (anti), 31.2 min (syn, minor.) and 41.4 min (anti). Processed chanel Descr.: PDA 210.0 nm).

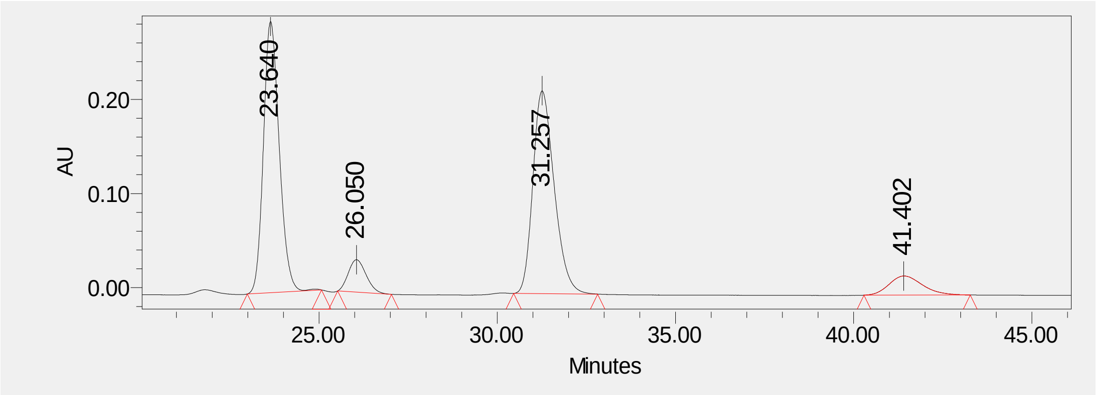

	Retention time	% Area
1	23.640	43.65
2	26.050	5.89
3	31.257	43.96
4	41.402	6.50

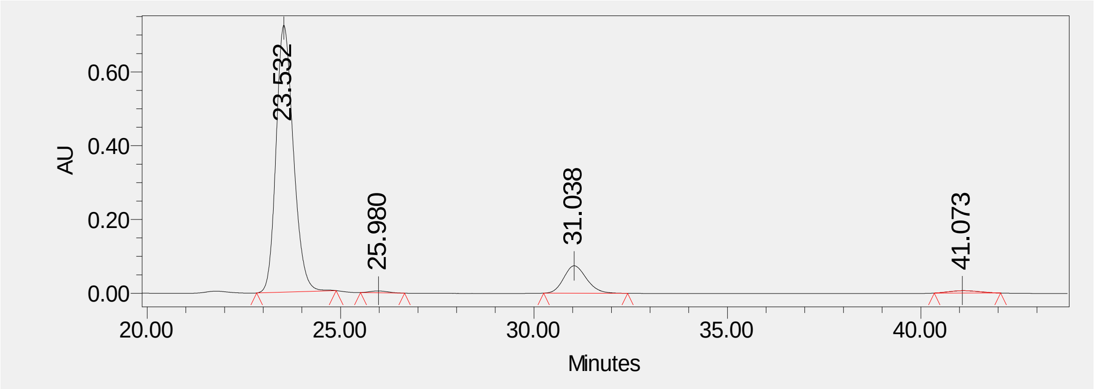

	Retention time	% Area
1	23.532	86.38
2	25.980	0.62
3	31.038	11.67
4	41.073	1.33

(*S*)-1-Methoxy-3-((*S*)-2-nitro-1-phenylethyl)-3-phenethylpyrrolo[1,2-*a*]pyrazin-4(3*H*)-one (3da)

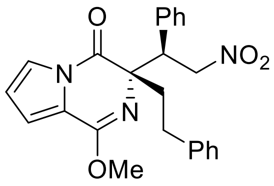

The enantiomeric purity was determined by HPLC analysis (Lux amylose-3), hexane/isopropanol 90/10, flow rate = 1 mL/min, retention times: 9.2 min (syn, minor.), 12.5 min (syn, major.), 13.4 min (anti) and 19.6 min (anti). Processed chanel Descr.: PDA 210.0 nm).

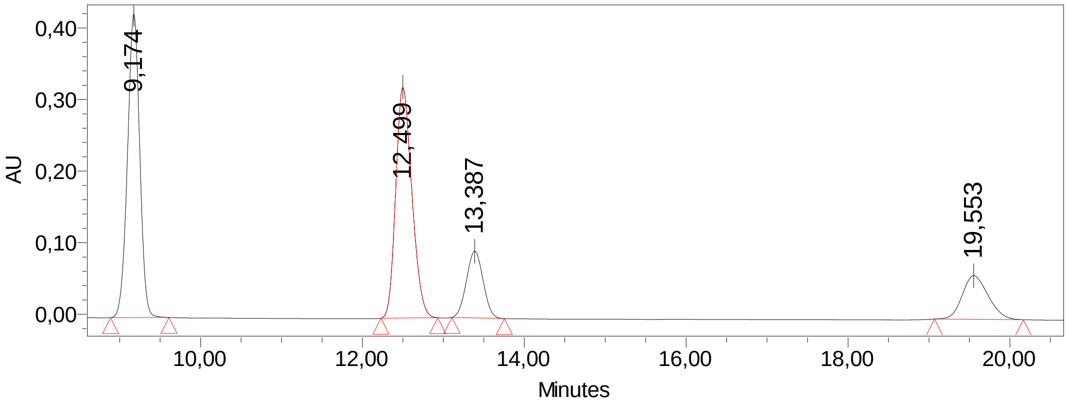

	Retention time	% Area
1	9,174	38,35
2	12,499	38,49
3	13,387	11,50
4	19,553	11,66

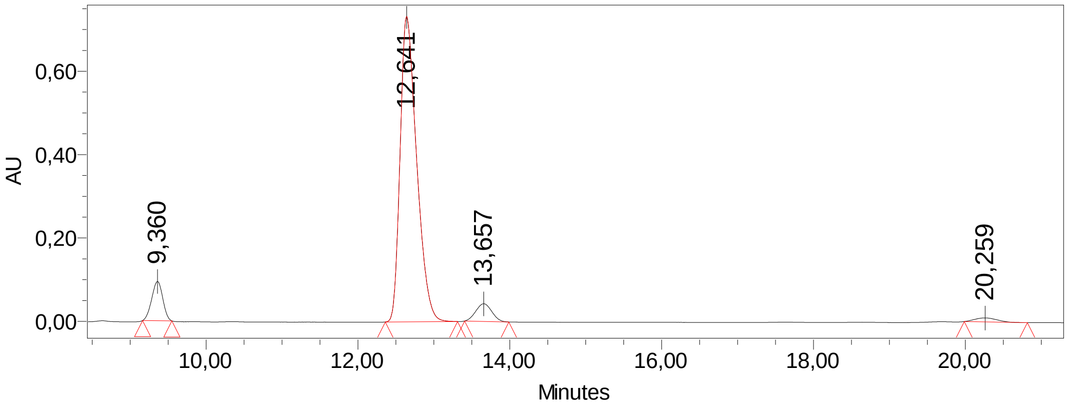

	Retention time	% Area
1	9,360	7,40
2	12,641	86,26
3	13,657	4,84
4	20,259	1,50

(*S*)-3-((*S*)-1-(Furan-3-yl)-2-nitroethyl)-1-methoxy-3-phenethylpyrrolo[1,2-*a*]pyrazin-4(3*H*)-one (3dg)

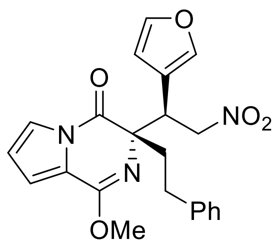

The enantiomeric purity was determined by HPLC analysis (Lux amylose-3), hexane/isopropanol 90/10, flow rate = 1 mL/min, retention times: 14.0 min (syn, minor.), 18.8 min (syn, major.), 19.4 min (anti) and 26.1 min (anti). Processed chanel Descr.: PDA 210.0 nm).

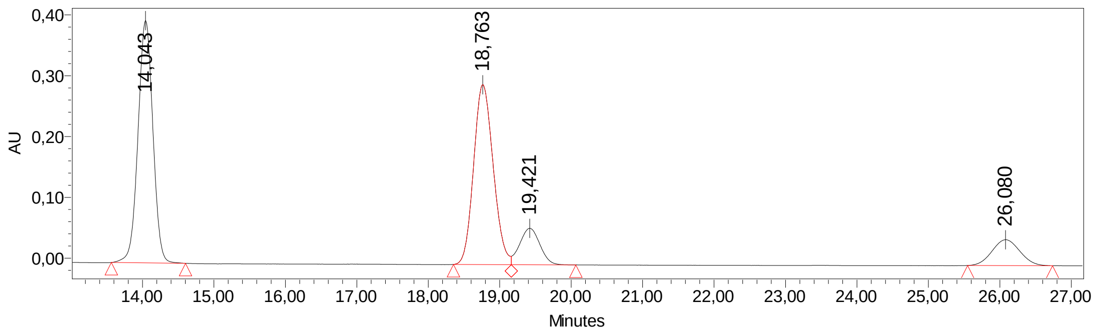

	Retention time	% Area
1	14,043	41,98
2	18,763	41,09
3	19,421	8,57
4	26,080	8,36

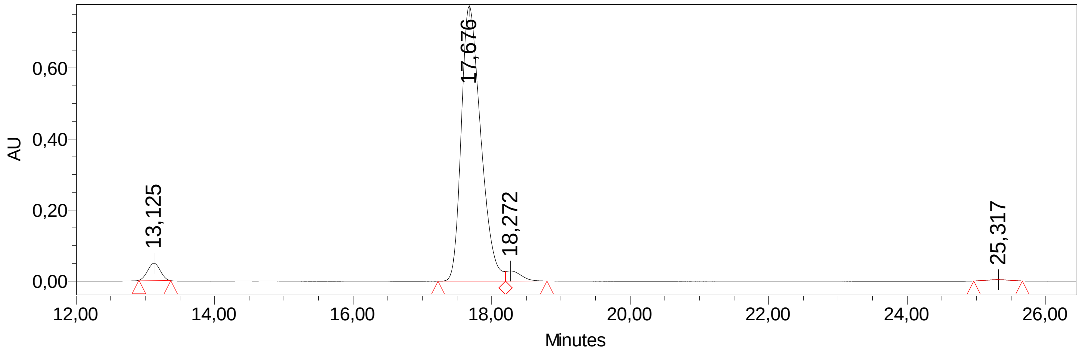

	Retention time	% Area
1	13,125	3,73
2	17,676	93,13
3	18,272	2,62
4	25,317	0,52

(*S*)-3-((*S*)-1-Cyclohexyl-2-nitroethyl)-1-methoxy-3-phenethylpyrrolo[1,2-*a*]pyrazin-4(3*H*)-one (3di)

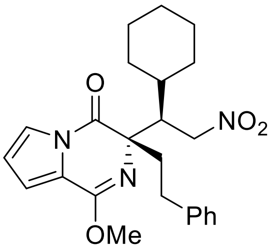

The enantiomeric purity was determined by HPLC analysis (Lux amylose-3), hexane/isopropanol 90/10, flow rate = 1 mL/min, retention times: 10.2 min (anti), 10.9 min (syn, minor.), 11.3 min (syn, major.) and 11.8 min (anti). Processed chanel Descr.: PDA 210.0 nm).

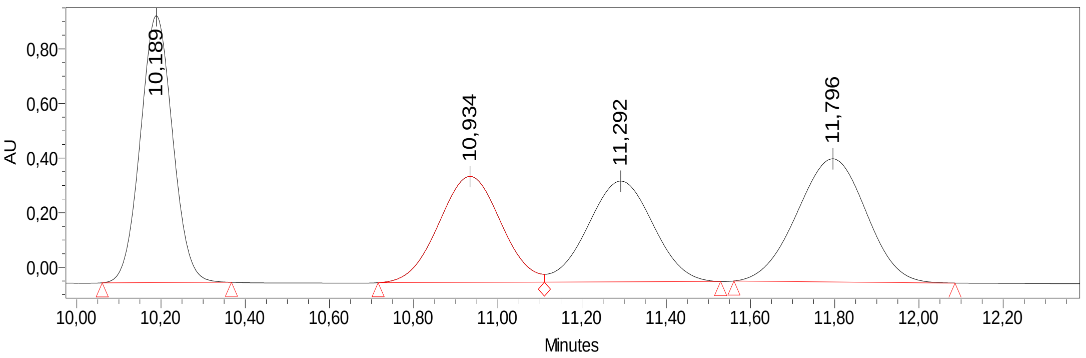

	Retention time	% Area
1	10,189	28,47
2	10,934	21,72
3	11,292	21,71
4	11,796	28,09

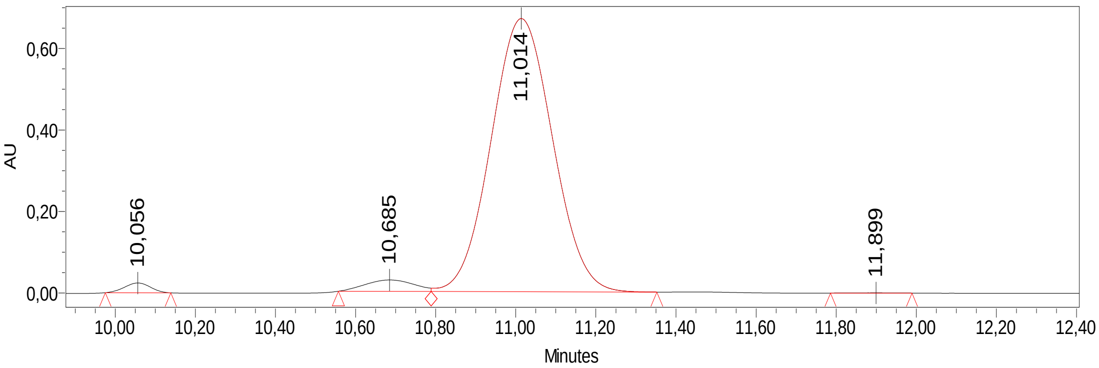

	Retention time	% Area
1	10,056	1,44
2	10,685	3,14
3	11,014	95,38
4	11,899	0,04

(*S*)-3-((1*H*-Indol-3-yl)methyl)-1-methoxy-3-((*S*)-2-nitro-1-phenylethyl)pyrrolo[1,2-*a*]pyrazin-4(3*H*)-one (3ea)

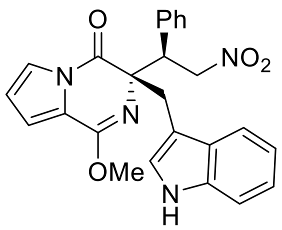

The enantiomeric purity was determined by HPLC analysis (Lux amylose-3), hexane/isopropanol 80/20, flow rate = 1 mL/min, retention times: 10.4 min (syn, major.), 10.7 min (anti), 15.6 min (syn, minor.) and 16.1 min (anti). Processed chanel Descr.: PDA 210.0 nm).

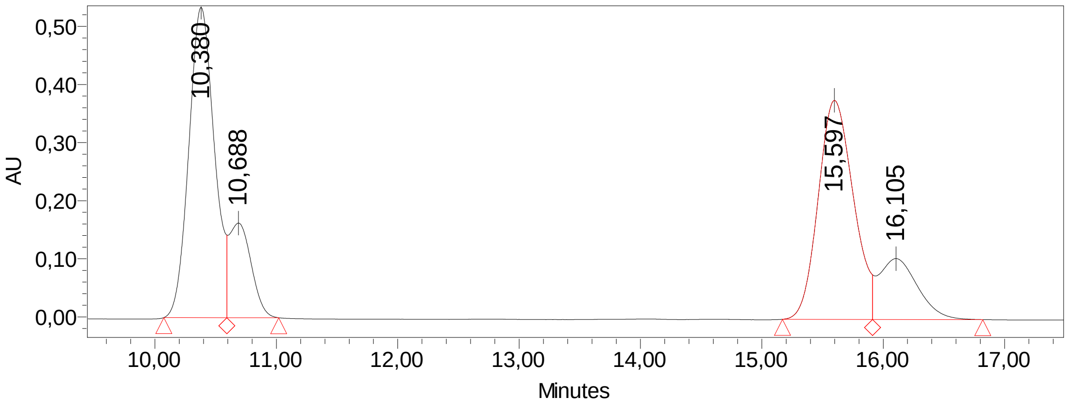

	Retention time	% Area
1	10,380	39,30
2	10,688	10,51
3	15,597	38,33
4	16,105	11,86

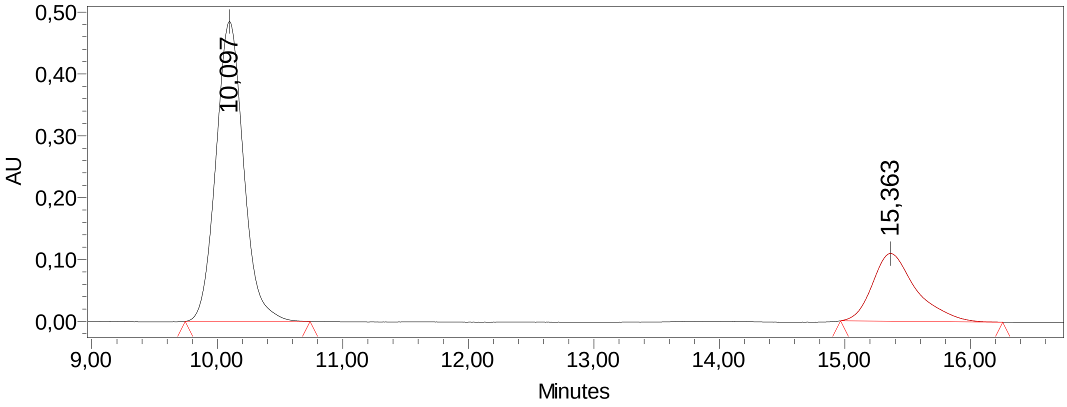

	Retention time	% Area
1	10,097	73,80
2	15,363	26,20

(*S*)-3-Allyl-1-methoxy-3-((*S*)-2-nitro-1-phenylethyl)pyrrolo[1,2-*a*]pyrazin-4(3*H*)-one (3fa)

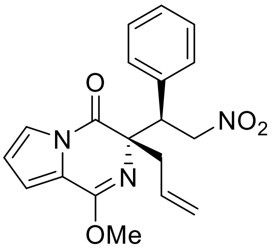

The enantiomeric purity was determined by HPLC analysis (Daicel Chiralpak OD-H), hexane/isopropanol 90/10, flow rate = 1 mL/min, retention times: 7.0 min (anti), 7.7 min (anti), 8.5 min (syn, minor.) and 11.9 min (syn, major.). Processed chanel Descr.: PDA 210.0 nm).

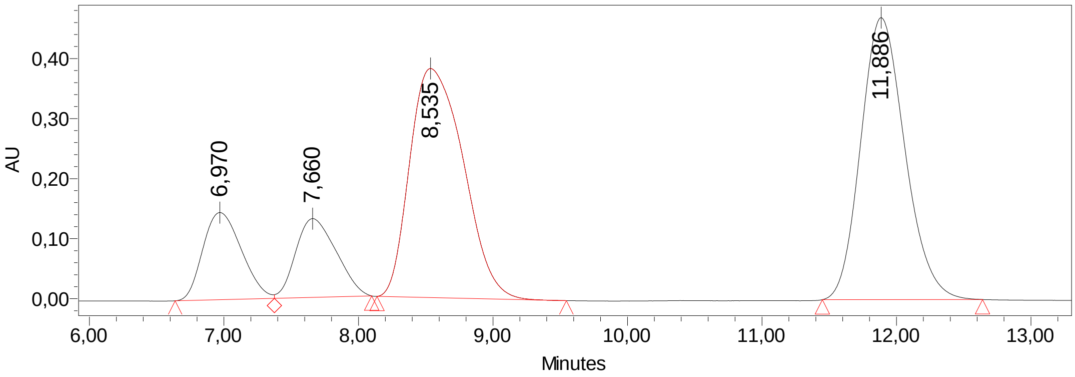

	Retention time	% Area
1	6,970	10,75
2	7,660	10,17
3	8,535	39,28
4	11,886	39,79

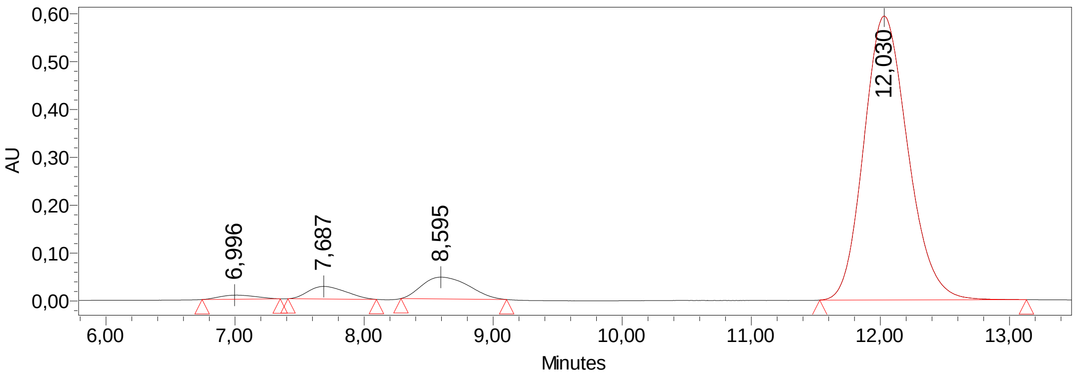

	Retention time	% Area
1	6,996	1,04
2	7,687	3,33
3	8,595	7,22
4	12,030	88,40

(*R*)-3-((*S*)-1-Cyclohexyl-2-nitroethyl)-1-methoxy-3-phenylpyrrolo[1,2-*a*]pyrazin-4(3*H*)-one (3gj)

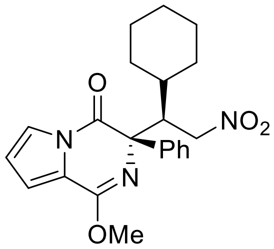

The enantiomeric purity was determined by HPLC analysis (Lux amylose-3), hexane/isopropanol 98/2, flow rate = 0.5 mL/min, retention times: 18.9 min (major.) and 19.7 min (minor.). Processed chanel Descr.: PDA 210.0 nm).

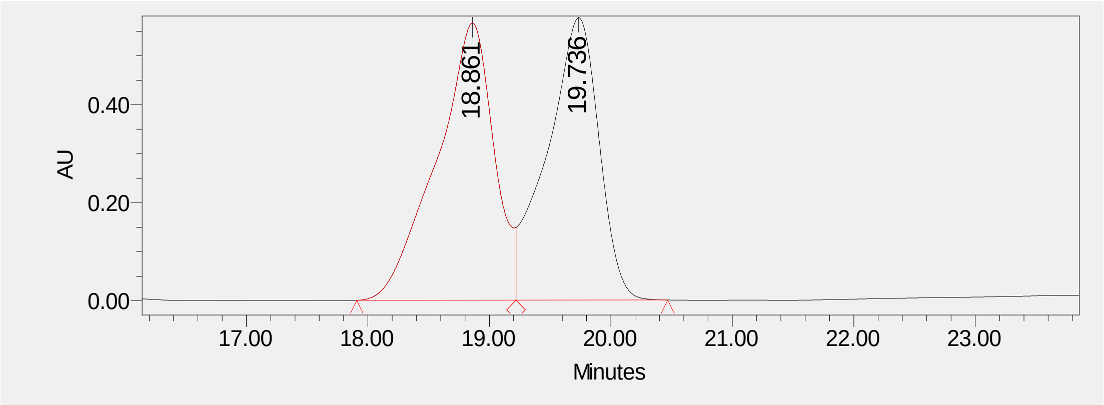

	Retention time	% Area
1	18.861	52.01
2	19.736	47.99

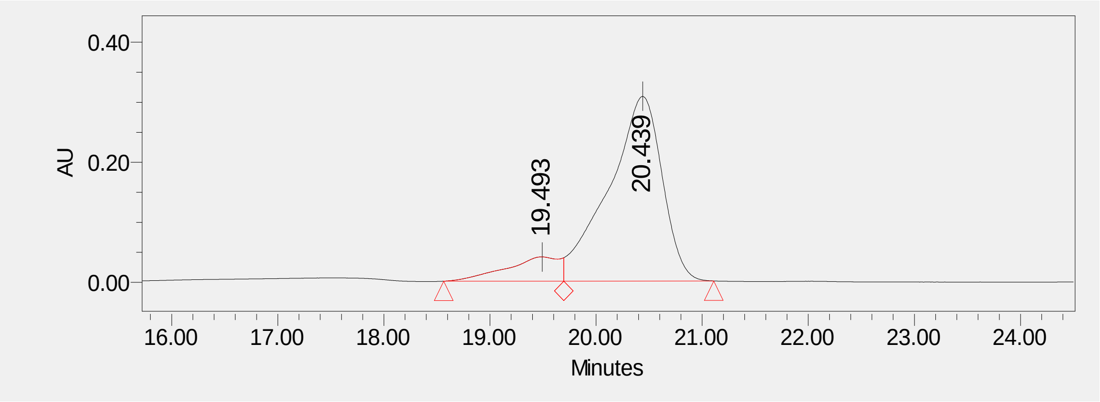

	Retention time	% Area
1	19.493	11.68
2	20.439	88.32

(*S*)-3-Hexyl-3-((*S*)-2-nitro-1-phenylethyl)-2,3-dihydropyrrolo[1,2-*a*]pyrazine-1,4-dione (4ha)

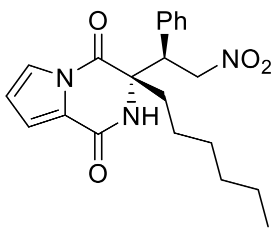

The enantiomeric purity was determined by HPLC analysis (Lux cellulose-5), hexane/isopropanol 95/5, flow rate = 0.5 mL/min, retention times: 25.2 min (anti), 28.5 min (syn, minor.), 29.9 min (anti) and 36.7 min (syn, major.). Processed chanel Descr.: PDA 210.0 nm).

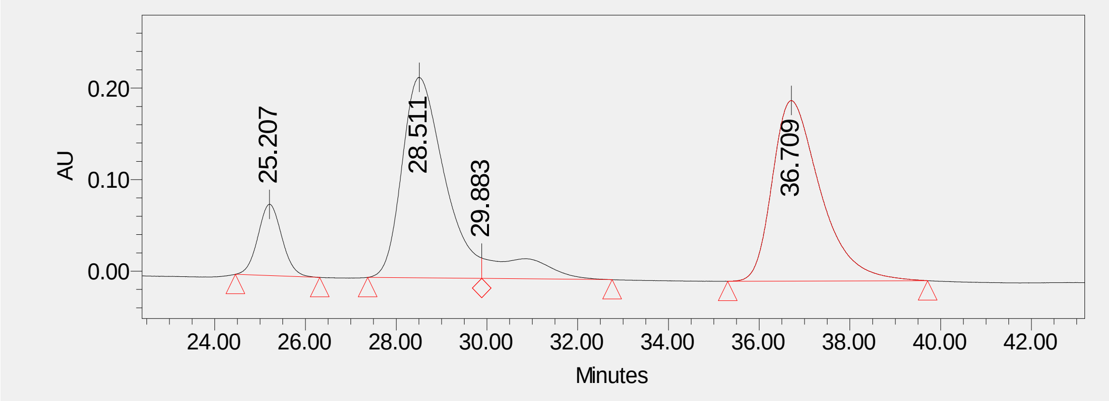

	Retention time	% Area
1	25.207	8.53
2	28.511	42.08
3	29.883	6.14
4	36.709	43.25

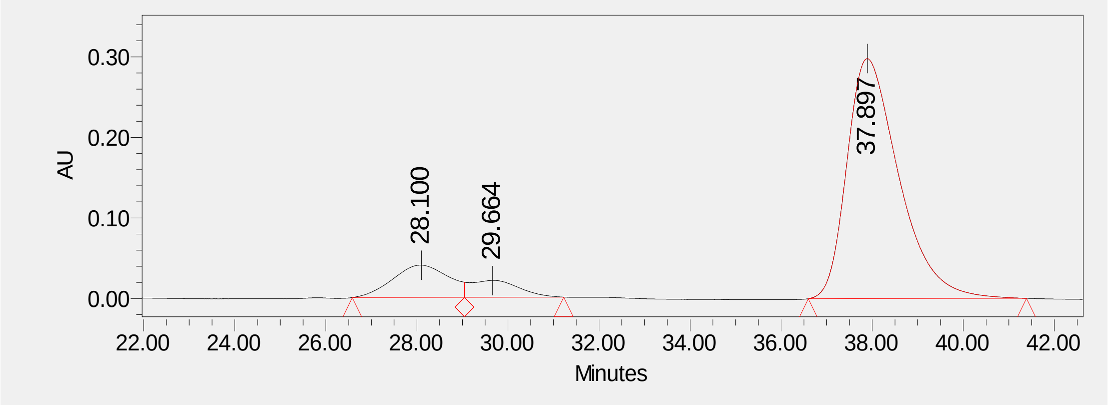

	Retention time	% Area
1	28.100	11.93
2	29.664	5.44
3	37.897	82.63
